# Gut microbiota as a residual risk factor causally influencing cardiac structure and function: Mendelian randomization analysis and biological annotation

**DOI:** 10.3389/fmicb.2024.1410272

**Published:** 2024-07-26

**Authors:** Yihua Li, Meidan Yao, Fei Xie, Yijun Qiu, Xinjun Zhao, Rong Li

**Affiliations:** ^1^The First Clinical Medical College, Guangzhou University of Chinese Medicine, Guangzhou, China; ^2^The First Affiliated Hospital of Guangzhou University of Chinese Medicine, Guangzhou, China; ^3^National Key Laboratory of Chinese Medicine Evidence, Guangzhou, China; ^4^Lingnan Medical Research Center, Guangzhou University of Chinese Medicine, Guangzhou, China

**Keywords:** gut-heart axis, gut microbiota, cardiac structure and function, causal inference, Mendelian randomization

## Abstract

**Background:**

The gut microbiota (GM) is widely acknowledged to have a significant impact on cardiovascular health and may act as a residual risk factor affecting cardiac structure and function. However, the causal relationship between GM and cardiac structure and function remains unclear.

**Objective:**

This study aims to employ a two-sample Mendelian randomization (MR) approach to investigate the causal association between GM and cardiac structure and function.

**Methods:**

Data on 119 GM genera were sourced from a genome-wide association study (GWAS) meta-analysis (13,266 European participants) conducted by the MiBioGen consortium, while data on 16 parameters of cardiac structure and function were obtained from the UK Biobank’s GWAS of cardiac magnetic resonance imaging (up to 41,135 European participants). Inverse variance weighted (IVW), MR-Egger, and weighted median (WM) methods were utilized for causal association assessments, with sensitivity analyses conducted to reinforce the findings. Finally, biological annotation was performed on the GWAS data of GM and cardiac phenotypes with causal associations to explore potential mechanisms.

**Results:**

The MR analysis, predominantly based on the IVW model, revealed 93 causal associations between the genetically predicted abundance of 44 GM genera and 16 cardiac structure and function parameters. These associations maintained consistent directions in MR-Egger and WM models, with no evidence of pleiotropy detected. Biological annotations suggest that GM may influence cardiac structure and function through pathways involved in myocardial cell development, cardiac contractility, and apoptosis.

**Conclusion:**

The MR analysis supports a causal association between certain abundances of genetically predicted GM and cardiac structure and function, suggesting that GM could be a residual risk factor impacting cardiac phenotypes.

## 1 Introduction

Cardiovascular diseases constitute a leading cause of mortality worldwide, posing significant challenges to public health systems (Roth et al., 2017). In the clinical practice of cardiology, the assessment of cardiac structure and function is a focal point for clinicians and researchers alike. Accumulating evidence has increasingly established that an enlargement of cardiac structures and a decline in function are closely associated with poor prognoses in cardiovascular diseases, a consensus that has gradually formed within the field of cardiovascular research (Lee et al., 1993; Sallach et al., 2009; Bourantas et al., 2011; Hoit, 2014; Di Bella et al., 2015; Sabe et al., 2016; Chioncel et al., 2017; Jain et al., 2019; Sanders et al., 2020; Thomas et al., 2020). In the current management strategies for cardiovascular diseases, active intervention targeting common cardiovascular risk factors (Roth et al., 2020) such as hypertension, diabetes, and hypercholesterolemia has been proven to effectively reduce the risk of cardiovascular disease incidence. Moreover, pharmacological treatments with beta-blockers and renin-angiotensin-aldosterone system (RAAS) antagonists have been demonstrated to effectively delay or ameliorate cardiac remodeling and the deterioration of cardiac function (Doughty et al., 1997; Konstam et al., 2011; von Lueder et al., 2015) Undeniably, these interventions and the use of specific medications have inhibited the deterioration of cardiac structure and function on multiple levels. However, an unavoidable issue is the existence of residual cardiovascular risk, which transcends the issues directly addressed by current standard management strategies (Libby, 2005; Fu et al., 2022) and may serve as a significant explanatory factor for the high incidence of cardiovascular events under standard intervention strategies. Therefore, following the control of classical cardiovascular risk factors, intervention targeting residual cardiovascular risk could potentially be a pathway to further suppress the deterioration of cardiac structure and function.

Within the multifactorial considerations of cardiovascular disease, the gut microbiota (GM) has garnered substantial attention from the scientific community due to its extensive impact on health (Zmora et al., 2019; Fan and Pedersen, 2021; Chakaroun et al., 2023). An increasing body of evidence has revealed associations between the GM and cardiovascular diseases (Witkowski et al., 2020). At the disease aspect, the GM has been observed to be associated with diseases such as coronary artery disease (Emoto et al., 2016; Cui et al., 2017), heart failure (Kummen et al., 2018), and diabetic cardiomyopathy (Yuan et al., 2022), exhibiting significant dysbiosis in affected populations. In terms of therapeutic strategies, research targeting the regulation of GM diversity has supported the possibility of GM as a potential therapeutic target for cardiovascular diseases. For instance, a clinical study by Moludi Jalal et al. (Moludi et al., 2021) demonstrated that probiotic supplementation in patients with myocardial infarction could alleviate post-infarction cardiac remodeling and reduce the levels of biomarkers associated with myocardial remodeling. Likewise, research by Costanza et al. (2015) found that supplementation with *Saccharomyces boulardii* could improve left atrial diameter and left ventricular ejection fraction in patients with heart failure. Moreover, a multitude of experimental studies have confirmed that modulating the GM can facilitate improvements in cardiac remodeling and function (Lam et al., 2012; Lin et al., 2013; Gan et al., 2014). These findings reinforce the credibility of the “gut-heart axis” hypothesis, suggesting the potential of considering the GM as a residual risk factor for cardiac structure and function. Nevertheless, it is imperative to acknowledge that most current studies only indicate associative results or have limitations in ruling out confounding factors, leaving the causal relationship between the GM and cardiac structure and function undetermined. If a causal link between the GM and cardiac structure and function were to be established, it would provide critical support for recognizing the GM as a potential residual cardiovascular risk factor and could offer new therapeutic targets for improving cardiac structure and function beyond conventional treatments.

Mendelian randomization (MR), as an emerging epidemiological tool, is increasingly being employed to investigate the causal relationships between exposure factors and specific health outcomes. The primary advantage of this method lies in its ability to effectively control for confounding factors and biases due to reverse causation (Richmond and Davey, 2022; Sanderson et al., 2022). With the growing accessibility of large-scale genome-wide association study (GWAS) data on GM, it is now feasible to preliminarily explore the causal links between the GM and cardiac structure and function using MR studies prior to conducting randomized controlled trials (RCTs). This approach can provide valuable insights for the design of future RCTs (Ference et al., 2021).

In this study, we employed publicly available GWAS summary-level data to conduct a two-sample MR analysis with the aim of exploring the causal relationship between the GM and cardiac structure and function. Furthermore, for those GM and cardiac traits that exhibited a causal link, we performed bioinformatics annotation on the GWAS data to investigate the potential biological mechanisms by which the GM may influence cardiac structure and function.

## 2 Materials and methods

### 2.1 Study design

**Figure 1:**
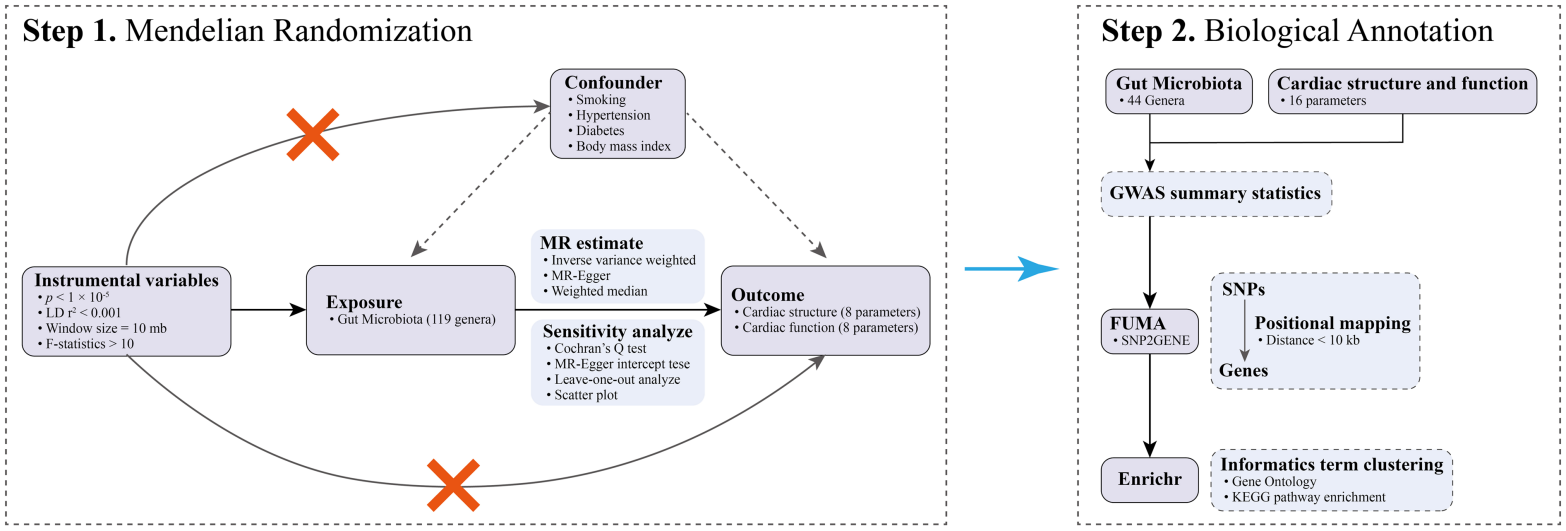
The flowchart of the current study.

This study comprises MR analysis and biological annotation, as depicted in Figure 1. In the MR analysis, we considered 119 genera of GM as the exposures, while 16 parameters pertaining to cardiac structure and function were identified as the outcomes. Importantly, the MR analysis adhered to the guidelines delineated in the STROBE-MR statement (Skrivankova et al., 2021), as detailed in Supplementary Table S1. As this research utilized publicly available, aggregated data from GWAS, it was exempt from the requirement of ethical approval.

### 2.2 Data sources

The summary-level GWAS data for GM originated from a large-scale association analysis released by the MiBioGen consortium (Kurilshikov et al., 2021). The study encompassed 18,340 participants across 24 independent cohorts, the majority of whom were of European ancestry (*n* = 13,266) (Table 1). The cohorts involved in the study employed 16S rRNA gene sequencing for microbial identification, successfully identifying 131 microbial genera with an average abundance greater than 1%, including 12 unidentified genera that have not yet been classified. In this MR study, we included GWAS data for 119 genera-level classifications of GM for comprehensive analysis. Detailed information of the 119 GM genera shown in Supplementary Table S2.

**Table 1:** Detailed information of GWAS data.

Trait	Consortium	Ancestry	Participants	Phenotypes
GM	MiBioGen	Mixed	18,340 (13,266 Europeans)	119 genera
Right heart	UK Biobank	European	41,135 Europeans	7 parameters
Left atrium	UK Biobank	European	35,658 Europeans	5 parameters
Left ventricle	UK Biobank	European	36,041 Europeans	4 parameters

Summary-level GWAS data on cardiac structure and function were derived from three extensive cardiac magnetic resonance imaging studies of UK Biobank participants (Table 1). The GWAS data for the right heart were extracted from the research conducted by Pirruccello et al. (2022), which included up to 41,135 participants of European ancestry. These participants contributed data to at least one GWAS project and were not diagnosed with heart failure, pulmonary hypertension, atrial fibrillation, or coronary artery disease at the time of their enrollment. In this MR study, we selected seven parameters measured in the aforementioned research as outcome variables for assessing right cardiac structure and function, including body surface area (BSA)-indexed right atrial maximum area (iRAMAX), BSA-indexed right atrial minimum area (iRAMIN), right atrial fractional area change (RAFAC), BSA-indexed right ventricular end diastolic volume (iRVEDV), BSA-indexed right ventricular end systolic volume (iRVESV), right ventricular ejection fraction (RVEF), and BSA-indexed right ventricular stroke volume (iRVSV). The GWAS data for the structure and function of the left atrium were obtained from the study conducted by Gustav Ahlberg et al. (Ahlberg et al., 2021), which encompassed up to 35,658 individuals of European descent. This study excluded participants with a history of myocardial infarction, heart failure, cardiomyopathy, and those with a body mass index below 16 or above 40 kg/m^2^. In our MR investigation, we selected five parameters of the left atrium as endpoints to characterize its structure and function, including BSA-indexed left atrial maximum volume (iLAMAX), BSA-indexed left atrial minimum volume (iLAMIN), left atrial active emptying fraction (LAAEF), left atrial passive emptying fraction (LAPEF), and left atrial total emptying fraction (LATEF). Regarding the structure and function of the left ventricle, we chose four parameters from the research by Pirruccello et al. (2020) as endpoints representing the left ventricular structure and function in this MR study, including BSA-indexed left ventricular end-diastolic volume (iLVEDV), BSA-indexed left ventricular end-systolic volume (iLVESV), BSA-indexed left ventricular stroke volume (iLVSV), and left ventricular ejection fraction (LVEF). This research included 36,041 European ancestry subjects who were not diagnosed with congestive heart failure, coronary artery disease, dilated cardiomyopathy, or hypertrophic cardiomyopathy at the time of enrollment.

### 2.3 Two sample MR analysis

#### 2.3.1 Instrumental variables (IVs) selection

In the selection of IVs, we established a set of criteria to ensure the validity and sufficient analytical strength of IVs: (1) The IVs must be significantly associated with the GM; therefore, we used a genome-wide significance threshold of *p* < 1 × 10^−5^ to filter potential instruments for more comprehensive results. (2) To ensure no linkage disequilibrium between instruments, we employed an *r*^2^ < 0.001 and a clumping window of 10 MB based on the 1,000 Genomes reference panel. (3) To account for the potential influence of pleiotropy, we used the PhenoScanner V2 database (Kamat et al., 2019) to check and exclude single nucleotide polymorphisms (SNPs) significantly associated with smoking, blood pressure, diabetes, and body mass index. (4) When matching exposure to outcome data, we excluded SNPs that could not be matched in the outcome dataset, palindromic SNPs, and those significantly associated with the outcome at a genome-wide level (*p* < 1 × 10^−5^) to ensure compliance with the fundamental assumptions of MR. (5) To avoid weak instrument bias, we calculated the *F*-statistic for each instrumental variable using the formula (Gill et al., 2019): *F* = *R*^2^ × (N-2)/(1-R^2^), and eliminated any instruments with an *F*-statistic less than 10 (Pierce et al., 2011).

#### 2.3.2 MR estimates

In the MR analysis, the inverse variance weighted (IVW) method was employed as the primary approach for assessing the causal effects of GM on cardiac structure and function (Burgess et al., 2013). This method presupposes the validity of all IVs and synthesizes the Wald ratio estimates from different SNPs to determine the aggregate effect of GM. In the presence of heterogeneity, a random-effects model is utilized; otherwise, a fixed-effects model is chosen. Additionally, the weighted median (WM) and MR-Egger methods were employed as supplementary statistical models to provide more robust MR estimates, albeit at the expense of reduced statistical power. The WM approach can yield a consistent estimate of causality on the condition that over 50% of the IVs are valid (Bowden et al., 2016). Meanwhile, the MR-Egger method offers a means to detect and correct for potential horizontal pleiotropy, thereby providing consistent estimates, though these may be associated with the lowest statistical power (Bowden et al., 2015).

#### 2.3.3 Sensitivity analyses

We further conducted sensitivity analyses to assess the robustness of our MR results. These analyses included Cochran’s Q test to detect potential heterogeneity among IVs, the MR-Egger intercept test for pleiotropy assessment, and leave-one-out (LOO) analysis to evaluate the influence of individual SNPs. Cochran’s Q test was utilized to detect heterogeneity, with a *p*-value of less than 0.05 indicating its presence, which also served as a criterion for selection of IVW model, as previously stated (Bowden et al., 2015). The MR-Egger intercept test was performed to detect any pleiotropy; here, a *p*-value less than 0.05 was indicative of its presence (Bowden et al., 2019). Additionally, LOO analysis was undertaken to determine whether the MR estimates were disproportionately influenced by any single SNPs.

#### 2.3.4 Statistical analysis

MR estimates and sensitivity analyses were performed using R packages “TwoSampleMR (version 0.5.6)” (Hemani et al., 2018) in R (version 4.3.2). *p*-values less than 0.05 in MR assessment are considered to indicate potential causal associations.

### 2.4 Biological annotation

To investigate the potential mechanisms by which the GM influences cardiac structure and function, we conducted post-GWAS analyses on summary-level GWAS data for GM and cardiac phenotypes that exhibited causal associations as revealed by MR analysis. Initially, SNP-to-gene mapping for the GWAS data of GM and cardiac phenotypes was performed using the FUMA platform (Watanabe et al., 2017, 2019) to obtain annotated gene sets for each. Within FUMA, the SNP2GENE function executed positional mapping based on ANNOVAR annotations (Wang et al., 2010), with a maximum distance of 10 kb set between SNPs and genes. Subsequently, bioinformatics enrichment clustering (Hendrickx et al., 2020) of the mapped genes related to the GM and cardiac phenotypes was carried out using Enrichr (https://maayanlab.cloud/Enrichr/) (Kuleshov et al., 2016), including Gene Ontology (GO) biological processes enrichment analysis and Kyoto Encyclopedia of Genes and Genomes (KEGG) pathway enrichment analysis, to facilitate subsequent biological function analysis.

## 3 Results

### 3.1 IVs selection for GM

Following multi-stage quality control procedures, we identified 1,246 SNPs associated with the GM to serve as IVs. The F-statistics for these instruments ranged from 14.58 to 36.57, all above the threshold of 10, which robustly suggests the absence of weak instruments. Detailed information regarding the IVs can be found in Supplementary Table S3.

### 3.2 MR estimates

In this study, we conducted a large-scale MR analysis to explore the potential causal relationships between 119 genera of the GM and 16 phenotypes related to cardiac structure and function. By utilizing IVW as the primary MR analysis method, unifying the direction of three MR models, and removing results with pleiotropy, we identified 93 causal associations between 44 GM genera and 16 cardiac structural and functional phenotypes. This suggests a broad causal impact of GM on cardiac structure and function. The complete MR estimates and sensitivity analysis results are shown in Supplementary Table S4.

### 3.3 The causal association between GM and cardiac structure

**Figure 2:**
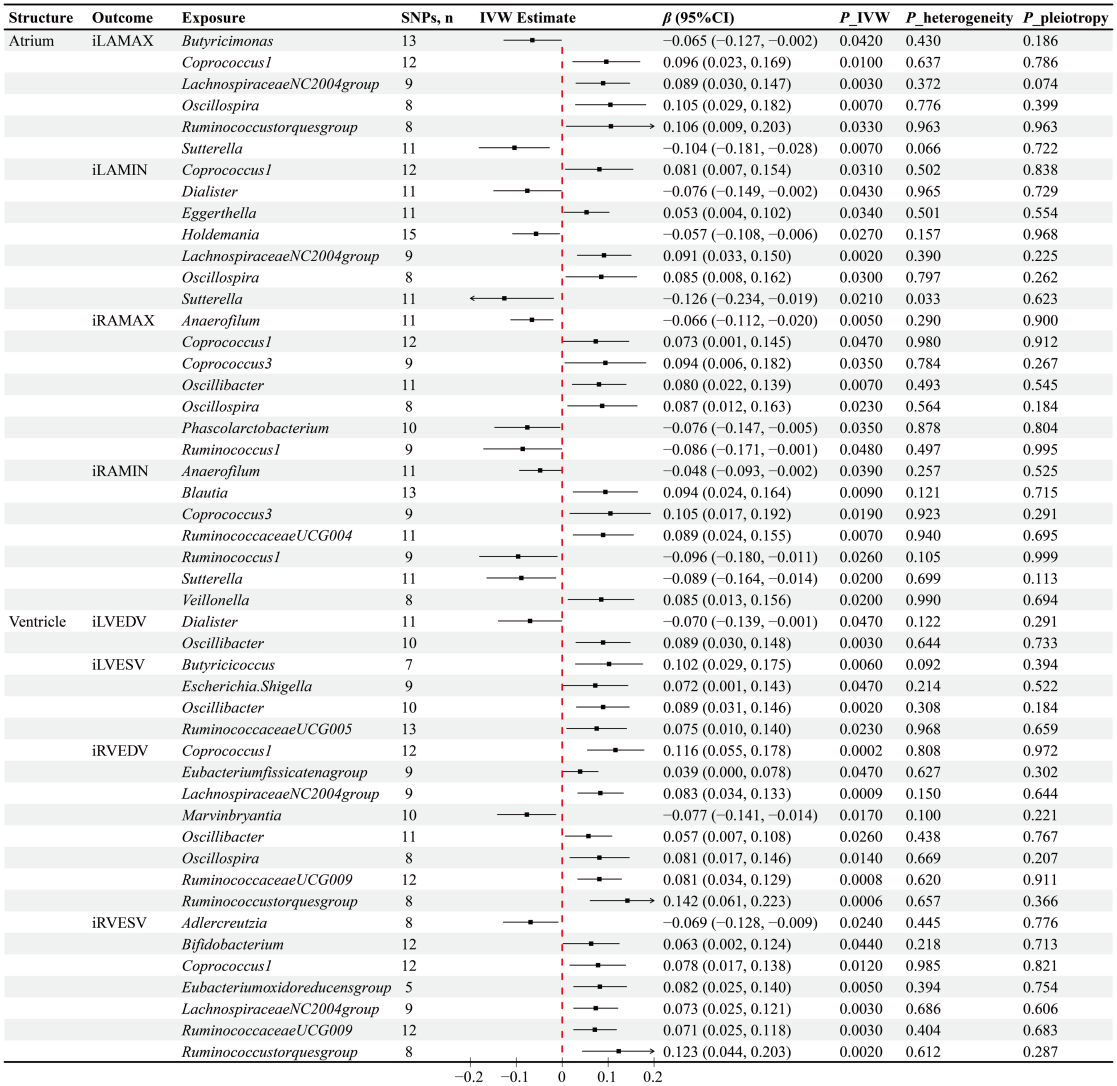
Results of the MR analysis between GM and cardiac structure. *IVW* inverse variance weighted, *β* beta coefficient, CI confidence interval.

In terms of atrial structure, a MR analysis utilizing the IVW method as the primary analytical tool indicated significant causal associations between the abundance of 18 GM genera and atrial structural features. Specifically, for the iLAMAX, increased abundance of *Sutterella* (*β* = −0.104, *p* = 0.007) and *Butyricimonas* (*β* = −0.065, *p* = 0.042) was causally associated with a reduction in iLAMAX; conversely, an increase in the abundance of *Coprococcus1* (*β* = 0.096, *p* = 0.01), *LachnospiraceaeNC2004group* (*β* = 0.089, *p* = 0.033), *Oscillospira* (*β* = 0.105, *p* = 0.007), and *Ruminococcustorquesgroup* (*β* = 0.106, *p* = 0.007) was causally associated with an increase in iLAMAX. Additionally, an increased abundance of *Dialister* (*β* = −0.076, *p* = 0.043), *Holdemania* (*β* = −0.057, *p* = 0.027), and *Sutterella* (*β* = −0.126, *p* = 0.021) was causally linked to a decrease in the iLAMIN, while an increased abundance of *Coprococcus1* (*β* = 0.081, *p* = 0.031), *Eggerthella* (*β* = 0.053, *p* = 0.034)*, LachnospiraceaeNC2004group* (*β* = 0.091, *p* = 0.002), and *Oscillospira* (*β* = 0.085, *p* = 0.03) was related to an increase in iLAMIN. For the right atrial structure, an increased abundance of *Anaerofilum* (*β* = −0.066, *p* = 0.005), *Phascolarctobacterium* (*β* = −0.076, *p* = 0.035), and *Ruminococcus1* (*β* = −0.086, *p* = 0.048) was causally associated with a reduction in the iRAMAX, whereas an increased abundance of *Anaerofilum* (*β* = −0.048, *p* = 0.039), *Ruminococcus1* (*β* = −0.096, *p* = 0.026), and *Sutterella* (*β* = −0.089, *p* = 0.02) was causally linked to a decrease in the iRAMIN. Furthermore, higher abundances of *Coprococcus1* (*β* = 0.073, *p* = 0.047), *Coprococcus3* (*β* = 0.094, *p* = 0.035), *Oscillibacter* (*β* = 0.08, *p* = 0.007), and *Oscillospira* (*β* = 0.087, *p* = 0.023) were causally associated with an increase in iRAMAX, and higher abundances of *Blautia* (*β* = 0.094, *p* = 0.009), *Coprococcus3* (*β* = 0.105, *p* = 0.019), *RuminococcaceaeUCG004* (*β* = 0.089, *p* = 0.007), and *Veillonella* (*β* = 0.085, *p* = 0.02) were causally related to an increase in iRAMIN (Figure 2).

Through the IVW method, a total of 15 GM genera were found to have causal associations with ventricular structure. Specifically for the left ventricular structure, higher levels of *Dialister* were observed to negatively impact iLVEDV (*β* = −0.07, *p* = 0.047), while *Oscillibacter* exhibited simultaneous positive associations with both iLVEDV (*β* = 0.089, *p* = 0.003) and iLVESV (*β* = 0.089, *p* = 0.002). Moreover, increased abundance of Butyricicoccus (*β* = 0.102, *p* = 0.006), *Escherichia.Shigella* (*β* = 0.072, *p* = 0.047), and *RuminococcaceaeUCG005* (*β* = 0.075, *p* = 0.023) was causally associated with higher iLVESV. Regarding the right ventricular structure, *Marvinbryantia* was found to negatively affect iRVEDV (*β* = −0.077, *p* = 0.017), along with *Adlercreutzia* negatively impacting iRVESV (*β* = −0.069, *p* = 0.024). Furthermore, higher abundances of *Coprococcus1* (*β* = 0.116, *p* = 0.0002), Eubacteriumfissicatenagroup (*β* = 0.039, *p* = 0.047), LachnospiraceaeNC2004group (*β* = 0.083, *p* = 0.0009), Oscillibacter (*β* = 0.057, *p* = 0.026), oscillospira (*β* = 0.081, *p* = 0.014), RuminococcaceaeUCG009 (*β* = 0.081, *p* = 0.0008), and Ruminococcustorquesgroup (*β* = 0.142, *p* = 0.0006) were found to positively impact iRVEDV. Additionally, Bifidobacterium (*β* = 0.063, *p* = 0.044), Coprococcus1 (*β* = 0.078, *p* = 0.012), Eubacteriumoxidoreducensgroup (*β* = 0.082, *p* = 0.005), LachnospiraceaeNC2004group (*β* = 0.073, *p* = 0.003), RuminococcaceaeUCG009 (*β* = 0.071, *p* = 0.003), and Ruminococcustorquesgroup (*β* = 0.123, *p* = 0.002) were identified to have causal relationships with increased iRVESV (Figure 2).

### 3.4 The causal association between GM and cardiac function

**Figure 3:**
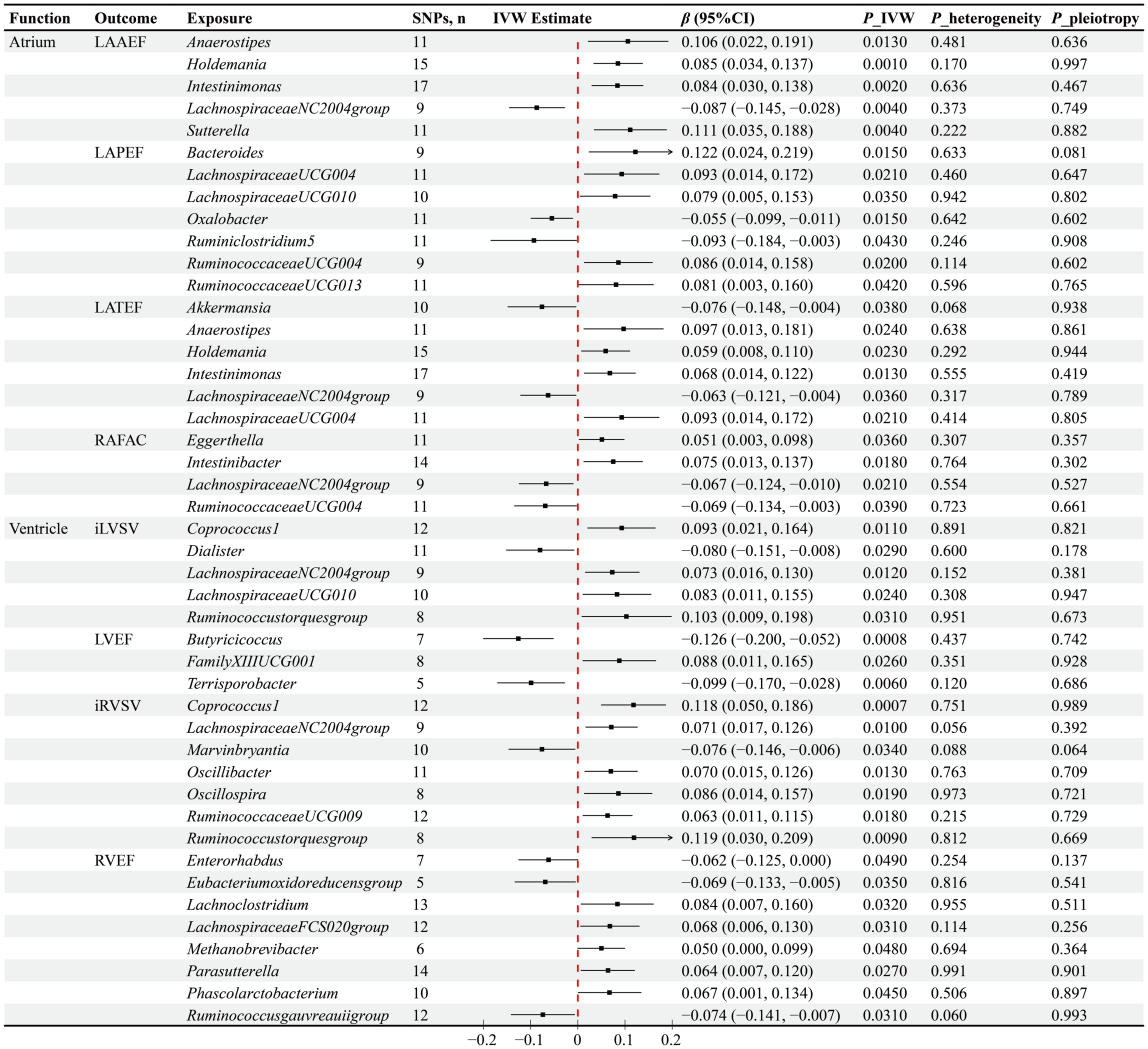
Results of the MR analysis between GM and cardiac function. *IVW* inverse variance weighted, *β* beta coefficient, CI confidence interval.

The IVW method has identified causal associations between the abundance of 15 GM genera and atrial function. Specifically, the increased abundance of *Anaerostipes* (*β* = 0.106, *p* = 0.013)*, Holdemania* (*β* = 0.085, *p* = 0.001), *Intestinimonas* (*β* = 0.084, *p* = 0.002), and *Sutterella* (*β* = 0.111, *p* = 0.004) genera has been observed to causally increase LAAEF, while *LachnospiraceaeNC2004group* (*β* = −0.087, *p* = 0.004) has the opposite effect. Furthermore, higher abundances of *Bacteroides* (*β* = 0.122, *p* = 0.015), *LachnospiraceaeUCG004* (*β* = 0.093, *p* = 0.021), *LachnospiraceaeUCG010* (*β* = 0.079, *p* = 0.035), *RuminococcaceaeUCG004* (*β* = 0.086, *p* = 0.02), and *RuminococcaceaeUCG013* (*β* = 0.081, *p* = 0.042) have been found to causally increase LAPEF, whereas *Oxalobacter* (*β* = −0.055, *p* = 0.015) and *Ruminiclostridium5* (*β* = −0.093, *p* = 0.043) genera have the reverse influence. In terms of LATEF, a positive causal influence of higher abundances of *Anaerostipes* (*β* = 0.097, *p* = 0.024)*, Holdemania* (*β* = 0.059, *p* = 0.023), *Intestinimonas* (*β* = 0.068, *p* = 0.013), and *LachnospiraceaeUCG004* (*β* = 0.093, *p* = 0.021) genera has been observed, along with a negative causal influence of *Akkermansia* (*β* = −0.076, *p* = 0.038) and *LachnospiraceaeNC2004group* (*β* = −0.063, *p* = 0.036) genera. Regarding RAFAC, the IVW method has determined the positive causal influence of *Eggerthella* (*β* = 0.051, *p* = 0.036) and *Intestinibacter* (*β* = 0.075, *p* = 0.018) genera, as well as the negative causal influence of *LachnospiraceaeNC2004group* (*β* = −0.067, *p* = 0.021) and *RuminococcaceaeUCG004* (*β* = −0.069, *p* = 0.039) genera (Figure 3).

The IVW method has determined causal associations between the abundance of 20 GM genera and ventricular function. Specifically, negative causal associations have been observed between *Dialister* (*β* = −0.08, *p* = 0.029) and iLVSV, *Butyricicoccus* (*β* = −0.126, *p* = 0.0008) and *Terrisporobacter* (*β* = −0.099, *p* = 0.006) with LVEF, *Marvinbryantia* (*β* = −0.076, *p* = 0.034) and iRVSV, as well as *Enterorhabdus* (*β* = −0.062, *p* = 0.049), *Eubacteriumoxidoreducensgroup* (*β* = −0.069, *p* = 0.035), *Ruminococcusgauvreauiigroup* (*β* = −0.074, *p* = 0.031), and RVEF. Furthermore, increased abundances of *Coprococcus1* (*β* = 0.093, *p* = 0.011), *LachnospiraceaeNC2004group* (*β* = 0.073, *p* = 0.012), *LachnospiraceaeUCG010* (*β* = 0.083, *p* = 0.024), and *Ruminococcustorquesgroup* (*β* = 0.103, *p* = 0.031) genera have been found to causally increase iLVSV, while increased abundances of *Coprococcus1* (*β* = 0.118, *p* = 0.0007), *LachnospiraceaeNC2004group* (*β* = 0.071, *p* = 0.01), *Oscillibacter* (*β* = 0.07, *p* = 0.013), *Oscillospira* (*β* = 0.086, *p* = 0.019)*, RuminococcaceaeUCG009* (*β* = 0.063, *p* = 0.018), and *Ruminococcustorquesgroup* (*β* = 0.119, *p* = 0.009) genera have been found to causally increase iRVSV. Additionally, a causal association has been observed between higher abundance of *FamilyXIIIUCG001* (*β* = 0.088, *p* = 0.026) genus and higher LVEF, as well as between higher abundances of *Lachnoclostridium* (*β* = 0.084, *p* = 0.032), *LachnospiraceaeFCS020group* (*β* = 0.068, *p* = 0.031), *Methanobrevibacter* (*β* = 0.05, *p* = 0.048)*, Parasutterella* (*β* = 0.064, *p* = 0.027), and *Phascolarctobacterium* (*β* = 0.067, *p* = 0.045) genera and higher RVEF (Figure 3).

### 3.5 Sensitivity analysis

To evaluate the robustness of our causal inference results, we conducted sensitivity analyses (Figures 2, 3). The Cochran’s Q test revealed significant heterogeneity in the causal assessment between the *Sutterella* genus and iLAMIN (*p* = 0.033), hence a random effects model of IVW was employed for the analysis. Moreover, no evidence of horizontal pleiotropy was detected in the assessment of 93 causal associations between GM and cardiac phenotypes, as indicated by Egger regression analysis, with all Egger’s intercept *p*-values being greater than 0.05. LOO analysis suggested that our MR results were not unduly influenced by any single SNPs. Scatter plots demonstrated consistent directions of causal estimates for the 93 associations between GM and cardiac phenotypes using the IVW method as well as two supplementary methods—MR Egger and WM, further reinforcing our confidence in the causal assessments.

### 3.6 Biological annotation

**Figure 4:**
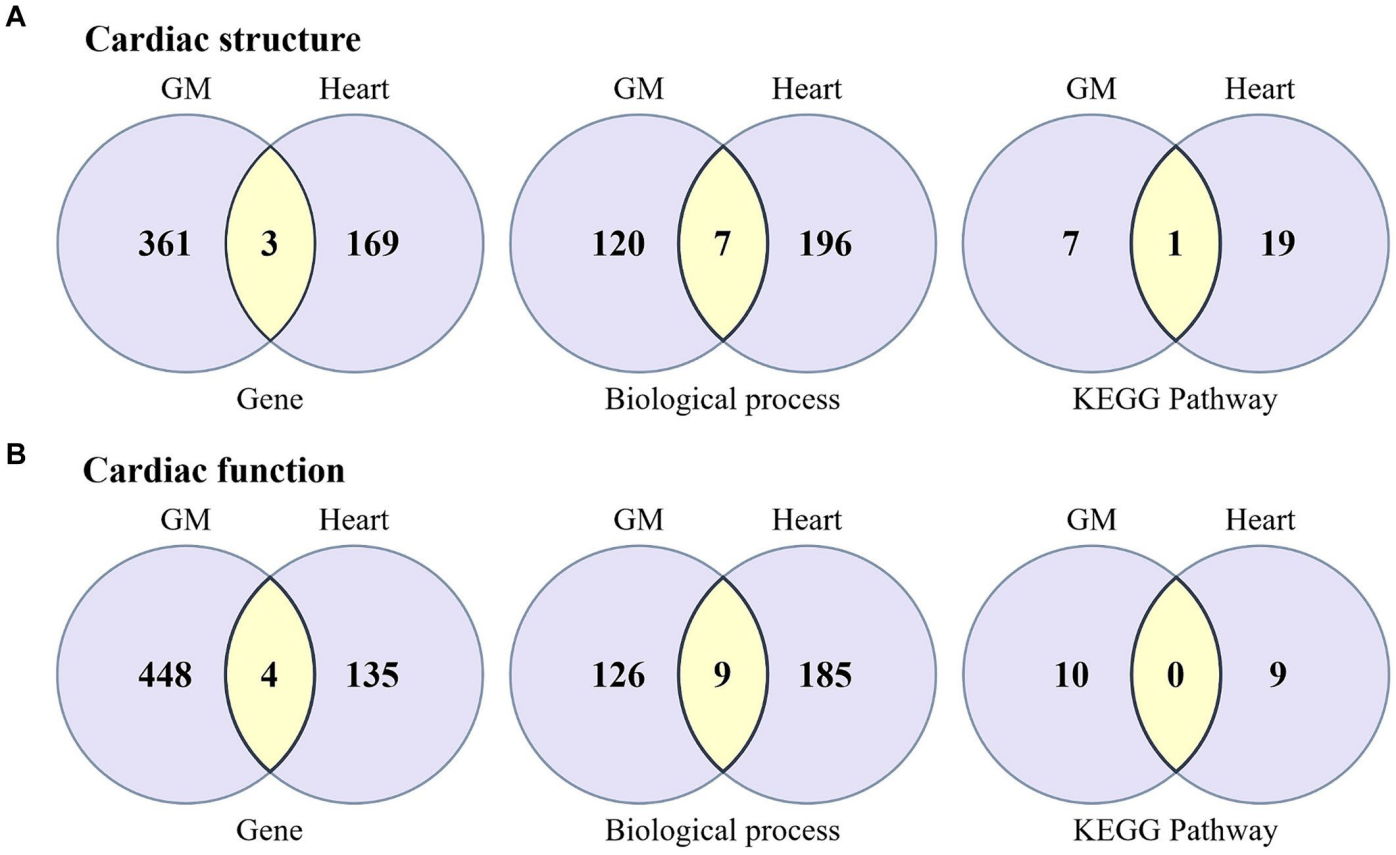
Venn diagram illustrating the biological annotation results for the associations between GM and cardiac structure and function, encompassing the outcomes of genes, biological processes, and pathway analyses. **(A)** The biological association between GM and cardiac structure. **(B)** The biological association between GM and cardiac function. Light purple represents the unique genes, biological processes, and pathways specific to GM and the heart, while yellow indicates the shared components between GM and the heart.

In the current MR study, 93 causal associations were identified between GM from 44 genera and 16 cardiac phenotype parameters. Specifically, causal links were found between 26 genera of GM and cardiac structural parameters, and between 33 genera and cardiac functional parameters. Utilizing FUMA to gene-map the summary-level GWAS data for these microbiota genera and cardiac phenotype parameters, we discovered that in studies concerning cardiac structural parameters, the 26 microbiota genera were mapped to 364 genes, and the 8 cardiac structural parameters were mapped to 172 genes (Supplementary Table S5), sharing three common genes among them: *PLEKHA3*, *TTN*, and *NPR3*. In the analysis of cardiac functional parameters, the 33 microbiota genera and 8 cardiac functional parameters were, respectively, mapped to 452 and 139 genes, including four shared genes: *PLEKHA3*, *TTN*, *PDE11A*, and *SLC28A1*, underscoring potential genetic intersections relevant to both GM composition and cardiac function (Figure 4).

To further understand the potential mechanisms by which GM influence cardiac structure and function, we conducted GO biological process enrichment analysis and KEGG pathway enrichment analysis on genes associated with both GM and cardiac phenotypes. The results indicated that for cardiac structural parameters, the genes localized to GM and cardiac structural parameters were significantly enriched in 127 and 203 biological processes, respectively, sharing seven processes including *Cardiac Muscle Cell Development* (GO:0055013), *Negative Regulation Of Calcium Ion Transmembrane Transport* (GO:1903170), *Regulation Of Cell Adhesion* (GO:0030155), *Regulation Of Signal Transduction* (GO:0009966), and *Regulation Of Calcium Ion Transmembrane Transporter Activity* (GO:1901019). In addition, these genes were significantly enriched in 8 and 20 KEGG pathways, respectively, with the *MAPK signaling pathway* being shared between them. On the other hand, for cardiac functional parameters, the genes related to GM and cardiac functional parameters were significantly enriched in 135 and 194 biological processes, respectively, sharing nine processes such as *Cardiac Muscle Cell Development* (GO:0055013), *Cardiac Cell Development* (GO:0055006), *Cardiac Myofibril Assembly* (GO:0055003), *Regulation Of Heart Contraction* (GO:0008016), *Muscle Filament Sliding* (GO:0030049), *Actin-Myosin Filament Sliding* (GO:0033275), among others. However, these genes were enriched in 10 and 9 KEGG pathways, respectively, without any shared pathways between them (Figure 4; Supplementary Table S6).

## 4 Discussion

The discovery and research of the “gut-heart” axis have broadened our thinking from a non-cardiac perspective on the influence of variable factors on cardiac structure and function. To the best of our knowledge, the current study represents the first attempt to utilize MR analysis to extensively explore causal relationships between GM and cardiac structure and function. It found that the abundance of certain genetically predicted GM has a causal effect on cardiac structure and function, indicating that GM might serve as a potential residual risk factor affecting cardiac structure and function. Further biological annotation and analysis hinted at the possible mechanisms through which GM affect cardiac structure and function.

Numerous clinical studies have revealed significant differences in the composition of the GM between patients with cardiovascular disease and healthy individuals, suggesting an association between GM and cardiovascular disease phenotypes (Karlsson et al., 2012; Pasini et al., 2016; Ziganshina et al., 2016; Jie et al., 2017; Li et al., 2017). However, these observational findings are limited in their ability to infer causality between the GM and cardiac structure and function. RCTs, as powerful tools for causal inference, have shown results that are both encouraging and uncertain. Interventions such as dietary modifications, probiotic supplementation, antibiotic treatments, and fecal microbiota transplantation are commonly used in the design of RCTs to alter the abundance of the GM (Witkowski et al., 2020). Research by Moludi et al. (2021) and Costanza et al. (2015) indicates that prebiotic supplementation may have beneficial effects on the cardiac structure and function of patients with cardiovascular disease. Moreover, prebiotic supplementation has been found to improve cardiovascular metabolic health (Ejtahed et al., 2011; DiRienzo, 2014; Kullisaar et al., 2016; Zhao et al., 2021), which may indirectly contribute to the improvement of cardiac structure and function. Experimental research has bolstered the confidence in causal inference between the GM and cardiac health, as direct or indirect interventions targeting the GM have shown impact on cardiac structure and function. The study by Marques et al. (2017) demonstrates that a high-fiber diet or acetate supplementation can reduce blood pressure, alleviate cardiac fibrosis, and attenuate left ventricular hypertrophy in mice treated with excess mineralocorticoids, potentially through reducing the proportion of *Firmicutes* and *Bacteroidetes* bacteria and ameliorating gut dysbiosis. The research by Battson et al. (2019) found that transplanting cecal microbiota from control mice into obese leptin-deficient mice altered the GM composition and enhanced myocardial ischemic tolerance, decreased infarct size, and inhibited left ventricular hypertrophy. Lam et al.’s (2012) study also suggests that manipulating the GM with probiotics may have beneficial effects on cardiac remodeling and mechanical function in rats post-myocardial infarction. Similar findings were reported by Gan et al. (2014), where probiotic use improved post-infarct left ventricular remodeling and dysfunction in rats, and these beneficial effects persisted even after discontinuation of probiotics, possibly related to improved myocardial metabolic status.

Among the gut microbiota genera identified in our study as causally linked to cardiac structure, an increase in abundance of *Sutterella* has been reported to have beneficial effects on blood pressure (de la Visitacion et al., 2021; Thomaz et al., 2021). Conversely, a decrease in its abundance may exacerbate arterial hardening (Hu et al., 2021). *Dialister*, on the other hand, has been found to negatively correlate with blood pressure (Shah et al., 2020) and glycated hemoglobin levels in prediabetic patients (Zhang et al., 2017). Clinical observations by Zheng Wang et al. indicate a close relationship between reduced *Adlercreutzia* abundance and carotid artery plaque formation, as well as a negative correlation with glycated hemoglobin (Wang et al., 2022); animal experiments suggest that increasing *Adlercreutzia* abundance may be a critical pathway for improving atherosclerosis (Hao et al., 2023). Additionally, in animal studies by Yang et al. (2021), Akebia saponin D improves metabolic syndrome by increasing *Butyricimonas* abundance. Research by Sareema Adnan et al. demonstrates a negative correlation between the relative abundance of acetate-producing genus *Holdemania* and systolic pressure (Adnan et al., 2017). Clinical studies indicate that *Marvinbryantia* abundance is higher in normotensive individuals compared to hypertensive patients (Dan et al., 2019), and is associated with reduced insulin resistance and risk of type 2 diabetes (Chen et al., 2021). Case–control studies by Li et al. (2022) show lower *Phascolarctobacterium* abundance in type 2 diabetes patients compared to healthy individuals, while research by Negar Naderpoor et al. reveals a significant positive correlation between *Phascolarctobacterium* and insulin sensitivity in obese individuals (Naderpoor et al., 2019). These GM genera, implicated in our MR study, were confirmed to have negative causal associations with cardiac structure. Conversely, clinical studies highlight increased abundance of *Escherichia.Shigella* (Wang et al., 2021), *Eggerthella* (Yan et al., 2017), *Coprococcus3* (Louca et al., 2021), and *Eubacteriumfissicatenagroup* (Nakai et al., 2021) in hypertensive patients. Thomaz et al.’s (2021) animal experiments show a positive correlation between *Oscillospira* abundance and systolic blood pressure. Moreover, *Escherichia.Shigella* is enriched in obese individuals (Hu et al., 2022), subclinical carotid atherosclerosis (Baragetti et al., 2021), and coronary artery disease patients (Zhu et al., 2018). *Veillonella* has been identified as a common oral-gut translocating microbe, stable in hypertensive participants (Chen et al., 2023), and detectable in atherosclerotic plaques (Koren et al., 2011). These GM genera, identified in our MR study, exhibit positive causal effects on cardiac structure. Overall, the GM genera identified in this MR study with causal associations to cardiac structure may have close links to subclinical cardiovascular disease risk or common cardiovascular risk factors, potentially partially explaining their causal effects on cardiac structure.

On the other hand, similar to the GM genera causally linked to cardiac structure mentioned above, the GM genera identified in this study as causally associated with cardiac function predominantly exhibit associations with subclinical cardiovascular diseases or traditional cardiovascular risk factors. For example, genera such as *Ruminiclostridium5*, *Oxalobacter*, *Terrisporobacter*, and *Enterorhabdus* have been reported to be closely associated with the phenotype of obesity. María Bailén et al. clinically observed a positive correlation between *Ruminiclostridium5* abundance and female adiposity (Bailen et al., 2020). Moreover, Hongchao Wang et al. found a significant positive correlation between *Oxalobacter* and body mass index (Zhang et al., 2020). Experimental studies have demonstrated that *Terrisporobacter* promotes obesity, with its abundance significantly increased by high-fat diets (Zheng et al., 2022). *Enterorhabdus* is enriched in mice fed a high-fat diet and positively correlates with their fasting blood glucose levels (Wang et al., 2022), as well as with liver total cholesterol and plasma trimethylamine N-oxide concentrations, while negatively correlating with plasma high-density lipoprotein cholesterol levels (Zheng et al., 2022). These GM genera identified in our MR study as causally associated with declining cardiac function likely contribute to the obesity phenotype, thereby potentially contributing to decreased heart function. Furthermore, this study also identified certain GM genera causally linked to improved cardiac function. Among these genera, *Methanobrevibacter* has been reported to decrease in abundance in overweight populations (Schwiertz et al., 2010; Million et al., 2012). Experimental research by Xiaoyun Fan suggests that the abundance of *LachnospiraceaeFCS020group* negatively correlates with serum total cholesterol and triglyceride levels in hyperlipidemic rats (Fan et al., 2023). Additionally, *Intestinimonas* (Guo et al., 2021), *LachnospiraceaeUCG004* (Lan et al., 2022), and *Anaerostipes* (Song et al., 2023) have been found to decrease in abundance in hypertensive populations; *Intestinibacter* (Chen et al., 2021) and *Anaerostipes* (Bui et al., 2021) genera are negatively associated with diabetes risk, while *LachnospiraceaeUCG004* (Toya et al., 2020) and *Bacteroides* (Emoto et al., 2016) are less abundant in coronary artery disease patients. These studies suggest that these GM genera may play a protective role in cardiovascular health, which could be a contributing factor to their positive causal effects on cardiac function.

In addition to the findings consistent with the conclusions drawn from our MR study, divergent results have also been reported in some other research. For instance, *Oscillospira* is purported to have potential benefits in weight loss, lipid reduction, slimming, and alleviating metabolic syndrome, positioning it as a promising candidate for the next generation of probiotics (Yang et al., 2021). However, as previously mentioned, its abundance shows a positive correlation with systolic blood pressure. Similarly, *Terrisporobacter*, identified in our MR study as negatively causally linked to heart function, is recognized elsewhere as a promoter of obesity-related GM. Conversely, studies have indicated higher abundance of *Terrisporobacter* in control groups compared to diabetic patients (Radwan et al., 2020). These disparate findings underscore inconsistencies in assessments of the cardiovascular health impacts of these GM genera, likely attributable to study heterogeneity and the confounding effects inherent in observational research. Although clinical and experimental studies have provided valuable insights into the causal relationship between the GM and cardiac structure and function, it is crucial not to overlook the confounding factors inherent in clinical research, as well as the issues of species differences and model validity in experimental studies that may affect causal inference. Given these limitations, the precise causal link between the GM and cardiac structure and function remains unclear. In this study, we employed a MR approach, using genetic variants of the GM as IVs, to infer the potential causality with cardiac structure and function, effectively mitigating the impact of confounders. Our study results lend support to the notion that certain specific GM may act as potential causal factors affecting cardiac structure and function.

Gene annotation and informatics term clustering analysis (Hendrickx et al., 2020) based on GWAS data may provide insights into the potential mechanisms underlying the causal relationship between GM and cardiac phenotypes. Among these shared genes, *TTN* and *NPR3* are identified as important genes that may mediate the biological connection between GM and cardiac phenotypes according to current knowledge. The *TTN* gene encodes Titin, a colossal protein that is a critical component of the contractile unit in cardiomyocytes, playing a significant role in maintaining passive tension within cardiac cells (LeWinter et al., 2007). Truncating mutations in the *TTN* gene are common genetic causes of dilated cardiomyopathy (Herman et al., 2012), leading to ventricular dilation and impaired contractile function. On the other hand, the *NPR3* gene codes for natriuretic peptide receptor 3, which plays an essential role in the clearance of natriuretic peptides (Potter, 2011). Natriuretic peptides significantly affect cardiac structure and function by modulating the RAAS, sodium and water excretion, and vascular tone (Sarzani et al., 2022). Genetic variations in *NPR3* have also been associated with phenotypes such as blood pressure and obesity (Sarzani et al., 2004; Kato et al., 2011; Saulnier et al., 2011). However, for the other three genes, *PDE11A*, *SLC28A1*, and *PLEKHA3*, there is insufficient evidence to support a direct link with cardiac structure and function. Informatics term clustering analysis has identified multiple biological processes shared between GM causally linked to cardiac structure and related cardiac structural parameters, primarily involving cardiomyocyte development, signal transduction, and transmembrane calcium ion transport. Additionally, these processes share the *MAPK signaling pathway*, which has been extensively validated in relation to heart development, hypertrophy, and pathological remodeling (Rose et al., 2010; Turner and Blythe, 2019; Romero-Becerra et al., 2020). At the functional level, the biological processes shared between GM and cardiac function extend beyond cardiomyocyte development to include regulation of cardiac contraction and actin-myosin filament sliding, which are crucial for cardiac function (Wang and Raunser, 2023). In summary, subsequent biological annotation analysis based on causality-driven MR analysis provides new insights into the mechanisms by which GM influences cardiac structure and function, offering important clues for further investigation into the “gut-heart” axis interaction. Nonetheless, further research is required to validate the specific mechanisms involved.

This study boasts several significant strengths and some noteworthy limitations. First and foremost, to our knowledge, this is the inaugural MR study to explore the causal relationship between the GM and cardiac structure and function, which may provide valuable insights into identifying potential residual risk factors that affect cardiac structure and function. The application of MR considerably mitigates confounding and reverse causation biases (Smith and Ebrahim, 2004), thereby strengthening the causal inference between the GM and cardiac phenotypes. Furthermore, by integrating a variety of MR analytical approaches and conducting a series of sensitivity analyses, we have enhanced the robustness of the causal inference.

Nonetheless, certain limitations should be acknowledged. Firstly, MR analysis utilizes genetic variants as IVs to infer the association between exposures and outcomes. This approach essentially relies on regression between the IVs and the outcome, rather than the exposure itself. In this study, the abundance of GM, serving as an exposure, is influenced not only by genetic factors but also by a multitude of factors such as diet, exercise, and emotional state (Campaniello et al., 2022). Therefore, causal inferences drawn from the effects of genetic variants should be interpreted with caution. Moreover, MR analysis emphasizes the lifelong impact of exposures on outcomes, yet the GM exhibits high variability (Zhernakova et al., 2016), suggesting that some causal inferences might be overestimated. Although MR is not considered the gold standard for assessing causality, our research aims to explore the potential causal relationship between GM and cardiac structure and function, and to evaluate the potential of GM as a contributing residual risk factor for cardiac phenotypes. This is also why we did not perform multiple testing correction during the statistical analysis stage, even though it may increase the risk of committing Type I errors. We aspire to reveal broader impacts of GM on cardiac structure and function, aiming to provide valuable biological insights for the design of future RCTs (Ference et al., 2021). Additionally, the majority of data used in this MR study were all from populations of European ancestry, which may limit the generalization of the findings to other populations. Future studies should include more diverse samples to examine the effect of GM on the risk of cardiac structure and function in different ethnic groups.

## 5 Conclusion

This MR study has uncovered a broad spectrum of genetically predicted causal associations between the GM and cardiac structure and function, and has provided a preliminary exploration into the potential mechanisms. These findings provide evidence for considering GM as a potential causal residual risk factor that may influence cardiac structure and function. However, further RCTs are still required to reinforce and confirm the causal association between GM and cardiac structure and function.

## Data availability statement

Publicly available datasets were analyzed in this study. These data can be found here: Gut microbiota: https://mibiogen.gcc.rug.nl/menu/main/home/; Cardiac structure and function: https://cvd.hugeamp.org/downloads.html#summary and https://zenodo.org/records/5074929.

## Author contributions

YL: Conceptualization, Methodology, Writing – original draft, Software. MY: Writing – original draft, Methodology. FX: Investigation, Methodology, Writing – original draft. YQ: Data curation, Funding acquisition, Writing – review & editing. XZ: Data curation, Writing – review & editing, Project administration. RL: Data curation, Writing – review & editing, Conceptualization, Funding acquisition.

## Supplementary material

SUPPLEMENTARY TABLE S1 STROBE-MR checklist.

SUPPLEMENTARY TABLE S2 Information of 119 gut microbiota genera.

SUPPLEMENTARY TABLE S3 Instrumental variables of 119 gut microbiota genera on 16 cardiac structural and functional parameters.

SUPPLEMENTARY TABLE S4 The complete results of Mendelian randomization estimates and sensitivity analyses.

SUPPLEMENTARY TABLE S5 The information of mapping genes for gut microbiota and cardiac structure and function.

SUPPLEMENTARY TABLE S6 Results of Gene Ontology biological process analysis and KEGG pathway enrichment.

SUPPLEMENTARY FIGURE S1 Leave-one-out analyses for gut microbiota on cardiac structure.

SUPPLEMENTARY FIGURE S2 Leave-one-out analyses for gut microbiota on cardiac function.

SUPPLEMENTARY FIGURE S3 Scatter plots for gut microbiota on cardiac structure

SUPPLEMENTARY FIGURE S4 Scatter plots for gut microbiota on cardiac function.
