# Supplementary material for: Gut microbiota as a residual risk factor causally influencing cardiac structure and function: Mendelian randomization analysis and biological annotation
Source: Front Microbiol. 2024 Jul 26;15:1410272. doi: 10.3389/fmicb.2024.1410272 (PMC11316272; doi:10.3389/fmicb.2024.1410272)
Supplement: SUPPLEMENTARY FIGURE S1 — Leave-one-out analyses for gut microbiota on cardiac structure. [file Data_Sheet_1.PDF]

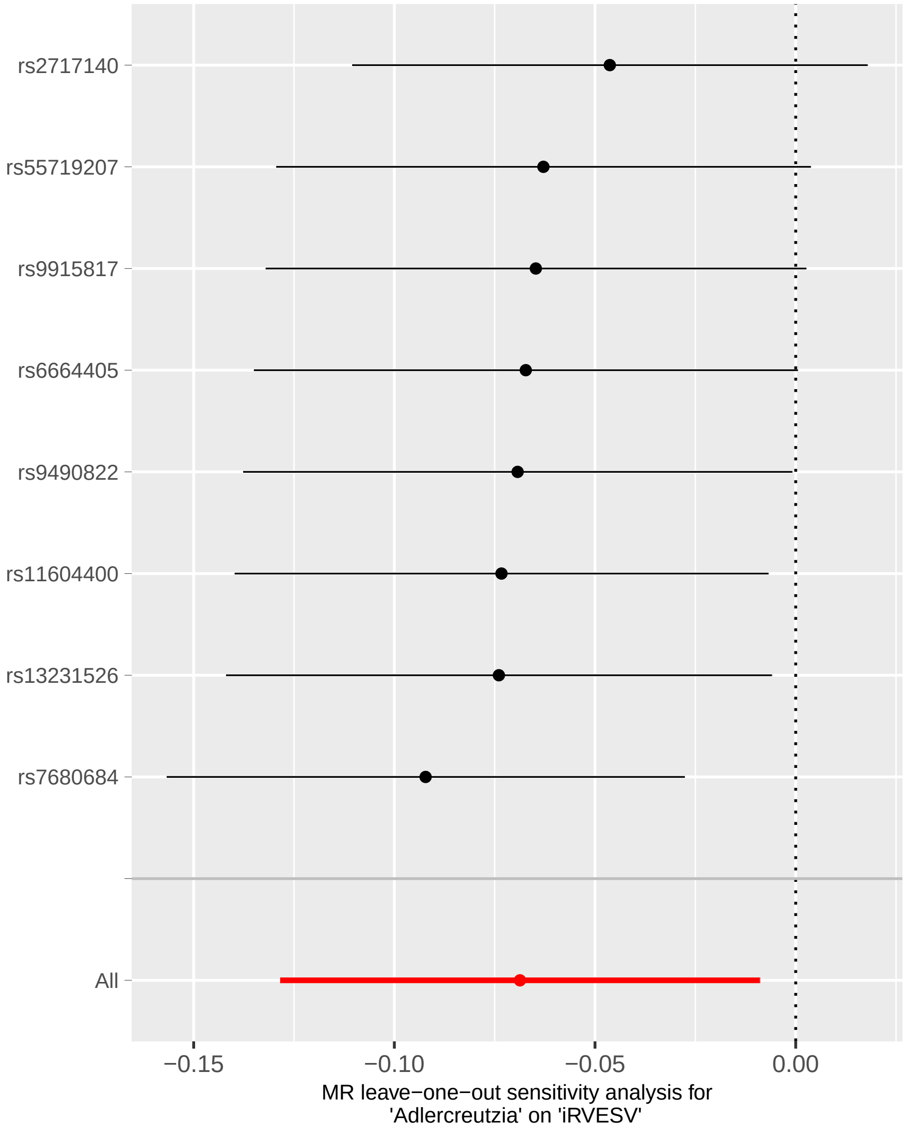
rs2717140
rs55719207
rs9915817
rs6664405
rs9490822
rs11604400
rs13231526
rs7680684
All
−0.15
−0.10
−0.05
0.00
MR leave−one−out sensitivity analysis for
'Adlercreutzia' on 'iRVESV'

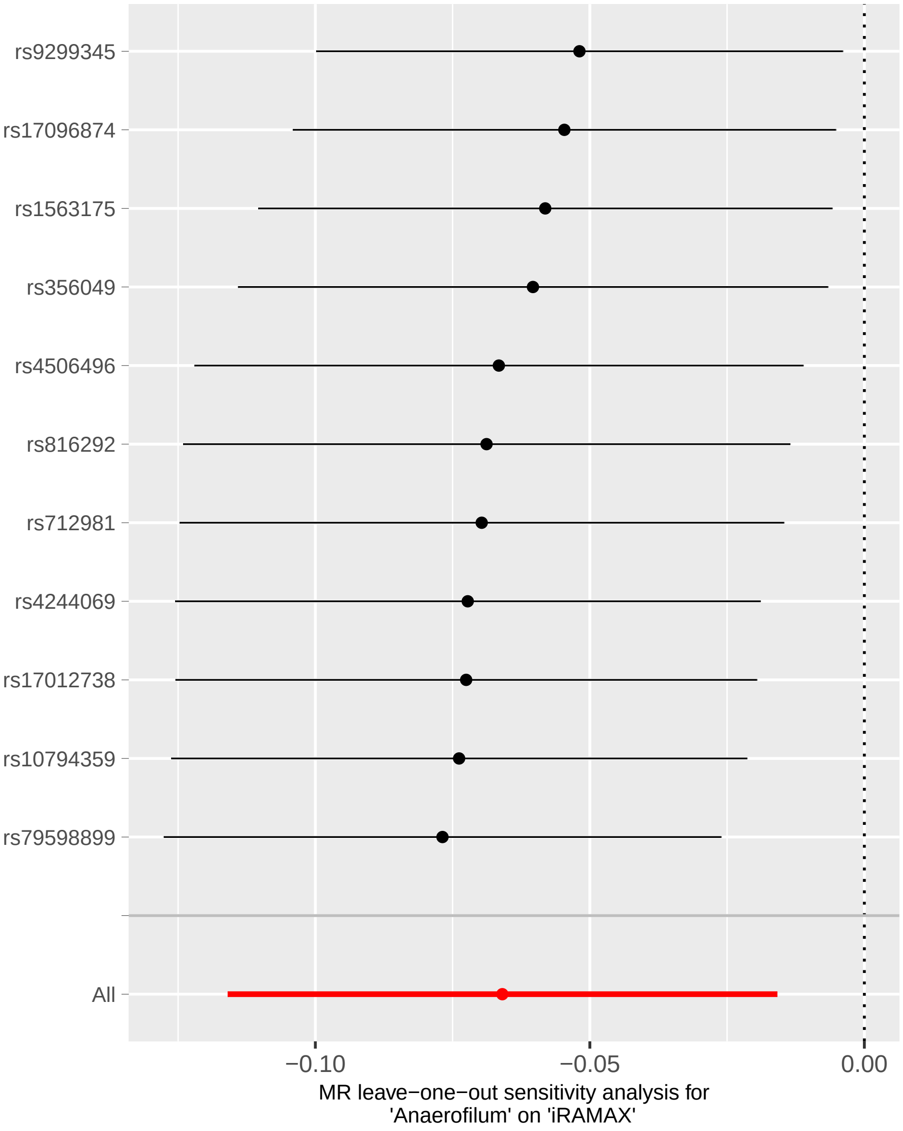

rs9299345
rs17096874
rs1563175
rs356049
rs4506496
rs816292
rs712981
rs4244069
rs17012738
rs10794359
rs79598899
All
−0.10
−0.05
0.00
MR leave−one−out sensitivity analysis for
'Anaerofilum' on 'iRAMAX'

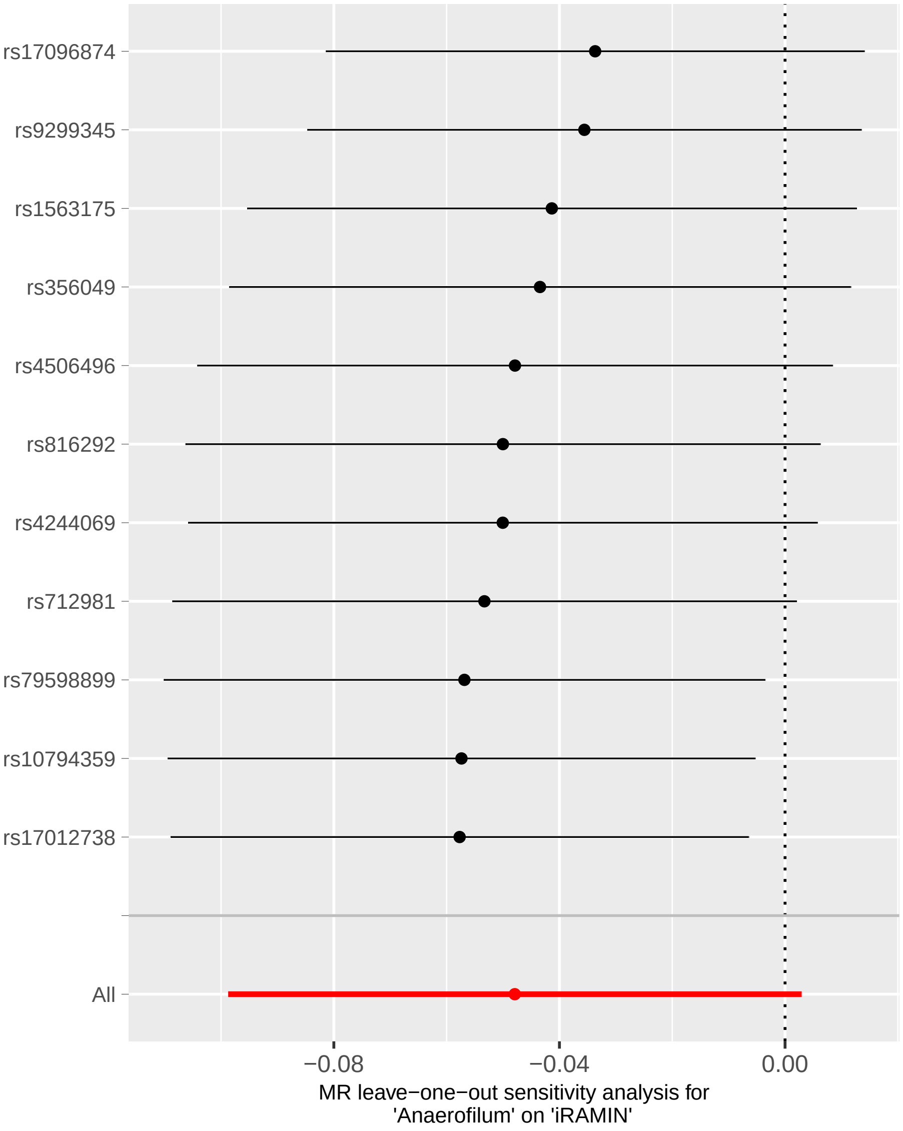
rs17096874
rs9299345
rs1563175
rs356049
rs4506496
rs816292
rs4244069
rs712981
rs79598899
rs10794359
rs17012738
All
−0.08
−0.04
0.00
MR leave−one−out sensitivity analysis for
'Anaerofilum' on 'iRAMIN'

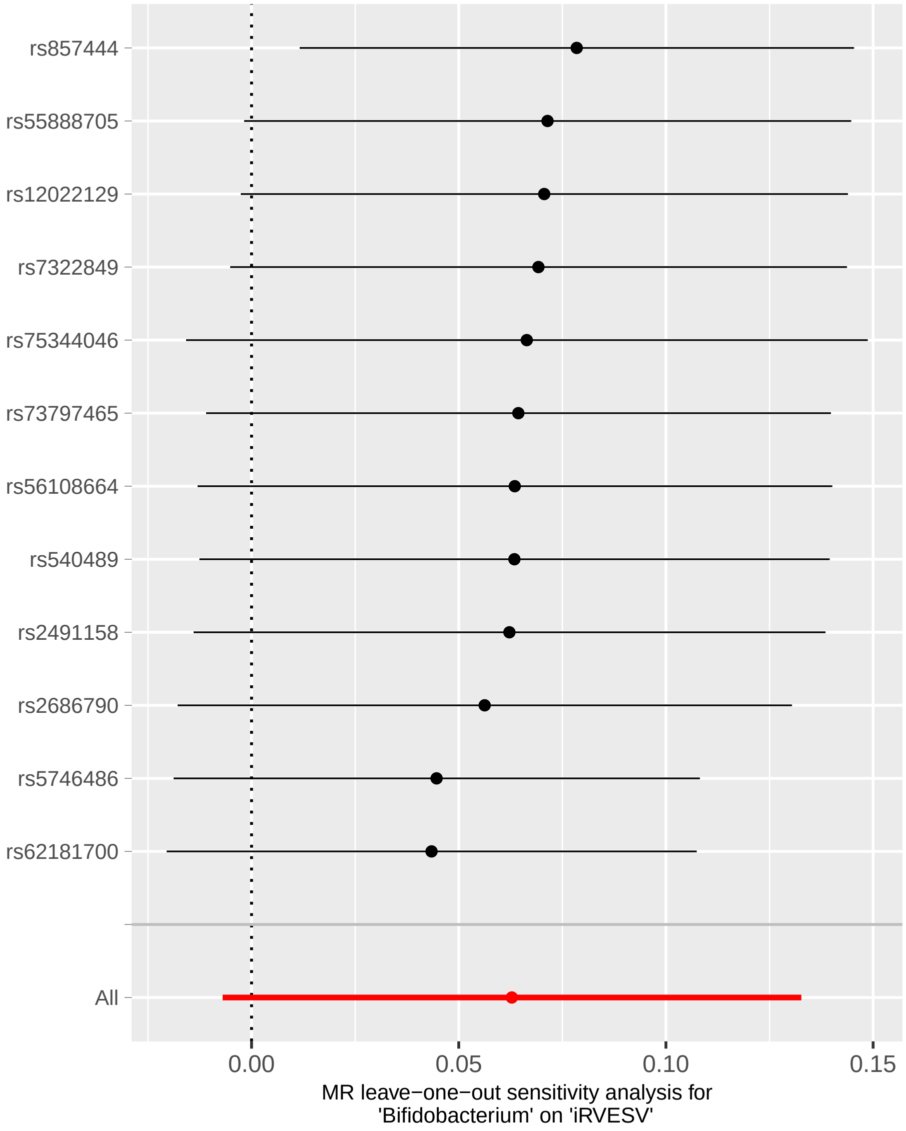
rs857444
rs55888705
rs12022129
rs7322849
rs75344046
rs73797465
rs56108664
rs540489
rs2491158
rs2686790
rs5746486
rs62181700
All
0.00
0.05
0.10
0.15
MR leave−one−out sensitivity analysis for
'Bifidobacterium' on 'iRVESV'

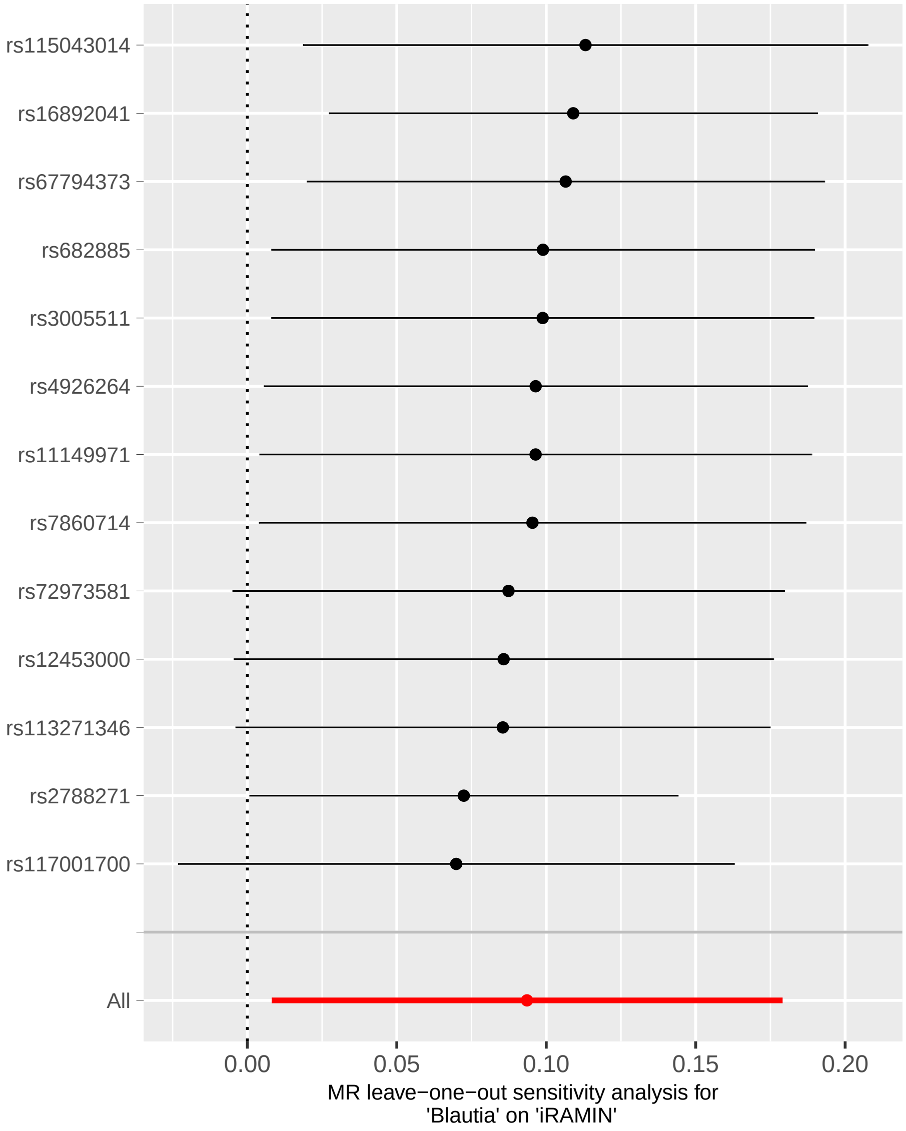
rs115043014
rs16892041
rs67794373
rs682885
rs3005511
rs4926264
rs11149971
rs7860714
rs72973581
rs12453000
rs113271346
rs2788271
rs117001700
All
0.00
0.05
0.10
0.15
0.20
MR leave−one−out sensitivity analysis for
'Blautia' on 'iRAMIN'

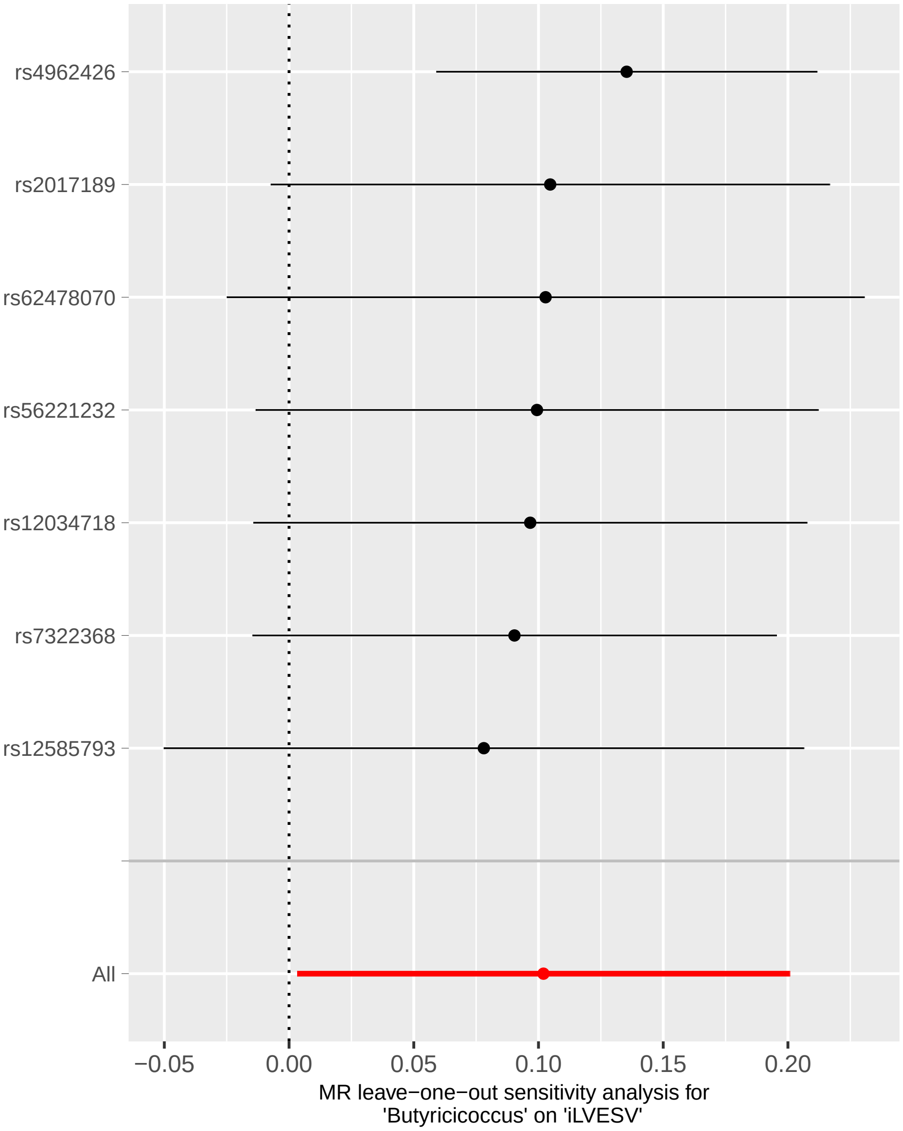
rs4962426
rs2017189
rs62478070
rs56221232
rs12034718
rs7322368
rs12585793
All
−0.05
0.00
0.05
0.10
0.15
0.20
MR leave−one−out sensitivity analysis for
'Butyricicoccus' on 'iLVESV'

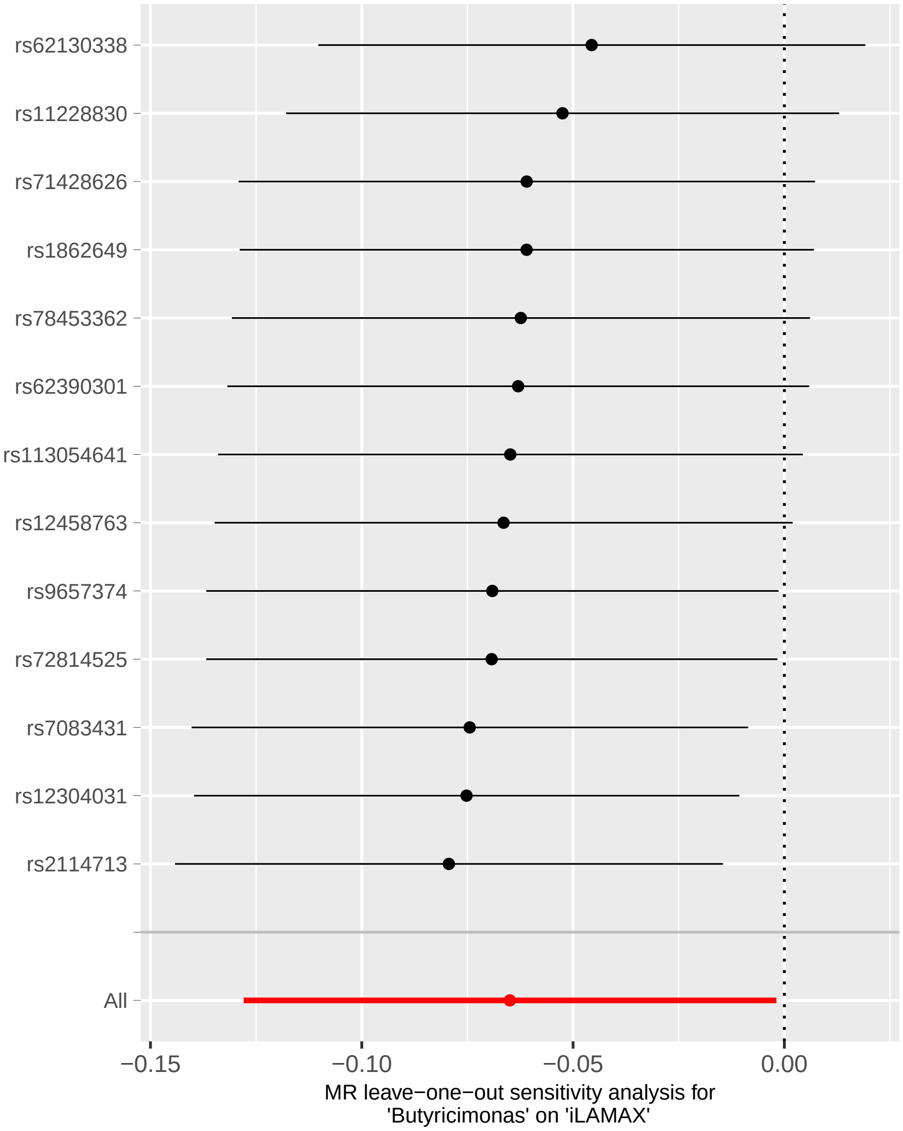
rs62130338
rs11228830
rs71428626
rs1862649
rs78453362
rs62390301
rs113054641
rs12458763
rs9657374
rs72814525
rs7083431
rs12304031
rs2114713
All
−0.15
−0.10
−0.05
0.00
MR leave−one−out sensitivity analysis for
'Butyricimonas' on 'iLAMAX'

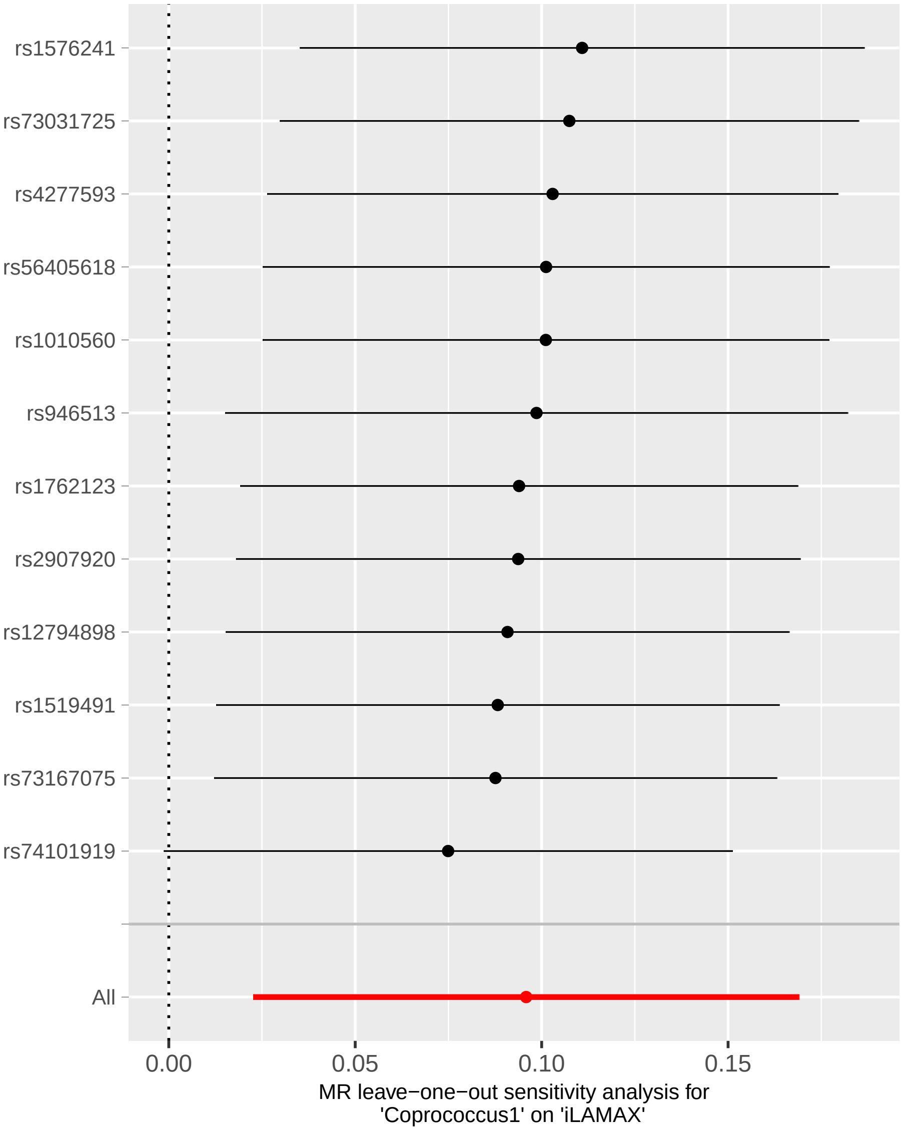
rs1576241
rs73031725
rs4277593
rs56405618
rs1010560
rs946513
rs1762123
rs2907920
rs12794898
rs1519491
rs73167075
rs74101919
All
0.00
0.05
0.10
0.15
MR leave−one−out sensitivity analysis for
'Coprococcus1' on 'iLAMAX'

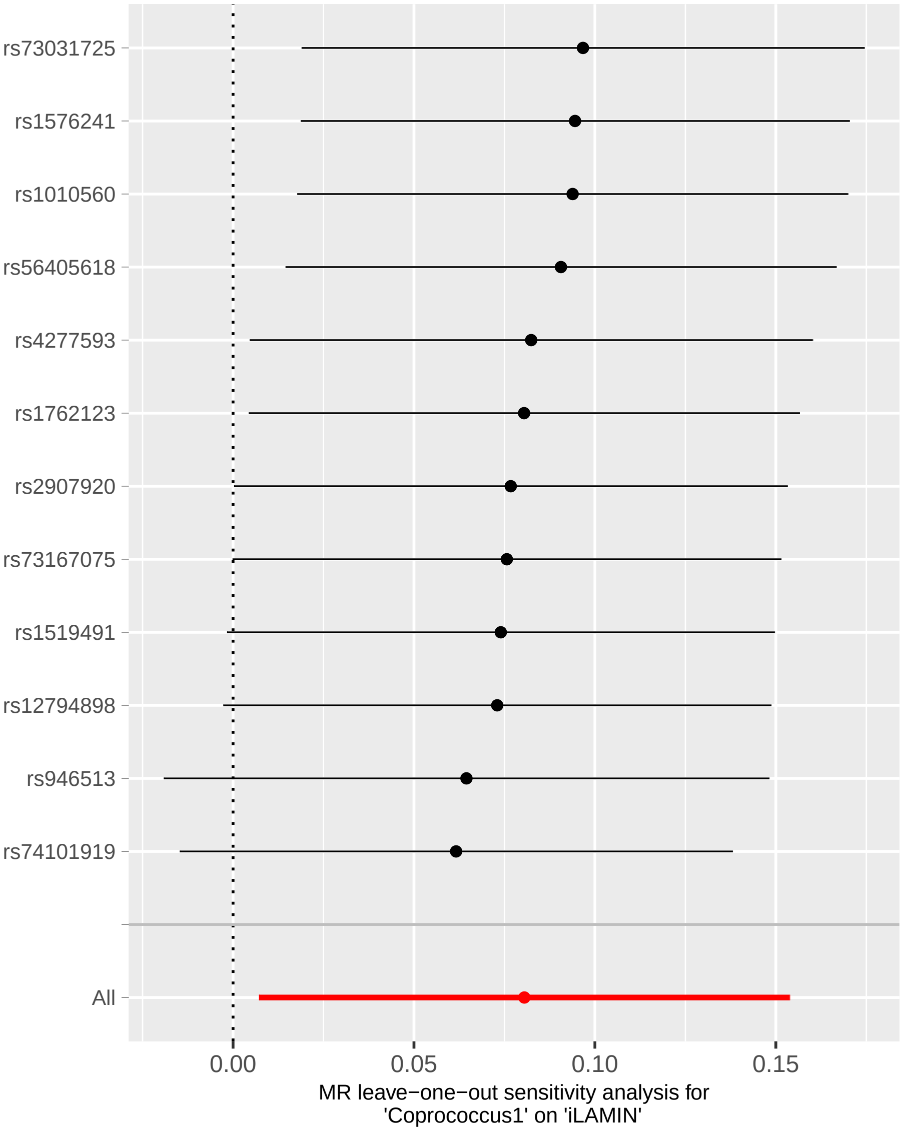
rs73031725
rs1576241
rs1010560
rs56405618
rs4277593
rs1762123
rs2907920
rs73167075
rs1519491
rs12794898
rs946513
rs74101919
All
0.00
0.05
0.10
0.15
MR leave−one−out sensitivity analysis for
'Coprococcus1' on 'iLAMIN'

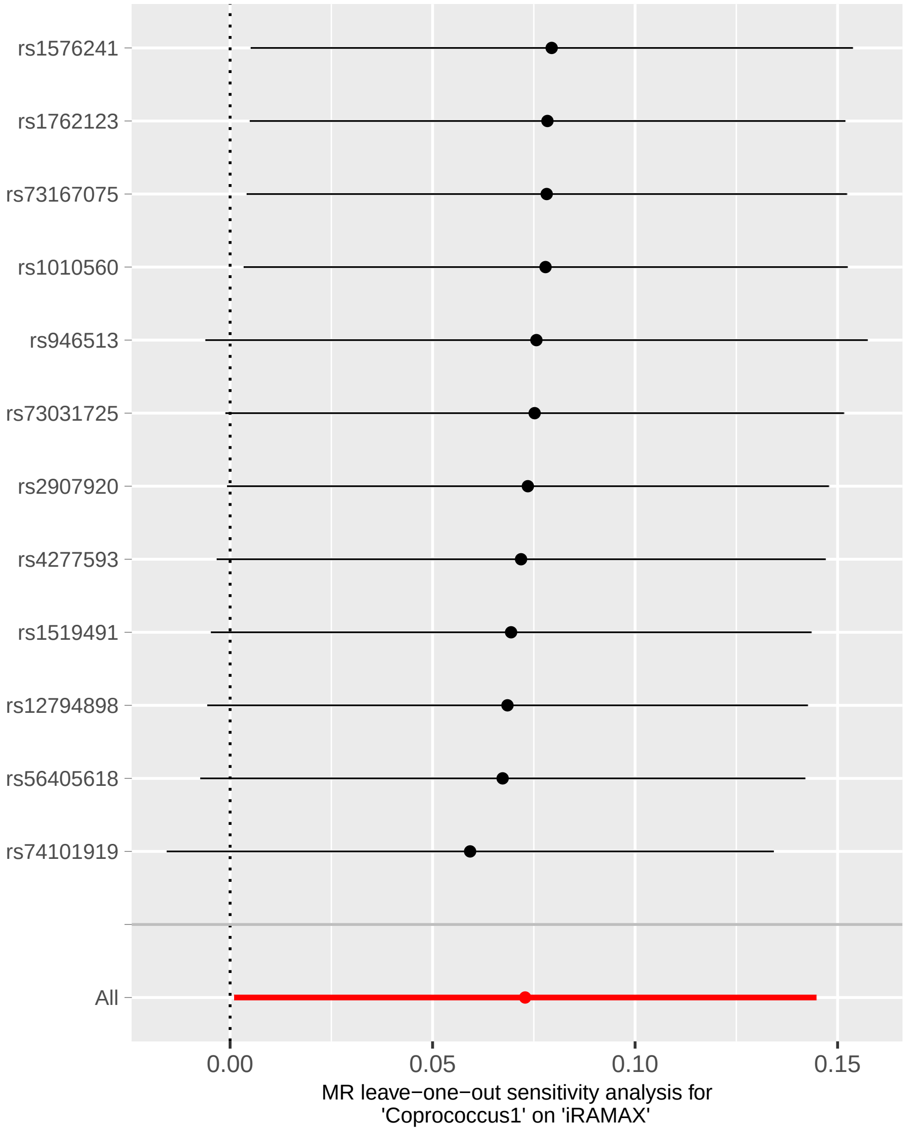

rs1576241
rs1762123
rs73167075
rs1010560
rs946513
rs73031725
rs2907920
rs4277593
rs1519491
rs12794898
rs56405618
rs74101919
All
0.00
0.05
0.10
0.15
MR leave−one−out sensitivity analysis for
'Coprococcus1' on 'iRAMAX'

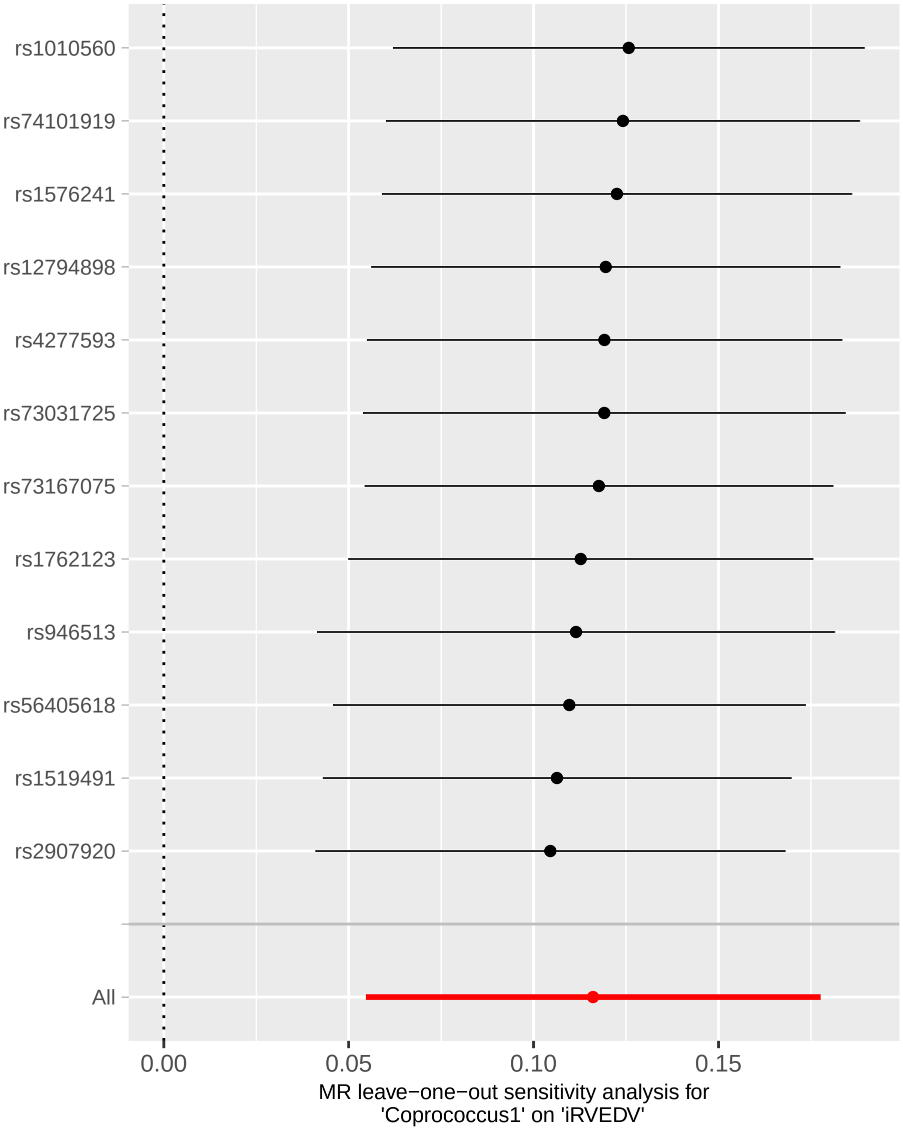

rs1010560
rs74101919
rs1576241
rs12794898
rs4277593
rs73031725
rs73167075
rs1762123
rs946513
rs56405618
rs1519491
rs2907920
All
0.00
0.05
0.10
0.15
MR leave−one−out sensitivity analysis for
'Coprococcus1' on 'iRVEDV'

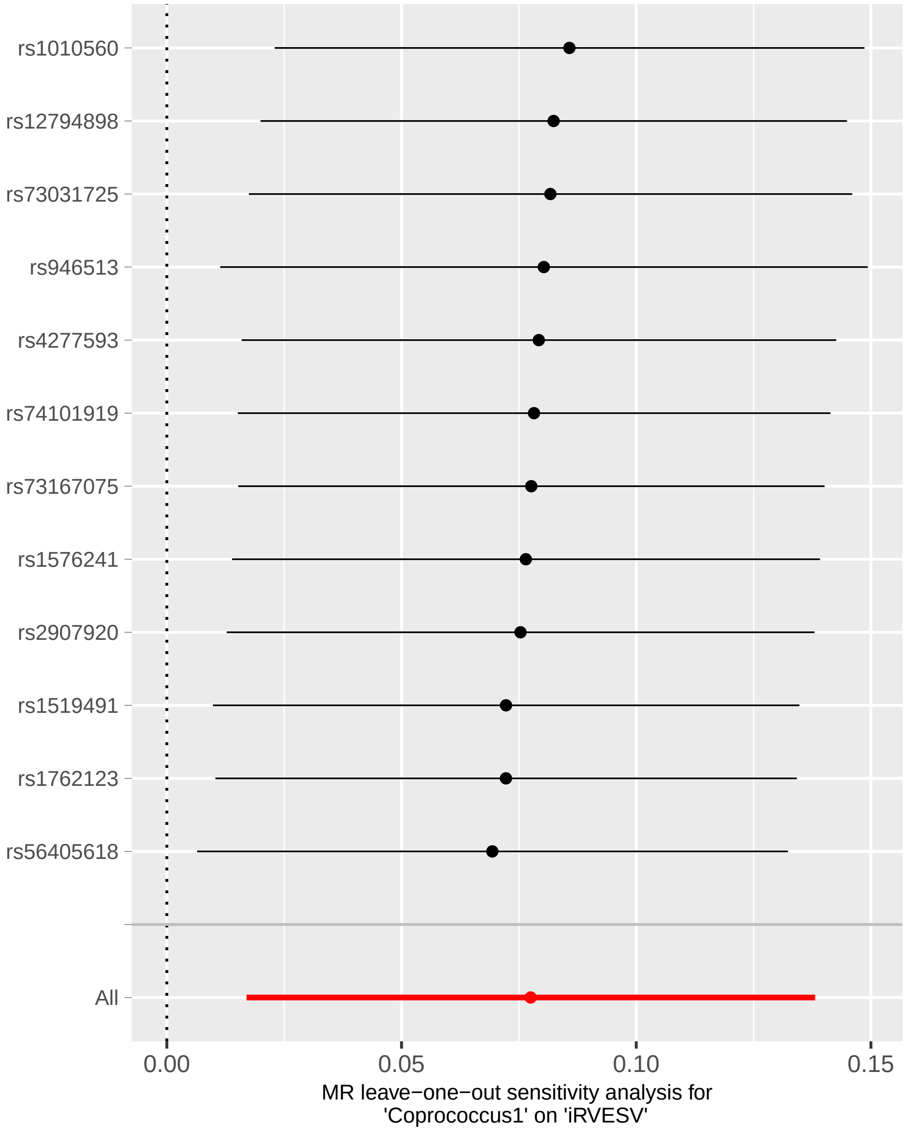

rs1010560
rs12794898
rs73031725
rs946513
rs4277593
rs74101919
rs73167075
rs1576241
rs2907920
rs1519491
rs1762123
rs56405618
All
0.00
0.05
0.10
0.15
MR leave−one−out sensitivity analysis for
'Coprococcus1' on 'iRVESV'

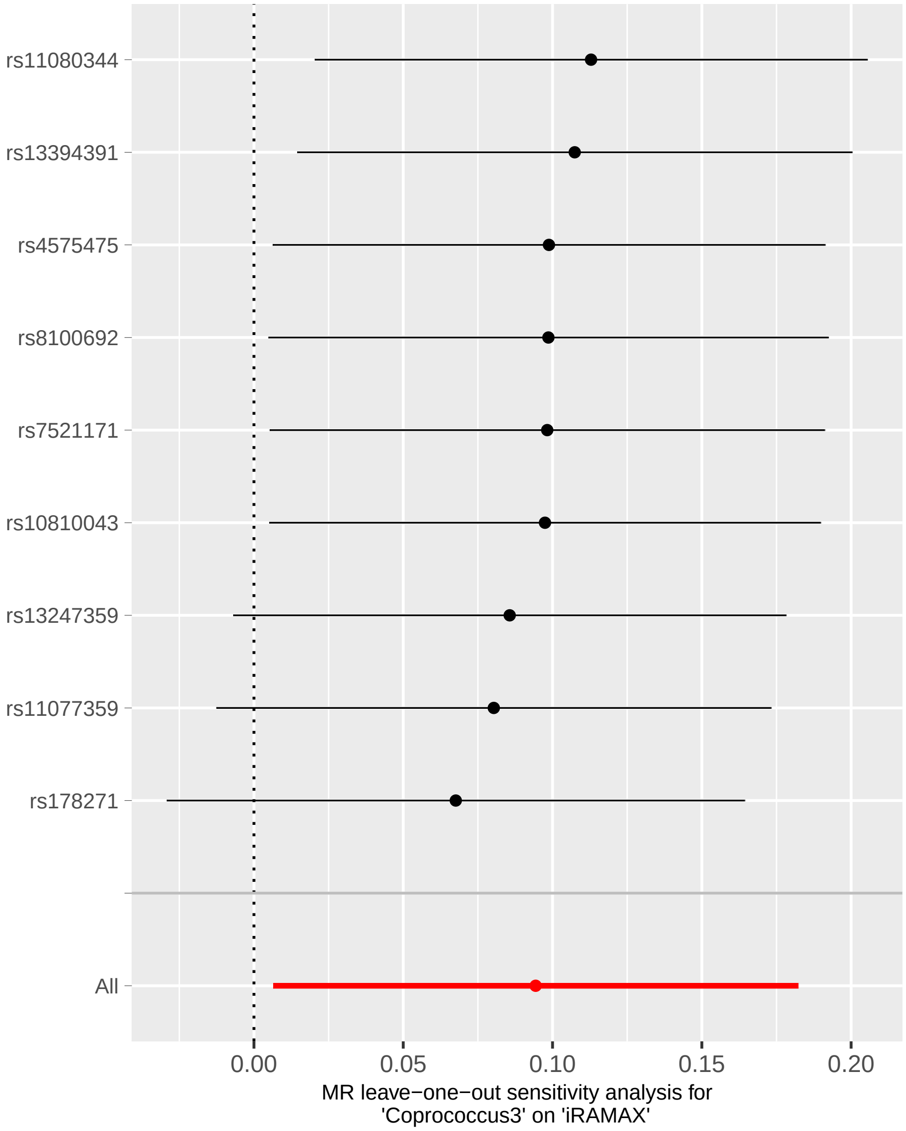
rs11080344
rs13394391
rs4575475
rs8100692
rs7521171
rs10810043
rs13247359
rs11077359
rs178271
All
0.00
0.05
0.10
0.15
0.20
MR leave−one−out sensitivity analysis for
'Coprococcus3' on 'iRAMAX'

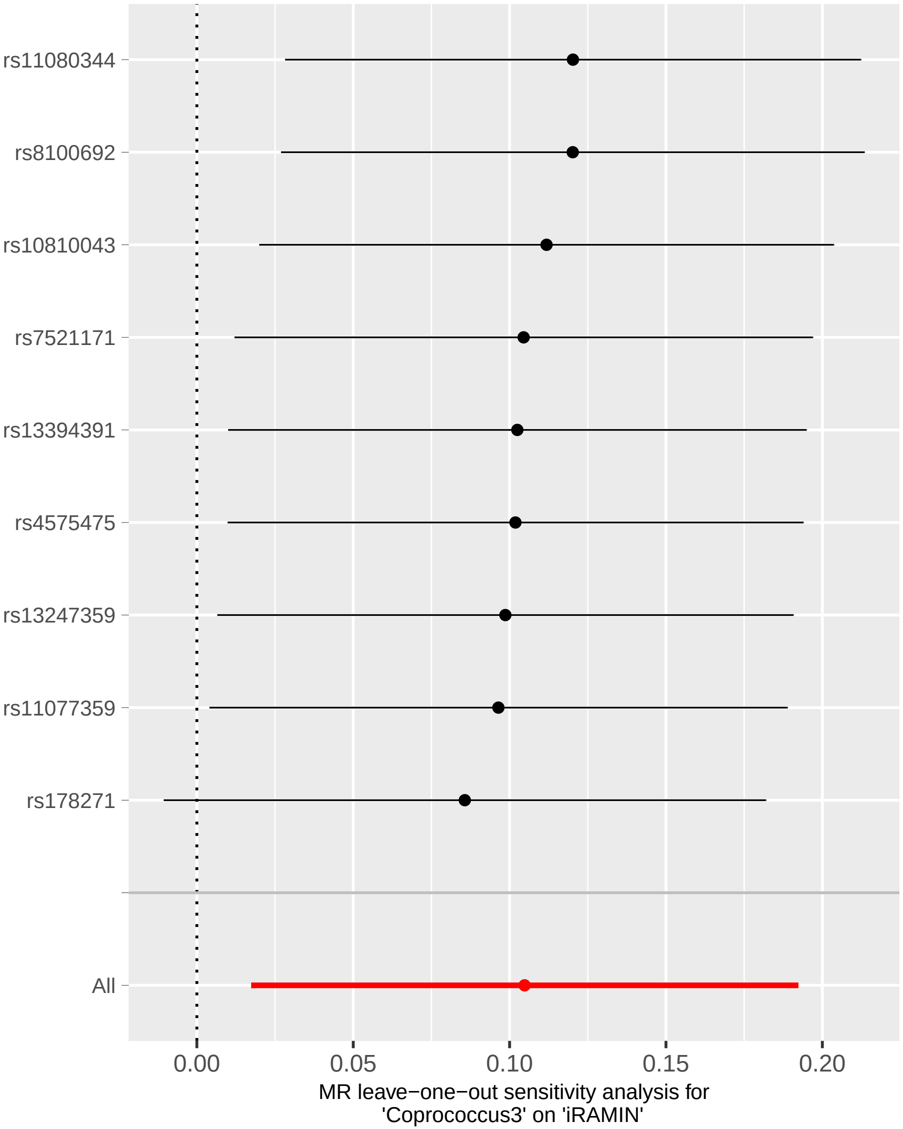

rs11080344
rs8100692
rs10810043
rs7521171
rs13394391
rs4575475
rs13247359
rs11077359
rs178271
All
0.00
0.05
0.10
0.15
0.20
MR leave−one−out sensitivity analysis for
'Coprococcus3' on 'iRAMIN'

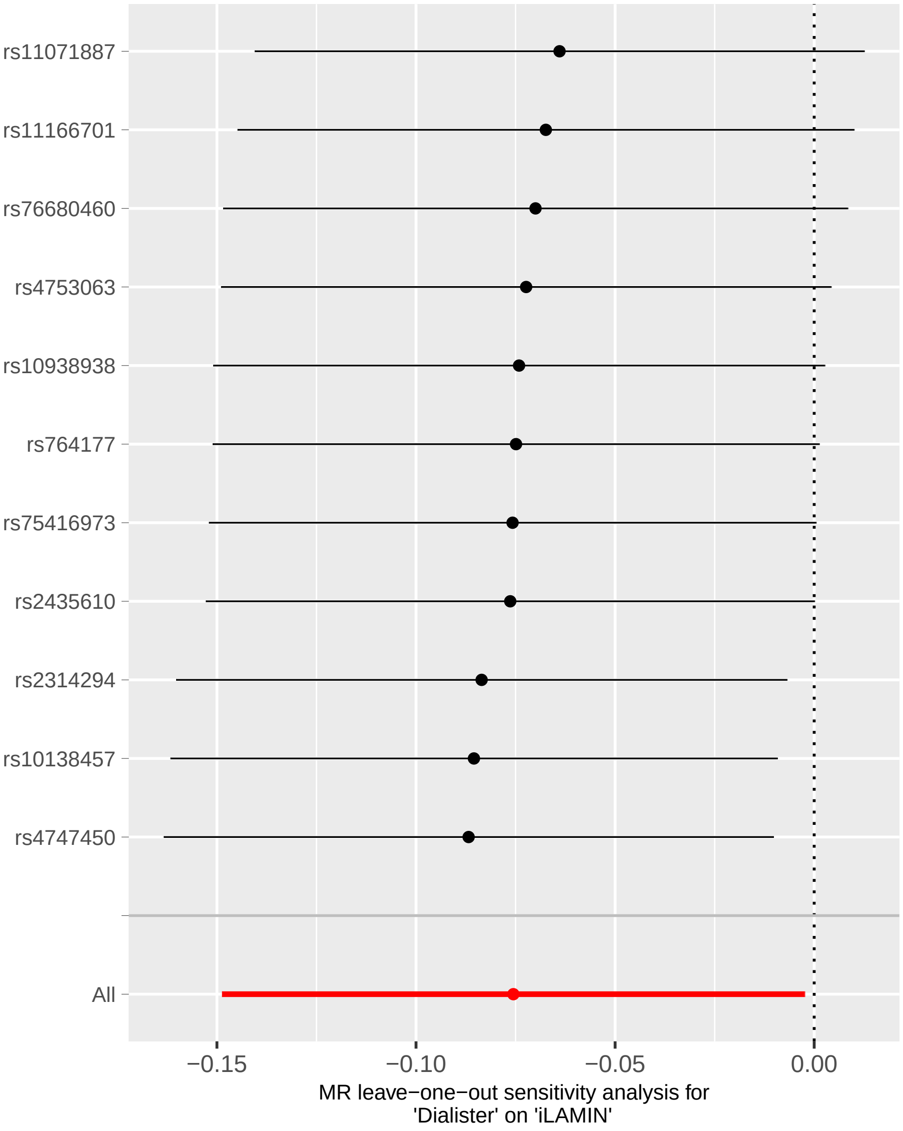
rs11071887
rs11166701
rs76680460
rs4753063
rs10938938
rs764177
rs75416973
rs2435610
rs2314294
rs10138457
rs4747450
All
−0.15
−0.10
−0.05
0.00
MR leave−one−out sensitivity analysis for
'Dialister' on 'iLAMIN'

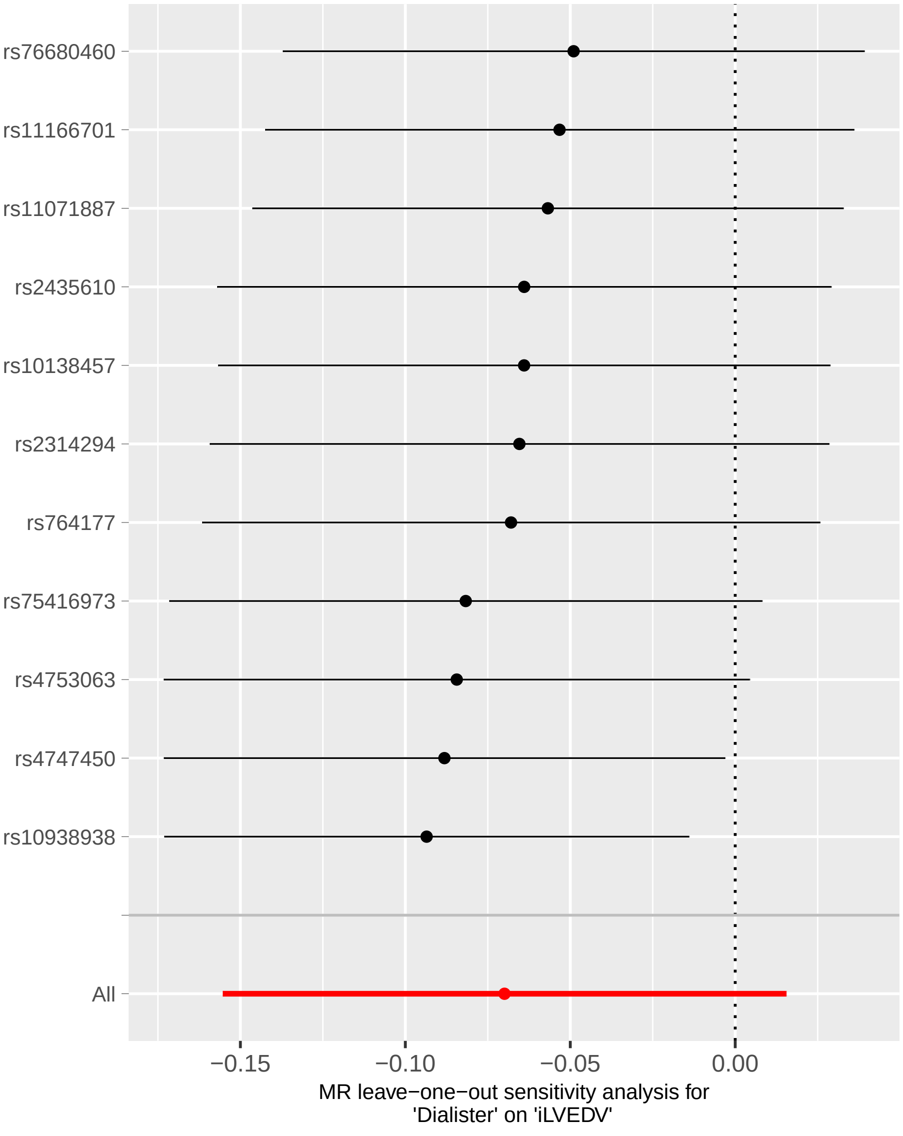
rs76680460
rs11166701
rs11071887
rs2435610
rs10138457
rs2314294
rs764177
rs75416973
rs4753063
rs4747450
rs10938938
All
−0.15
−0.10
−0.05
0.00
MR leave−one−out sensitivity analysis for
'Dialister' on 'iLVEDV'

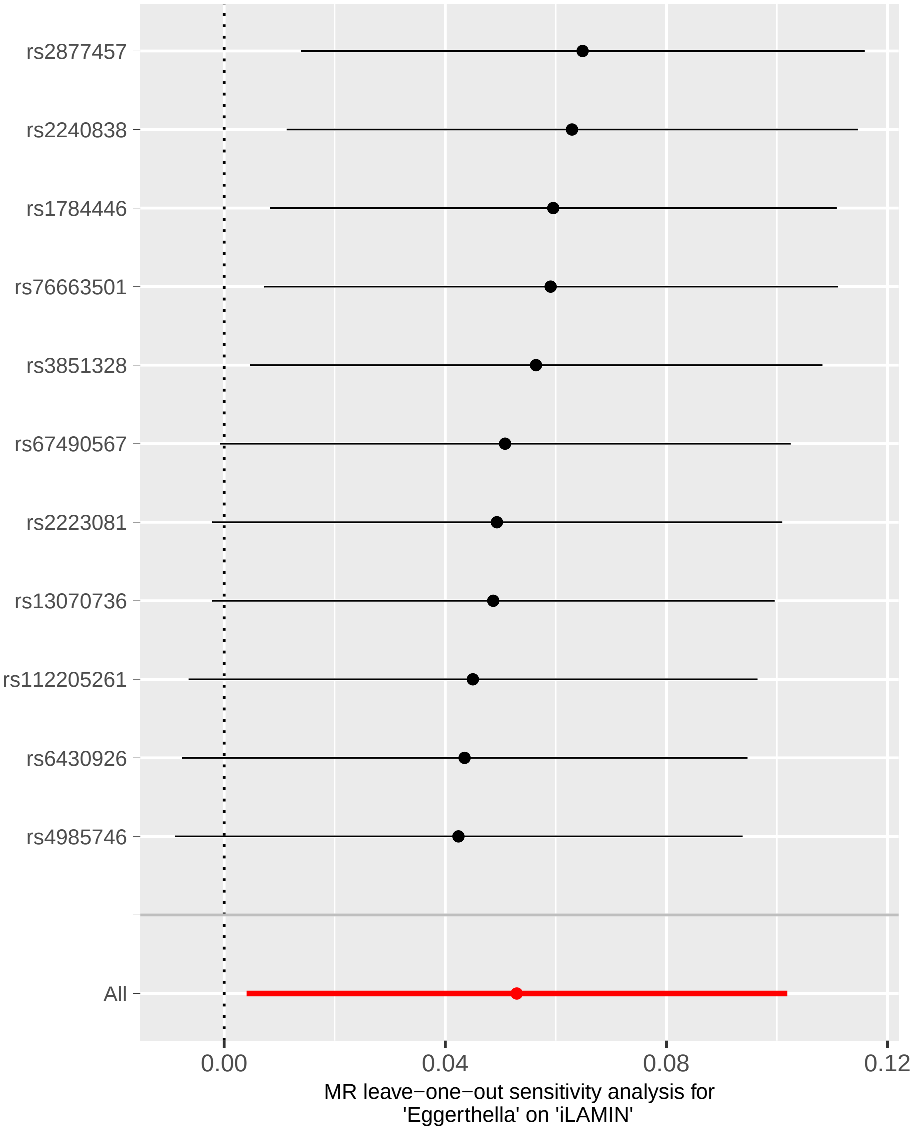
rs2877457
rs2240838
rs1784446
rs76663501
rs3851328
rs67490567
rs2223081
rs13070736
rs112205261
rs6430926
rs4985746
All
0.00
0.04
0.08
0.12
MR leave−one−out sensitivity analysis for
'Eggerthella' on 'iLAMIN'

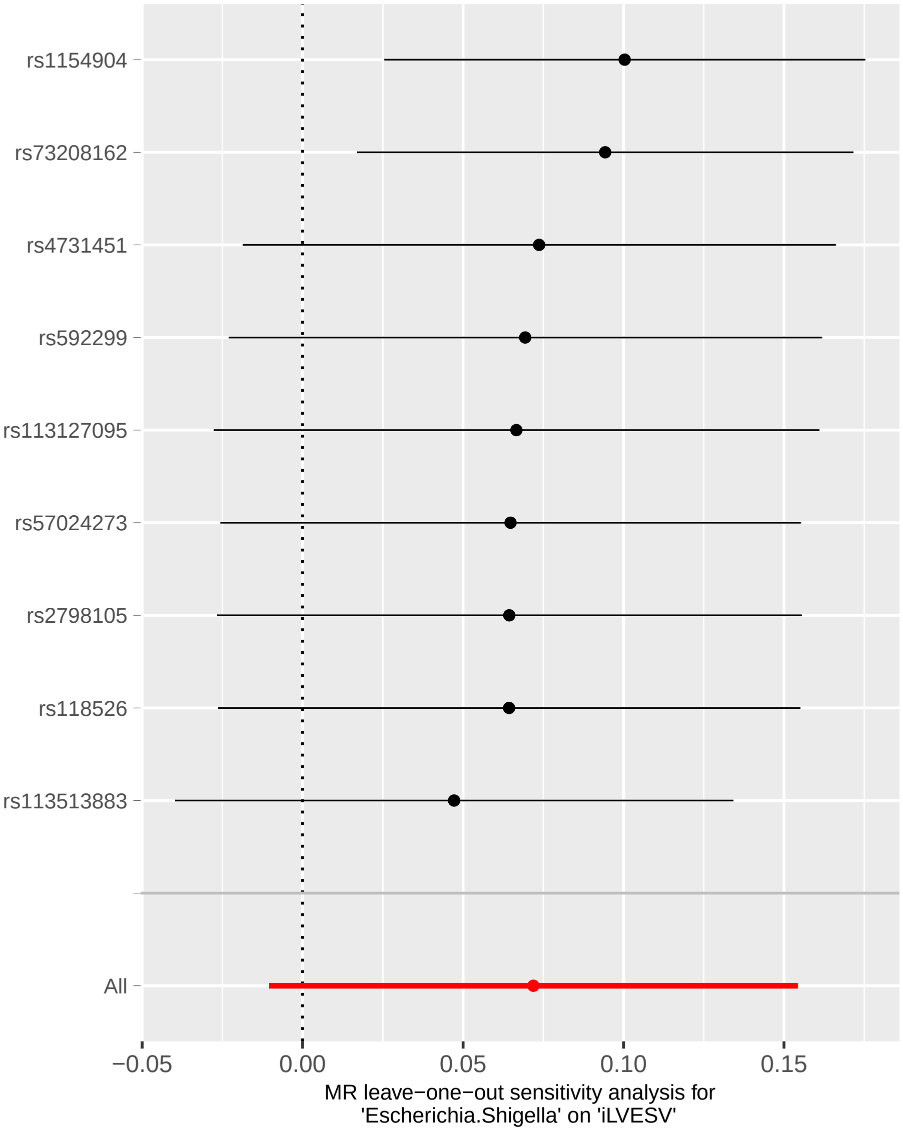
rs1154904
rs73208162
rs4731451
rs592299
rs113127095
rs57024273
rs2798105
rs118526
rs113513883
All
−0.05
0.00
0.05
0.10
0.15
MR leave−one−out sensitivity analysis for
'Escherichia.Shigella' on 'iLVESV'

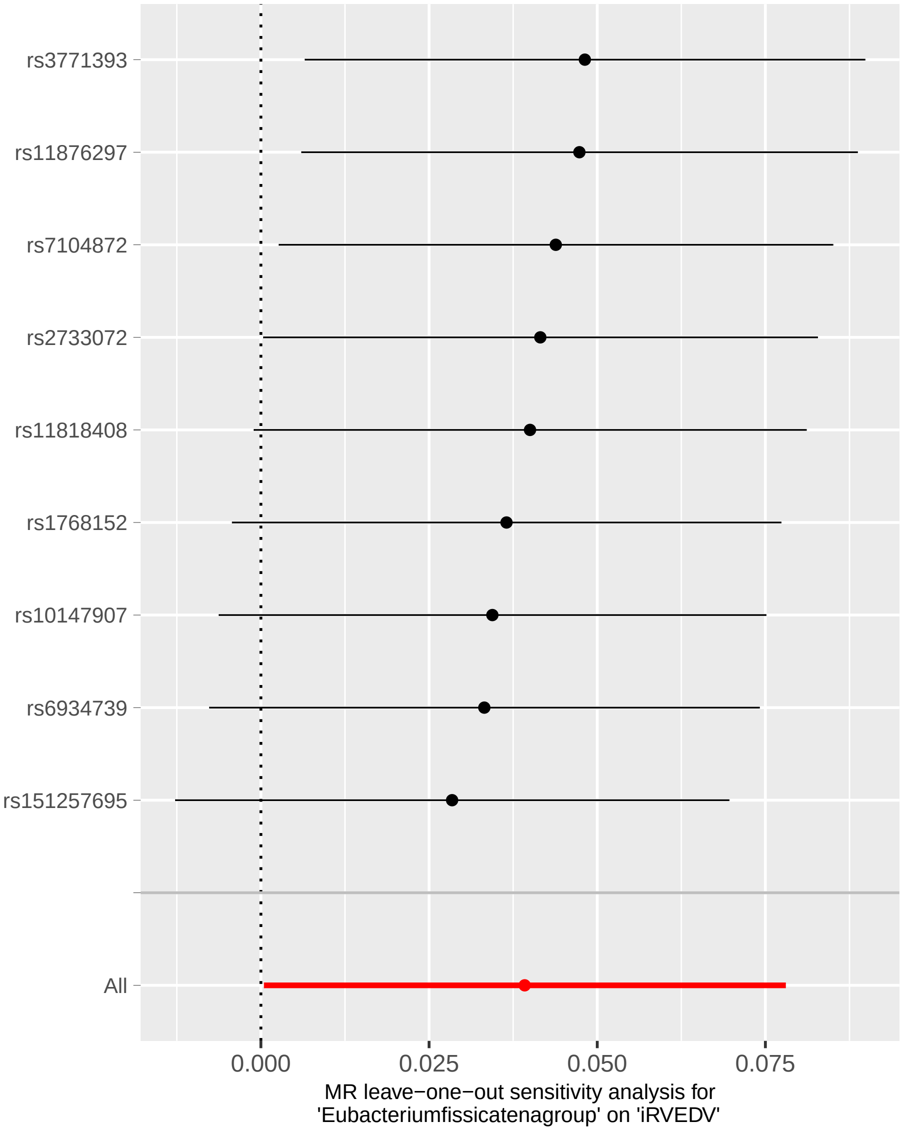

rs3771393
rs11876297
rs7104872
rs2733072
rs11818408
rs1768152
rs10147907
rs6934739
rs151257695
All
0.000
0.025
0.050
0.075
MR leave−one−out sensitivity analysis for
'Eubacteriumfissicatenagroup' on 'iRVEDV'

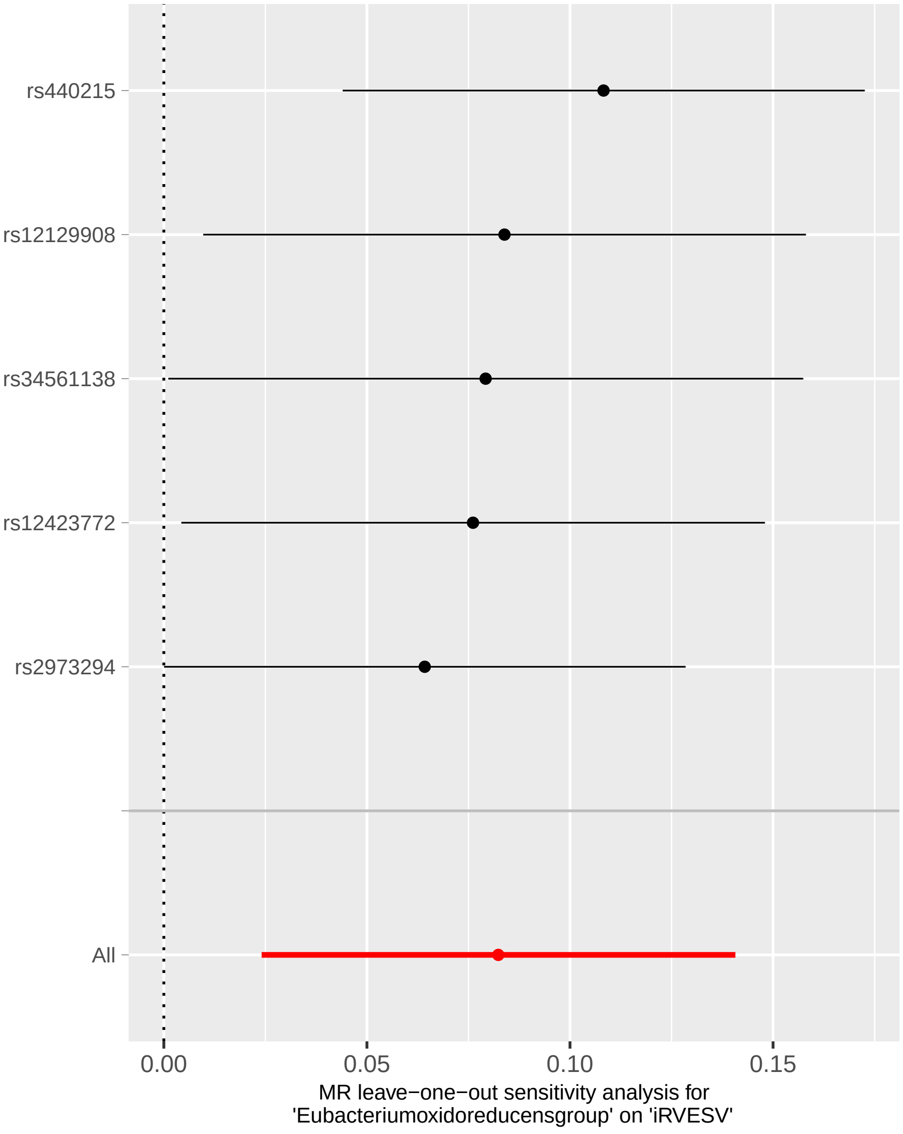
rs440215
rs12129908
rs34561138
rs12423772
rs2973294
All
0.00
0.05
0.10
0.15
MR leave−one−out sensitivity analysis for
'Eubacteriumoxidoreducensgroup' on 'iRVESV'

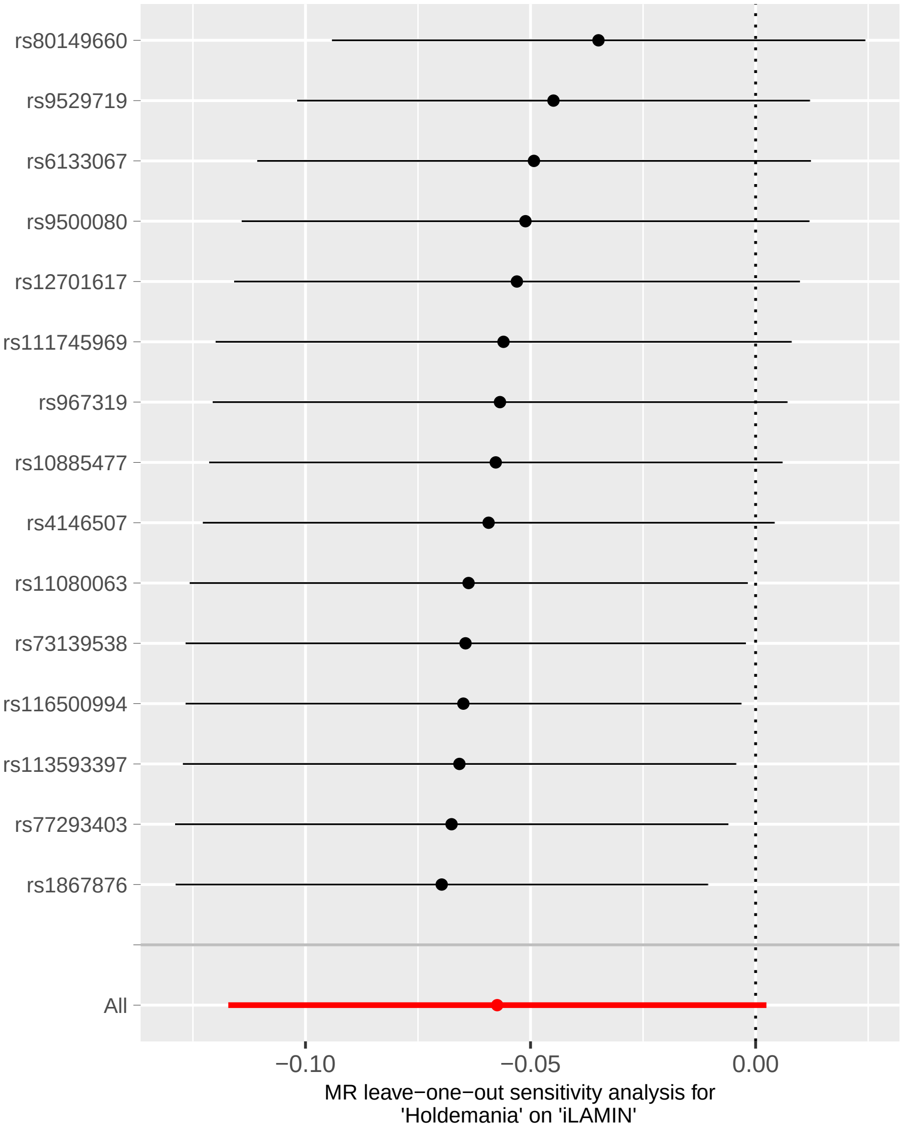

rs80149660
rs9529719
rs6133067
rs9500080
rs12701617
rs111745969
rs967319
rs10885477
rs4146507
rs11080063
rs73139538
rs116500994
rs113593397
rs77293403
rs1867876
All
−0.10
−0.05
0.00
MR leave−one−out sensitivity analysis for
'Holdemania' on 'iLAMIN'

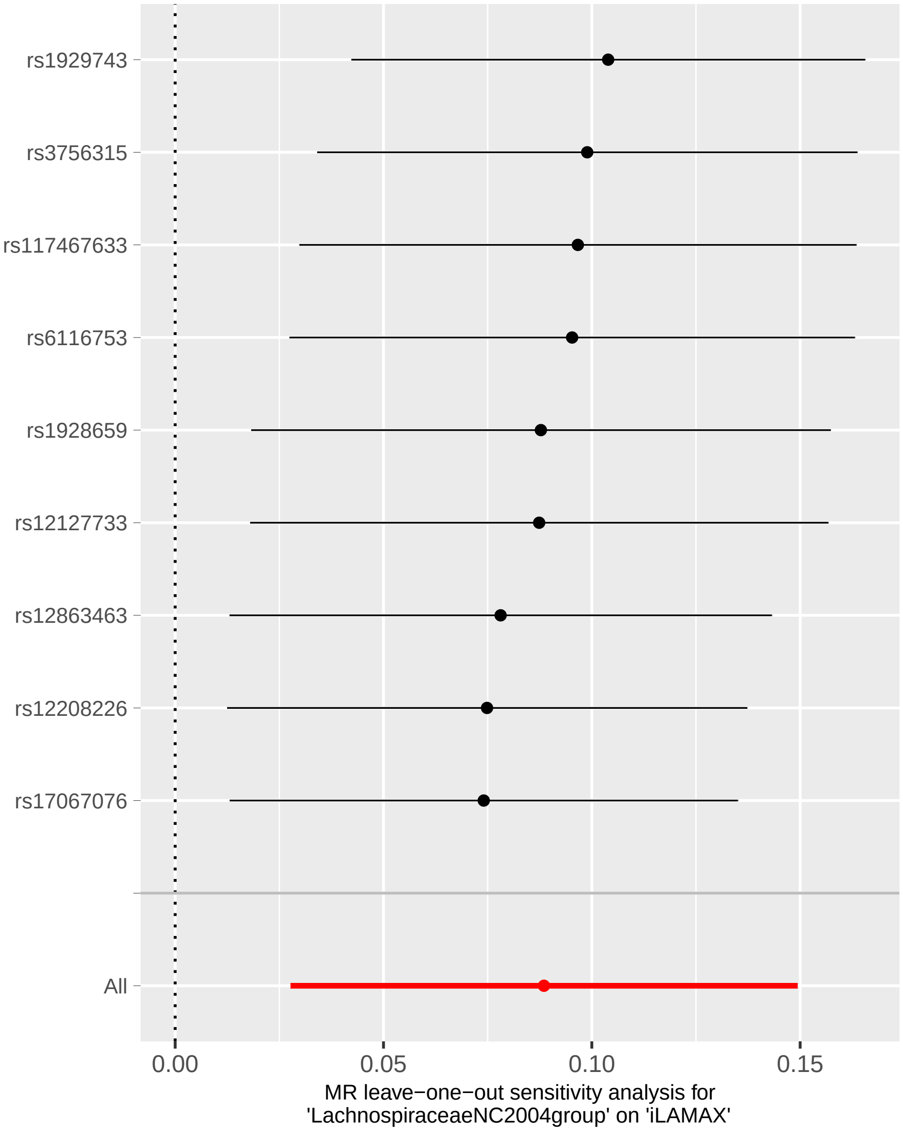
rs1929743
rs3756315
rs117467633
rs6116753
rs1928659
rs12127733
rs12863463
rs12208226
rs17067076
All
0.00
0.05
0.10
0.15
MR leave−one−out sensitivity analysis for
'LachnospiraceaeNC2004group' on 'iLAMAX'

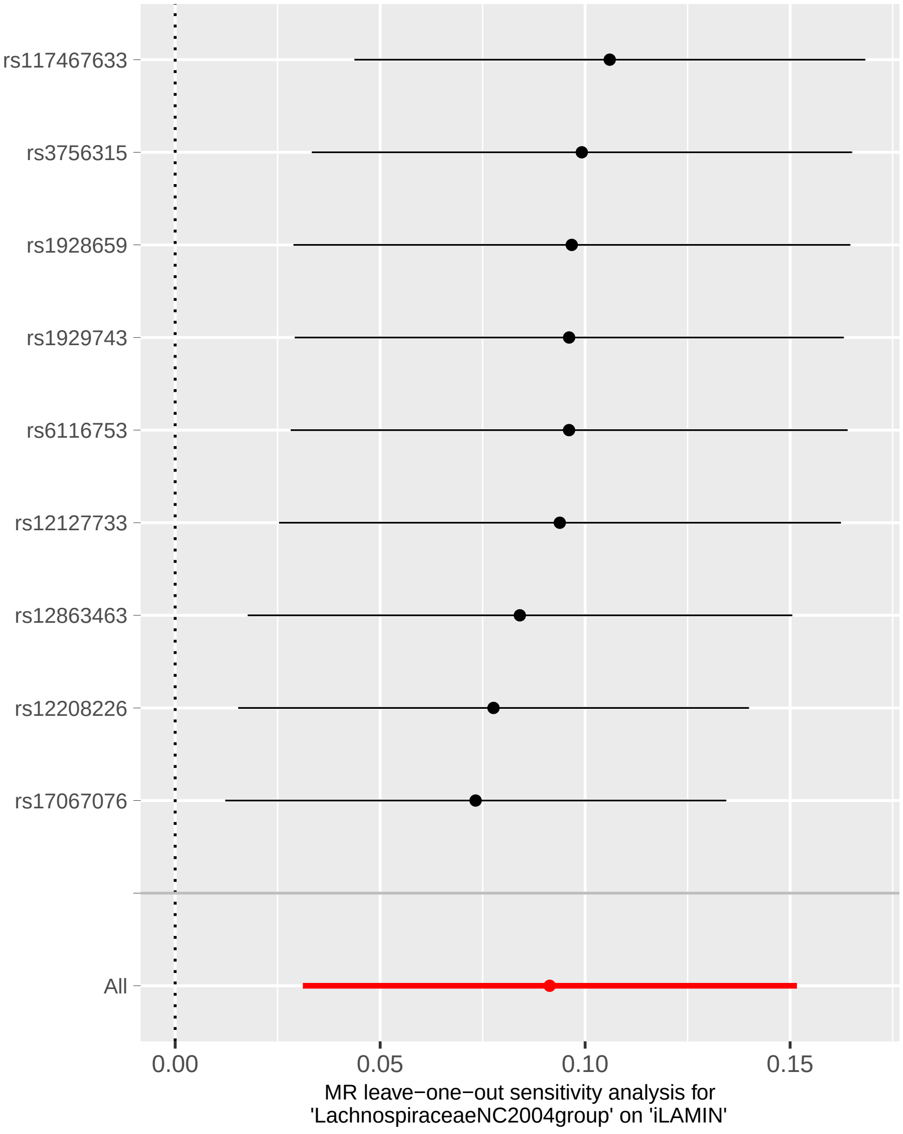
rs117467633
rs3756315
rs1928659
rs1929743
rs6116753
rs12127733
rs12863463
rs12208226
rs17067076
All
0.00
0.05
0.10
0.15
MR leave−one−out sensitivity analysis for
'LachnospiraceaeNC2004group' on 'iLAMIN'

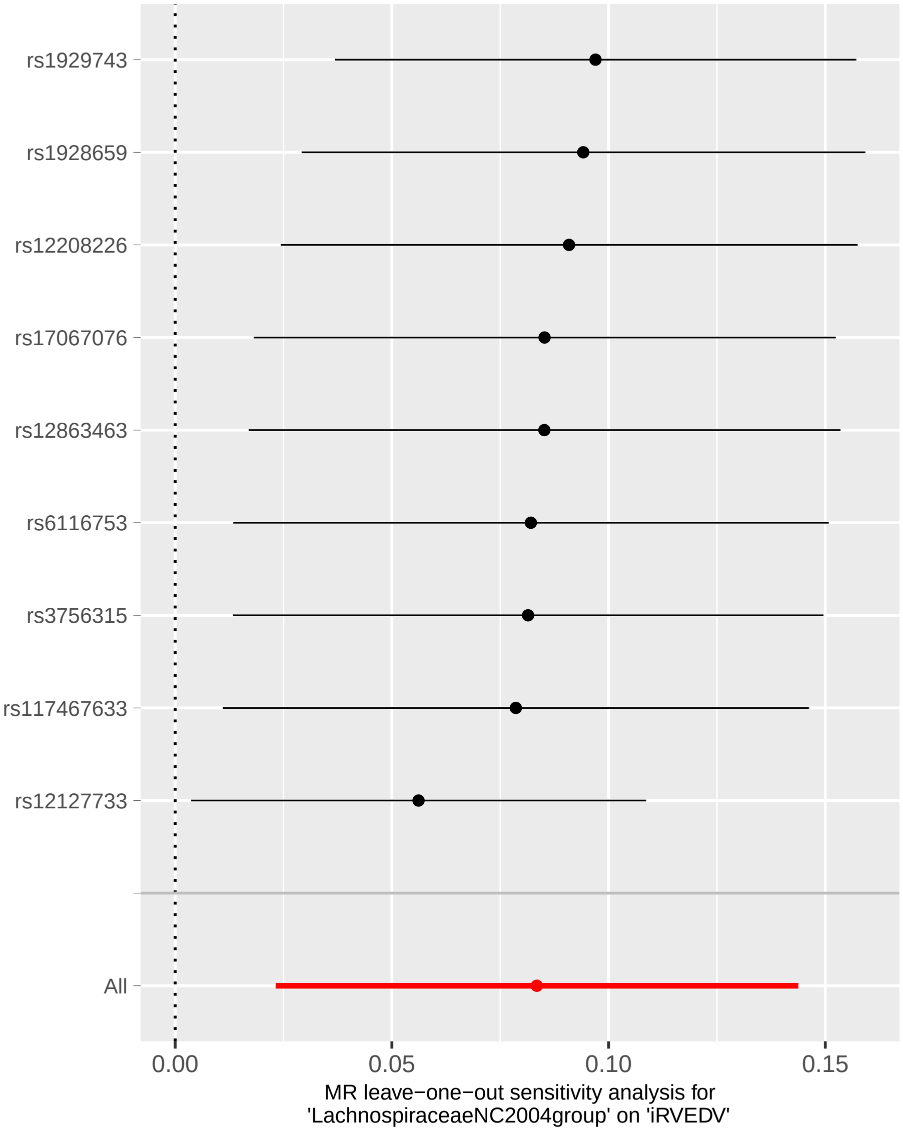
rs1929743
rs1928659
rs12208226
rs17067076
rs12863463
rs6116753
rs3756315
rs117467633
rs12127733
All
0.00
0.05
0.10
0.15
MR leave−one−out sensitivity analysis for
'LachnospiraceaeNC2004group' on 'iRVEDV'

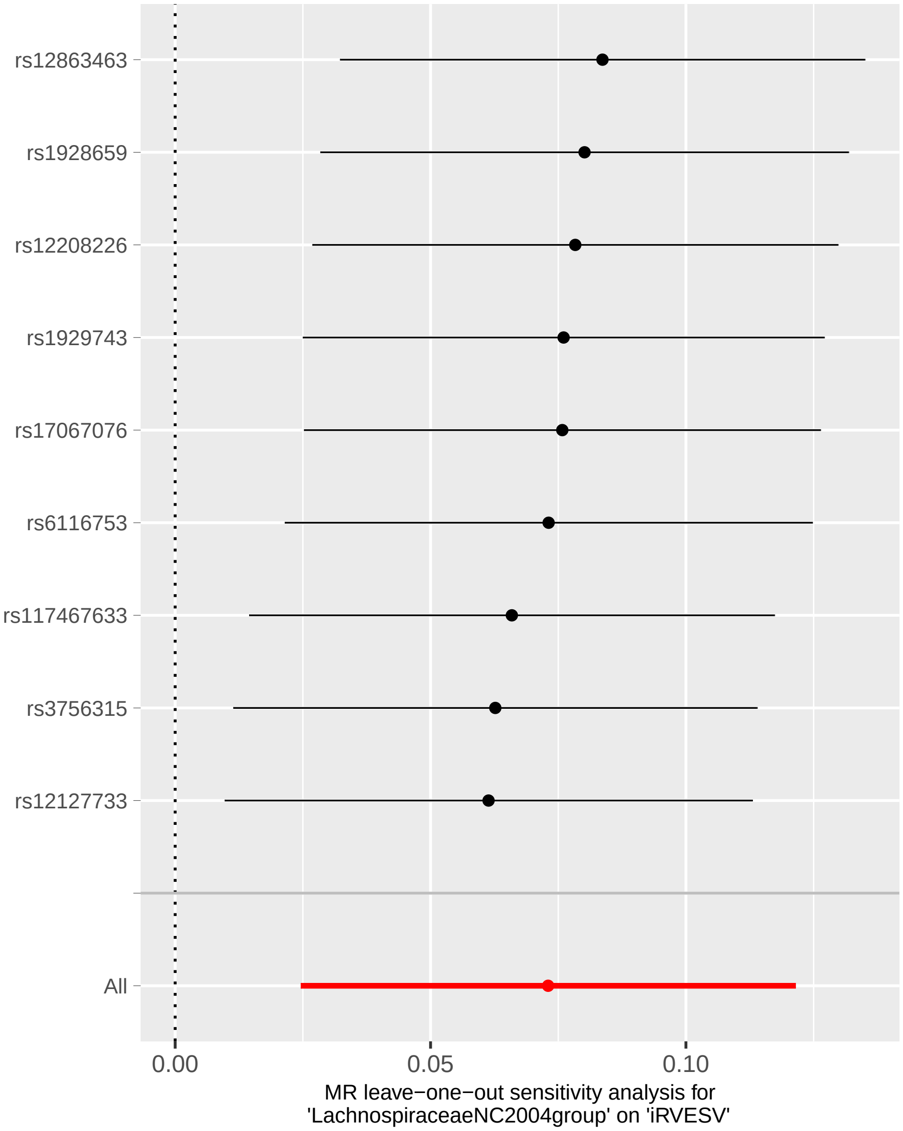

rs12863463
rs1928659
rs12208226
rs1929743
rs17067076
rs6116753
rs117467633
rs3756315
rs12127733
All
0.00
0.05
0.10
MR leave−one−out sensitivity analysis for
'LachnospiraceaeNC2004group' on 'iRVESV'

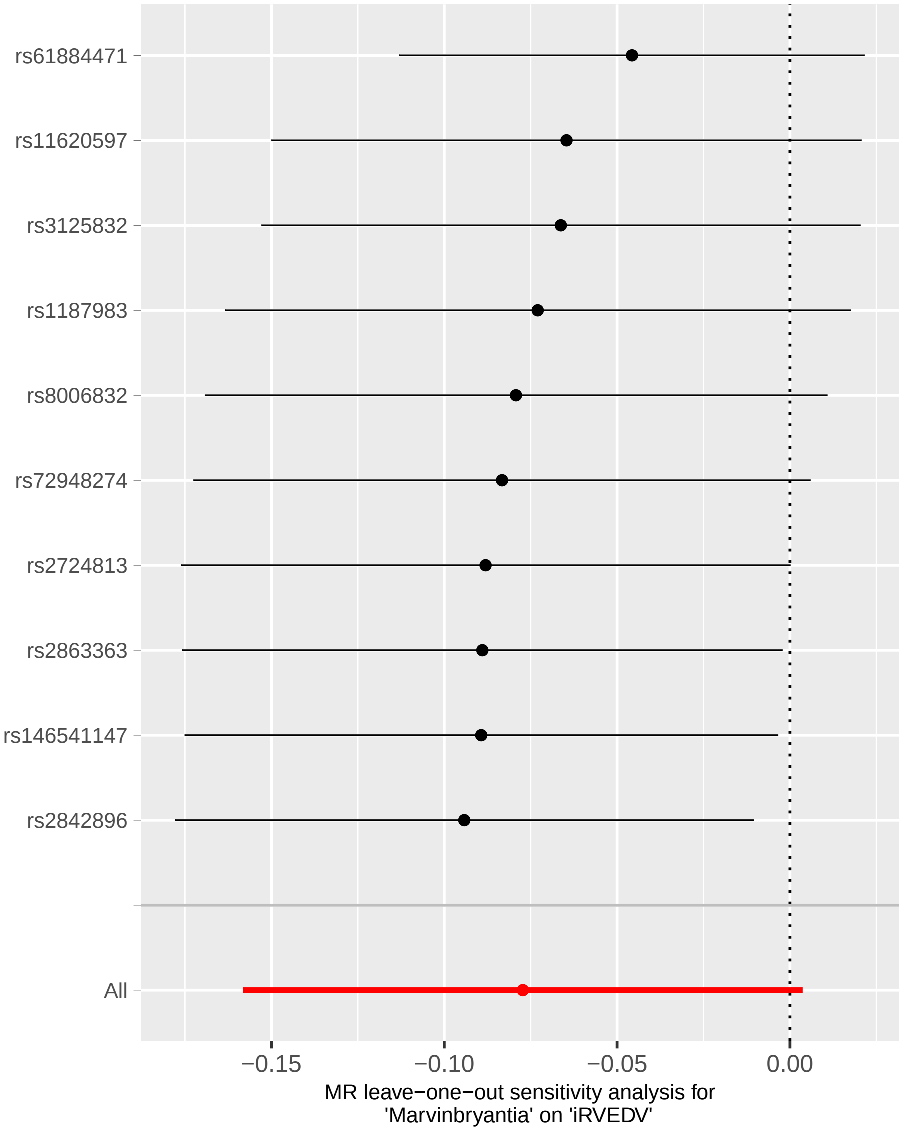
rs61884471
rs11620597
rs3125832
rs1187983
rs8006832
rs72948274
rs2724813
rs2863363
rs146541147
rs2842896
All
−0.15
−0.10
−0.05
0.00
MR leave−one−out sensitivity analysis for
'Marvinbryantia' on 'iRVEDV'

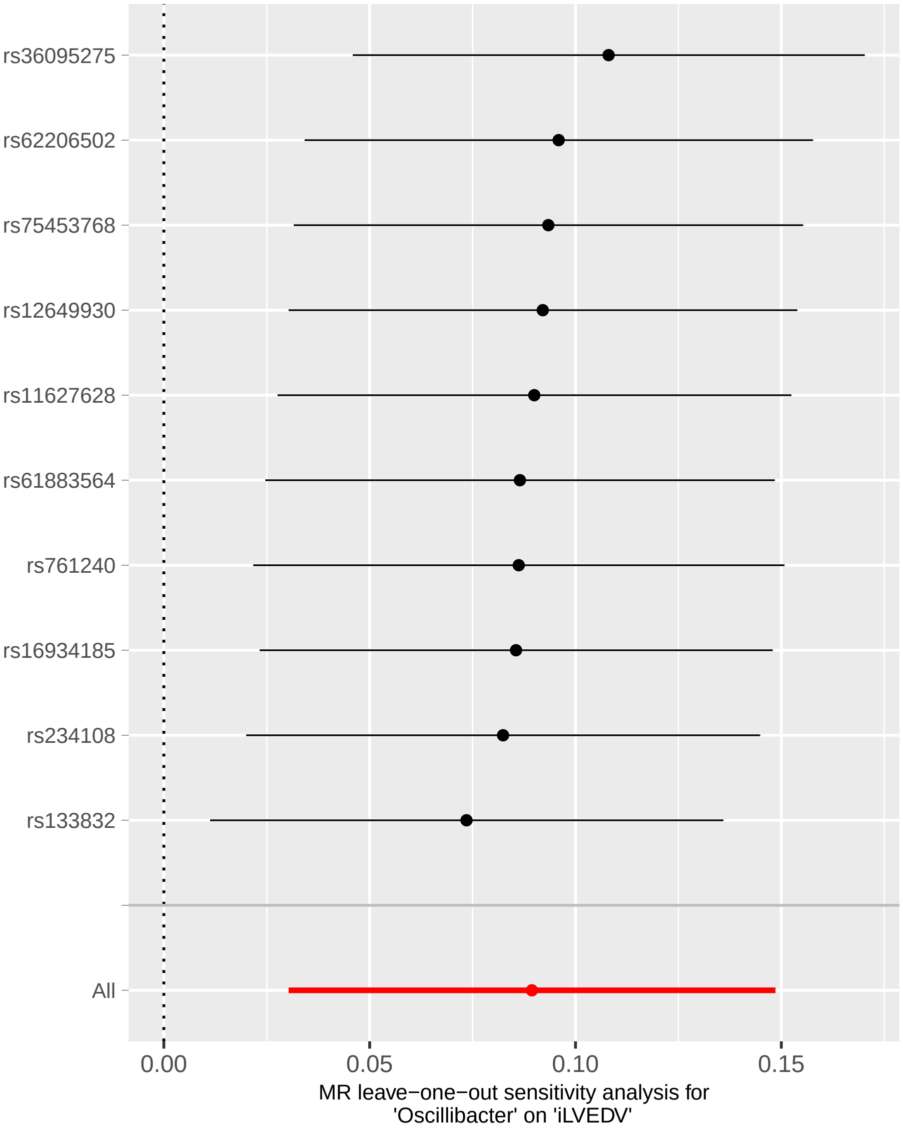
rs36095275
rs62206502
rs75453768
rs12649930
rs11627628
rs61883564
rs761240
rs16934185
rs234108
rs133832
All
0.00
0.05
0.10
0.15
MR leave−one−out sensitivity analysis for
'Oscillibacter' on 'iLVEDV'

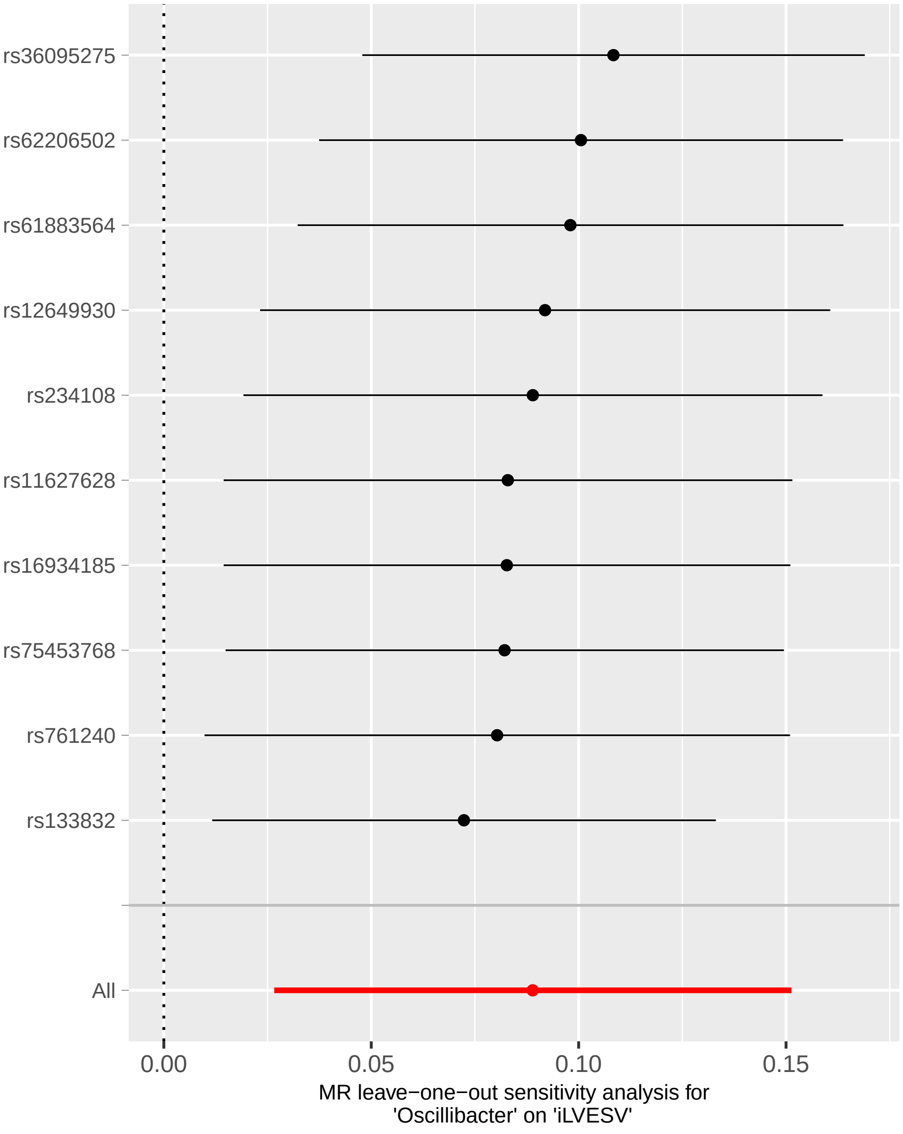
rs36095275
rs62206502
rs61883564
rs12649930
rs234108
rs11627628
rs16934185
rs75453768
rs761240
rs133832
All
0.00
0.05
0.10
0.15
MR leave−one−out sensitivity analysis for
'Oscillibacter' on 'iLVESV'

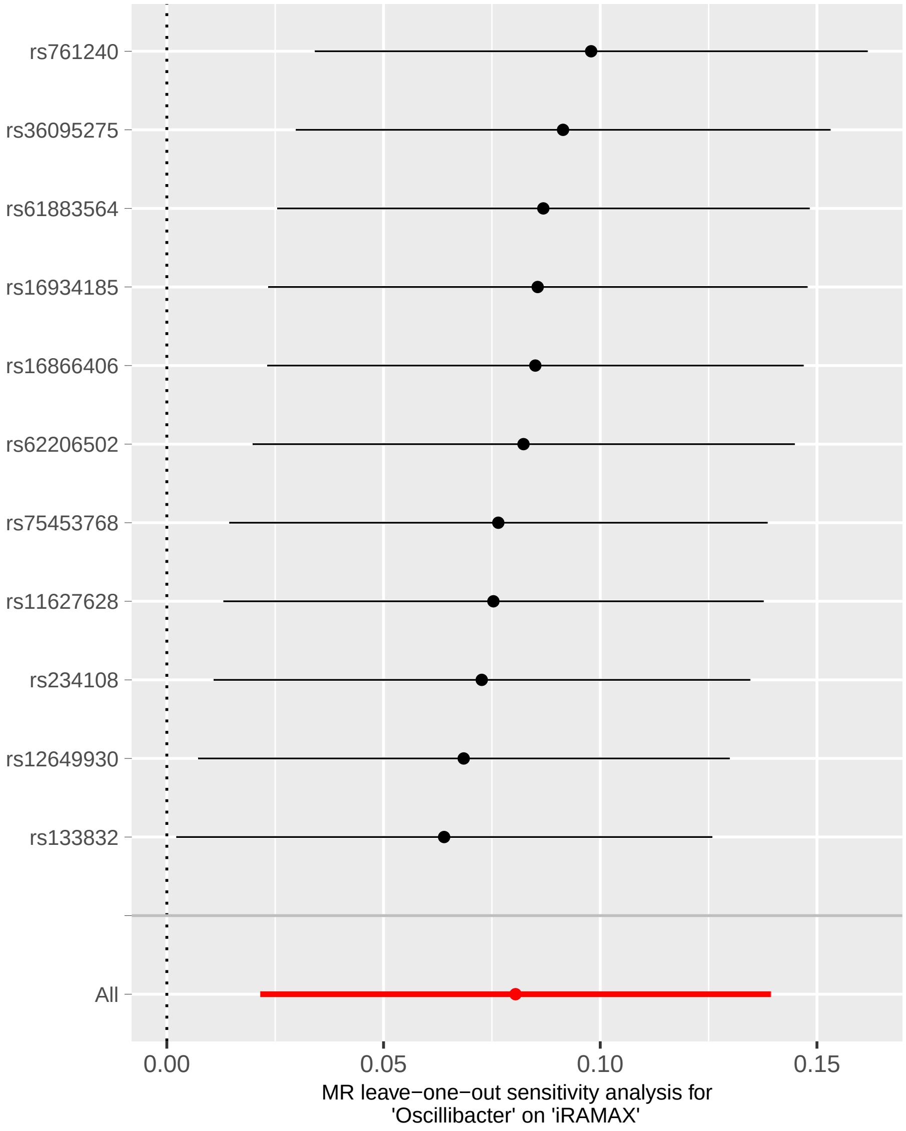
rs761240
rs36095275
rs61883564
rs16934185
rs16866406
rs62206502
rs75453768
rs11627628
rs234108
rs12649930
rs133832
All
0.00
0.05
0.10
0.15
MR leave−one−out sensitivity analysis for
'Oscillibacter' on 'iRAMAX'

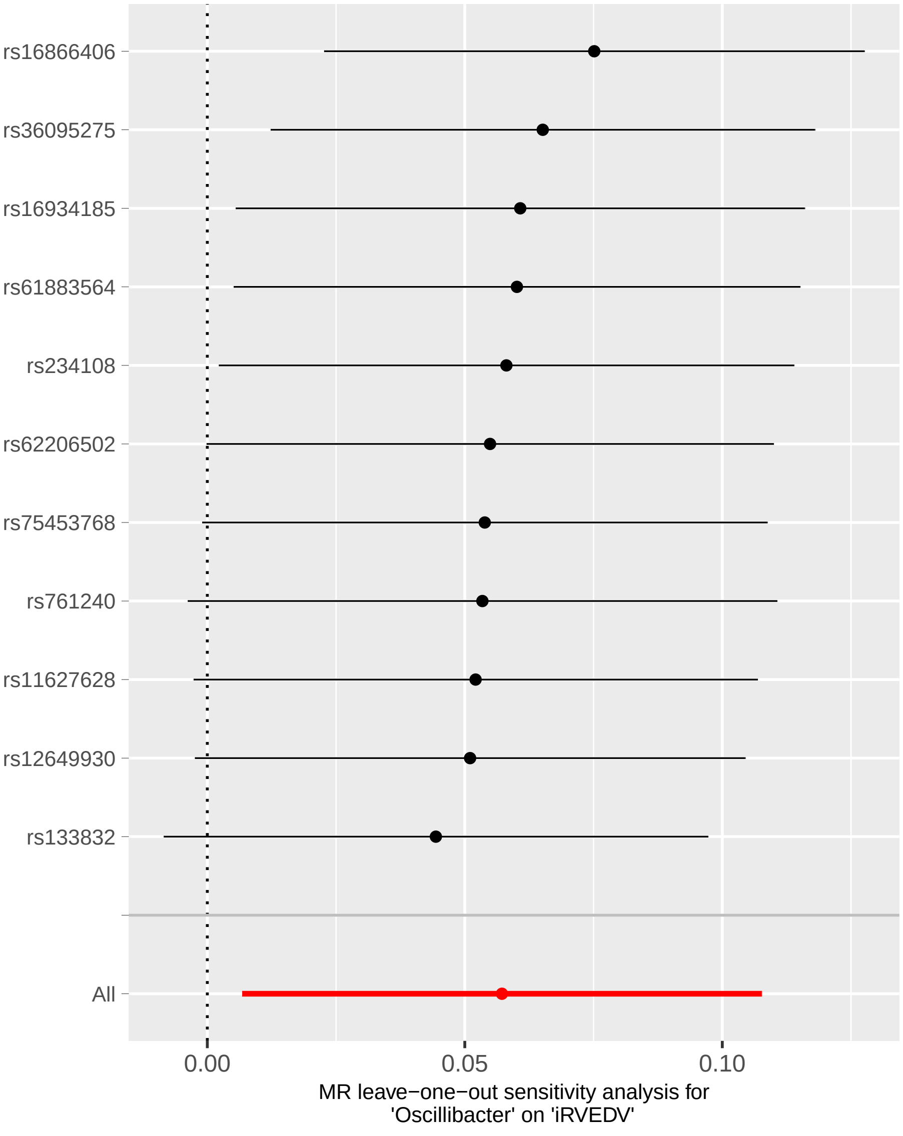
rs16866406
rs36095275
rs16934185
rs61883564
rs234108
rs62206502
rs75453768
rs761240
rs11627628
rs12649930
rs133832
All
0.00
0.05
0.10
MR leave−one−out sensitivity analysis for
'Oscillibacter' on 'iRVEDV'

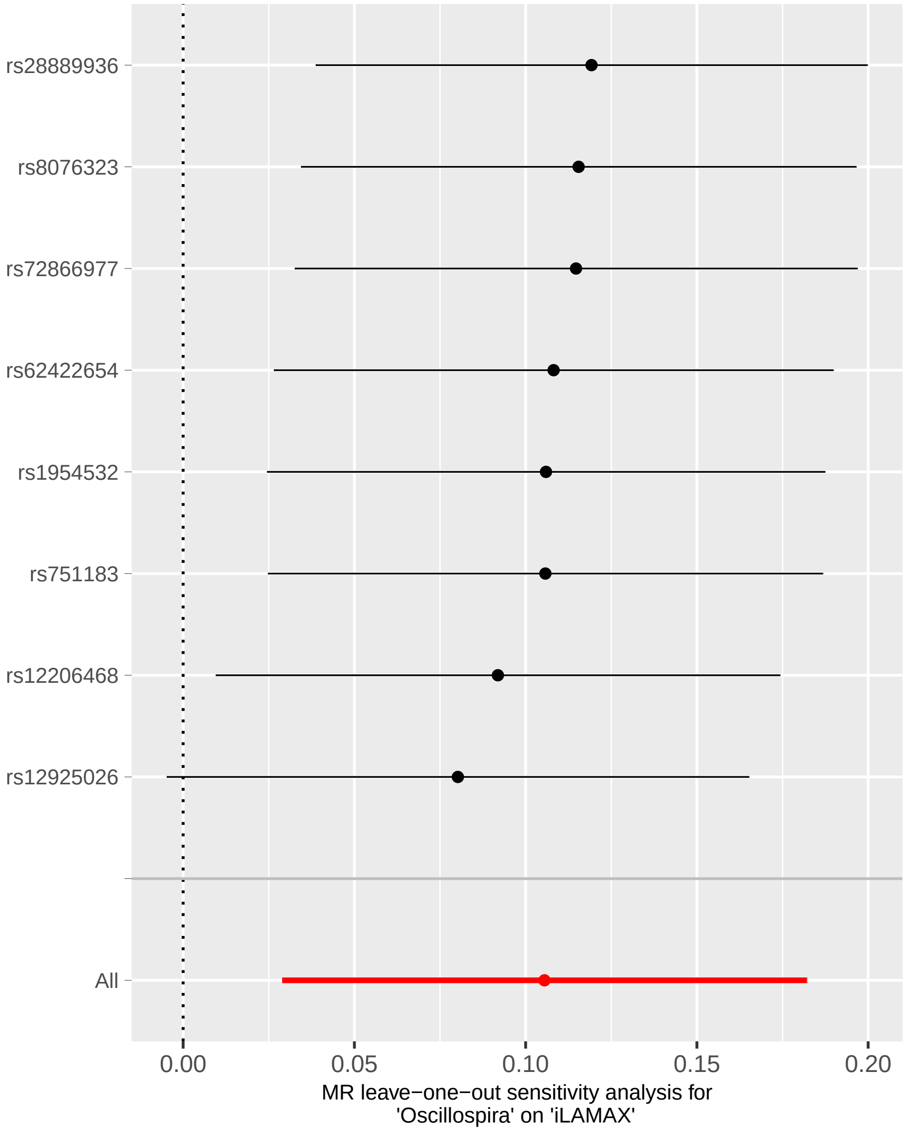
rs28889936
rs8076323
rs72866977
rs62422654
rs1954532
rs751183
rs12206468
rs12925026
All
0.00
0.05
0.10
0.15
0.20
MR leave−one−out sensitivity analysis for
'Oscillospira' on 'iLAMAX'

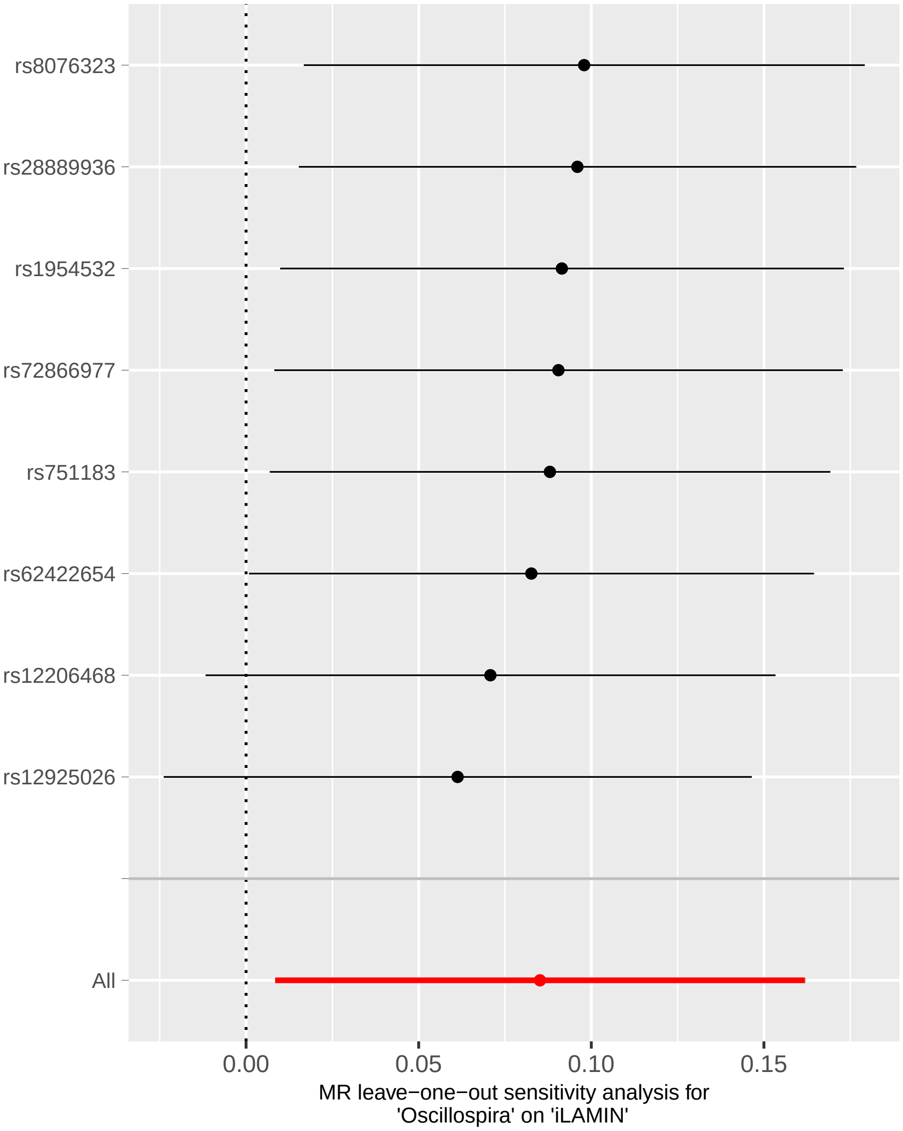

rs8076323
rs28889936
rs1954532
rs72866977
rs751183
rs62422654
rs12206468
rs12925026
All
0.00
0.05
0.10
0.15
MR leave−one−out sensitivity analysis for
'Oscillospira' on 'iLAMIN'

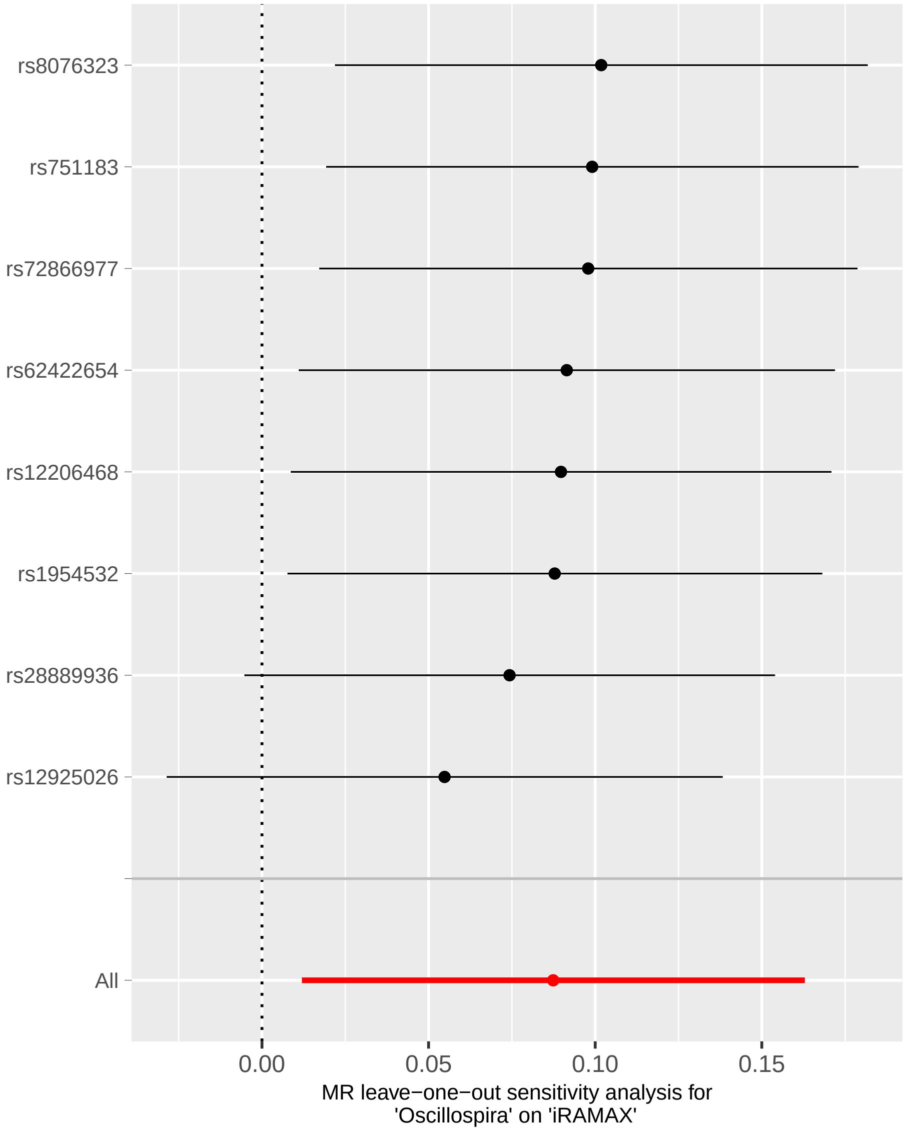

rs8076323
rs751183
rs72866977
rs62422654
rs12206468
rs1954532
rs28889936
rs12925026
All
0.00
0.05
0.10
0.15
MR leave−one−out sensitivity analysis for
'Oscillospira' on 'iRAMAX'

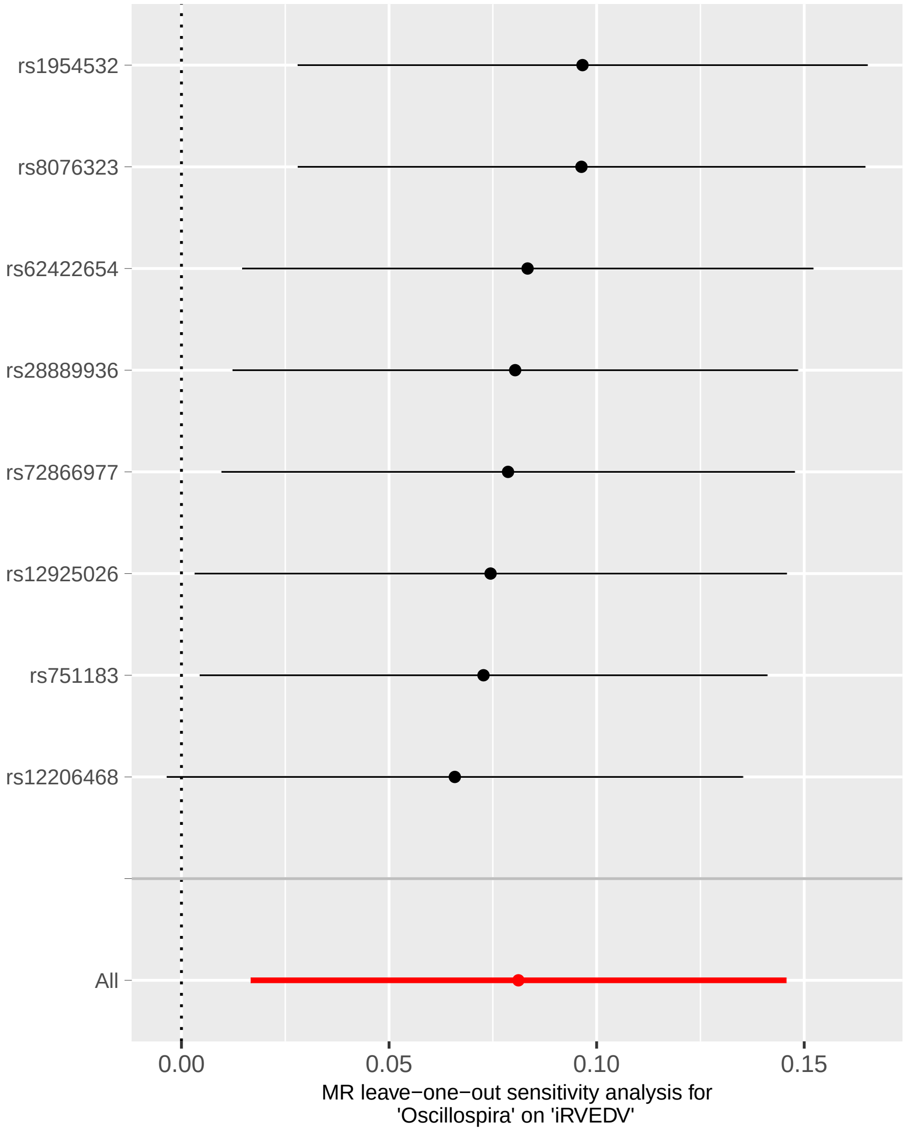
rs1954532
rs8076323
rs62422654
rs28889936
rs72866977
rs12925026
rs751183
rs12206468
All
0.00
0.05
0.10
0.15
MR leave−one−out sensitivity analysis for
'Oscillospira' on 'iRVEDV'

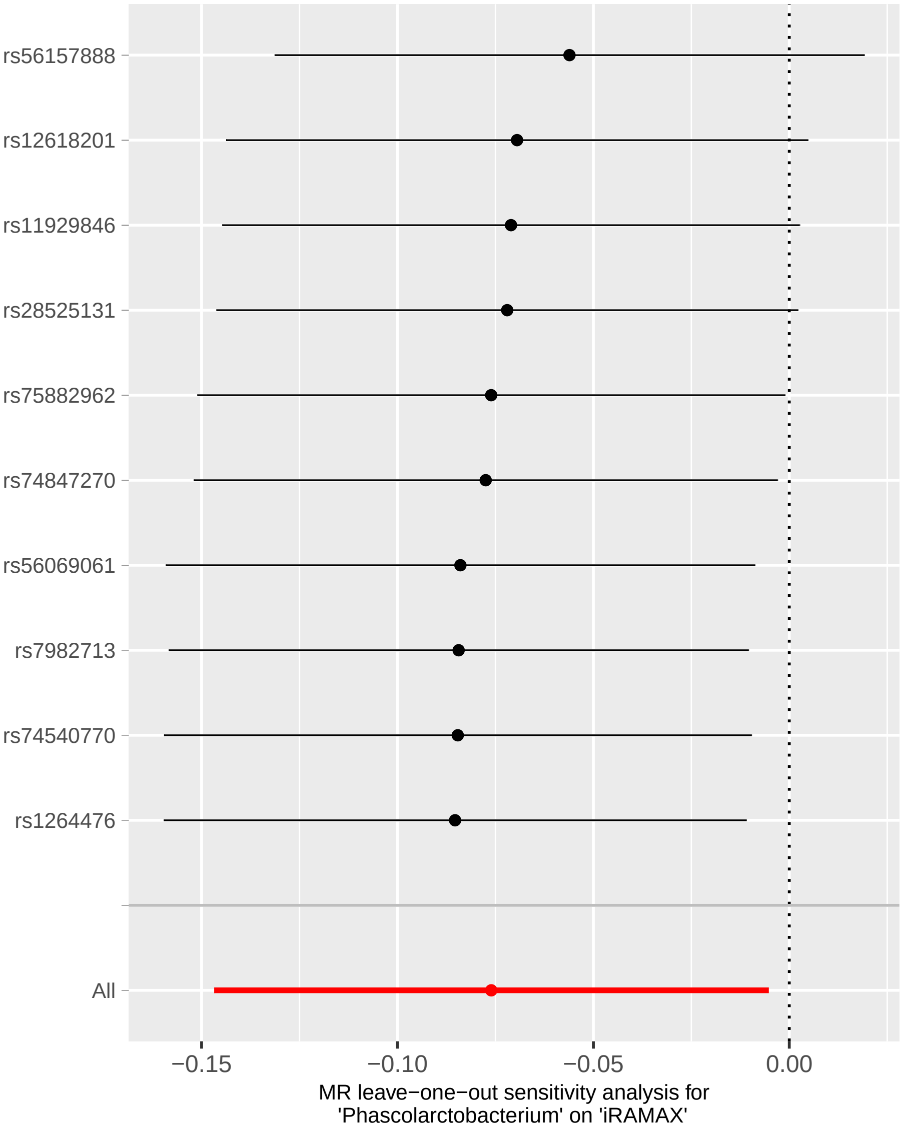

rs56157888
rs12618201
rs11929846
rs28525131
rs75882962
rs74847270
rs56069061
rs7982713
rs74540770
rs1264476
All
−0.15
−0.10
−0.05
0.00
MR leave−one−out sensitivity analysis for
'Phascolarctobacterium' on 'iRAMAX'

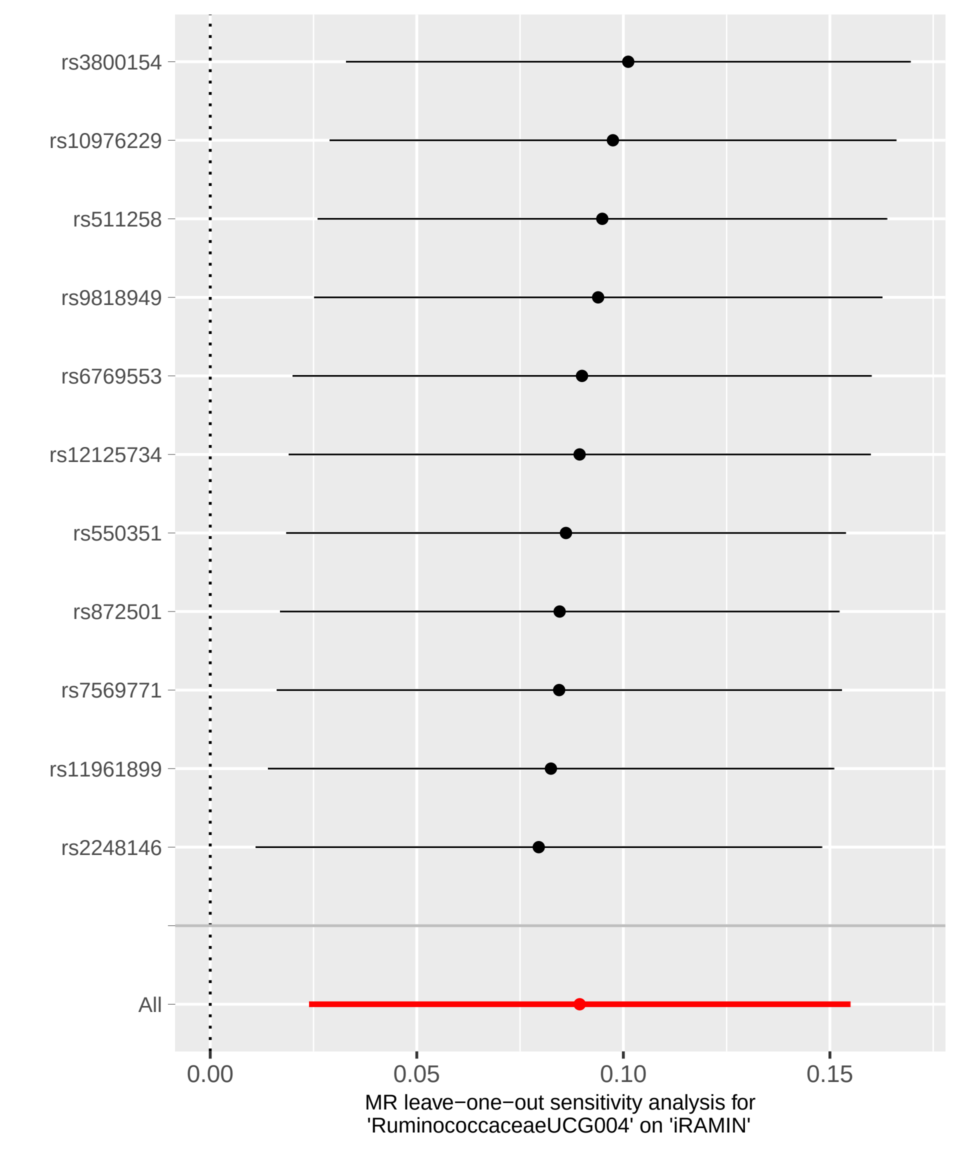

rs3800154
rs10976229
rs511258
rs9818949
rs6769553
rs12125734
rs550351
rs872501
rs7569771
rs11961899
rs2248146
All
0.00
0.05
0.10
0.15
MR leave−one−out sensitivity analysis for
'RuminococcaceaeUCG004' on 'iRAMIN'

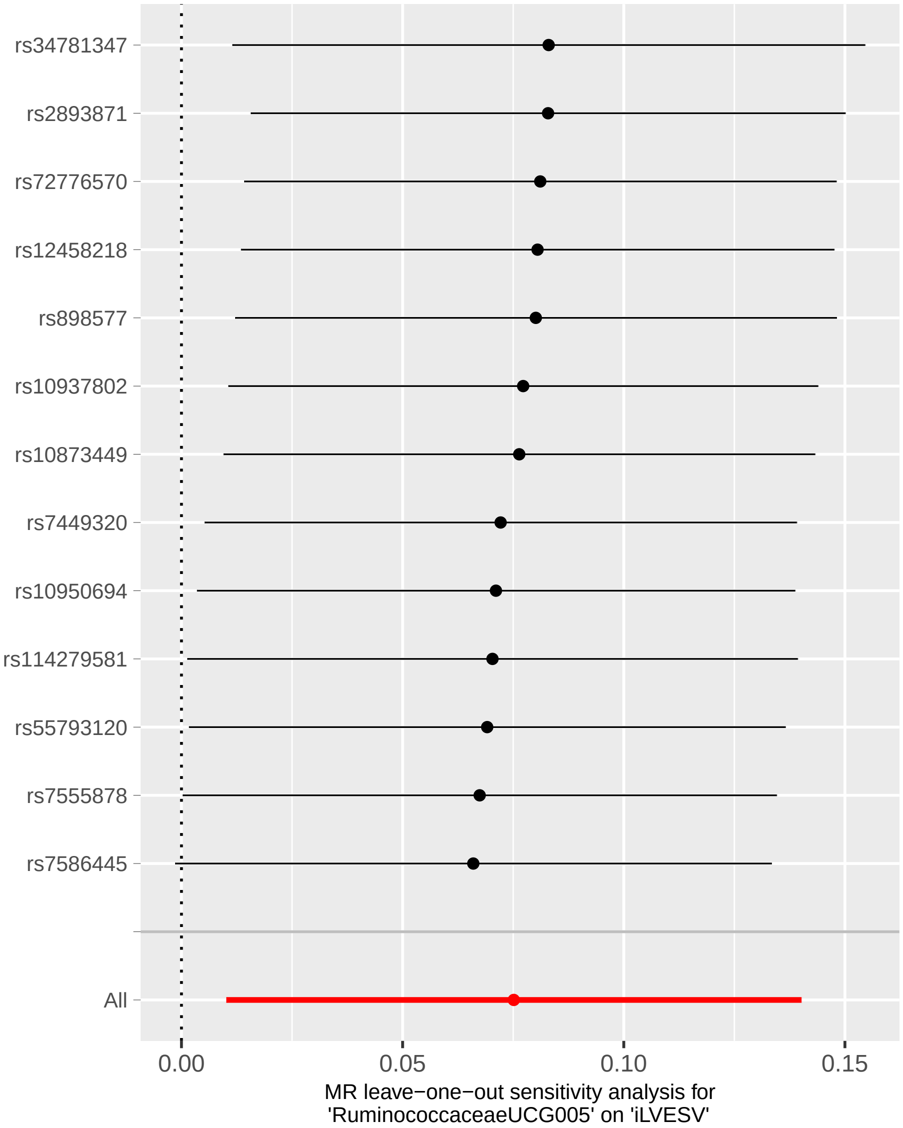

rs34781347
rs2893871
rs72776570
rs12458218
rs898577
rs10937802
rs10873449
rs7449320
rs10950694
rs114279581
rs55793120
rs7555878
rs7586445
All
0.00
0.05
0.10
0.15
MR leave−one−out sensitivity analysis for
'RuminococcaceaeUCG005' on 'iLVESV'

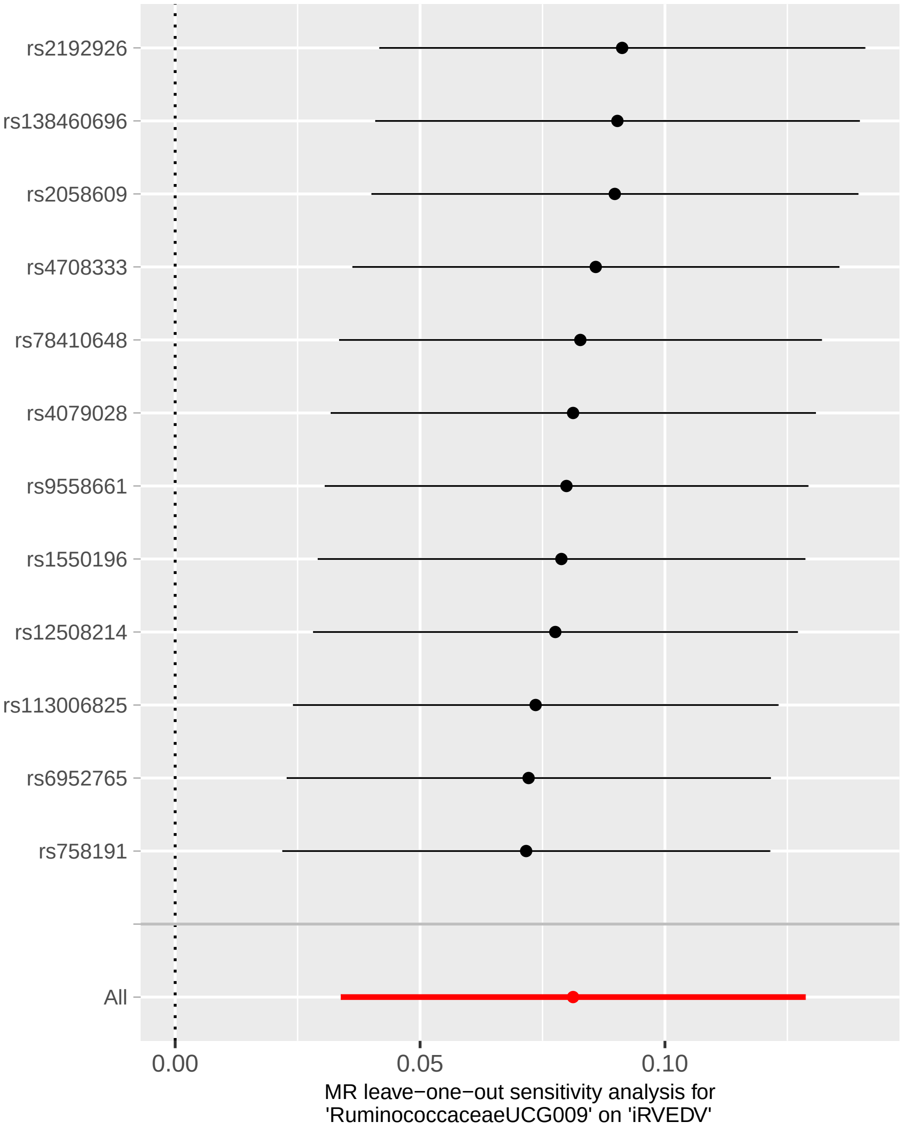
rs2192926
rs138460696
rs2058609
rs4708333
rs78410648
rs4079028
rs9558661
rs1550196
rs12508214
rs113006825
rs6952765
rs758191
All
0.00
0.05
0.10
MR leave−one−out sensitivity analysis for
'RuminococcaceaeUCG009' on 'iRVEDV'

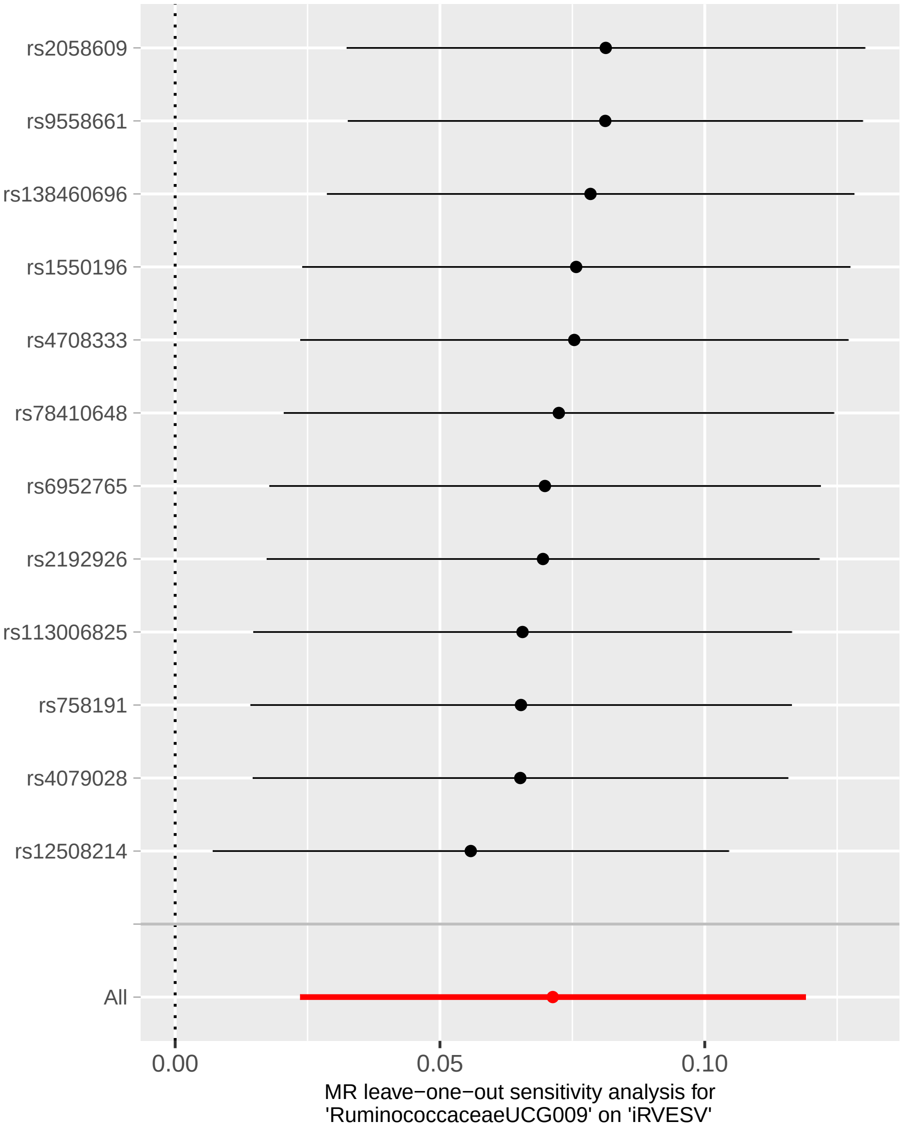
rs2058609
rs9558661
rs138460696
rs1550196
rs4708333
rs78410648
rs6952765
rs2192926
rs113006825
rs758191
rs4079028
rs12508214
All
0.00
0.05
0.10
MR leave−one−out sensitivity analysis for
'RuminococcaceaeUCG009' on 'iRVESV'

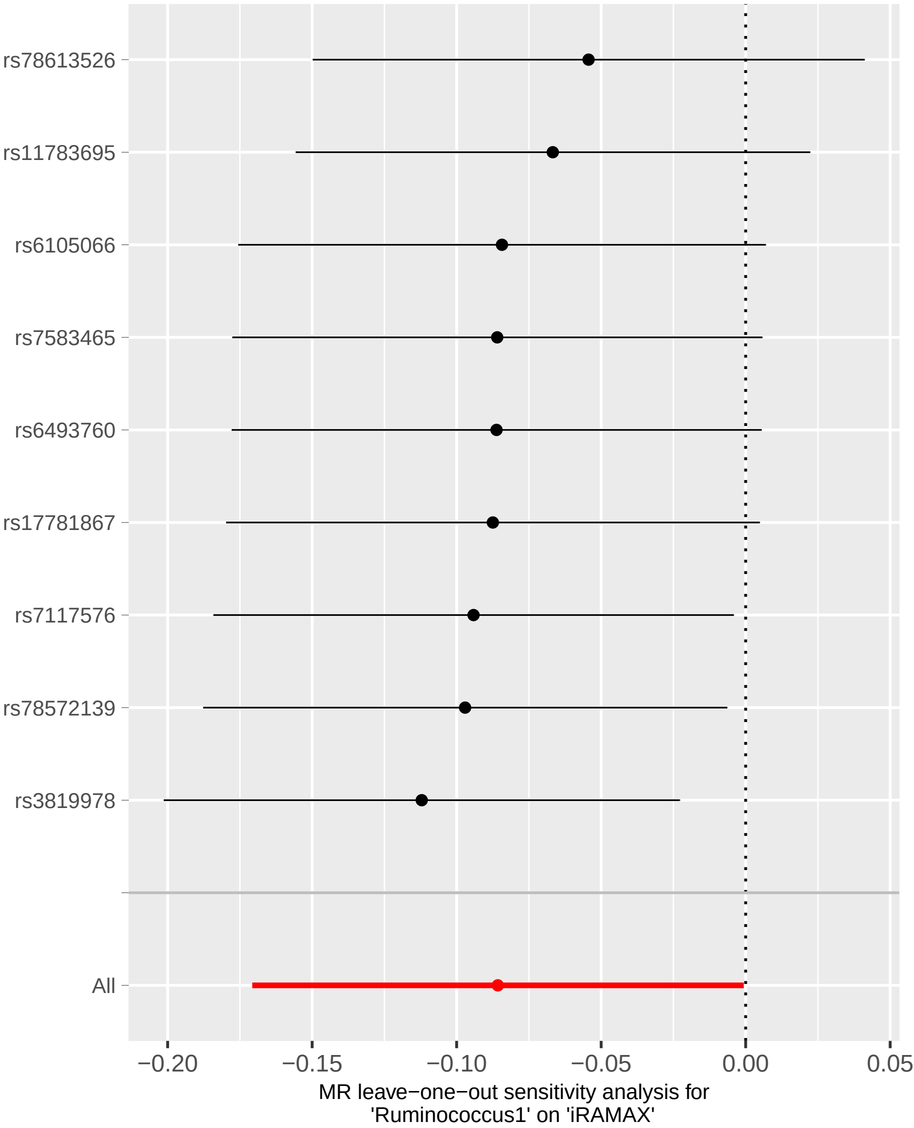
rs78613526
rs11783695
rs6105066
rs7583465
rs6493760
rs17781867
rs7117576
rs78572139
rs3819978
All
−0.20
−0.15
−0.10
−0.05
0.00
0.05
MR leave−one−out sensitivity analysis for
'Ruminococcus1' on 'iRAMAX'

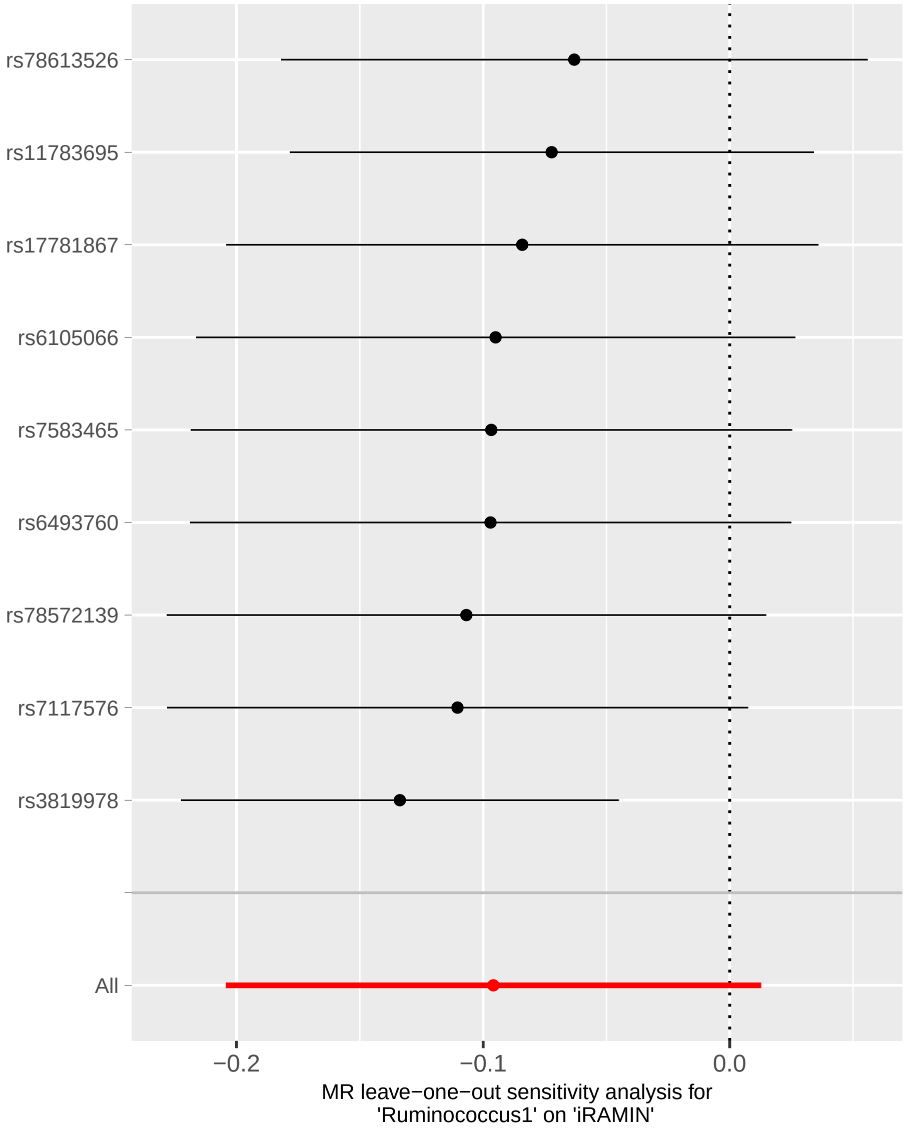
rs78613526
rs11783695
rs17781867
rs6105066
rs7583465
rs6493760
rs78572139
rs7117576
rs3819978
All
−0.2
−0.1
0.0
MR leave−one−out sensitivity analysis for
'Ruminococcus1' on 'iRAMIN'

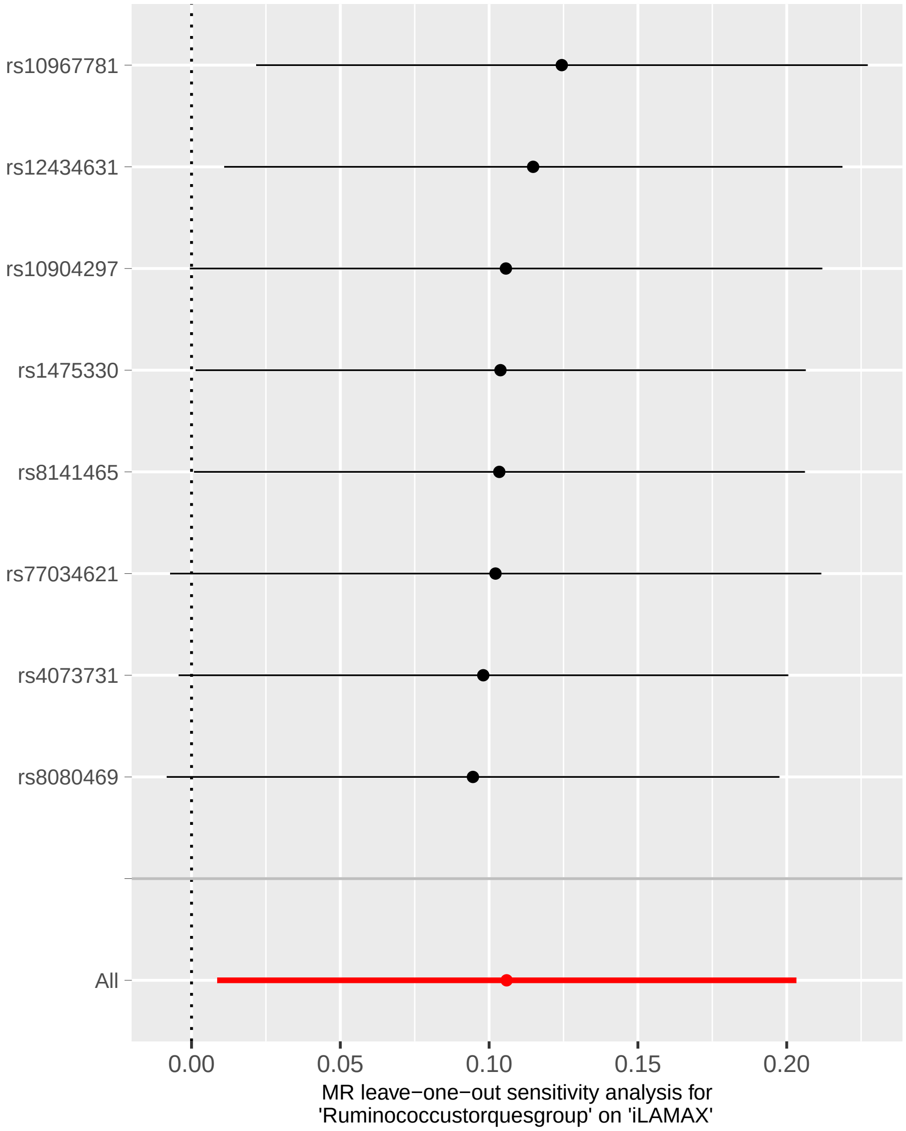
rs10967781
rs12434631
rs10904297
rs1475330
rs8141465
rs77034621
rs4073731
rs8080469
All
0.00
0.05
0.10
0.15
0.20
MR leave−one−out sensitivity analysis for
'Ruminococcustorquesgroup' on 'iLAMAX'

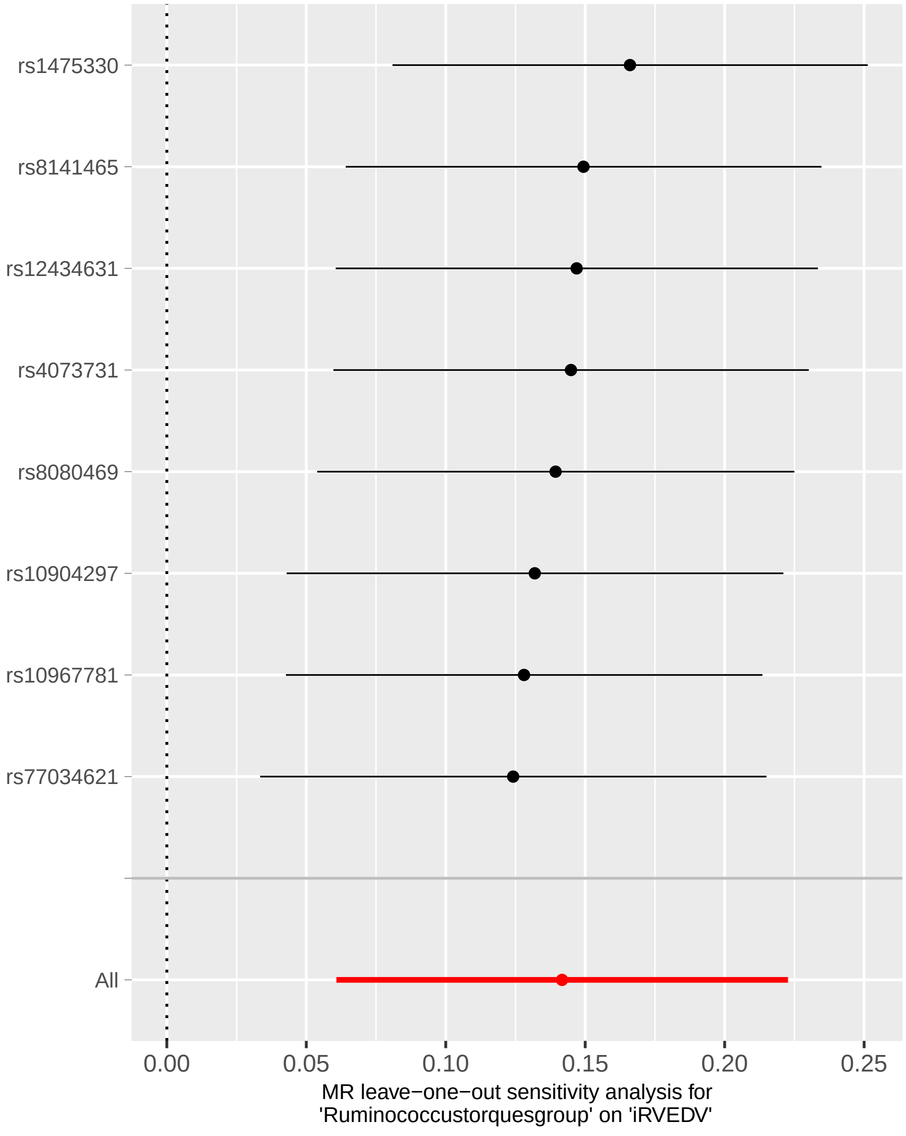
rs1475330
rs8141465
rs12434631
rs4073731
rs8080469
rs10904297
rs10967781
rs77034621
All
0.00
0.05
0.10
0.15
0.20
0.25
MR leave−one−out sensitivity analysis for
'Ruminococcustorquesgroup' on 'iRVEDV'

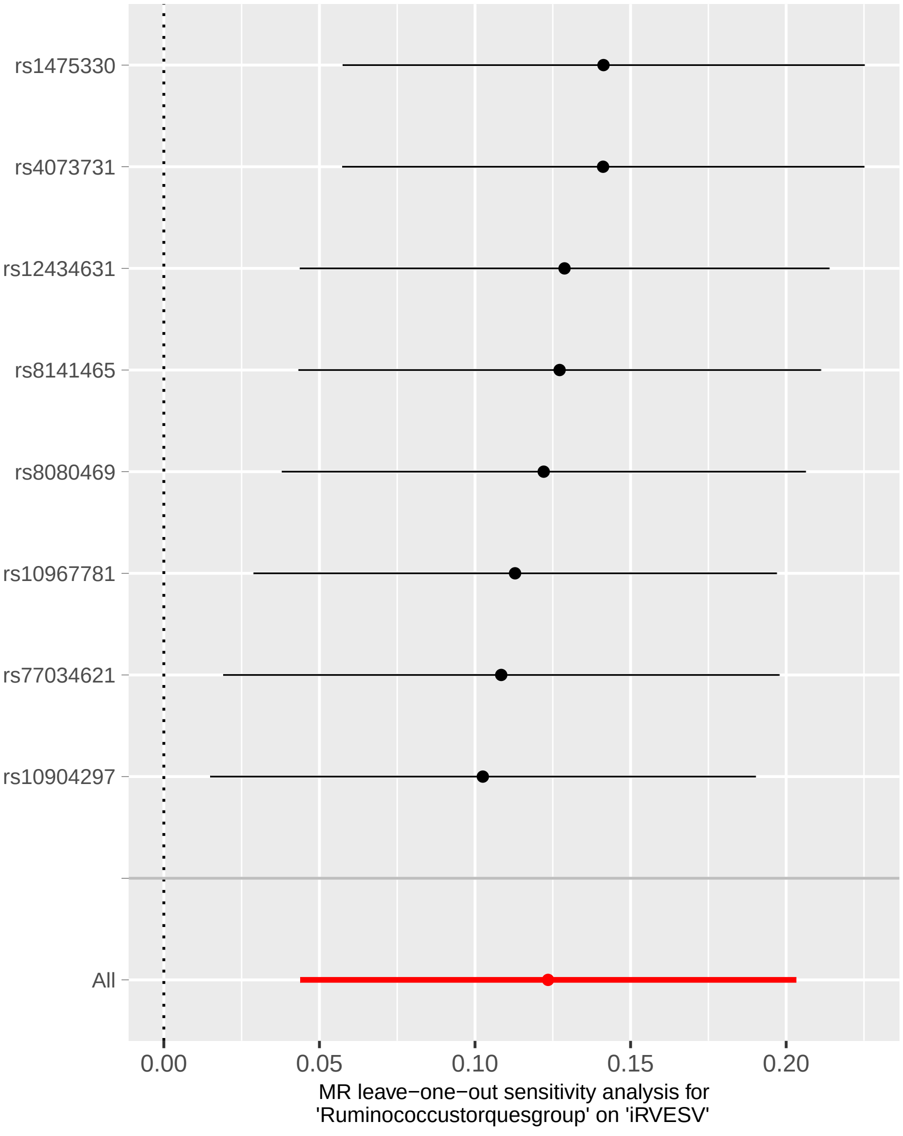

rs1475330
rs4073731
rs12434631
rs8141465
rs8080469
rs10967781
rs77034621
rs10904297
All
0.00
0.05
0.10
0.15
0.20
MR leave−one−out sensitivity analysis for
'Ruminococcustorquesgroup' on 'iRVESV'

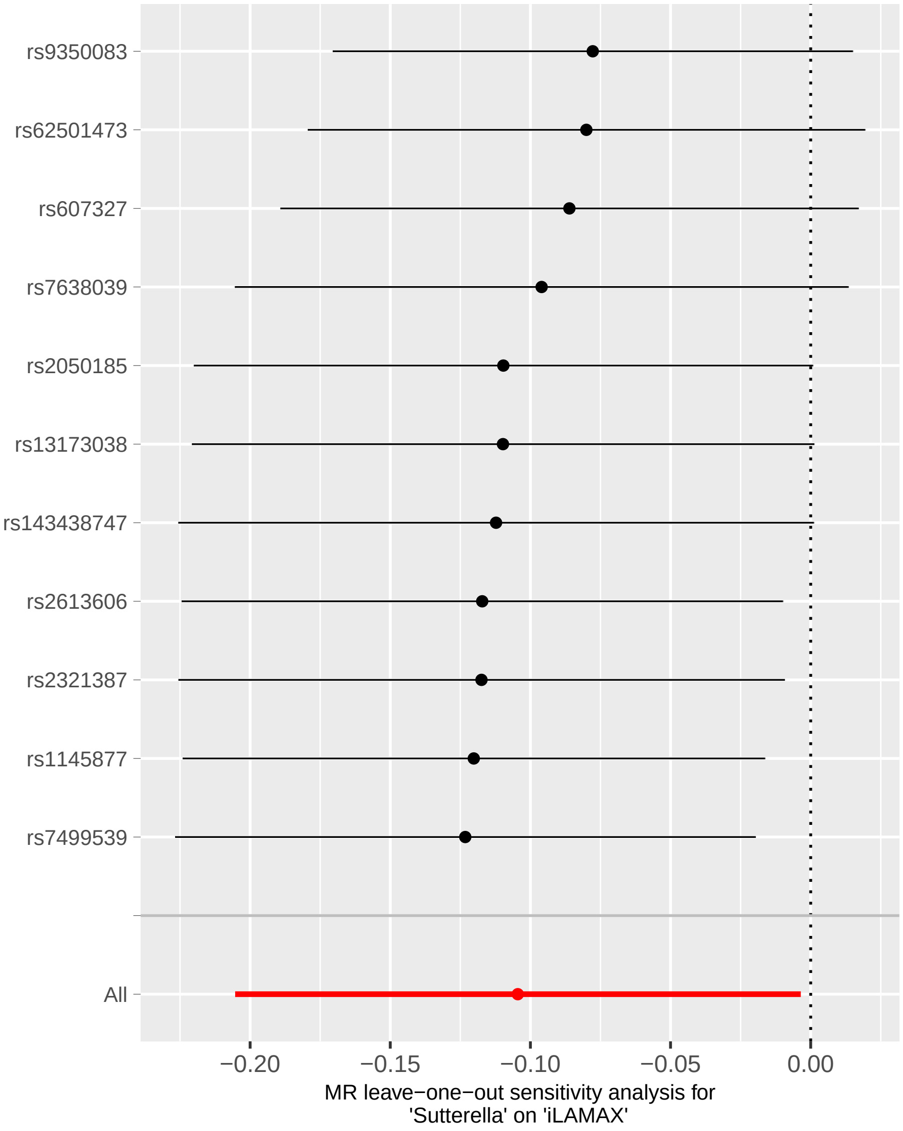
rs9350083
rs62501473
rs607327
rs7638039
rs2050185
rs13173038
rs143438747
rs2613606
rs2321387
rs1145877
rs7499539
All
−0.20
−0.15
−0.10
−0.05
0.00
MR leave−one−out sensitivity analysis for
'Sutterella' on 'iLAMAX'

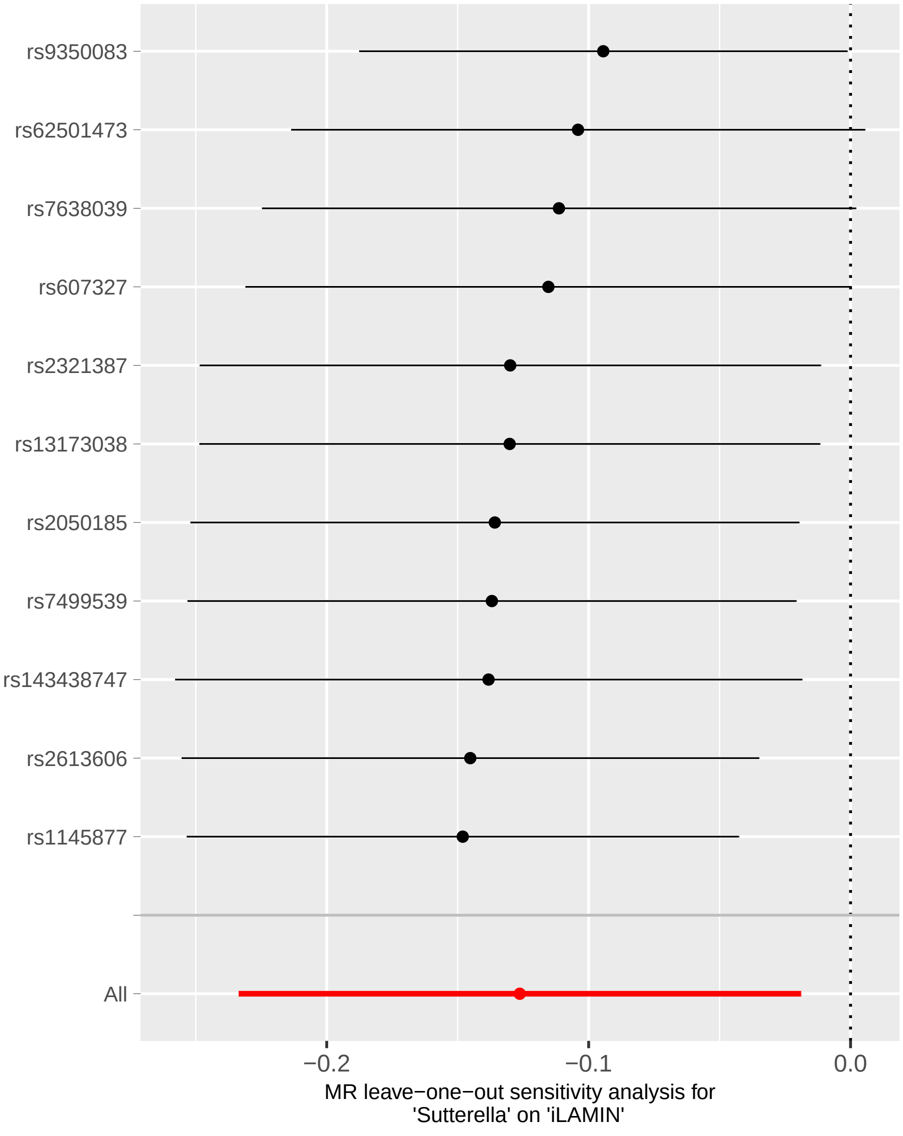
rs9350083
rs62501473
rs7638039
rs607327
rs2321387
rs13173038
rs2050185
rs7499539
rs143438747
rs2613606
rs1145877
All
−0.2
−0.1
0.0
MR leave−one−out sensitivity analysis for
'Sutterella' on 'iLAMIN'

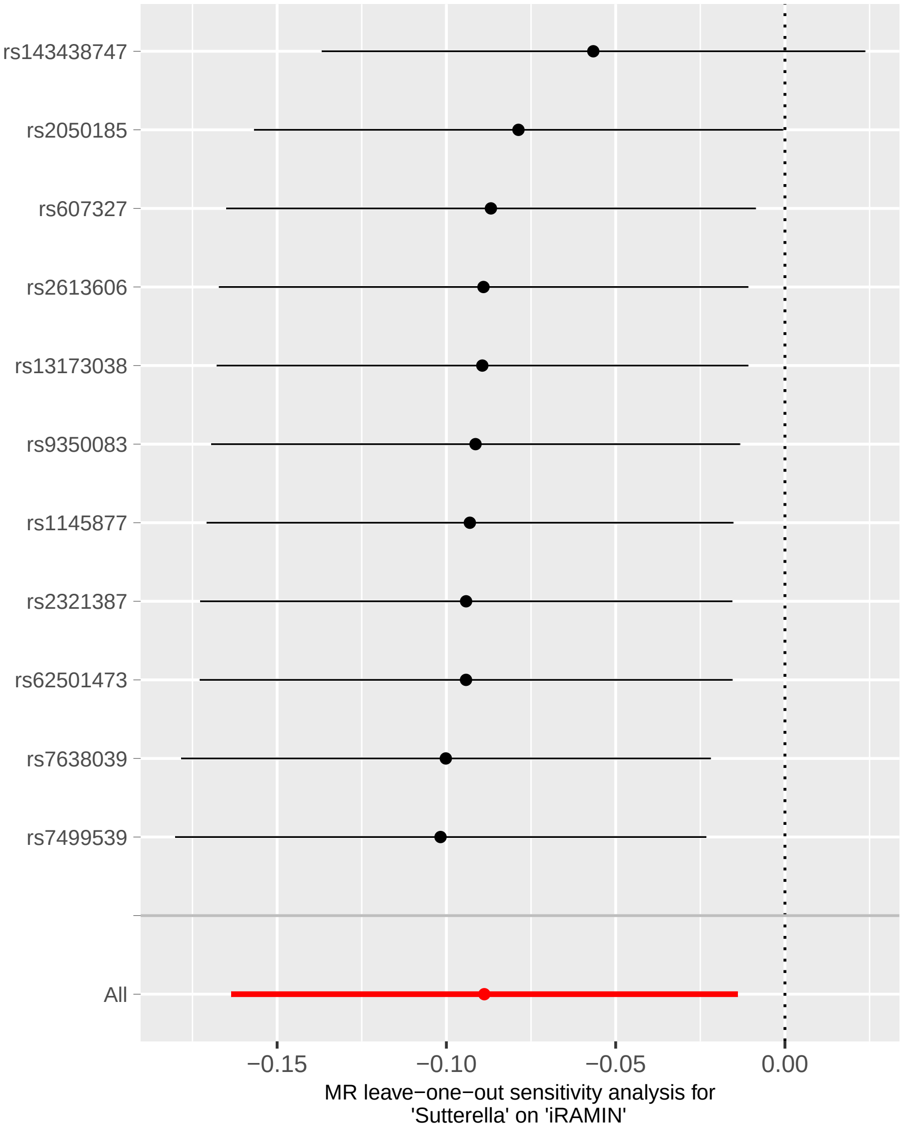

rs143438747
rs2050185
rs607327
rs2613606
rs13173038
rs9350083
rs1145877
rs2321387
rs62501473
rs7638039
rs7499539
All
−0.15
−0.10
−0.05
0.00
MR leave−one−out sensitivity analysis for
'Sutterella' on 'iRAMIN'

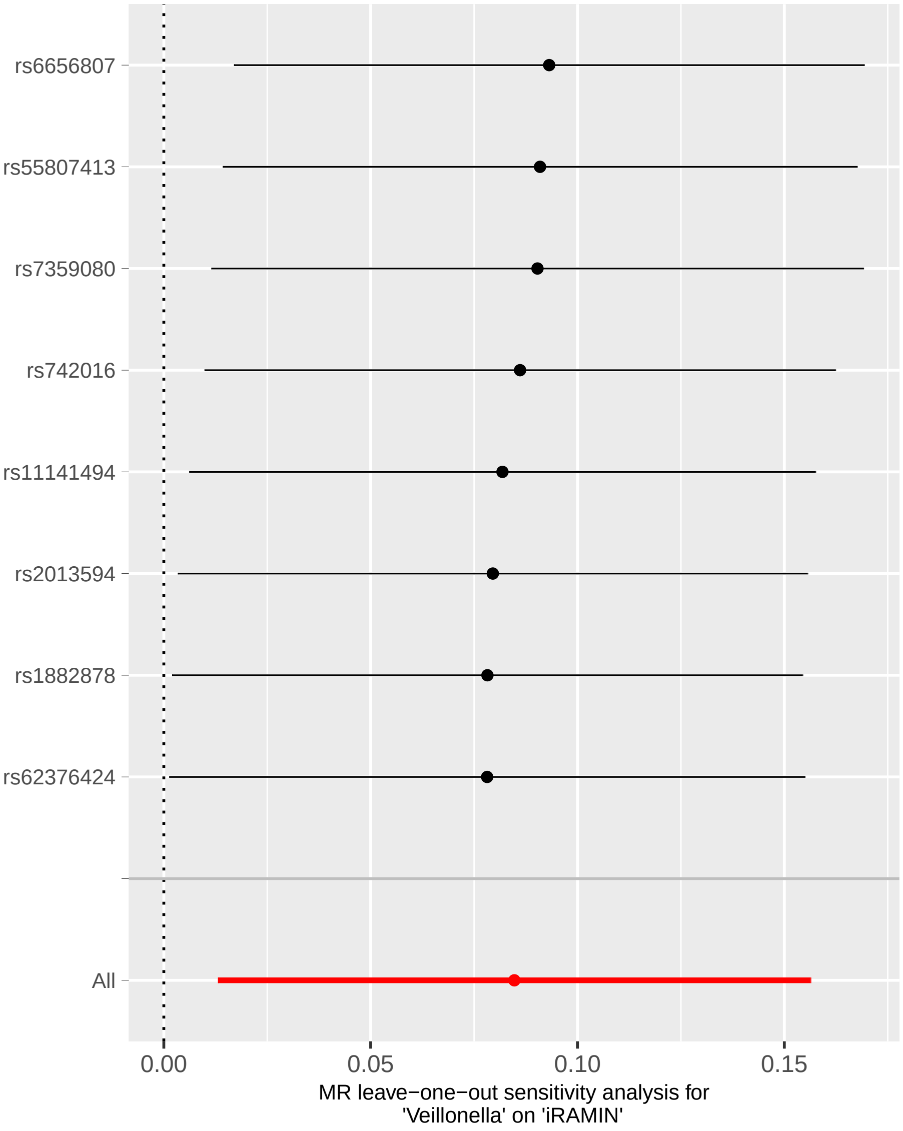

rs6656807
rs55807413
rs7359080
rs742016
rs11141494
rs2013594
rs1882878
rs62376424
All
0.00
0.05
0.10
0.15
MR leave−one−out sensitivity analysis for
'Veillonella' on 'iRAMIN'
