# Supplementary material for: Gut microbiota as a residual risk factor causally influencing cardiac structure and function: Mendelian randomization analysis and biological annotation
Source: Front Microbiol. 2024 Jul 26;15:1410272. doi: 10.3389/fmicb.2024.1410272 (PMC11316272; doi:10.3389/fmicb.2024.1410272)
Supplement: SUPPLEMENTARY FIGURE S2 — Leave-one-out analyses for gut microbiota on cardiac function. [file Data_Sheet_2.PDF]

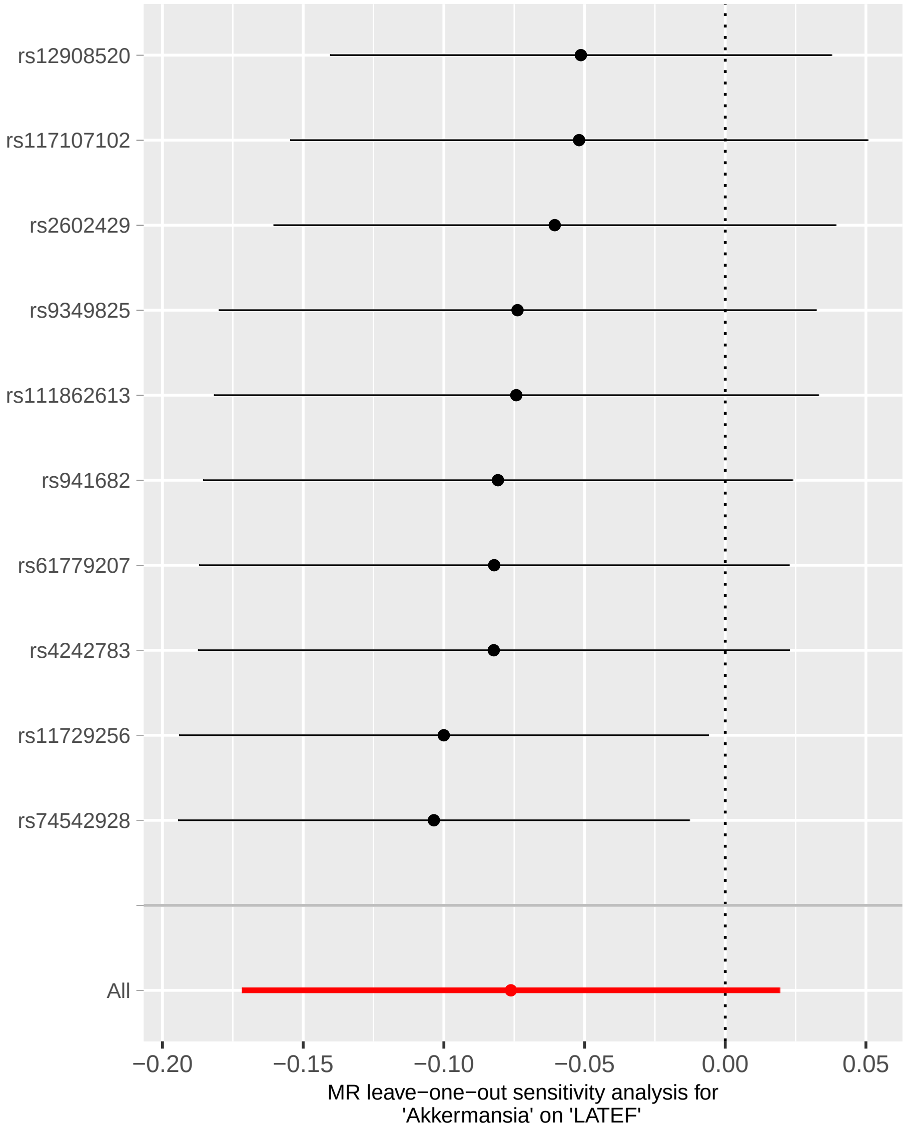
rs12908520
rs117107102
rs2602429
rs9349825
rs111862613
rs941682
rs61779207
rs4242783
rs11729256
rs74542928
All
−0.20
−0.15
−0.10
−0.05
0.00
0.05
MR leave−one−out sensitivity analysis for
'Akkermansia' on 'LATEF'

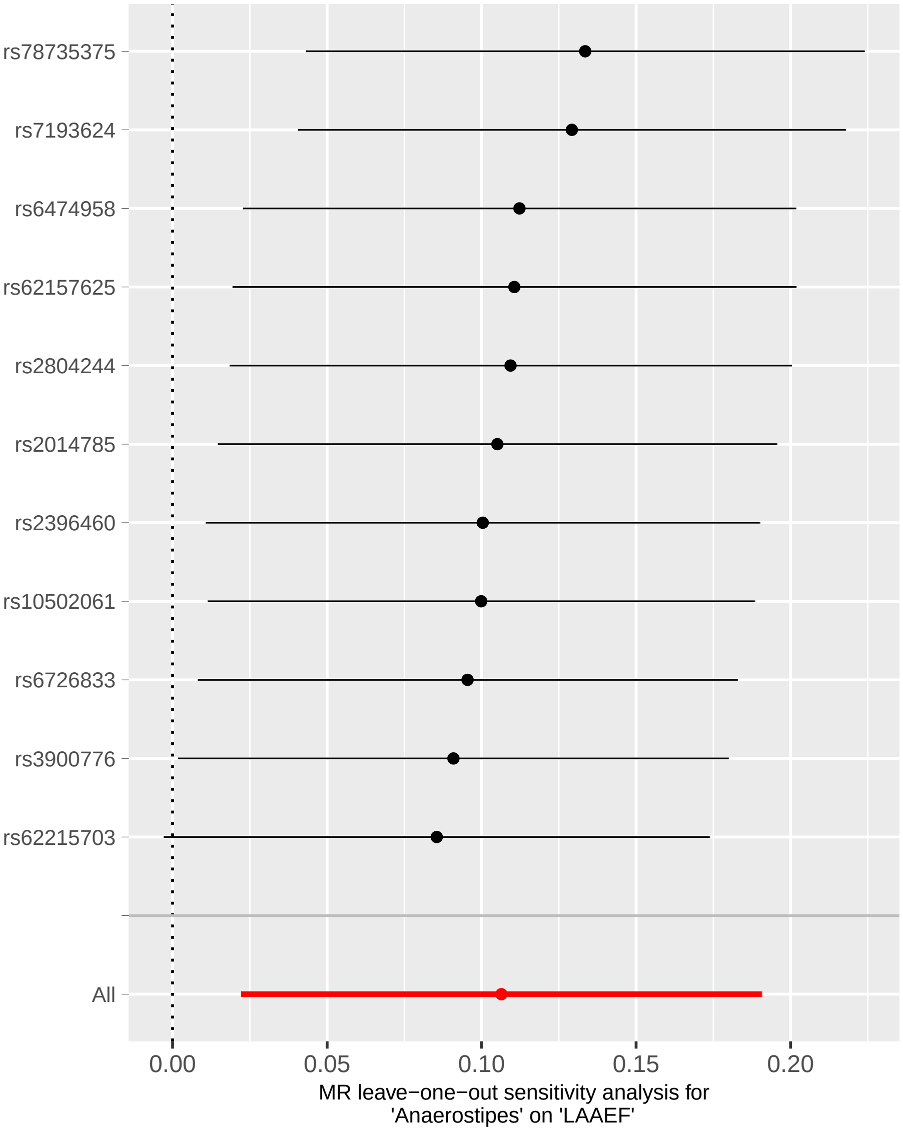
rs78735375
rs7193624
rs6474958
rs62157625
rs2804244
rs2014785
rs2396460
rs10502061
rs6726833
rs3900776
rs62215703
All
0.00
0.05
0.10
0.15
0.20
MR leave−one−out sensitivity analysis for
'Anaerostipes' on 'LAAEF'

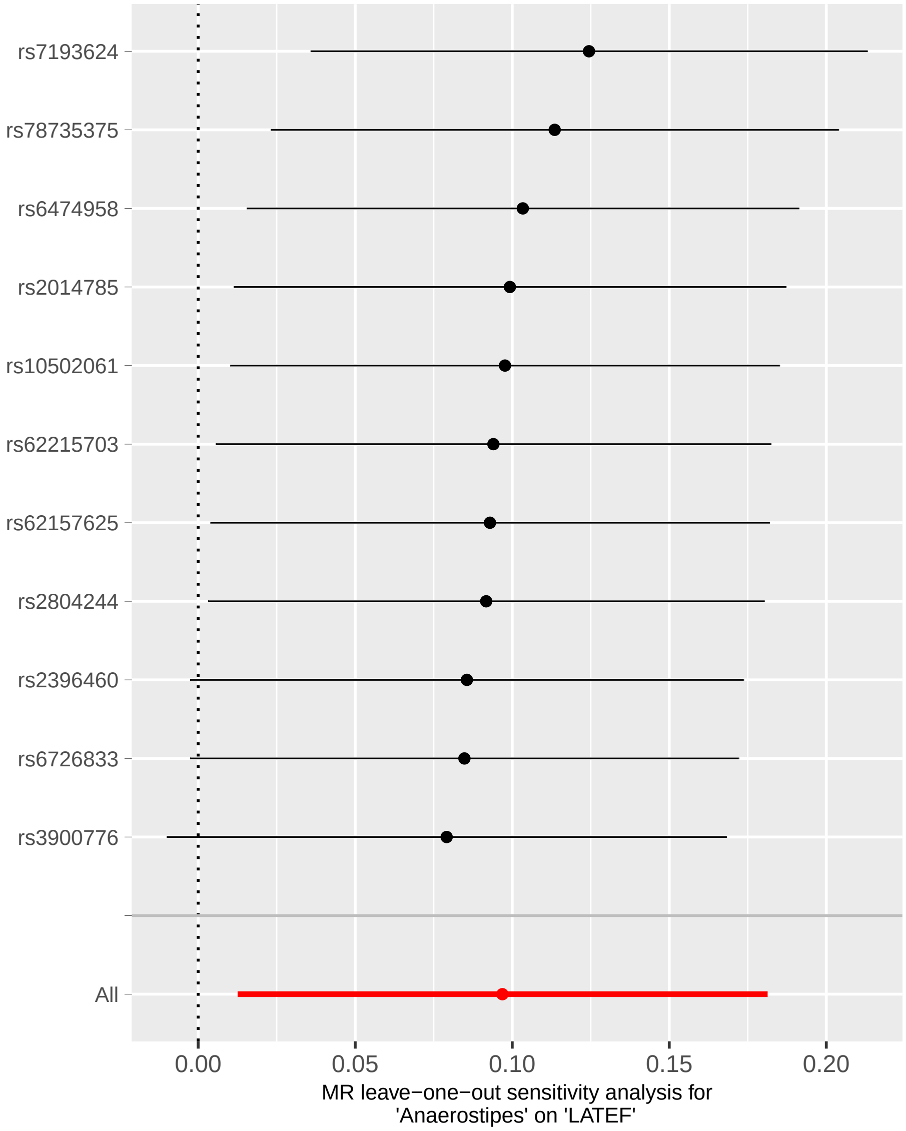

rs7193624
rs78735375
rs6474958
rs2014785
rs10502061
rs62215703
rs62157625
rs2804244
rs2396460
rs6726833
rs3900776
All
0.00
0.05
0.10
0.15
0.20
MR leave−one−out sensitivity analysis for
'Anaerostipes' on 'LATEF'

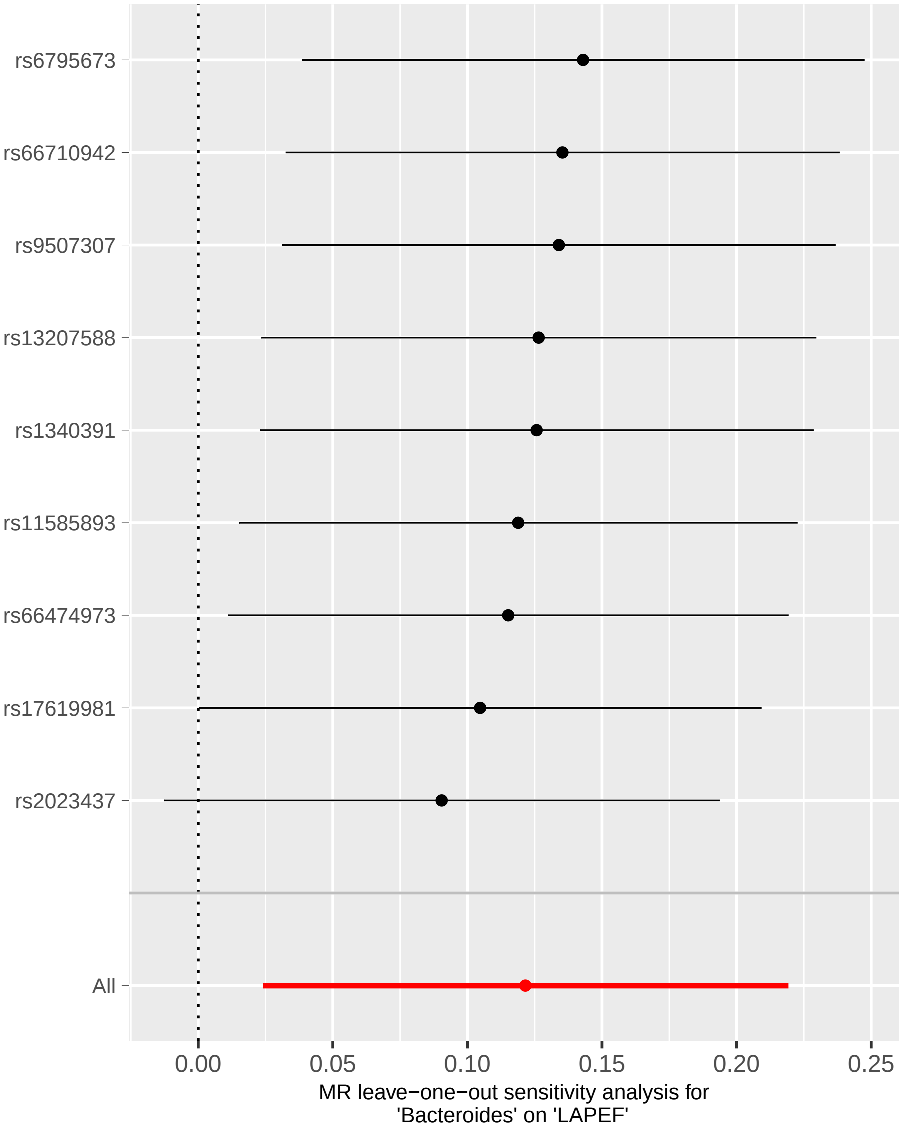

rs6795673
rs66710942
rs9507307
rs13207588
rs1340391
rs11585893
rs66474973
rs17619981
rs2023437
All
0.00
0.05
0.10
0.15
0.20
0.25
MR leave−one−out sensitivity analysis for
'Bacteroides' on 'LAPEF'

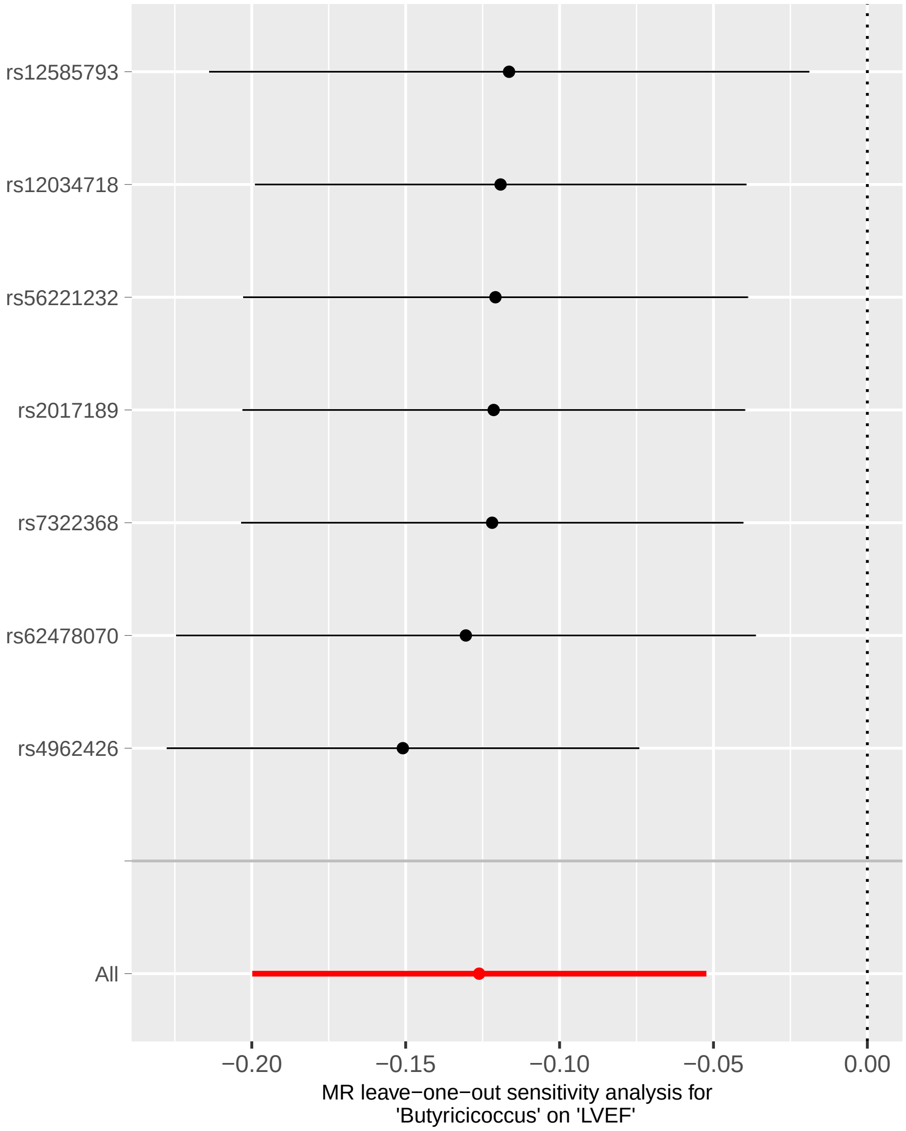

rs12585793
rs12034718
rs56221232
rs2017189
rs7322368
rs62478070
rs4962426
All
−0.20
−0.15
−0.10
−0.05
0.00
MR leave−one−out sensitivity analysis for
'Butyricicoccus' on 'LVEF'

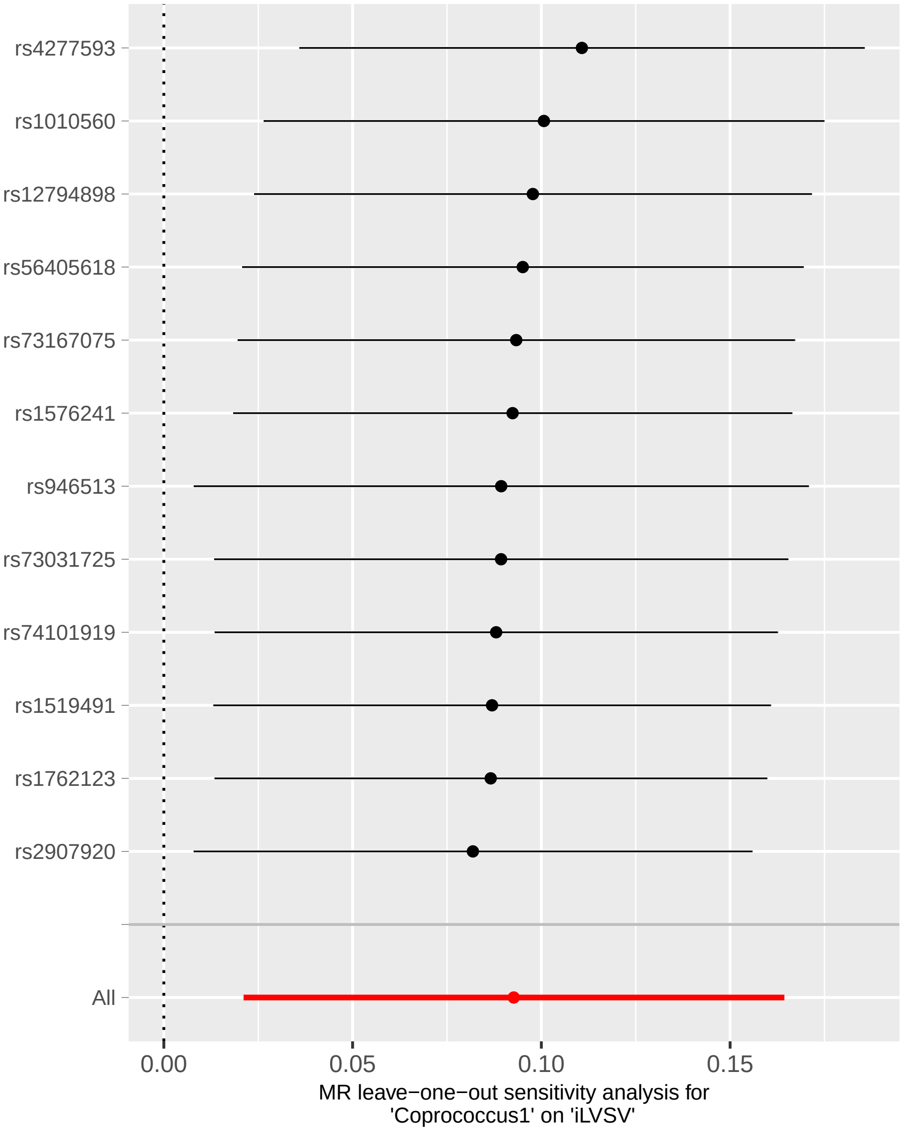

rs4277593
rs1010560
rs12794898
rs56405618
rs73167075
rs1576241
rs946513
rs73031725
rs74101919
rs1519491
rs1762123
rs2907920
All
0.00
0.05
0.10
0.15
MR leave−one−out sensitivity analysis for
'Coprococcus1' on 'iLVSV'

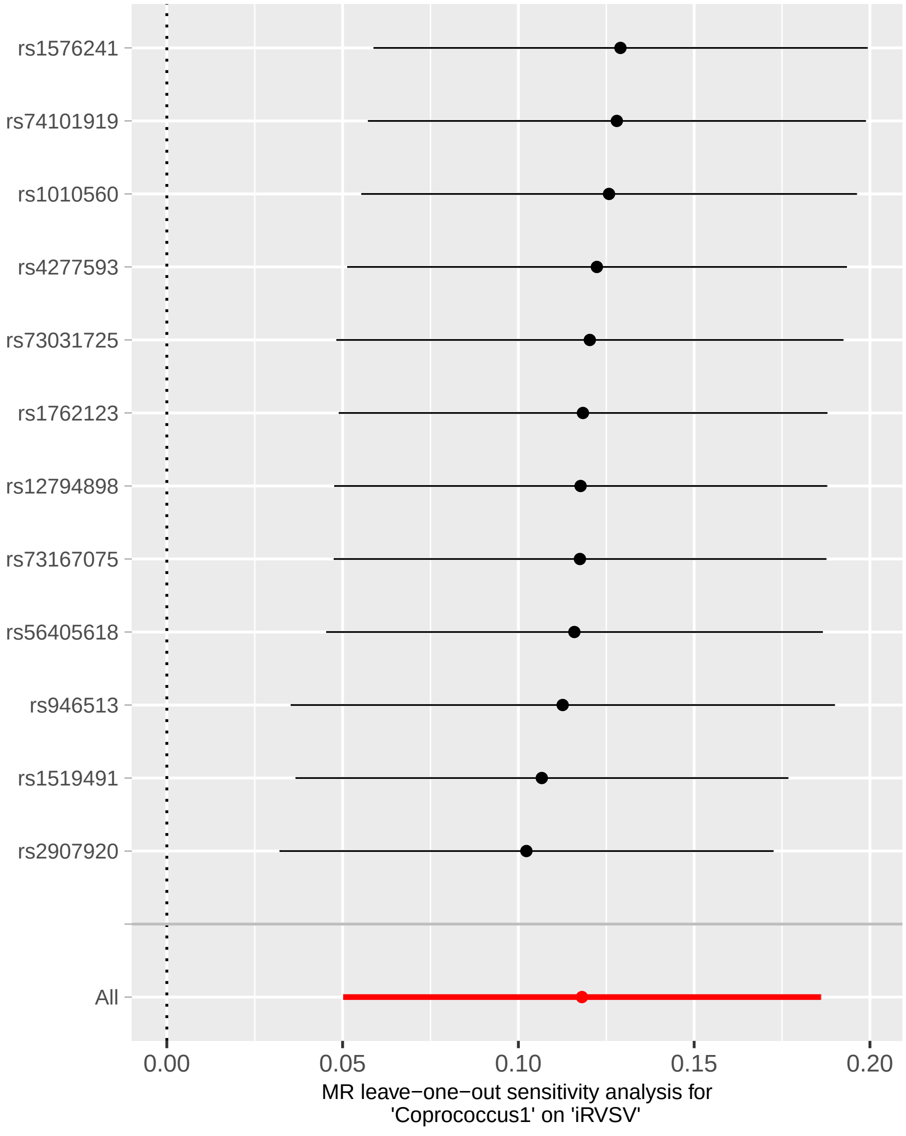
rs1576241
rs74101919
rs1010560
rs4277593
rs73031725
rs1762123
rs12794898
rs73167075
rs56405618
rs946513
rs1519491
rs2907920
All
0.00
0.05
0.10
0.15
0.20
MR leave−one−out sensitivity analysis for
'Coprococcus1' on 'iRVSV'

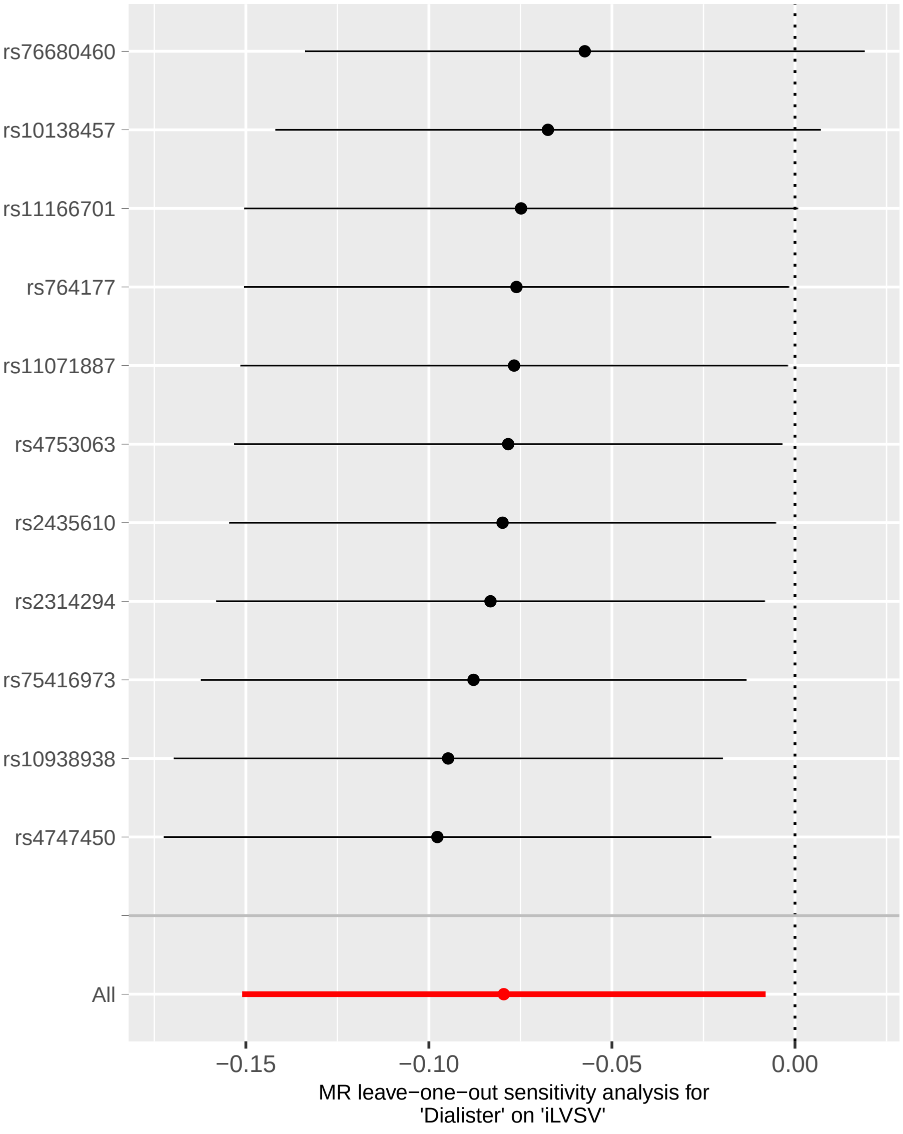

rs76680460
rs10138457
rs11166701
rs764177
rs11071887
rs4753063
rs2435610
rs2314294
rs75416973
rs10938938
rs4747450
All
−0.15
−0.10
−0.05
0.00
MR leave−one−out sensitivity analysis for
'Dialister' on 'iLVSV'

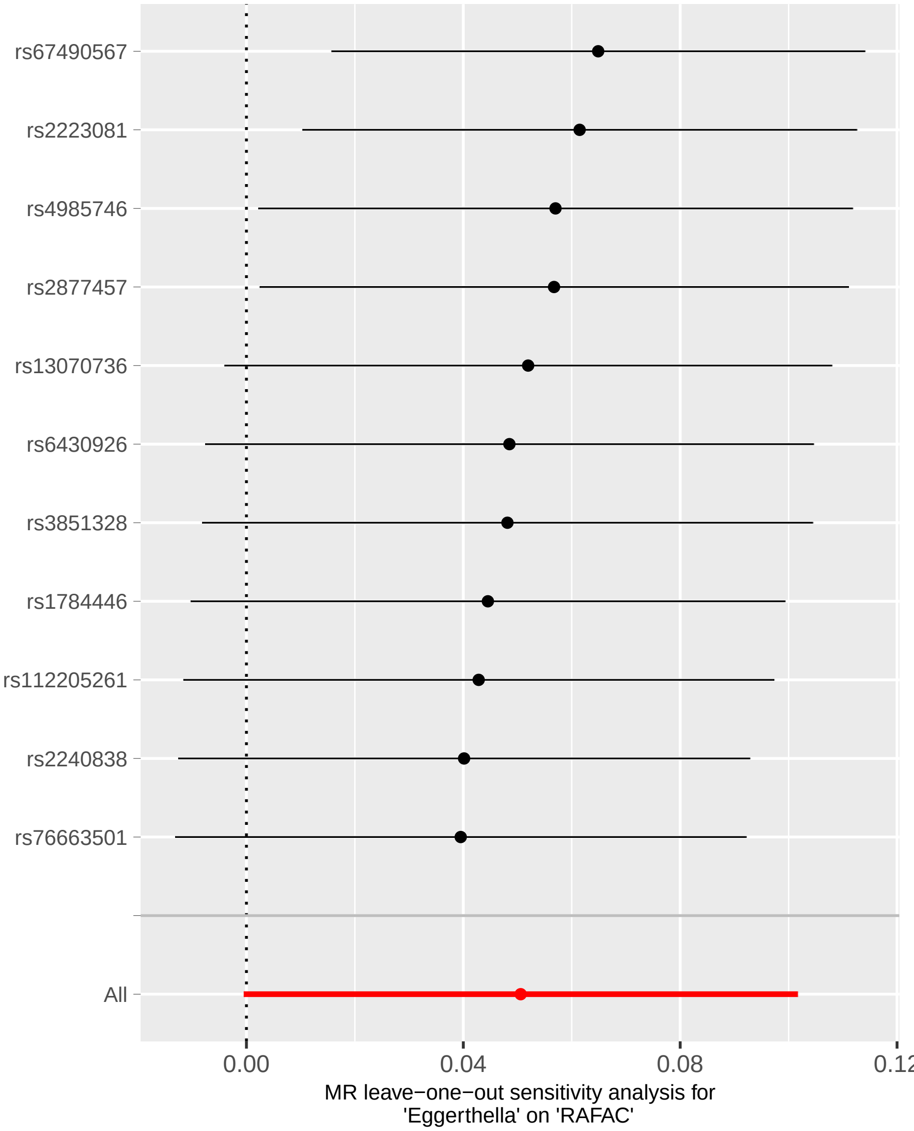

rs67490567
rs2223081
rs4985746
rs2877457
rs13070736
rs6430926
rs3851328
rs1784446
rs112205261
rs2240838
rs76663501
All
0.00
0.04
0.08
0.12
MR leave−one−out sensitivity analysis for
'Eggerthella' on 'RAFAC'

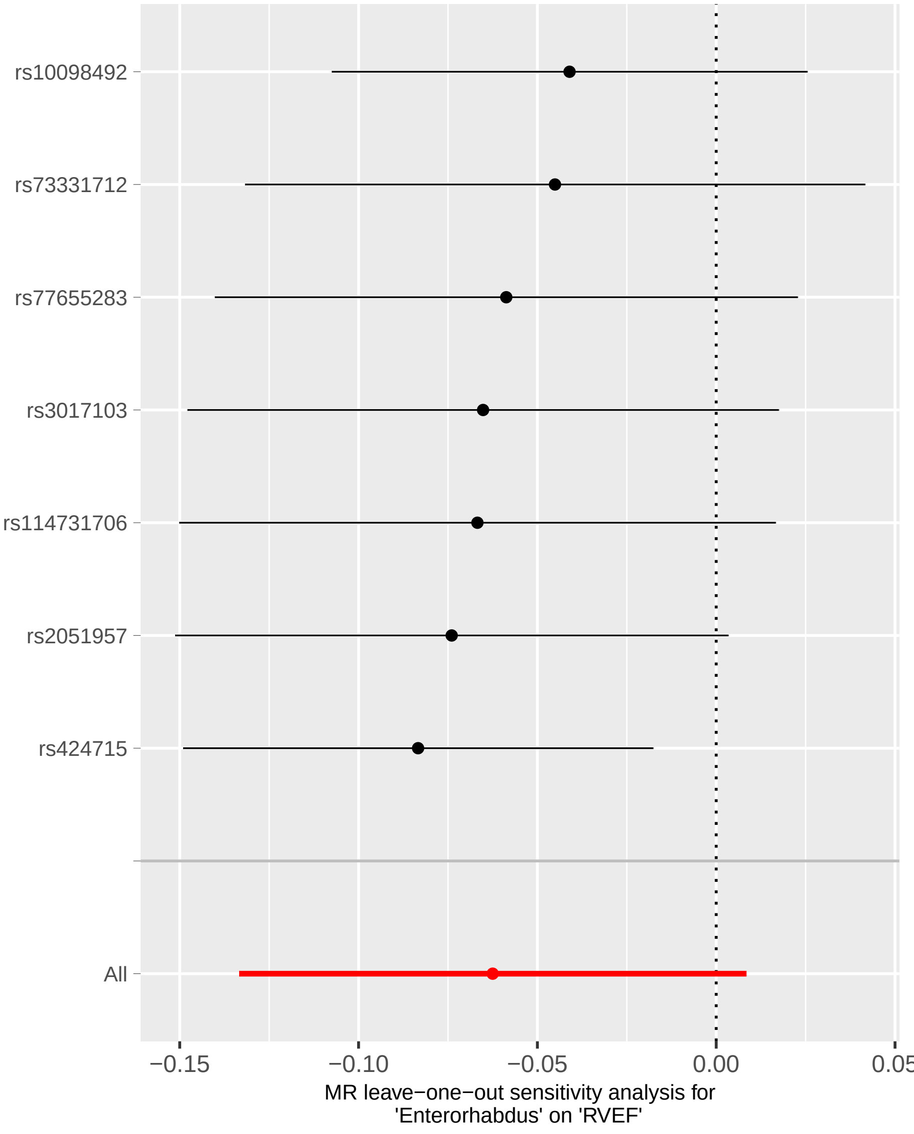
rs10098492
rs73331712
rs77655283
rs3017103
rs114731706
rs2051957
rs424715
All
−0.15
−0.10
−0.05
0.00
0.05
MR leave−one−out sensitivity analysis for
'Enterorhabdus' on 'RVEF'

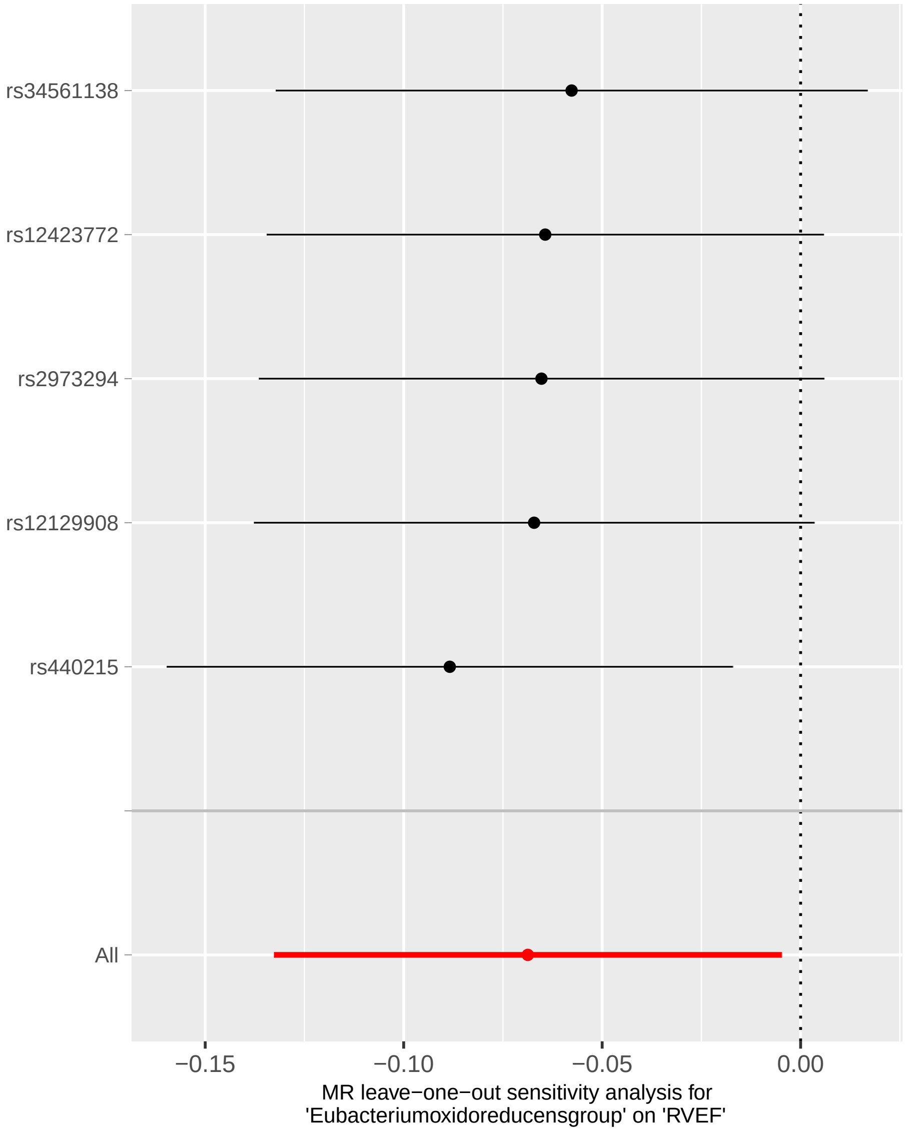
rs34561138
rs12423772
rs2973294
rs12129908
rs440215
All
−0.15
−0.10
−0.05
0.00
MR leave−one−out sensitivity analysis for
'Eubacteriumoxidoreducensgroup' on 'RVEF'

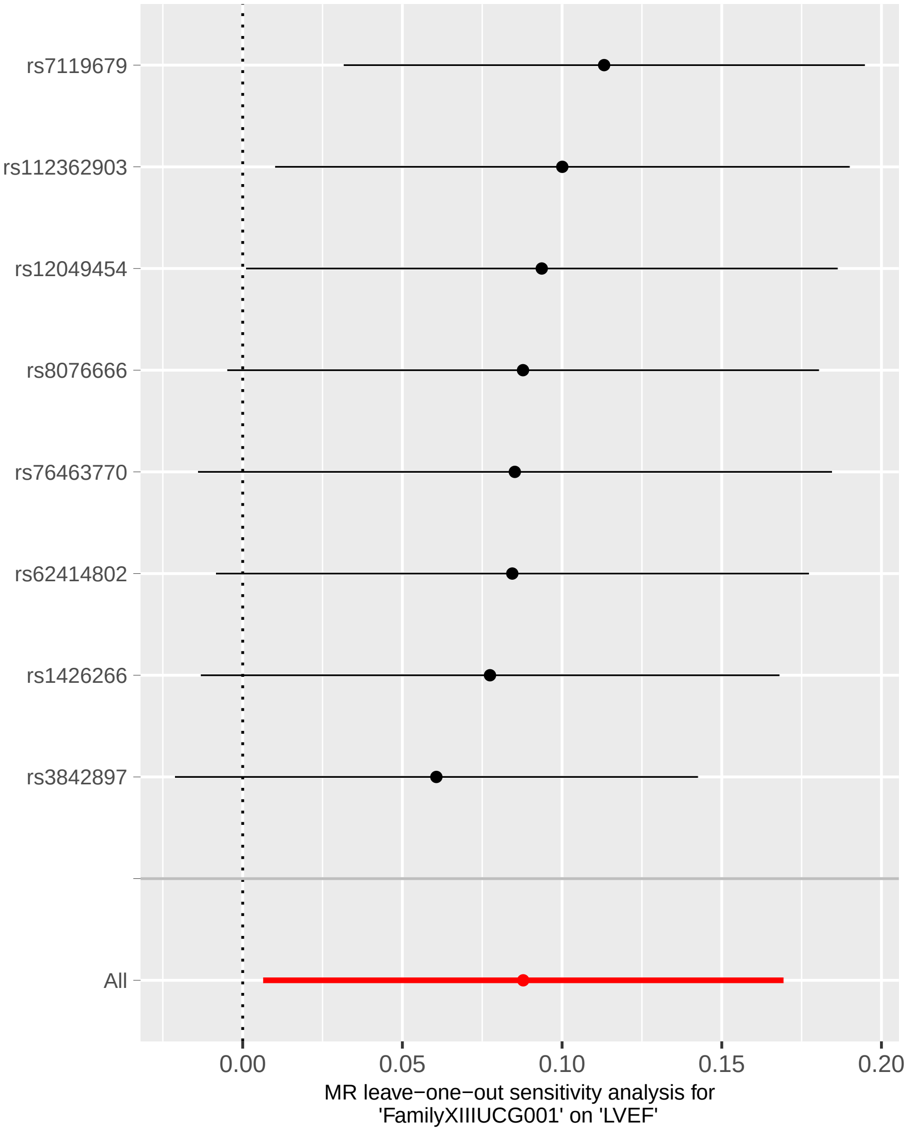
rs7119679
rs112362903
rs12049454
rs8076666
rs76463770
rs62414802
rs1426266
rs3842897
All
0.00
0.05
0.10
0.15
0.20
MR leave−one−out sensitivity analysis for
'FamilyXIIIUCG001' on 'LVEF'

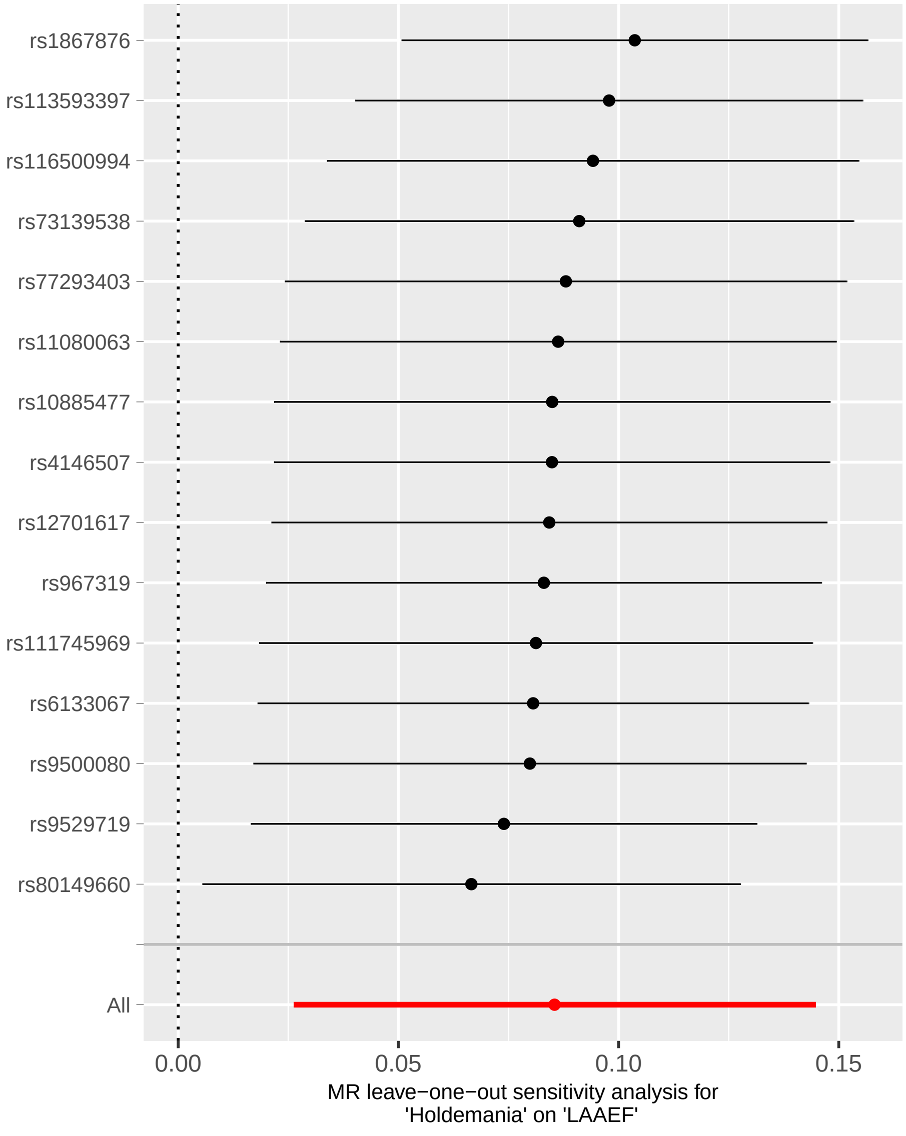
rs1867876
rs113593397
rs116500994
rs73139538
rs77293403
rs11080063
rs10885477
rs4146507
rs12701617
rs967319
rs111745969
rs6133067
rs9500080
rs9529719
rs80149660
All
0.00
0.05
0.10
0.15
MR leave−one−out sensitivity analysis for
'Holdemania' on 'LAAEF'

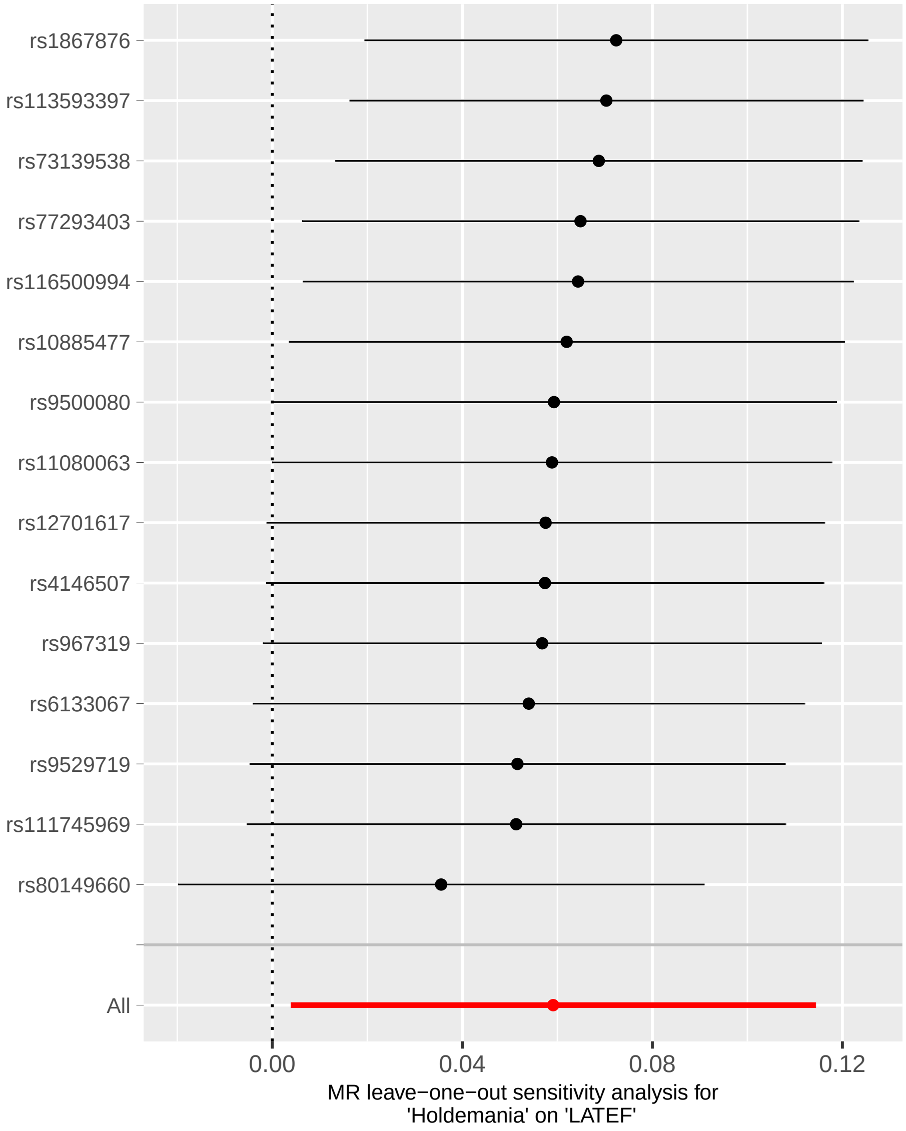

rs1867876
rs113593397
rs73139538
rs77293403
rs116500994
rs10885477
rs9500080
rs11080063
rs12701617
rs4146507
rs967319
rs6133067
rs9529719
rs111745969
rs80149660
All
0.00
0.04
0.08
0.12
MR leave−one−out sensitivity analysis for
'Holdemania' on 'LATEF'

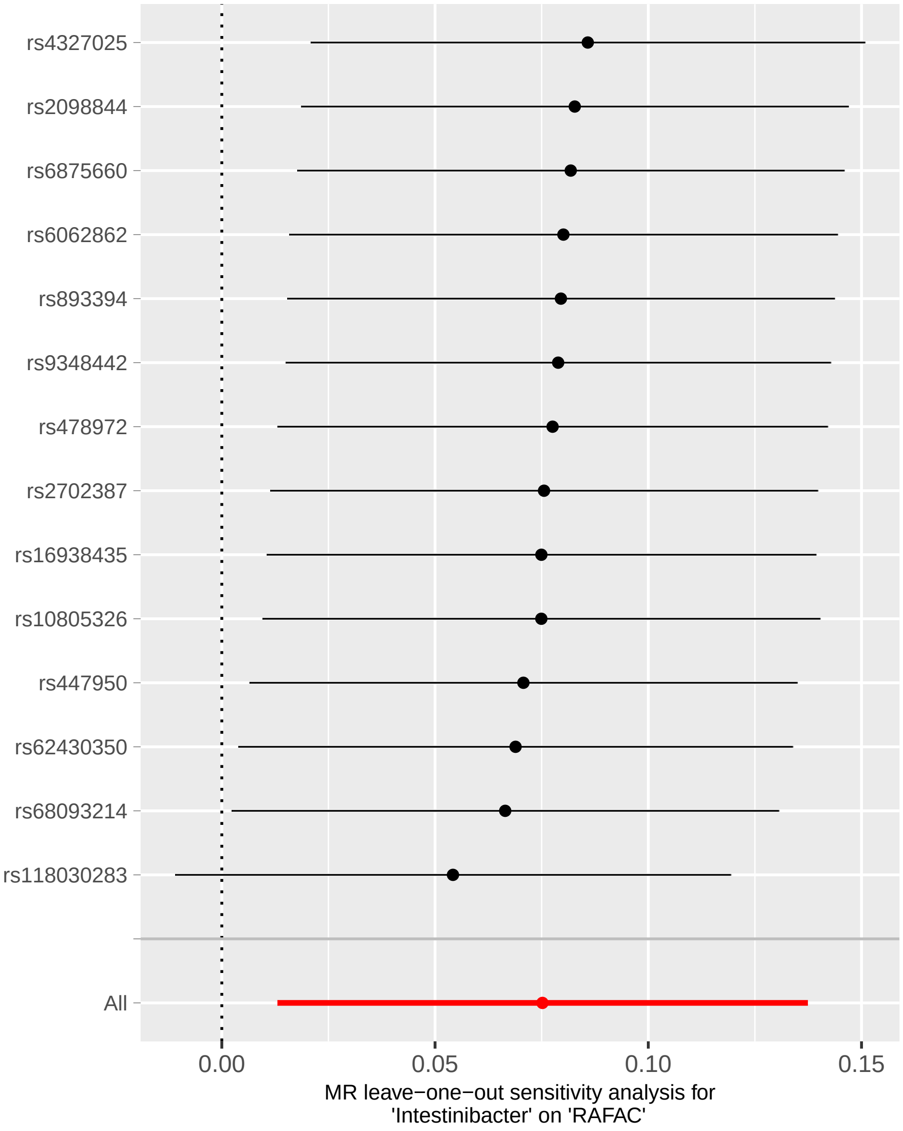
rs4327025
rs2098844
rs6875660
rs6062862
rs893394
rs9348442
rs478972
rs2702387
rs16938435
rs10805326
rs447950
rs62430350
rs68093214
rs118030283
All
0.00
0.05
0.10
0.15
MR leave−one−out sensitivity analysis for
'Intestinibacter' on 'RAFAC'

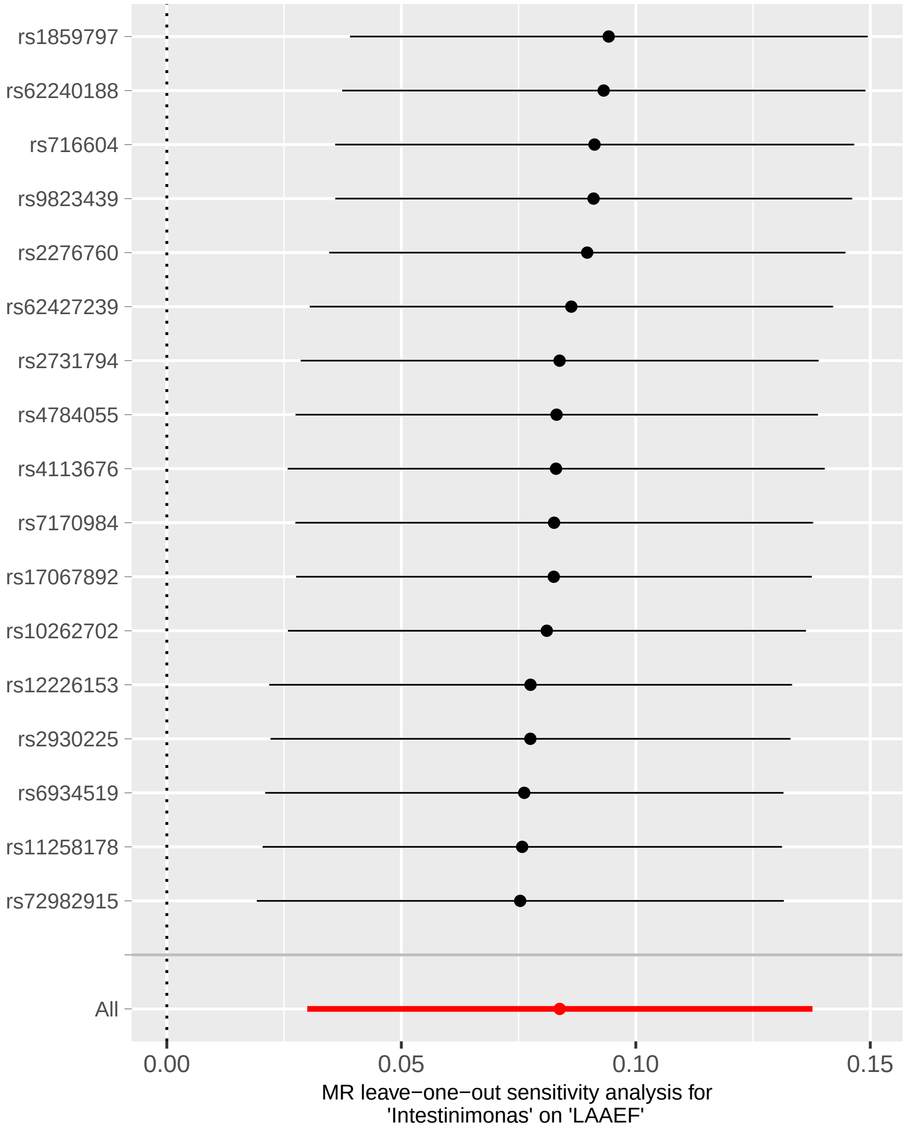
rs1859797
rs62240188
rs716604
rs9823439
rs2276760
rs62427239
rs2731794
rs4784055
rs4113676
rs7170984
rs17067892
rs10262702
rs12226153
rs2930225
rs6934519
rs11258178
rs72982915
All
0.00
0.05
0.10
0.15
MR leave−one−out sensitivity analysis for
'Intestinimonas' on 'LAAEF'

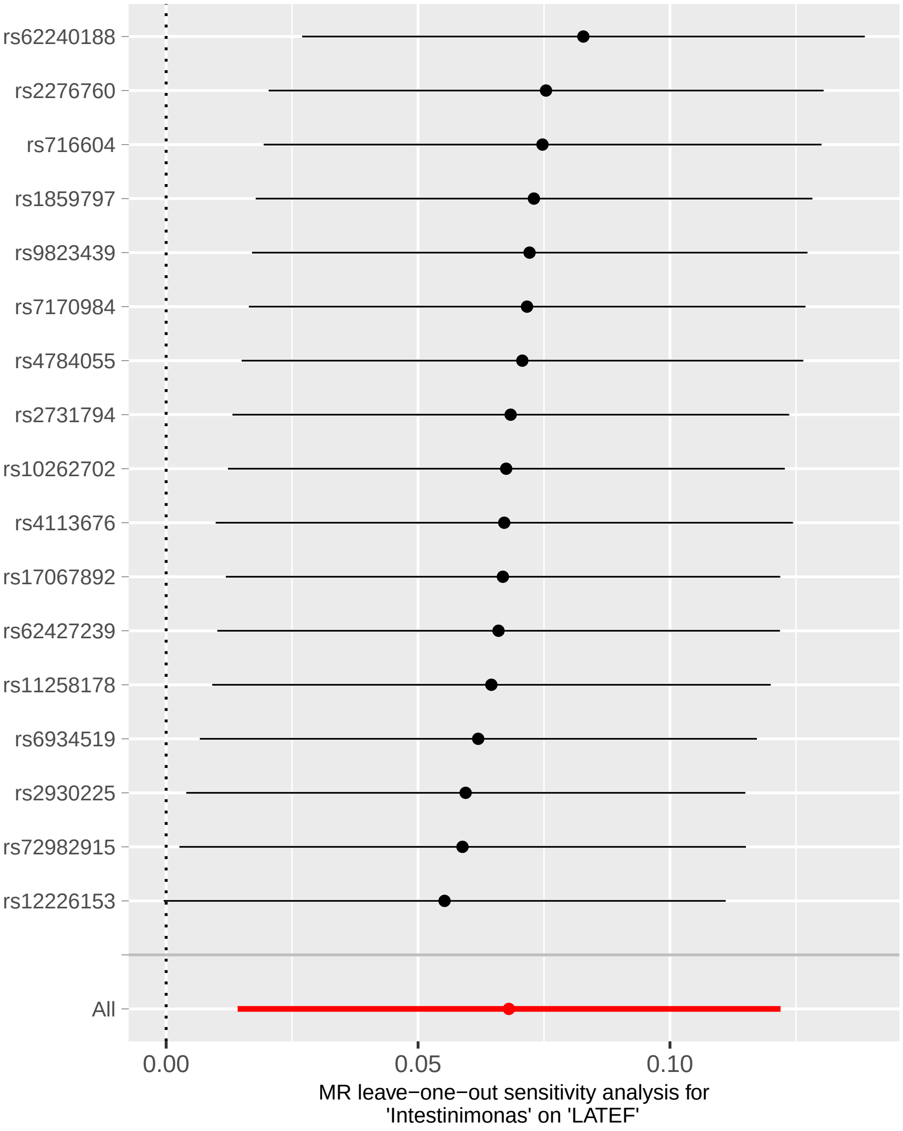
rs62240188
rs2276760
rs716604
rs1859797
rs9823439
rs7170984
rs4784055
rs2731794
rs10262702
rs4113676
rs17067892
rs62427239
rs11258178
rs6934519
rs2930225
rs72982915
rs12226153
All
0.00
0.05
0.10
MR leave−one−out sensitivity analysis for
'Intestinimonas' on 'LATEF'

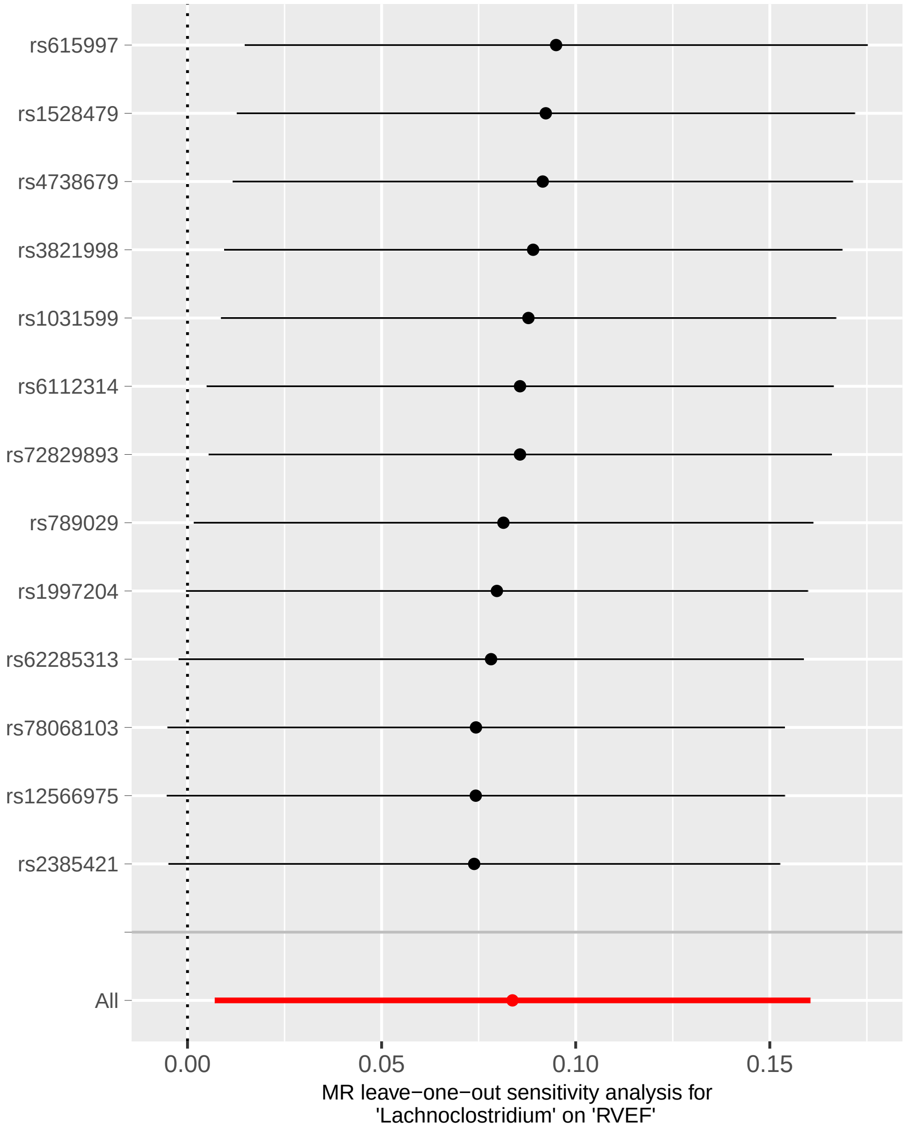
rs615997
rs1528479
rs4738679
rs3821998
rs1031599
rs6112314
rs72829893
rs789029
rs1997204
rs62285313
rs78068103
rs12566975
rs2385421
All
0.00
0.05
0.10
0.15
MR leave−one−out sensitivity analysis for
'Lachnoclostridium' on 'RVEF'

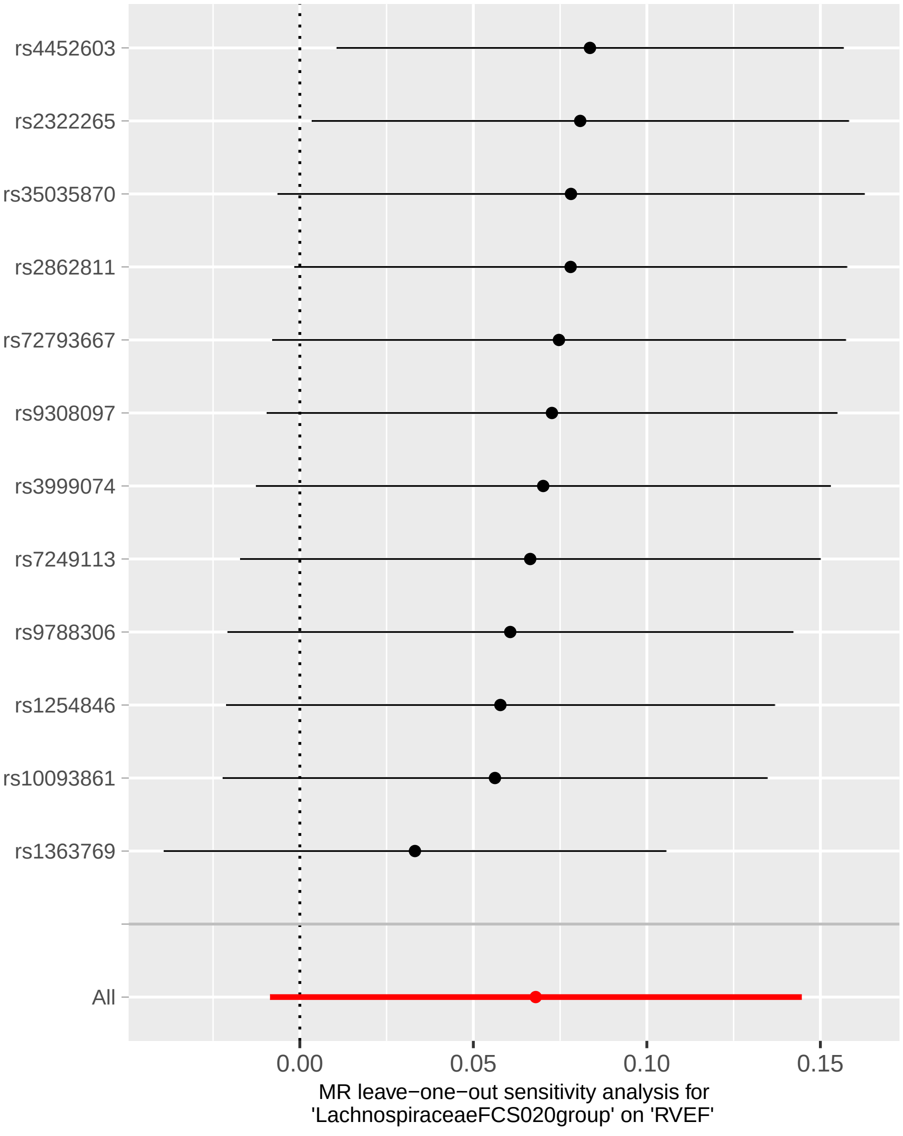
rs4452603
rs2322265
rs35035870
rs2862811
rs72793667
rs9308097
rs3999074
rs7249113
rs9788306
rs1254846
rs10093861
rs1363769
All
0.00
0.05
0.10
0.15
MR leave−one−out sensitivity analysis for
'LachnospiraceaeFCS020group' on 'RVEF'

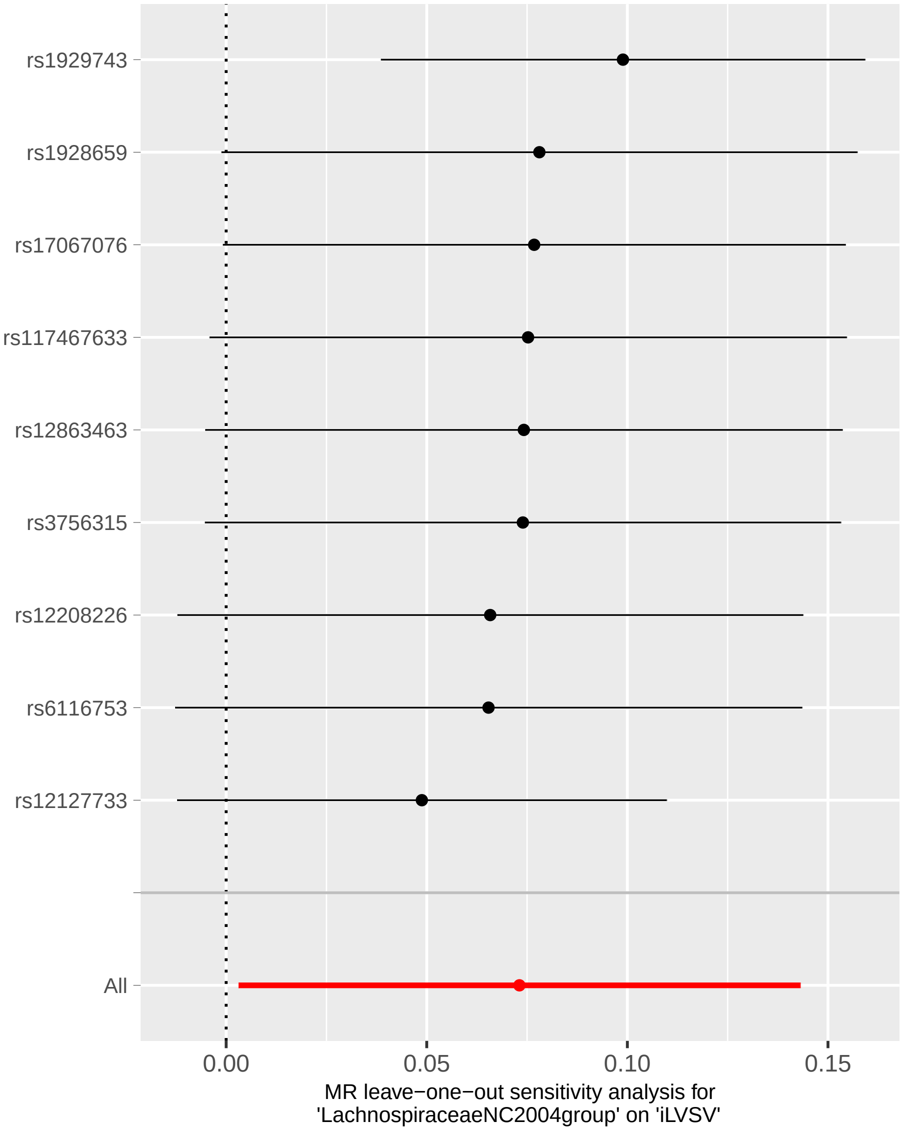
rs1929743
rs1928659
rs17067076
rs117467633
rs12863463
rs3756315
rs12208226
rs6116753
rs12127733
All
0.00
0.05
0.10
0.15
MR leave−one−out sensitivity analysis for
'LachnospiraceaeNC2004group' on 'iLVSV'

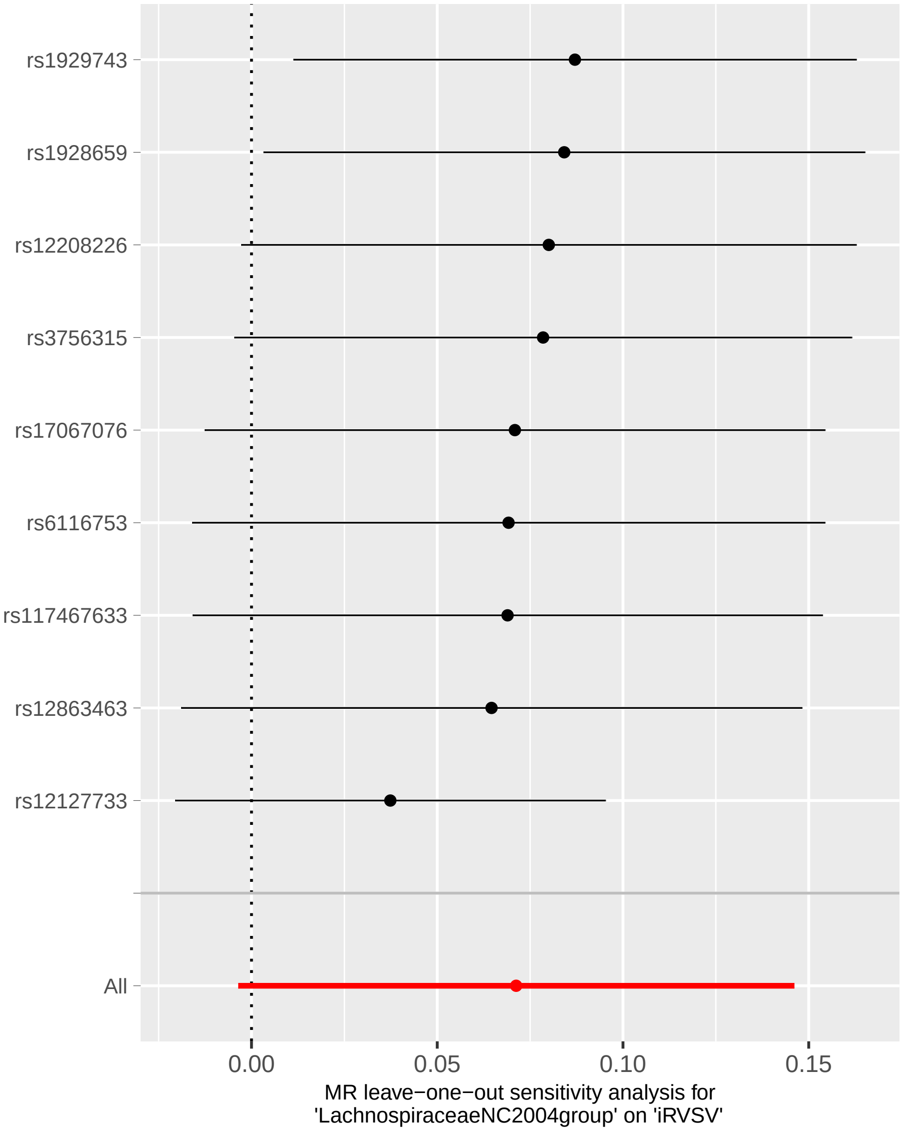
rs1929743
rs1928659
rs12208226
rs3756315
rs17067076
rs6116753
rs117467633
rs12863463
rs12127733
All
0.00
0.05
0.10
0.15
MR leave−one−out sensitivity analysis for
'LachnospiraceaeNC2004group' on 'iRVSV'

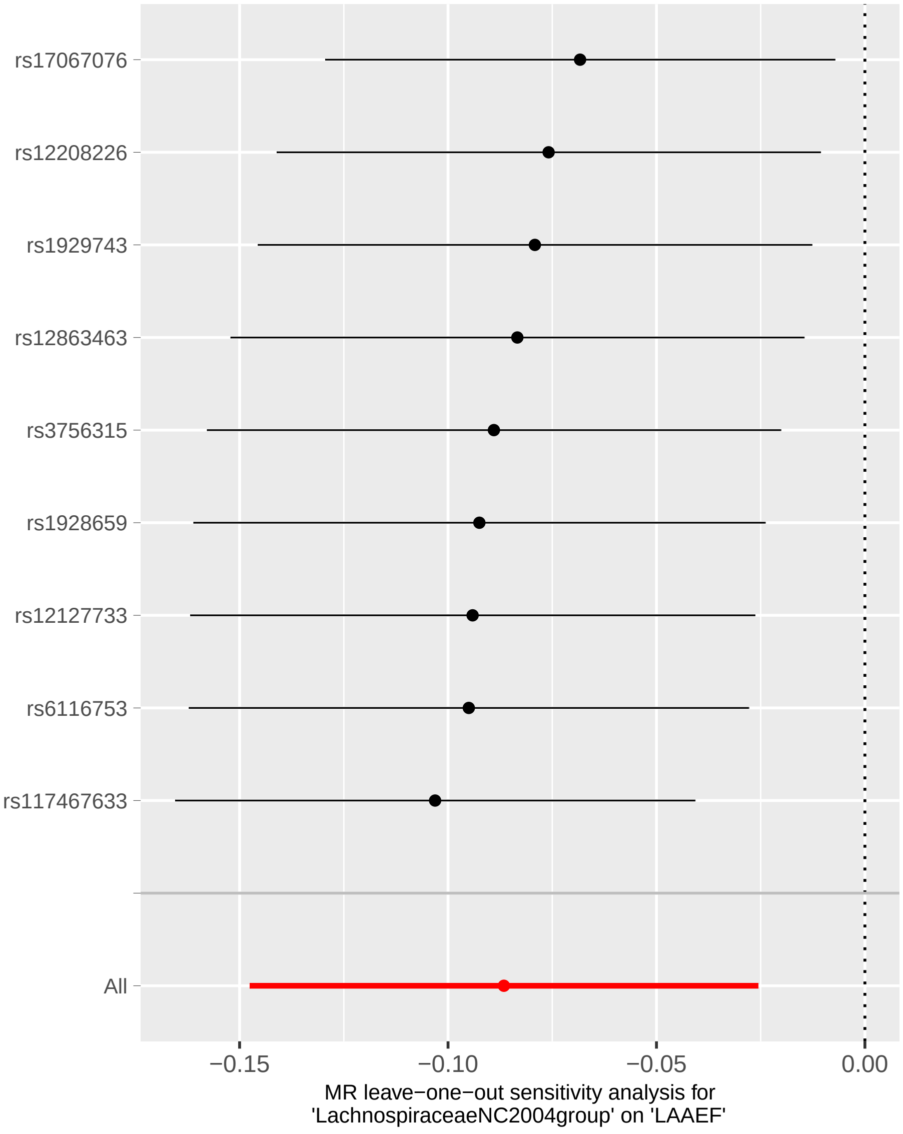
rs17067076
rs12208226
rs1929743
rs12863463
rs3756315
rs1928659
rs12127733
rs6116753
rs117467633
All
−0.15
−0.10
−0.05
0.00
MR leave−one−out sensitivity analysis for
'LachnospiraceaeNC2004group' on 'LAAEF'

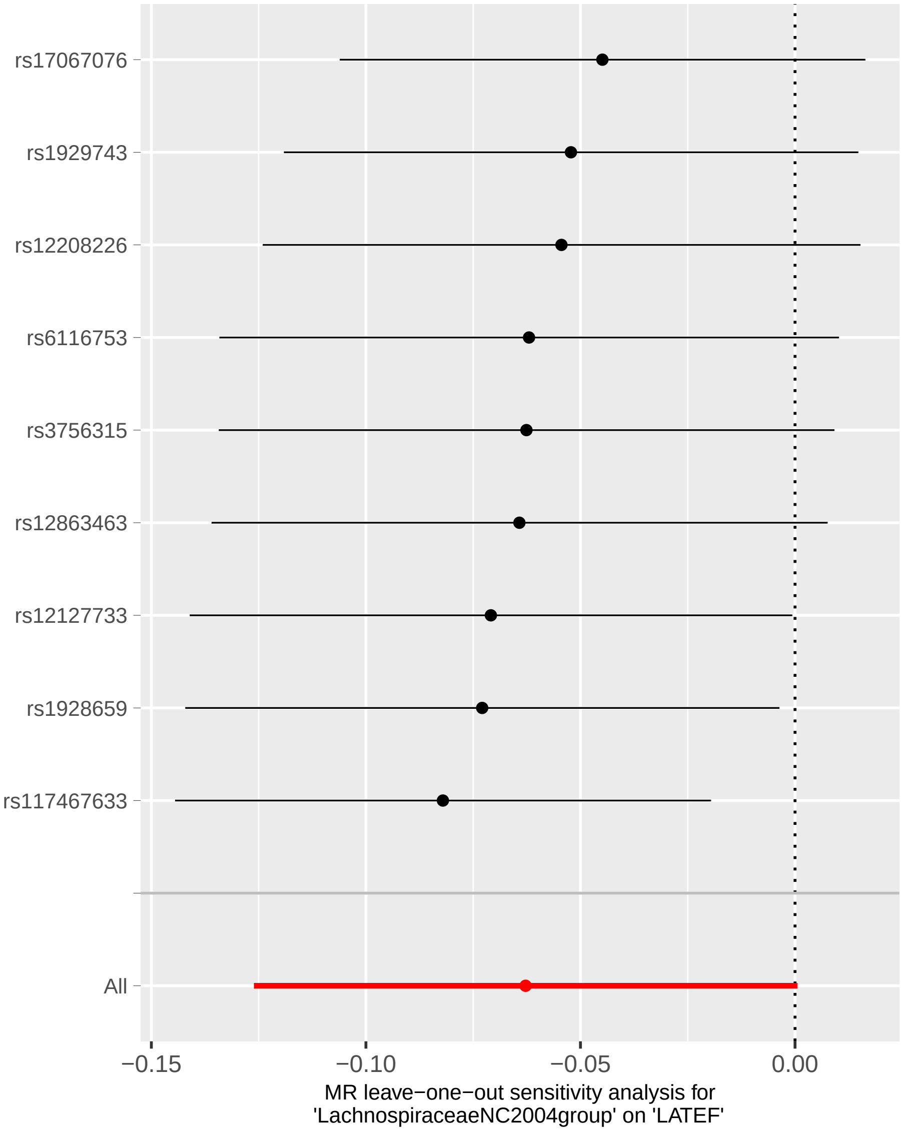

rs17067076
rs1929743
rs12208226
rs6116753
rs3756315
rs12863463
rs12127733
rs1928659
rs117467633
All
−0.15
−0.10
−0.05
0.00
MR leave−one−out sensitivity analysis for
'LachnospiraceaeNC2004group' on 'LATEF'

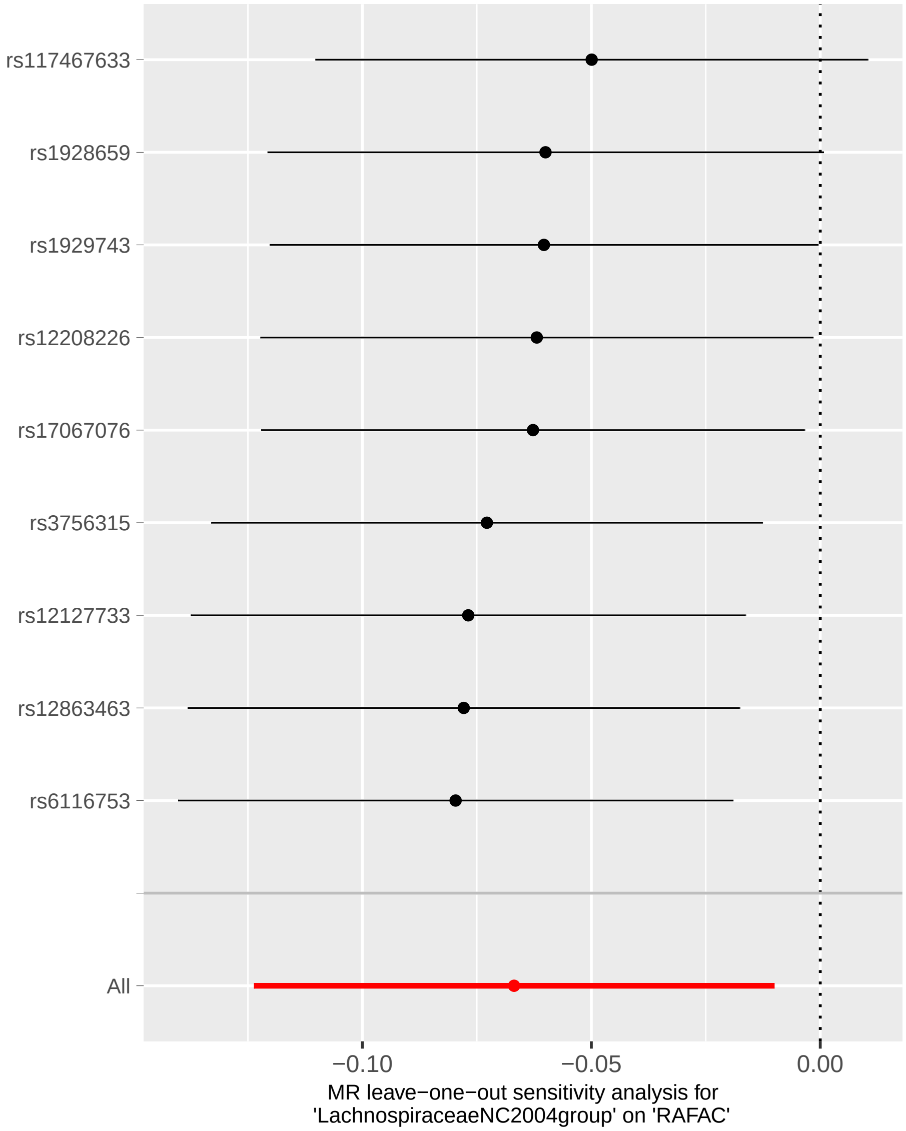

rs117467633
rs1928659
rs1929743
rs12208226
rs17067076
rs3756315
rs12127733
rs12863463
rs6116753
All
−0.10
−0.05
0.00
MR leave−one−out sensitivity analysis for
'LachnospiraceaeNC2004group' on 'RAFAC'

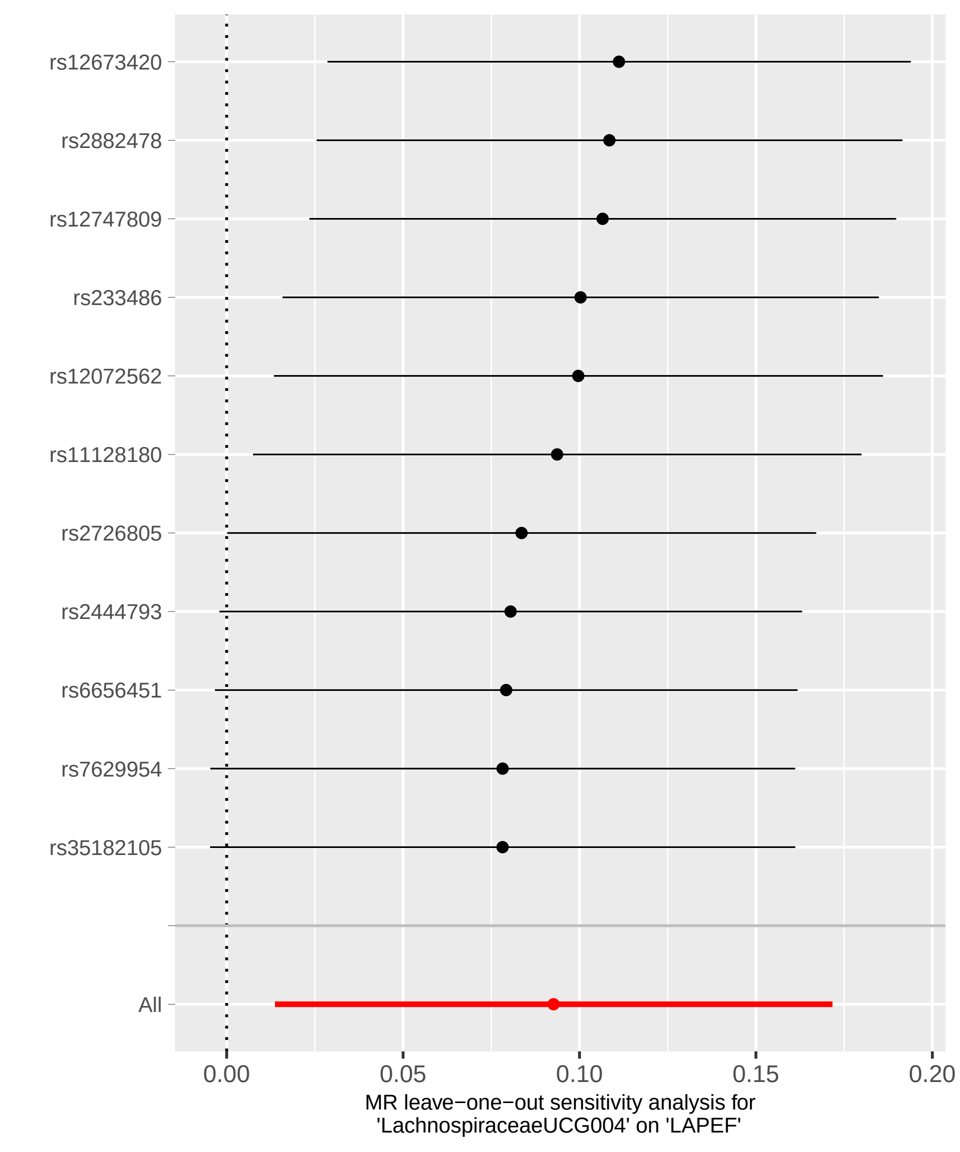
rs12673420
rs2882478
rs12747809
rs233486
rs12072562
rs11128180
rs2726805
rs2444793
rs6656451
rs7629954
rs35182105
All
0.00
0.05
0.10
0.15
0.20
MR leave−one−out sensitivity analysis for
'LachnospiraceaeUCG004' on 'LAPEF'

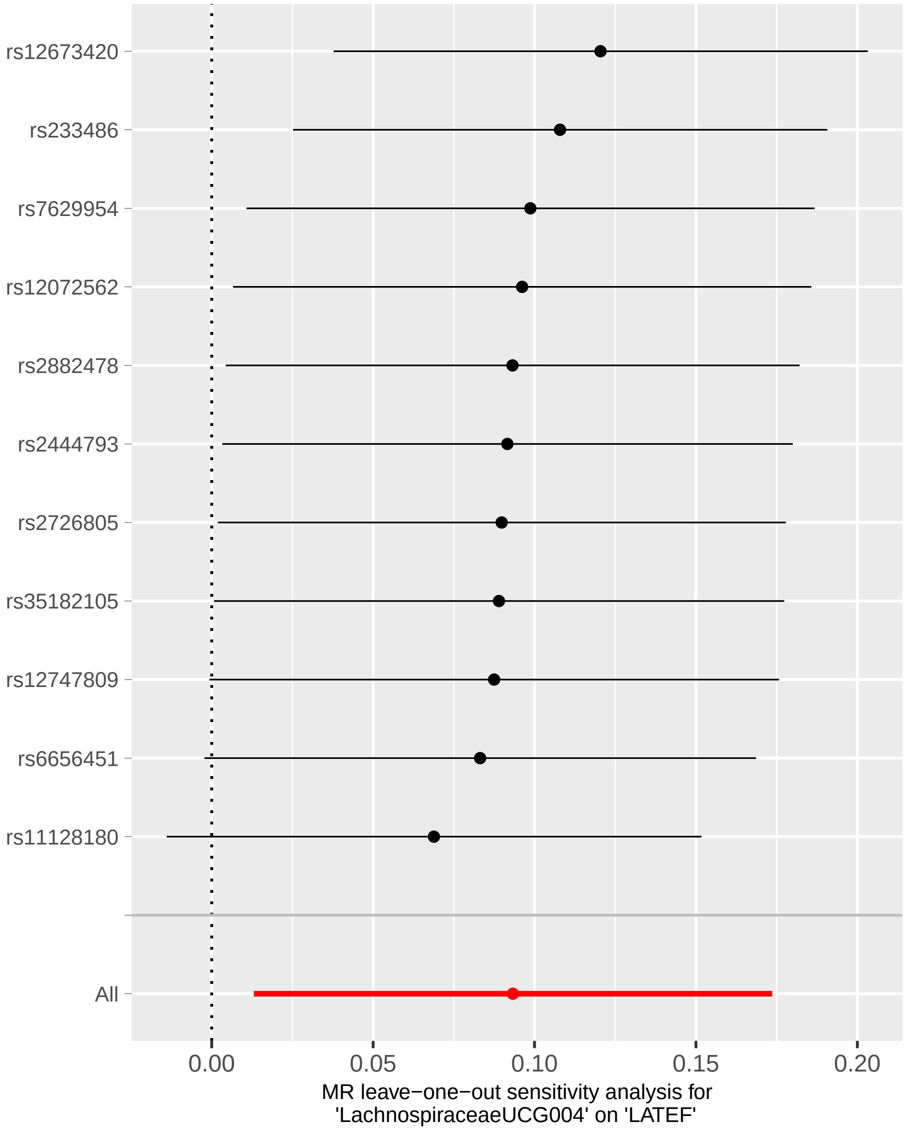

rs12673420
rs233486
rs7629954
rs12072562
rs2882478
rs2444793
rs2726805
rs35182105
rs12747809
rs6656451
rs11128180
All
0.00
0.05
0.10
0.15
0.20
MR leave−one−out sensitivity analysis for
'LachnospiraceaeUCG004' on 'LATEF'

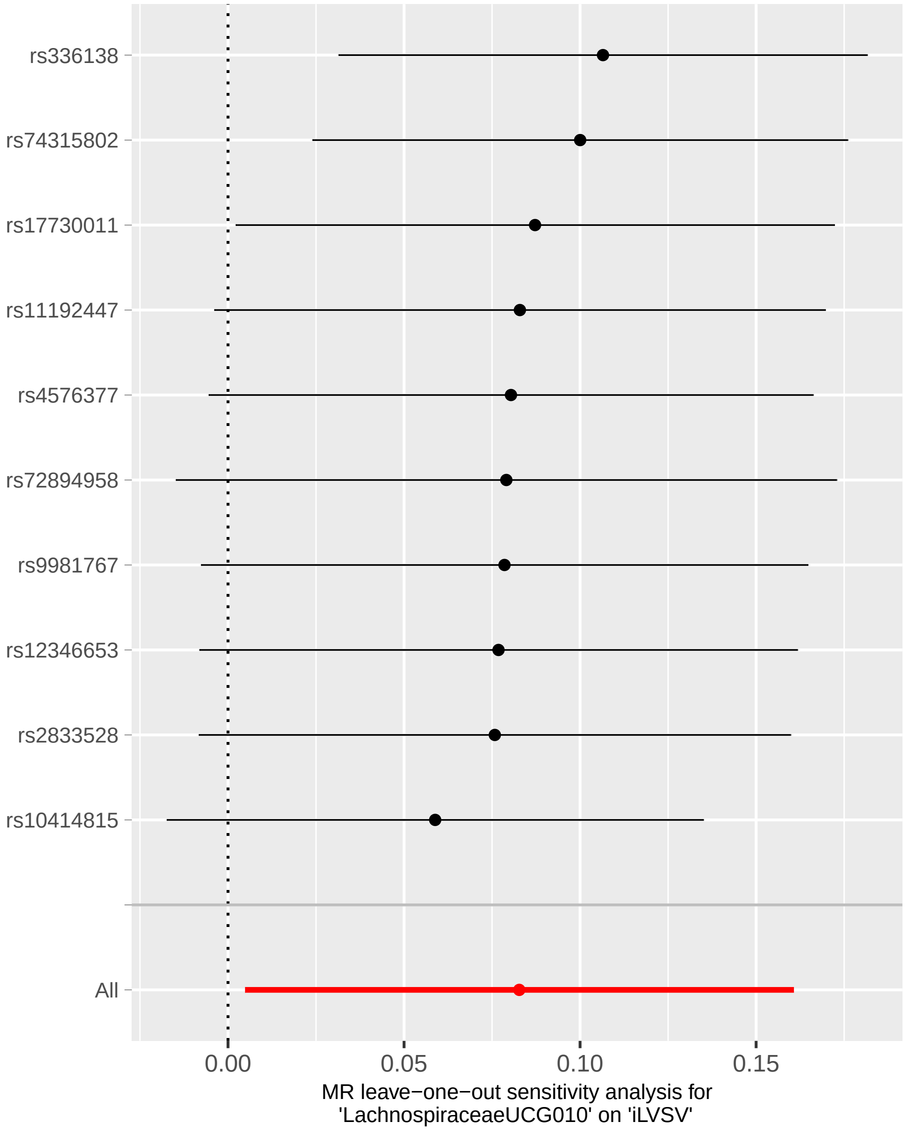

rs336138
rs74315802
rs17730011
rs11192447
rs4576377
rs72894958
rs9981767
rs12346653
rs2833528
rs10414815
All
0.00
0.05
0.10
0.15
MR leave−one−out sensitivity analysis for
'LachnospiraceaeUCG010' on 'iLVSV'

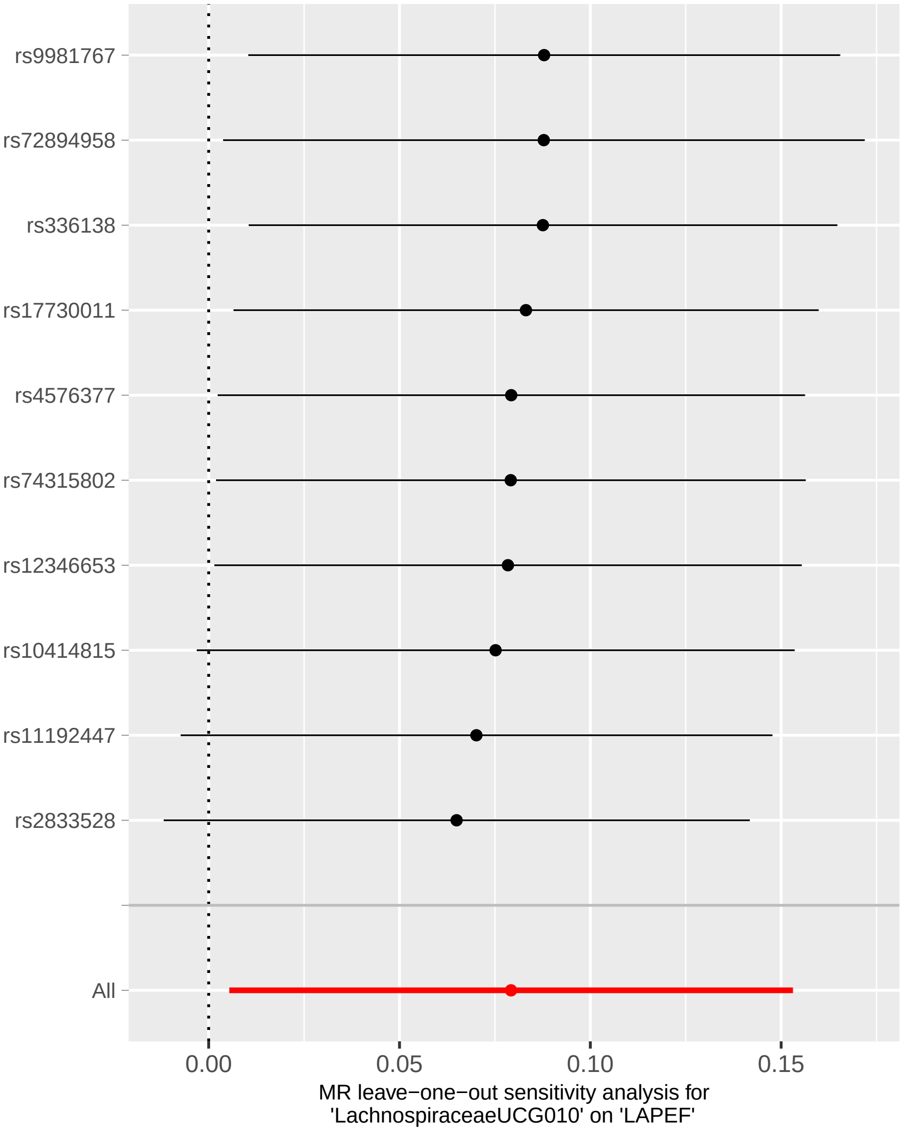
rs9981767
rs72894958
rs336138
rs17730011
rs4576377
rs74315802
rs12346653
rs10414815
rs11192447
rs2833528
All
0.00
0.05
0.10
0.15
MR leave−one−out sensitivity analysis for
'LachnospiraceaeUCG010' on 'LAPEF'

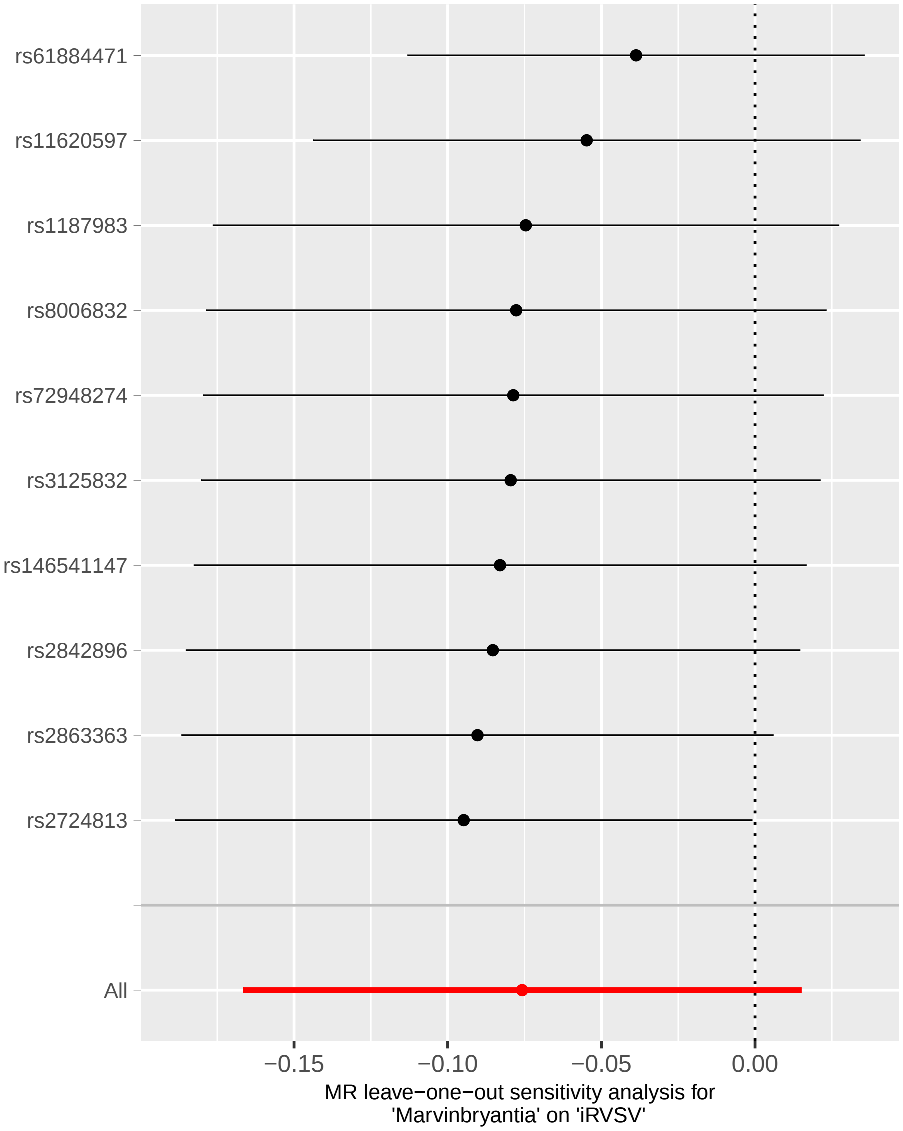

rs61884471
rs11620597
rs1187983
rs8006832
rs72948274
rs3125832
rs146541147
rs2842896
rs2863363
rs2724813
All
−0.15
−0.10
−0.05
0.00
MR leave−one−out sensitivity analysis for
'Marvinbryantia' on 'iRVSV'

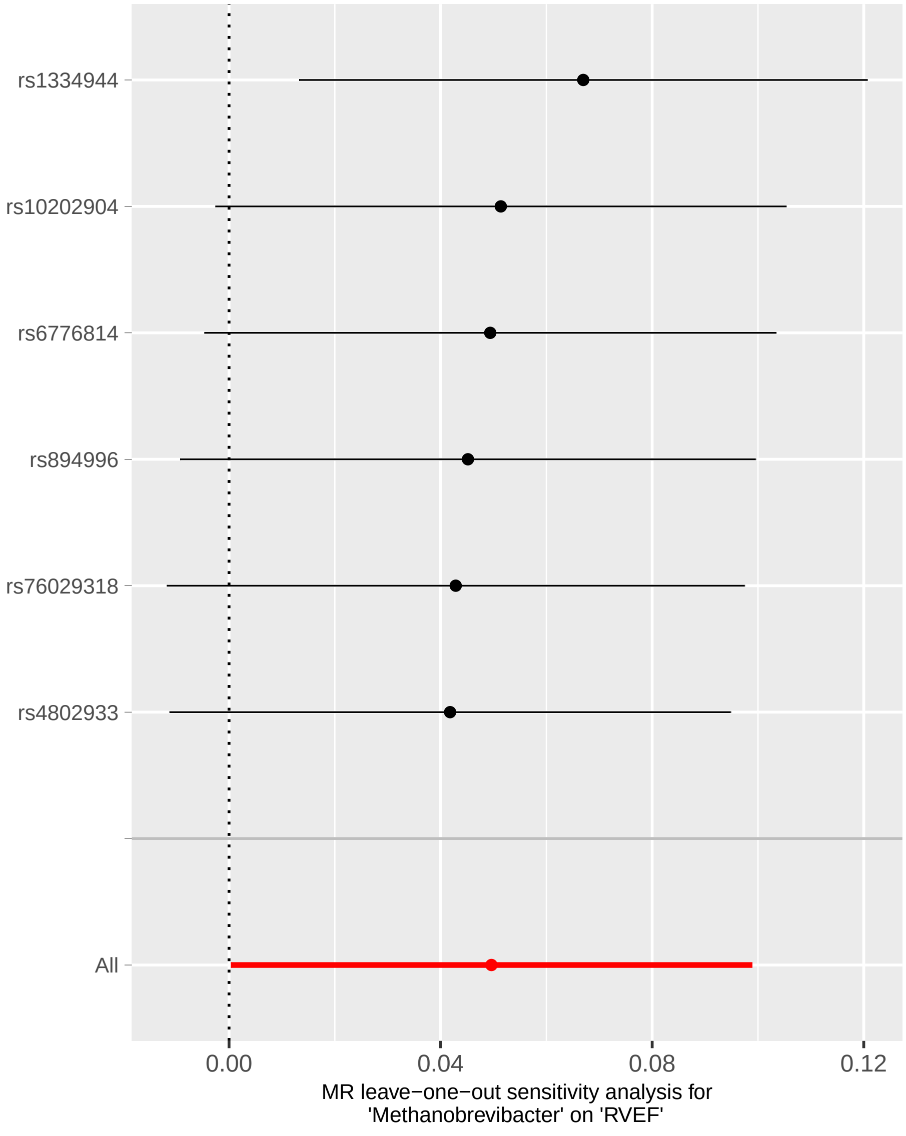
rs1334944
rs10202904
rs6776814
rs894996
rs76029318
rs4802933
All
0.00
0.04
0.08
0.12
MR leave−one−out sensitivity analysis for
'Methanobrevibacter' on 'RVEF'

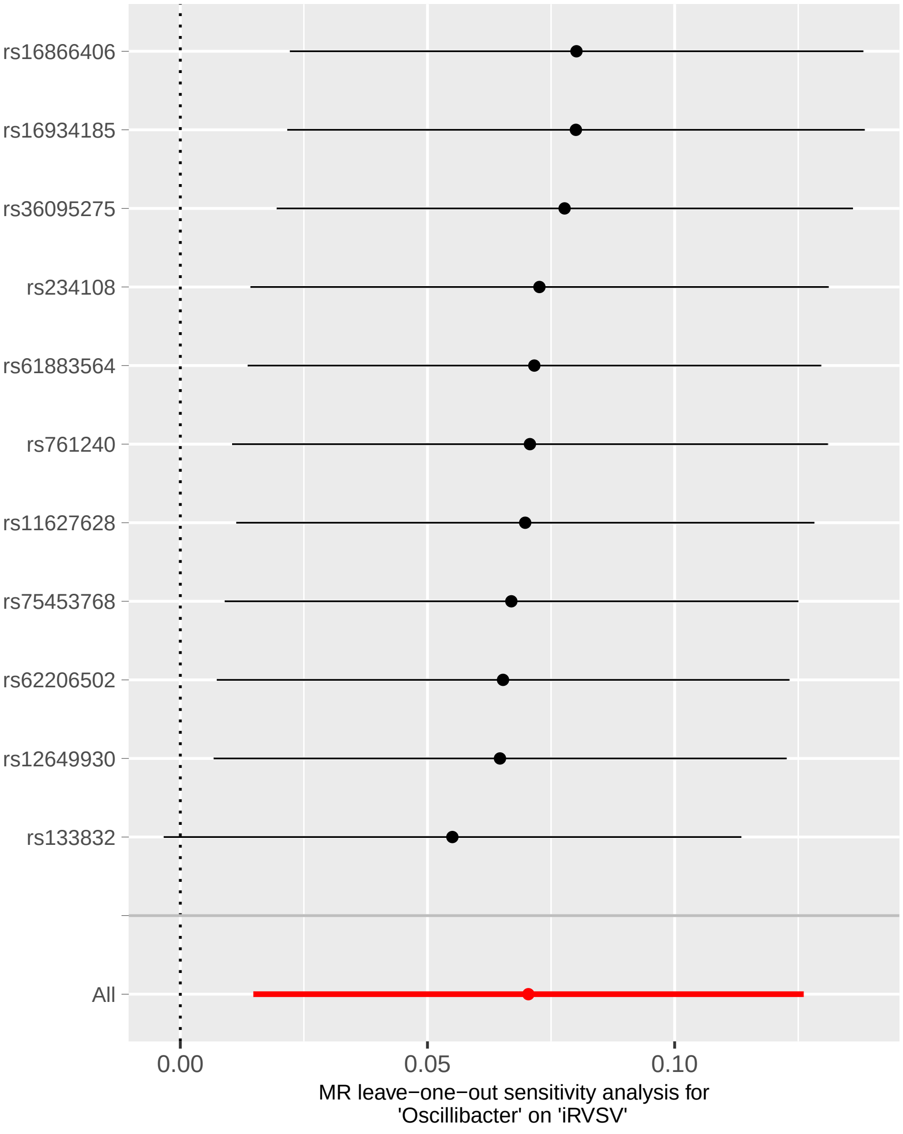
rs16866406
rs16934185
rs36095275
rs234108
rs61883564
rs761240
rs11627628
rs75453768
rs62206502
rs12649930
rs133832
All
0.00
0.05
0.10
MR leave−one−out sensitivity analysis for
'Oscillibacter' on 'iRVSV'

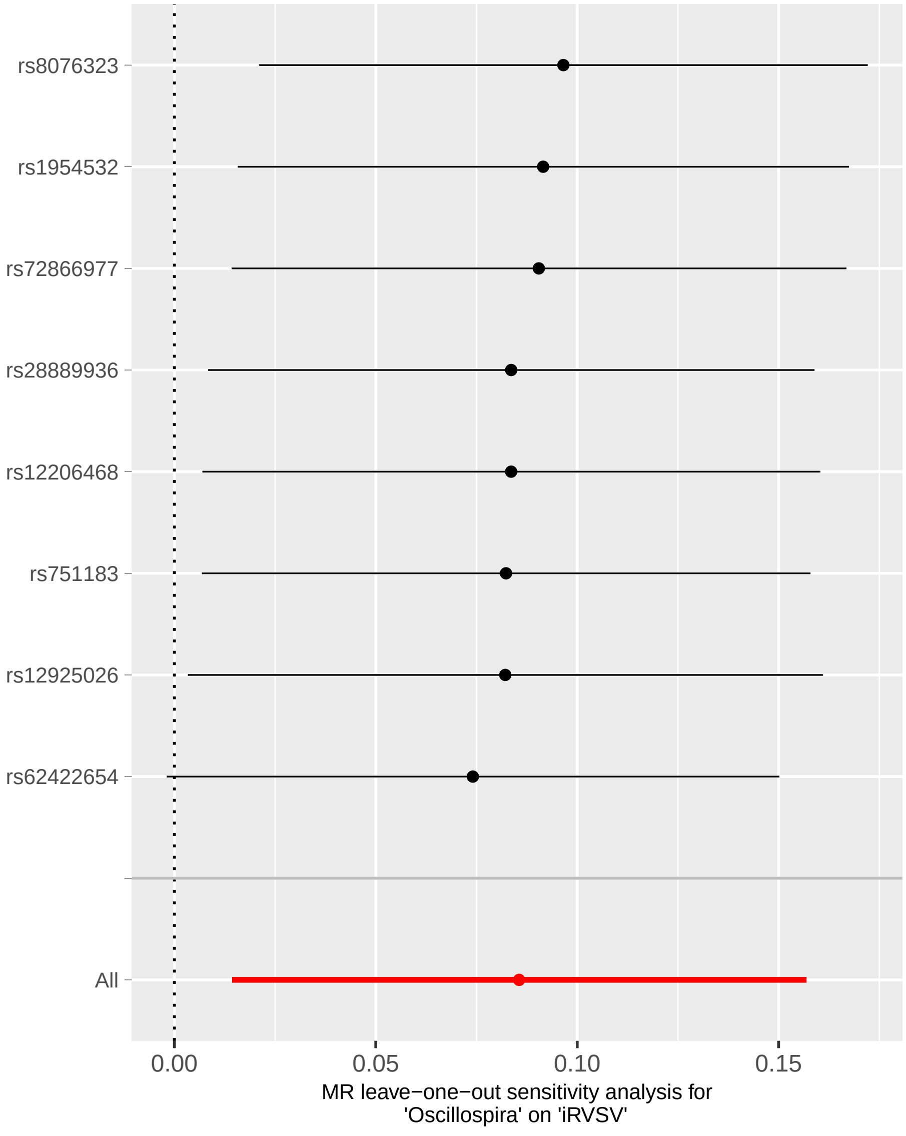

rs8076323
rs1954532
rs72866977
rs28889936
rs12206468
rs751183
rs12925026
rs62422654
All
0.00
0.05
0.10
0.15
MR leave−one−out sensitivity analysis for
'Oscillospira' on 'iRVSV'

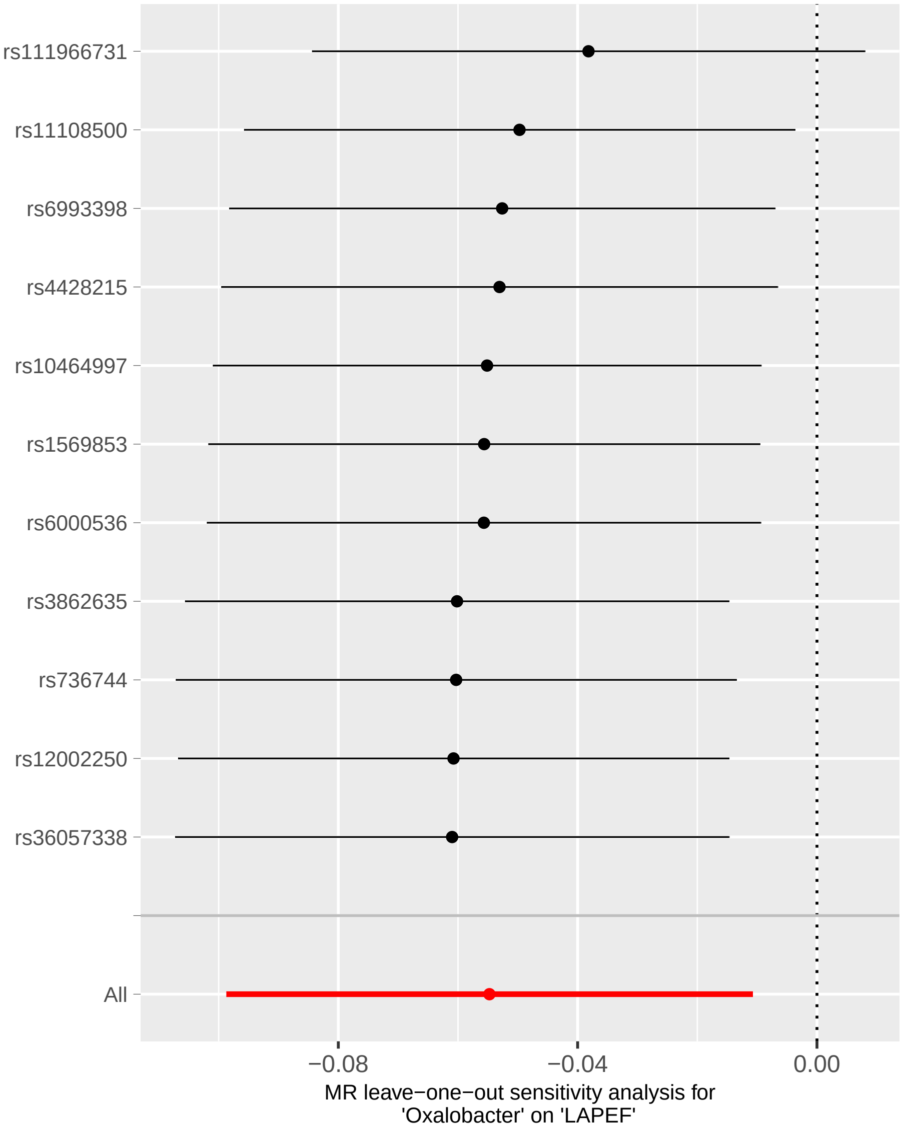
rs111966731
rs11108500
rs6993398
rs4428215
rs10464997
rs1569853
rs6000536
rs3862635
rs736744
rs12002250
rs36057338
All
−0.08
−0.04
0.00
MR leave−one−out sensitivity analysis for
'Oxalobacter' on 'LAPEF'

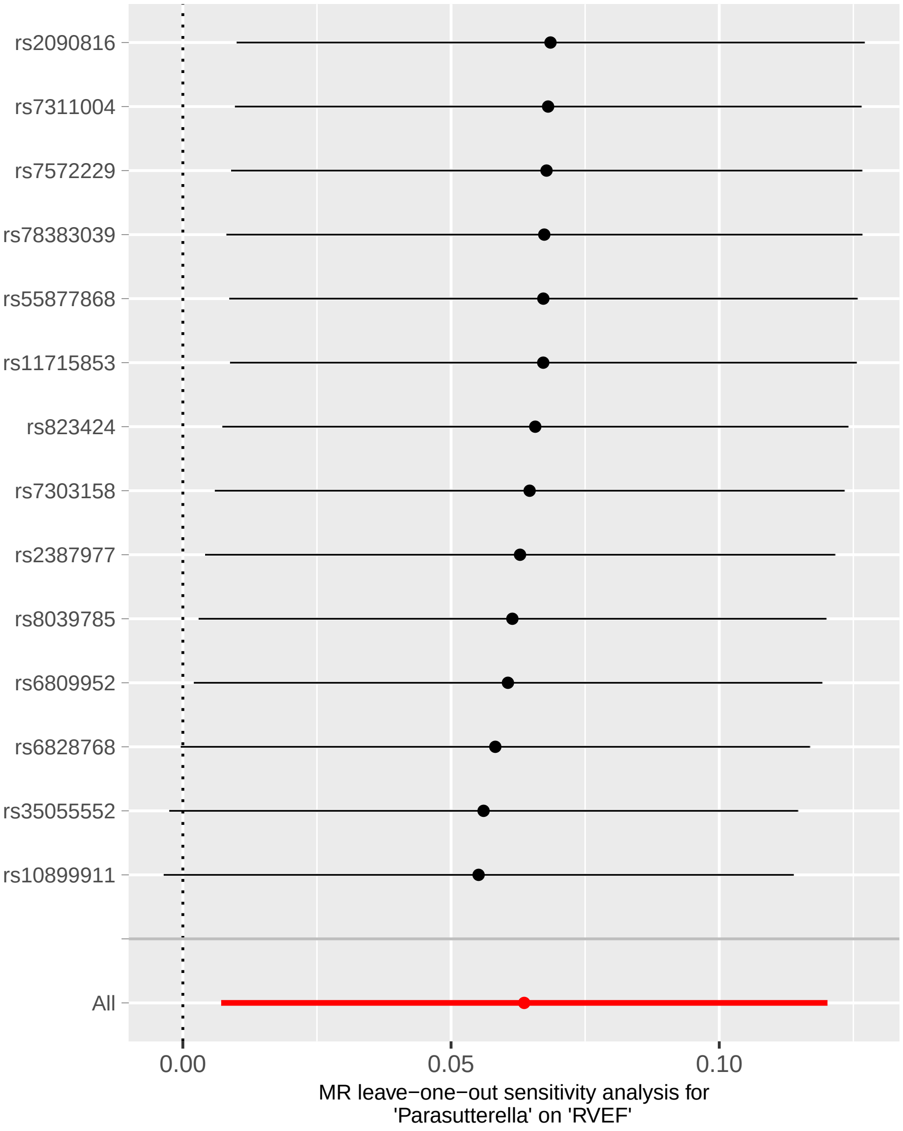
rs2090816
rs7311004
rs7572229
rs78383039
rs55877868
rs11715853
rs823424
rs7303158
rs2387977
rs8039785
rs6809952
rs6828768
rs35055552
rs10899911
All
0.00
0.05
0.10
MR leave−one−out sensitivity analysis for
'Parasutterella' on 'RVEF'

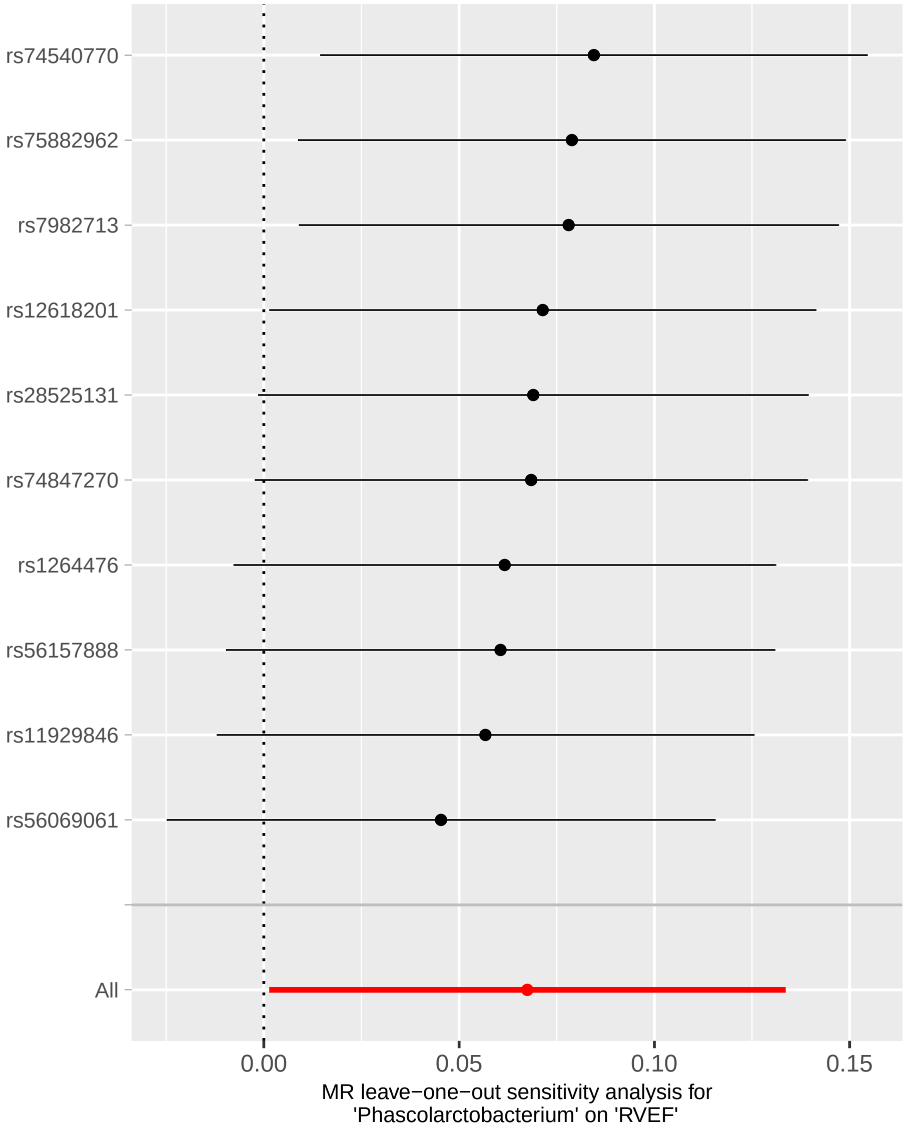
rs74540770
rs75882962
rs7982713
rs12618201
rs28525131
rs74847270
rs1264476
rs56157888
rs11929846
rs56069061
All
0.00
0.05
0.10
0.15
MR leave−one−out sensitivity analysis for
'Phascolarctobacterium' on 'RVEF'

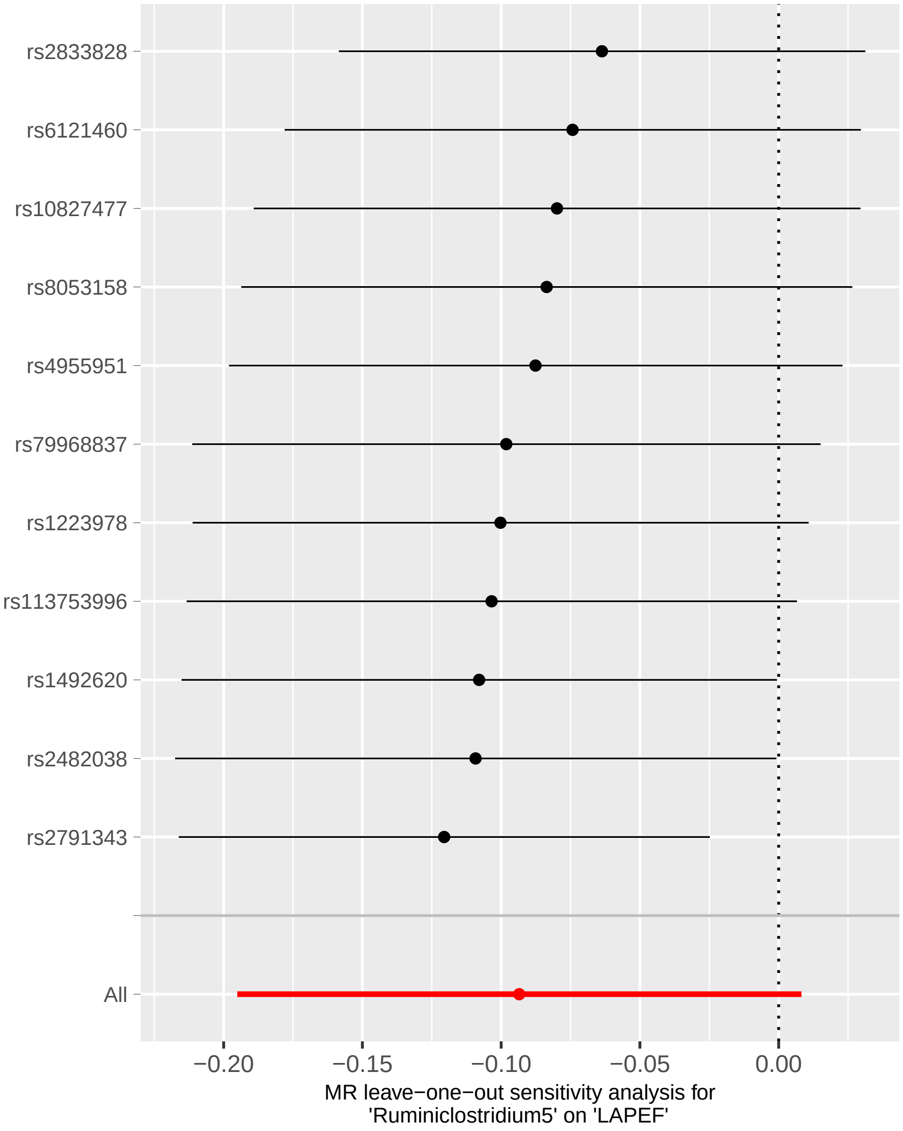
rs2833828
rs6121460
rs10827477
rs8053158
rs4955951
rs79968837
rs1223978
rs113753996
rs1492620
rs2482038
rs2791343
All
−0.20
−0.15
−0.10
−0.05
0.00
MR leave−one−out sensitivity analysis for
'Ruminiclostridium5' on 'LAPEF'

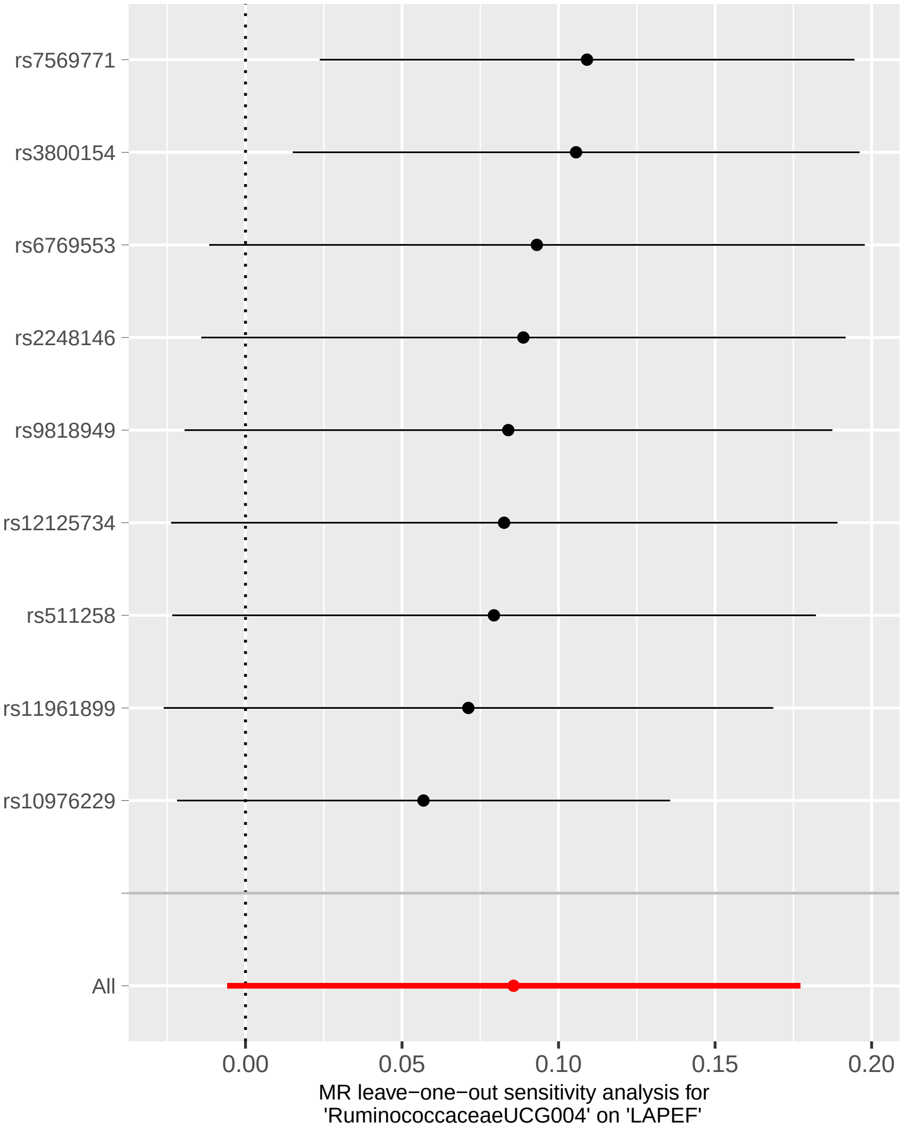
rs7569771
rs3800154
rs6769553
rs2248146
rs9818949
rs12125734
rs511258
rs11961899
rs10976229
All
0.00
0.05
0.10
0.15
0.20
MR leave−one−out sensitivity analysis for
'RuminococcaceaeUCG004' on 'LAPEF'

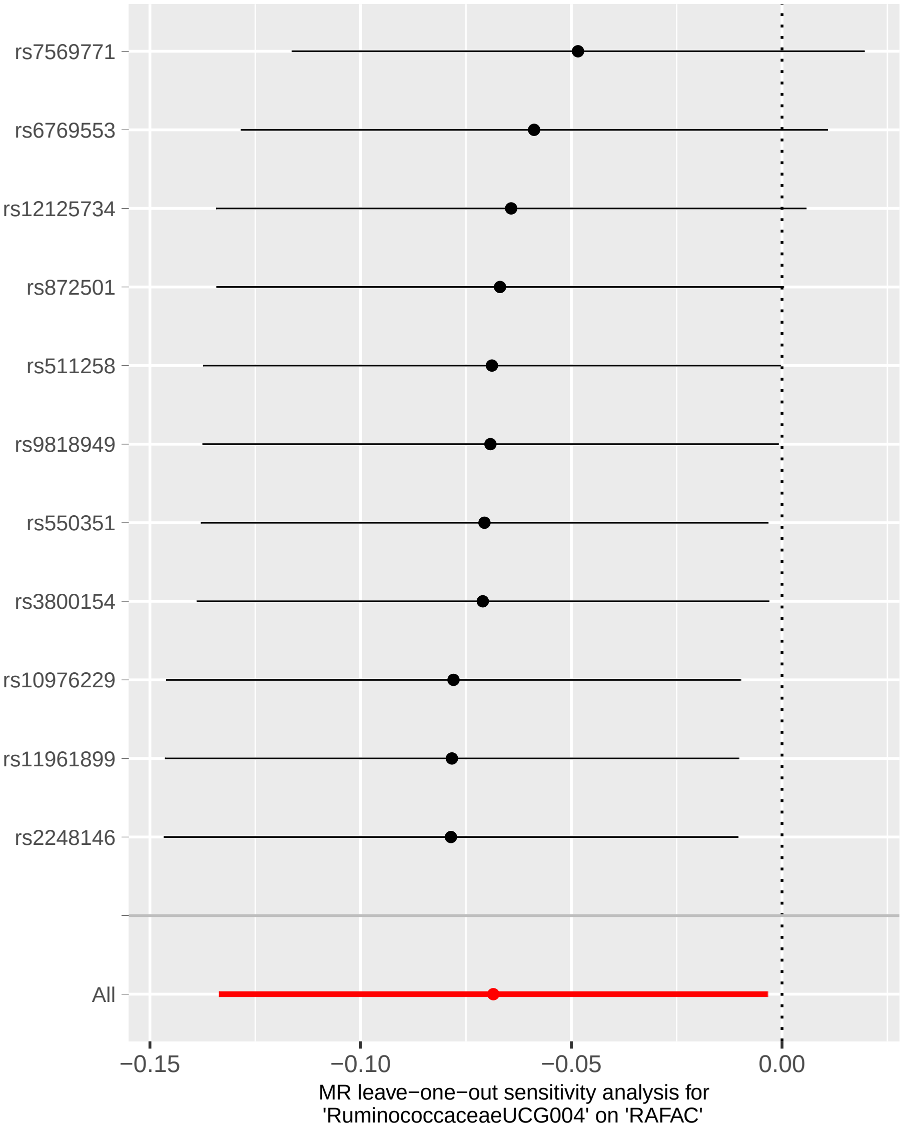
rs7569771
rs6769553
rs12125734
rs872501
rs511258
rs9818949
rs550351
rs3800154
rs10976229
rs11961899
rs2248146
All
−0.15
−0.10
−0.05
0.00
MR leave−one−out sensitivity analysis for
'RuminococcaceaeUCG004' on 'RAFAC'

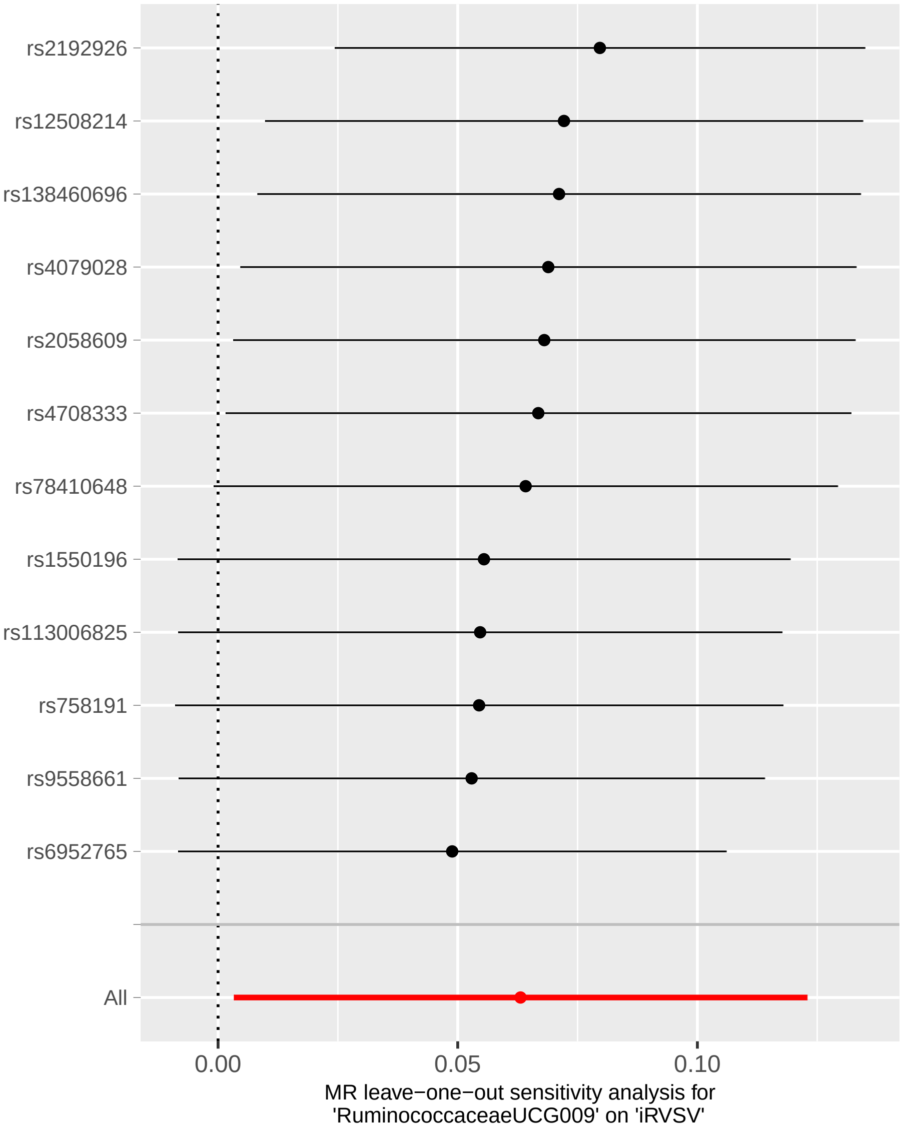
rs2192926
rs12508214
rs138460696
rs4079028
rs2058609
rs4708333
rs78410648
rs1550196
rs113006825
rs758191
rs9558661
rs6952765
All
0.00
0.05
0.10
MR leave−one−out sensitivity analysis for
'RuminococcaceaeUCG009' on 'iRVSV'

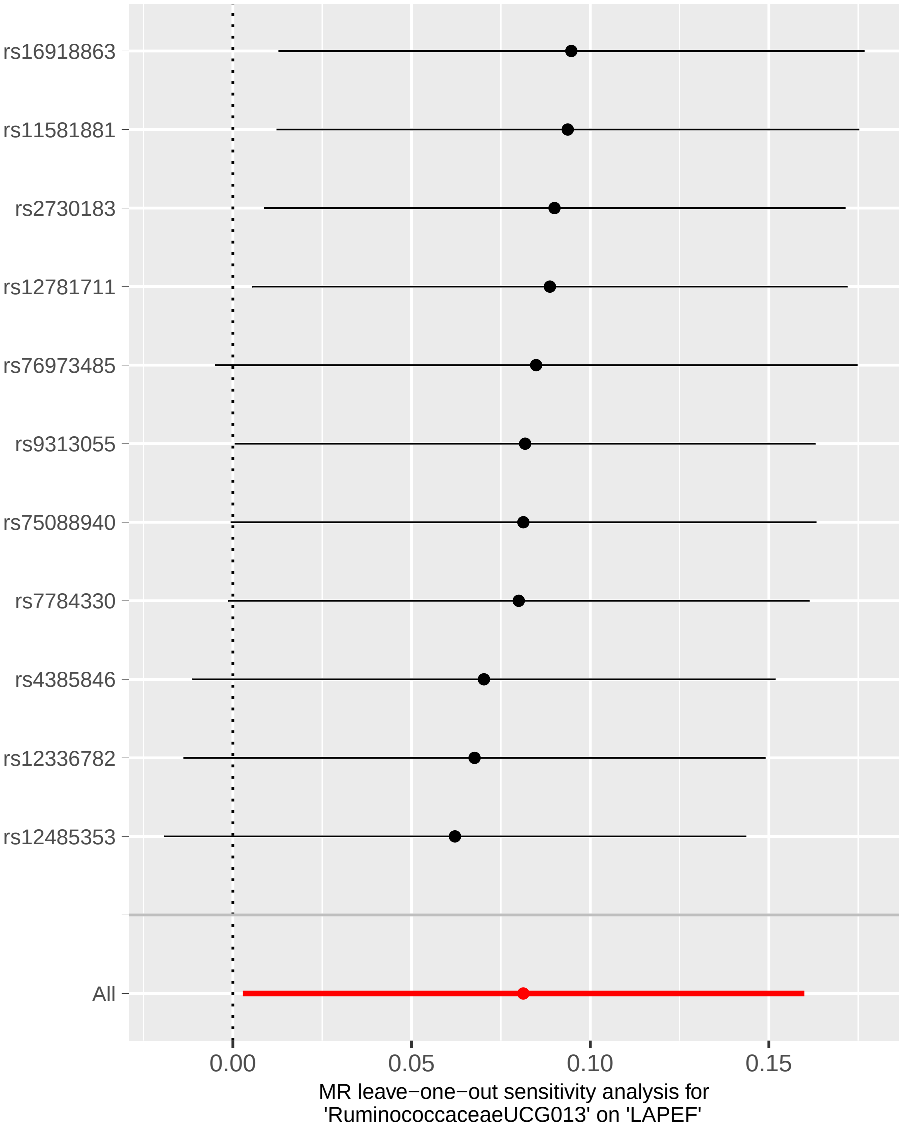

rs16918863
rs11581881
rs2730183
rs12781711
rs76973485
rs9313055
rs75088940
rs7784330
rs4385846
rs12336782
rs12485353
All
0.00
0.05
0.10
0.15
MR leave−one−out sensitivity analysis for
'RuminococcaceaeUCG013' on 'LAPEF'

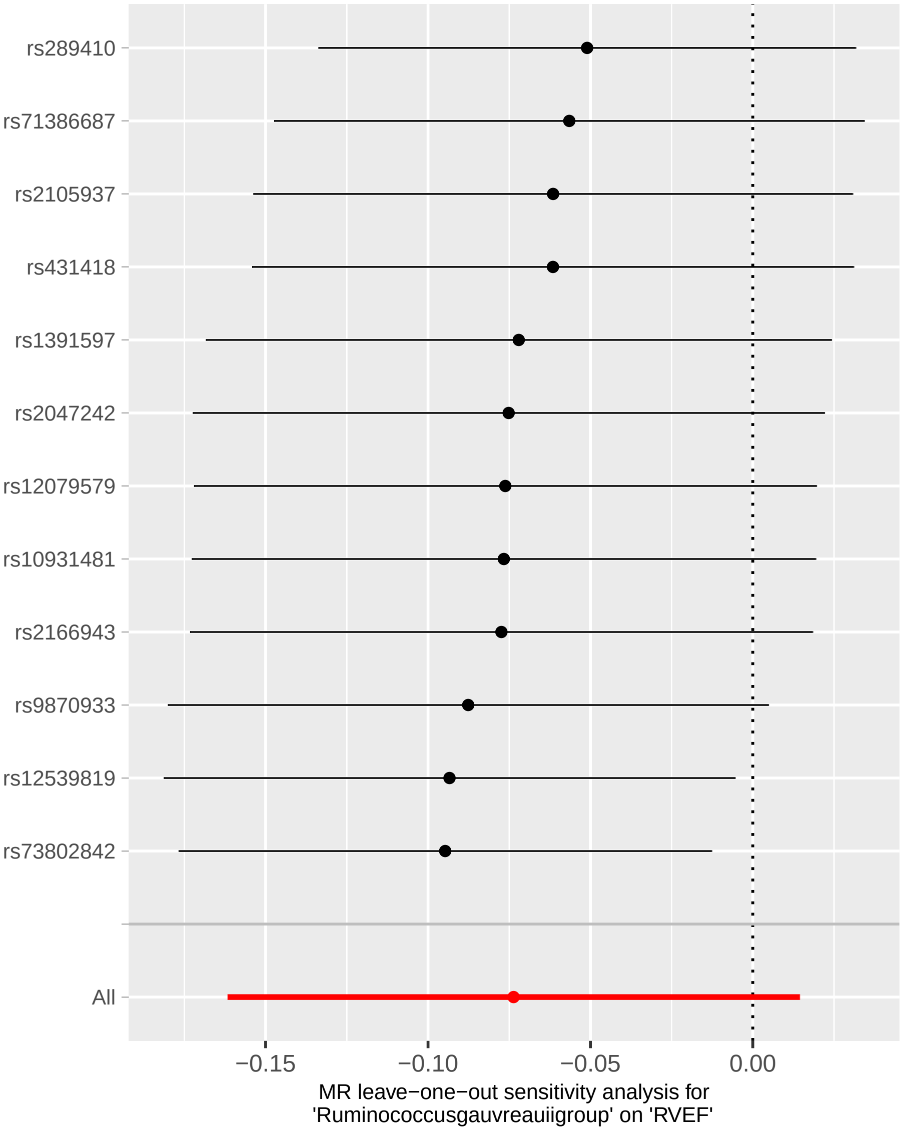

rs289410
rs71386687
rs2105937
rs431418
rs1391597
rs2047242
rs12079579
rs10931481
rs2166943
rs9870933
rs12539819
rs73802842
All
−0.15
−0.10
−0.05
0.00
MR leave−one−out sensitivity analysis for
'Ruminococcusgauvreauiigroup' on 'RVEF'

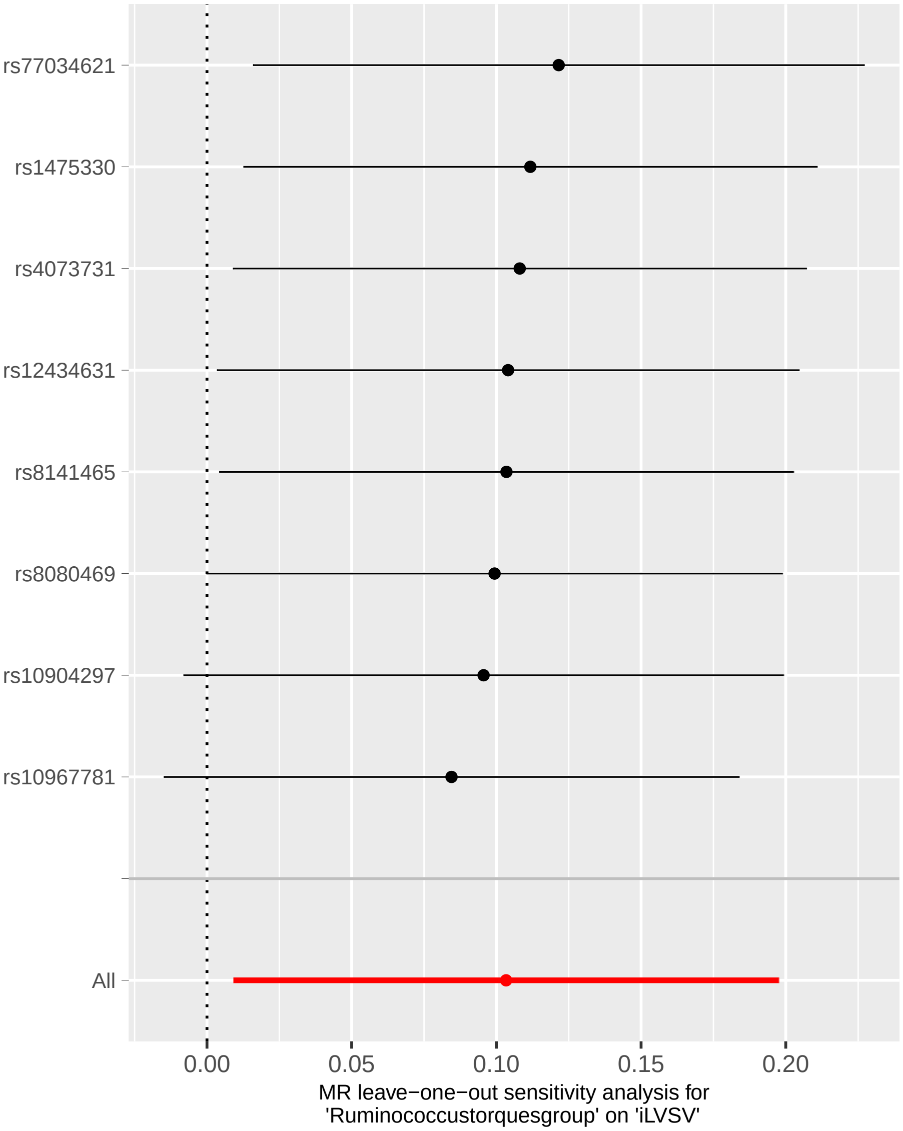
rs77034621
rs1475330
rs4073731
rs12434631
rs8141465
rs8080469
rs10904297
rs10967781
All
0.00
0.05
0.10
0.15
0.20
MR leave−one−out sensitivity analysis for
'Ruminococcustorquesgroup' on 'iLVSV'

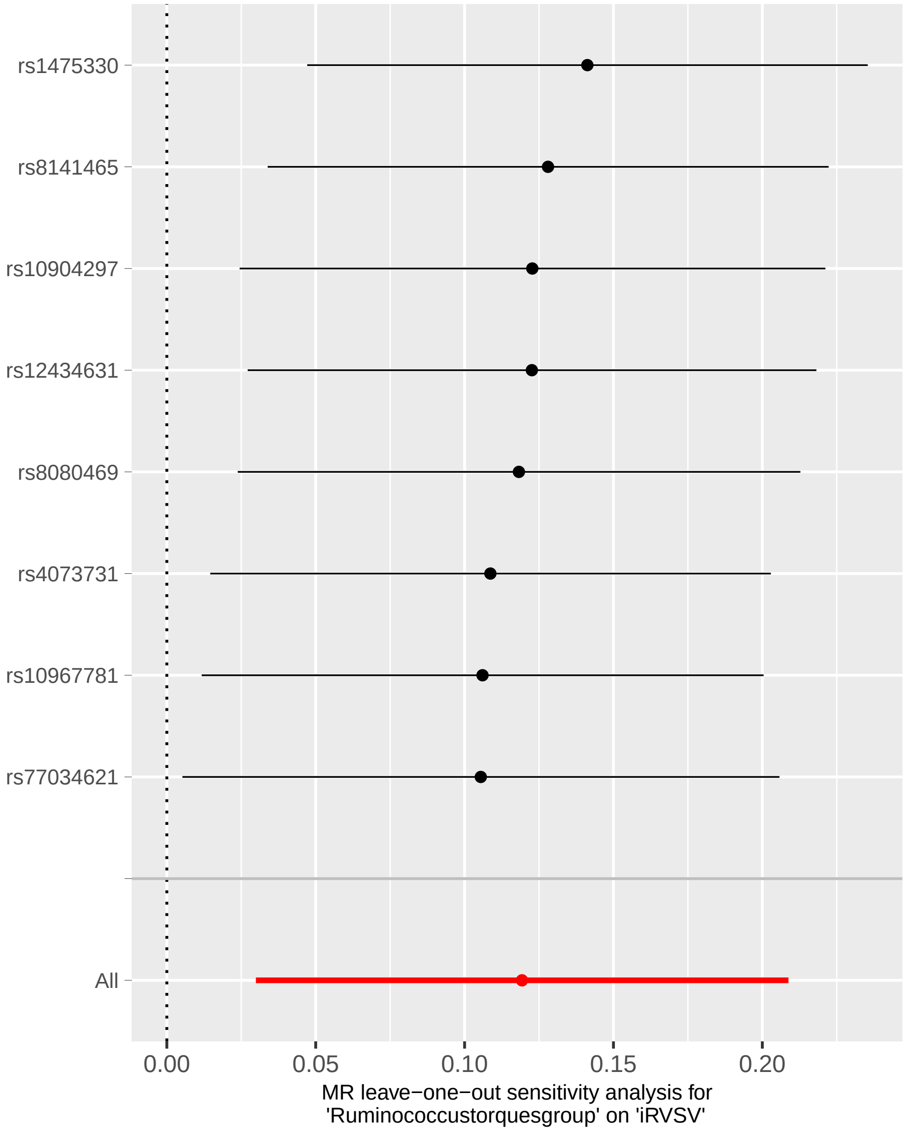
rs1475330
rs8141465
rs10904297
rs12434631
rs8080469
rs4073731
rs10967781
rs77034621
All
0.00
0.05
0.10
0.15
0.20
MR leave−one−out sensitivity analysis for
'Ruminococcustorquesgroup' on 'iRVSV'

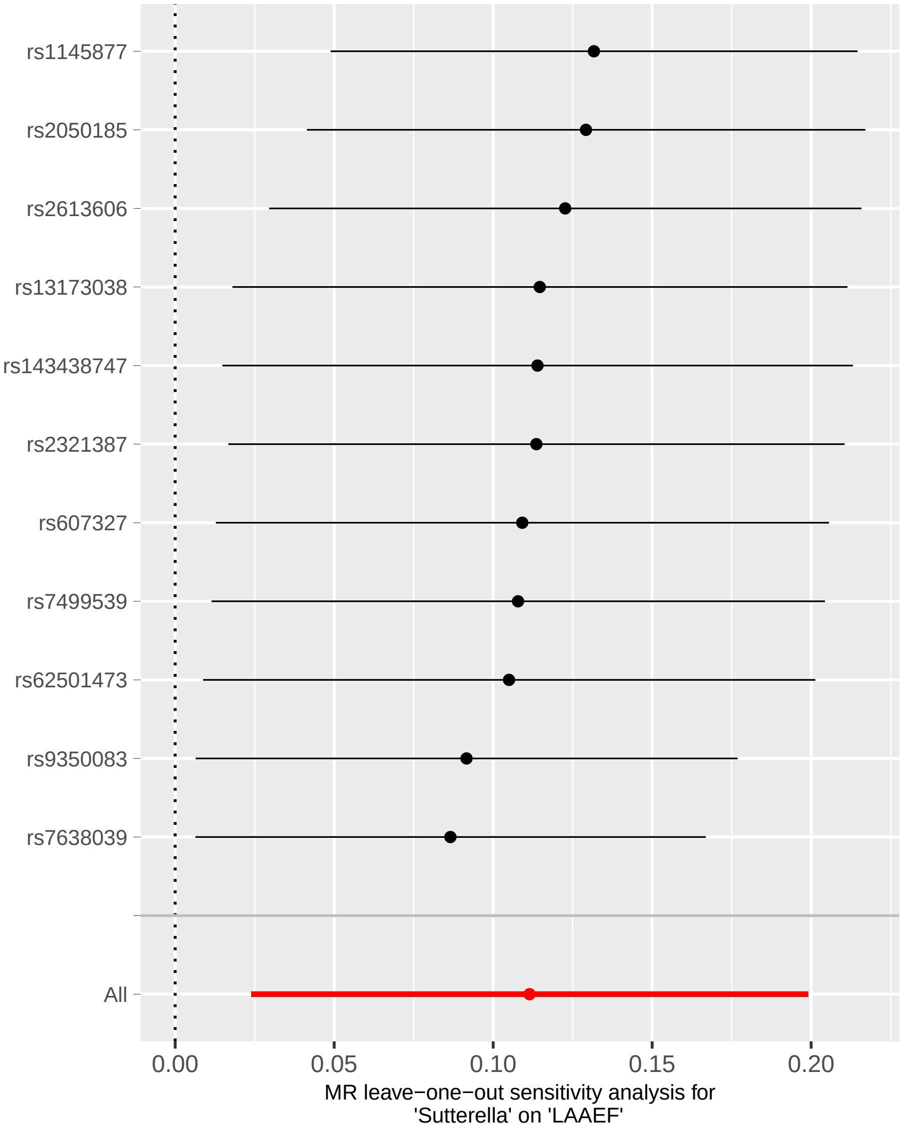
rs1145877
rs2050185
rs2613606
rs13173038
rs143438747
rs2321387
rs607327
rs7499539
rs62501473
rs9350083
rs7638039
All
0.00
0.05
0.10
0.15
0.20
MR leave−one−out sensitivity analysis for
'Sutterella' on 'LAAEF'

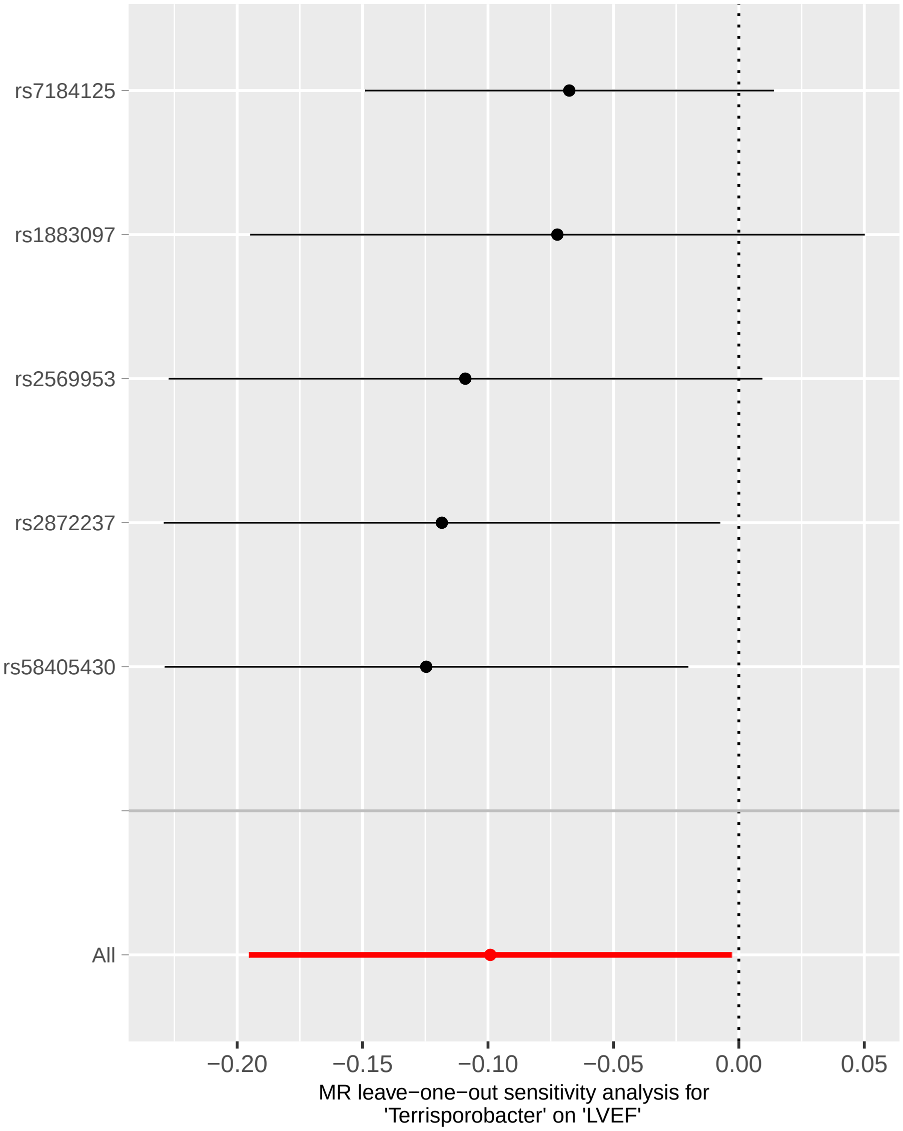
rs7184125
rs1883097
rs2569953
rs2872237
rs58405430
All
−0.20
−0.15
−0.10
−0.05
0.00
0.05
MR leave−one−out sensitivity analysis for
'Terrisporobacter' on 'LVEF'
