# Supplementary material for: Gut microbiota as a residual risk factor causally influencing cardiac structure and function: Mendelian randomization analysis and biological annotation
Source: Front Microbiol. 2024 Jul 26;15:1410272. doi: 10.3389/fmicb.2024.1410272 (PMC11316272; doi:10.3389/fmicb.2024.1410272)
Supplement: SUPPLEMENTARY FIGURE S3 — Scatter plots for gut microbiota on cardiac structure [file Data_Sheet_3.PDF]

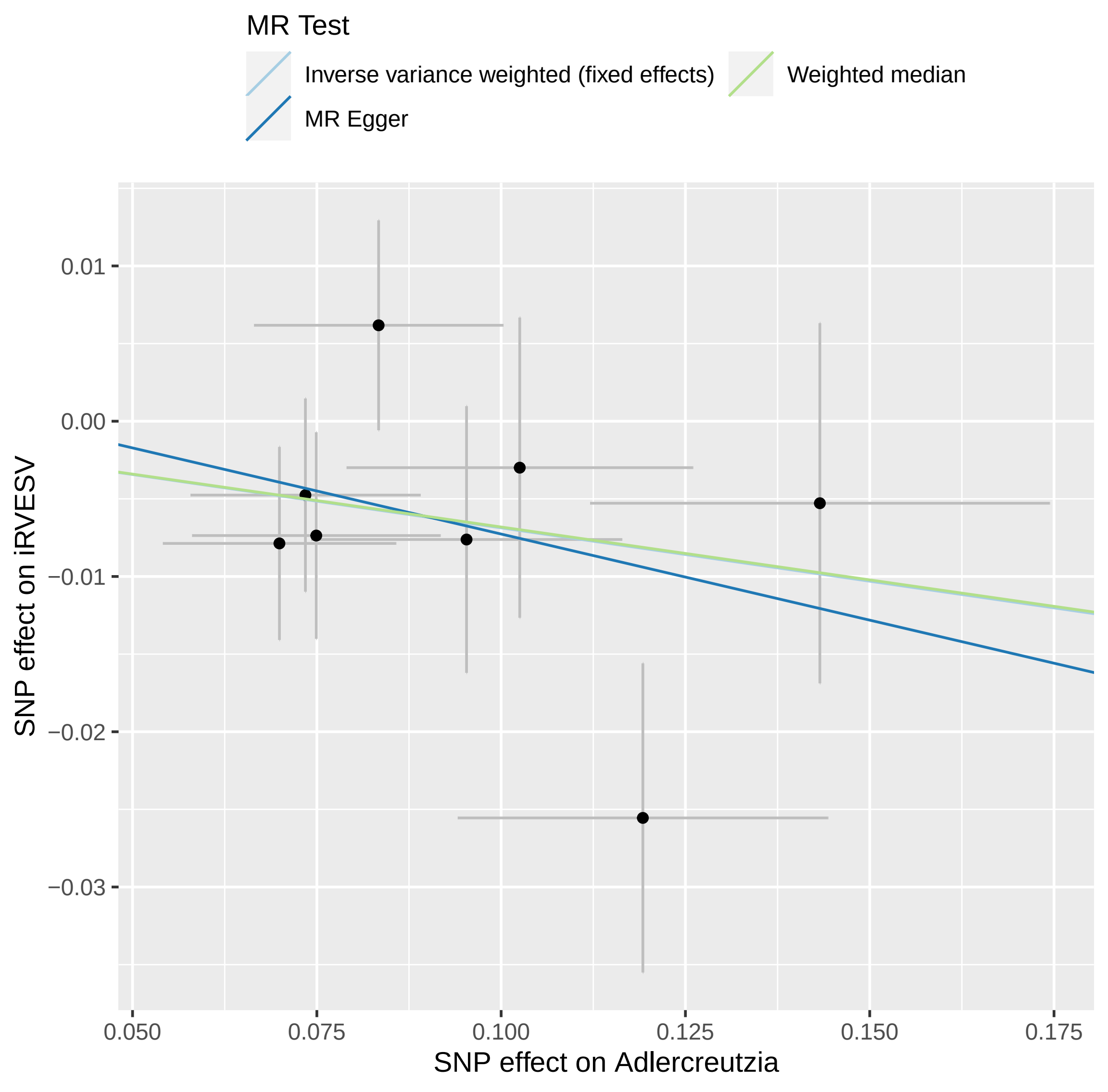
MR Test
Inverse variance weighted (fixed effects)
Weighted median
MR Egger
SNP effect on iRVESV
SNP effect on Adlercreutzia
0.01
0.00
−0.01
−0.02
−0.03
0.050
0.075
0.100
0.125
0.150
0.175

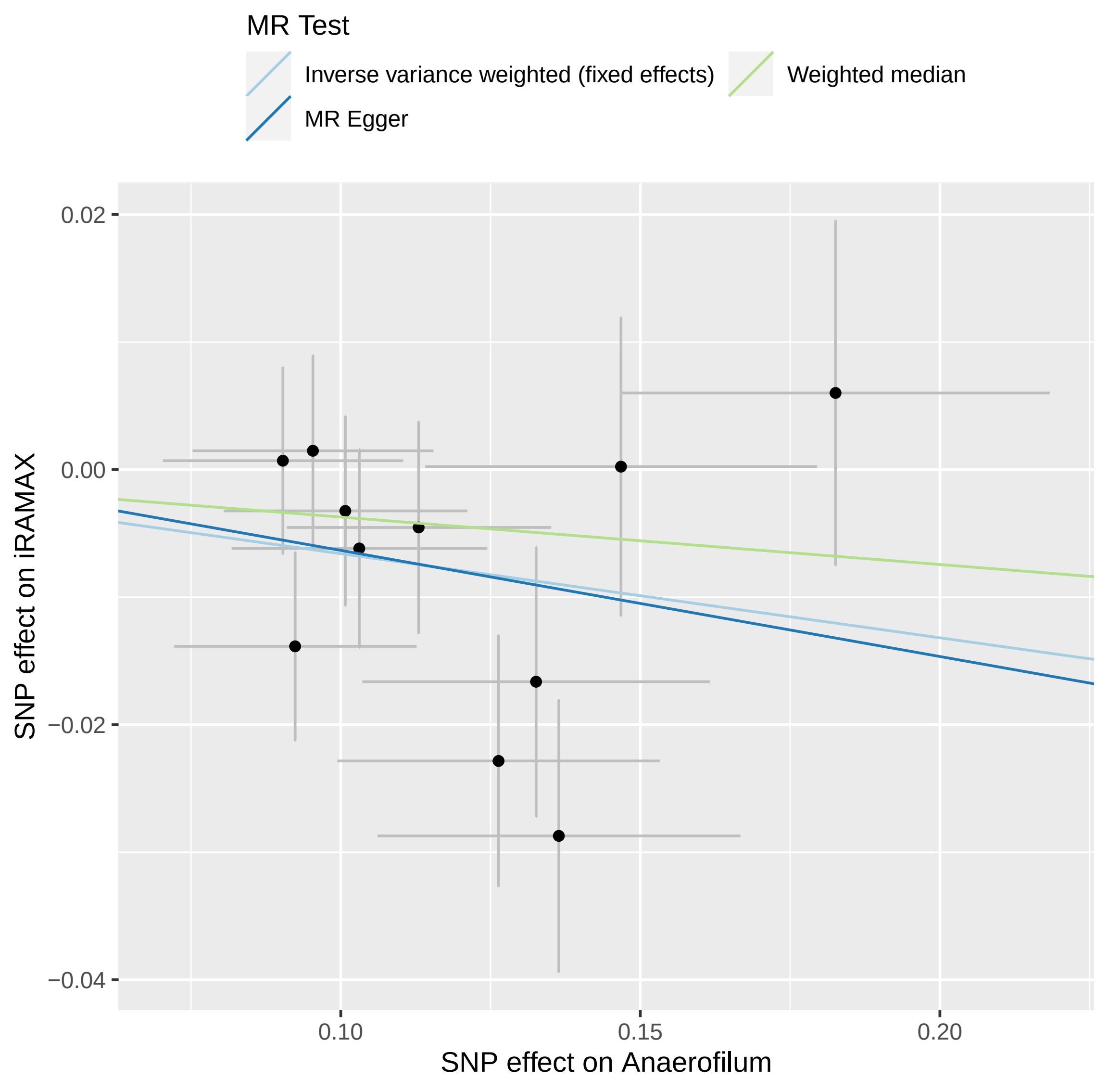
MR Test
Inverse variance weighted (fixed effects)
Weighted median
MR Egger
SNP effect on iRAMAX
0.02
0.00
−0.02
−0.04
SNP effect on Anaerofilum
0.10
0.15
0.20

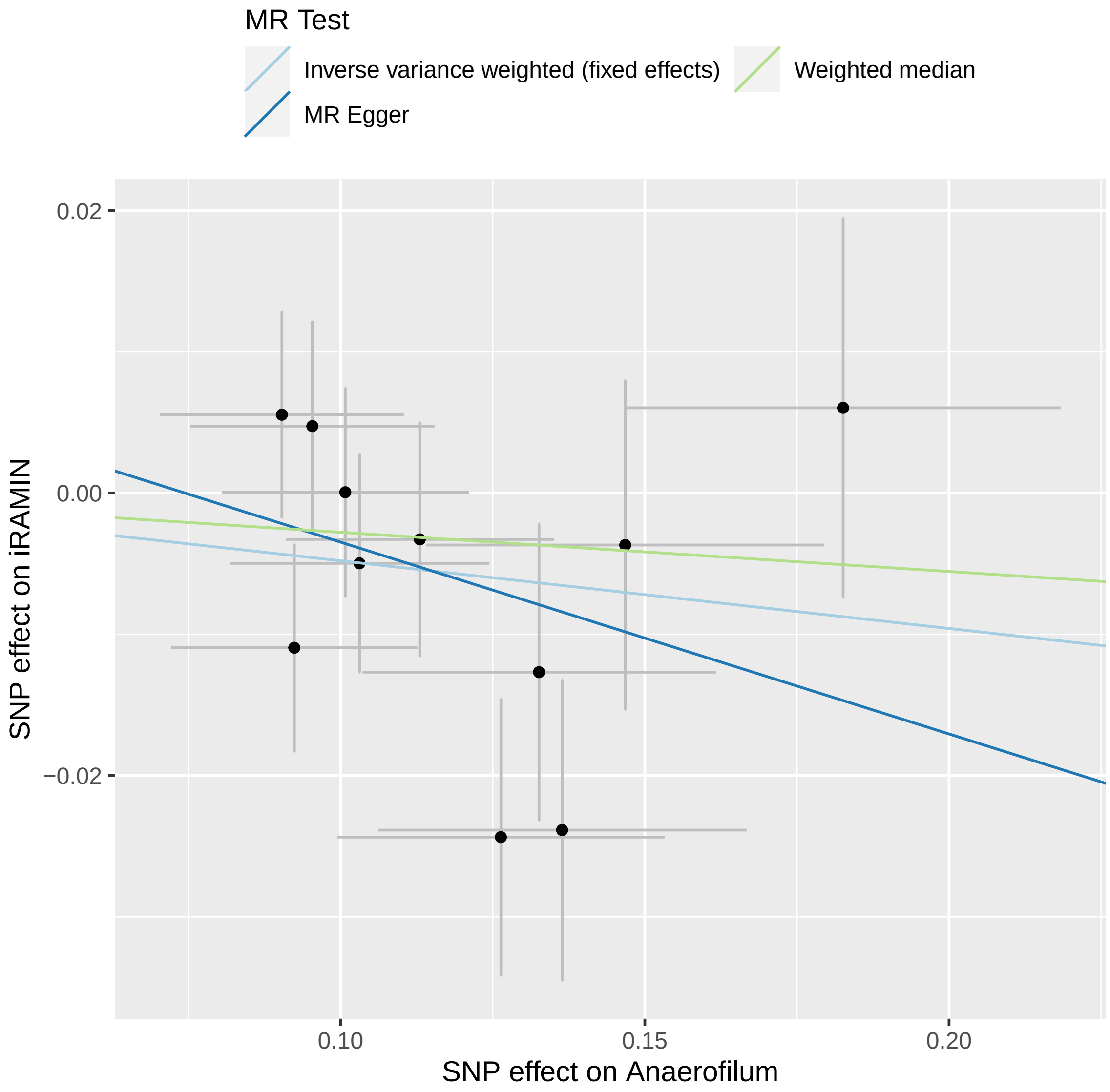
MR Test
Inverse variance weighted (fixed effects)
Weighted median
MR Egger
0.02
0.00
−0.02
0.10
0.15
0.20
SNP effect on iRAMIN
SNP effect on Anaerofilum

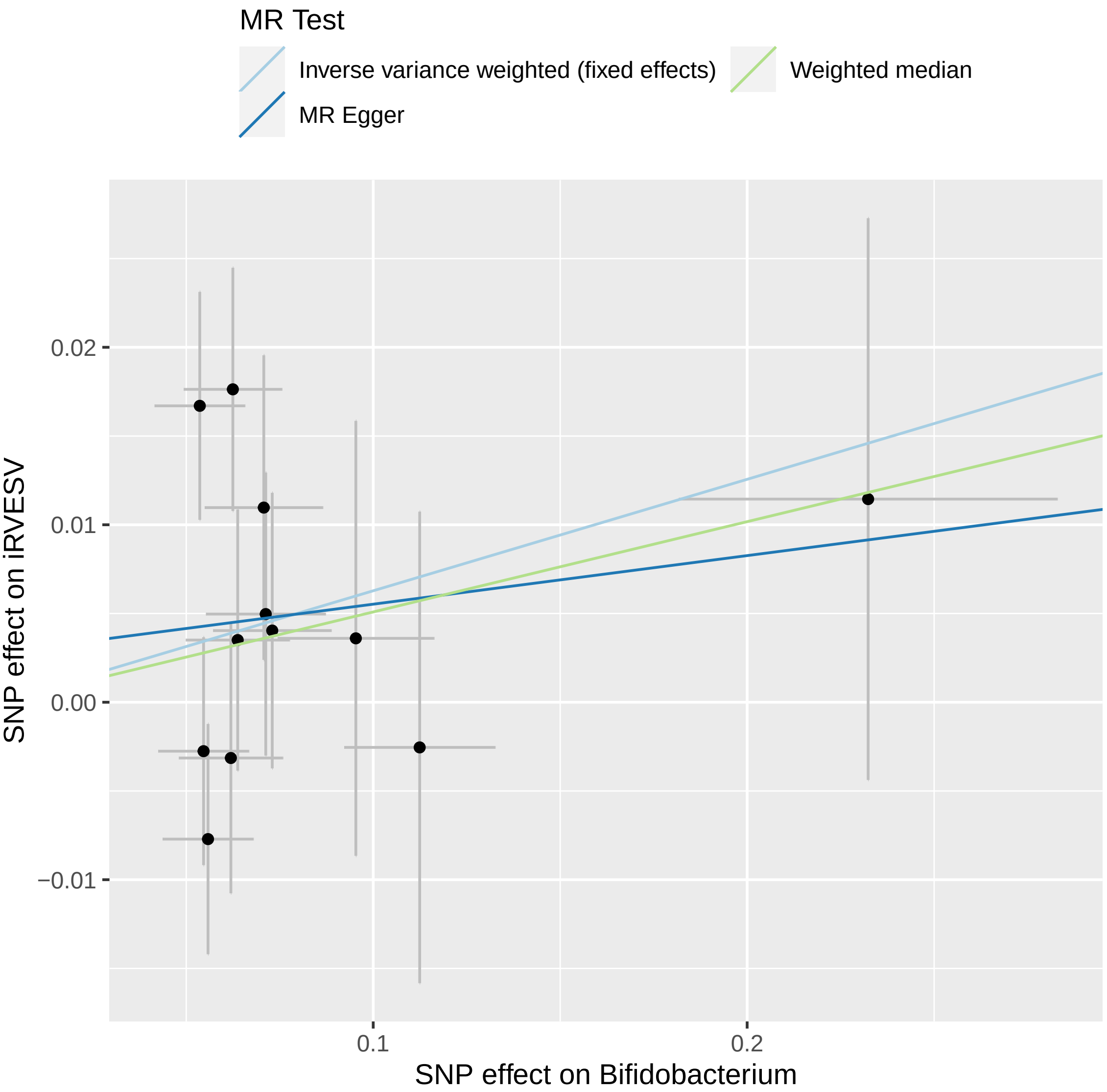

MR Test
Inverse variance weighted (fixed effects)
Weighted median
MR Egger
0.02
0.01
0.00
−0.01
0.1
0.2
SNP effect on iRVESV
SNP effect on Bifidobacterium

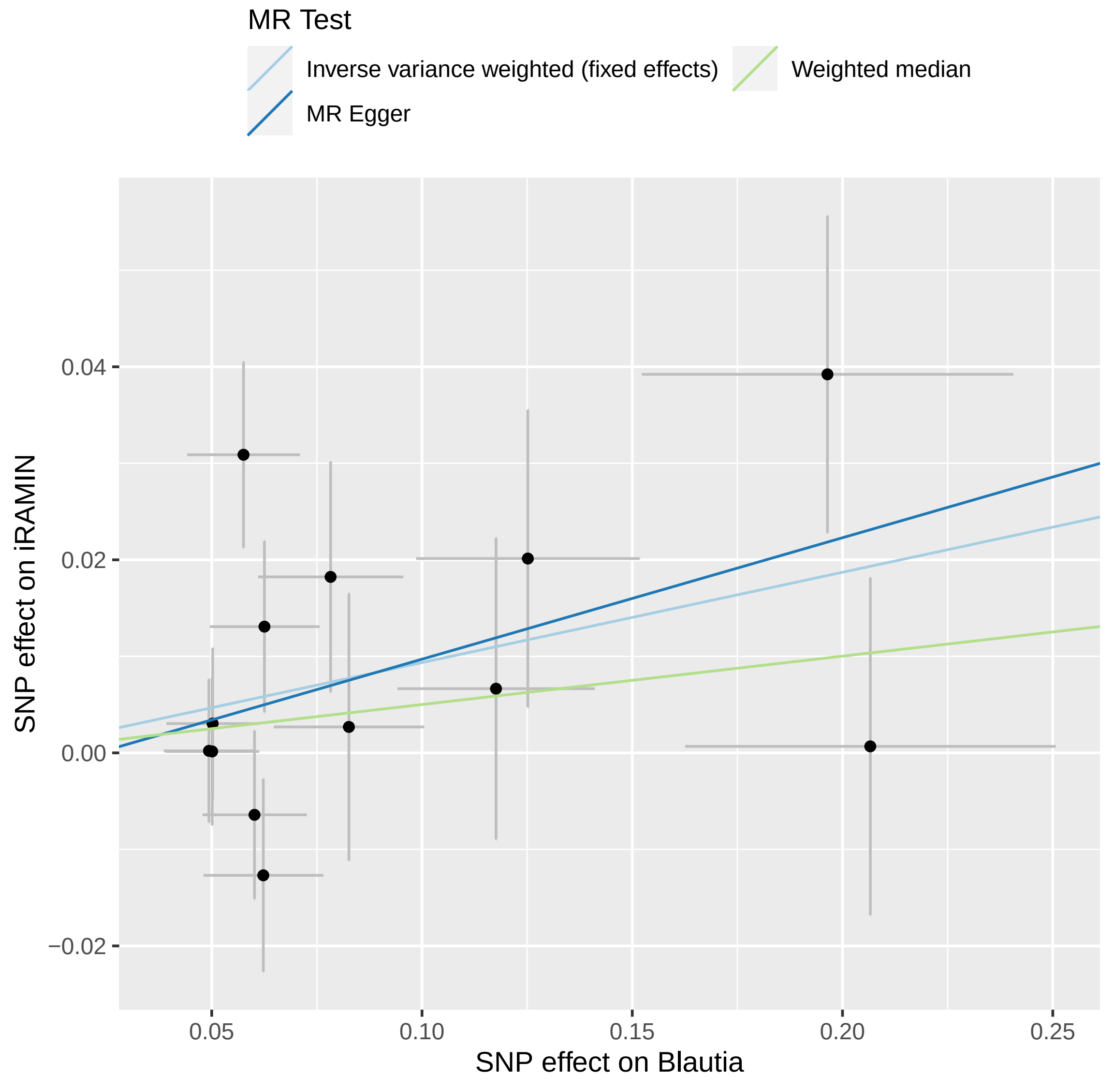

MR Test
Inverse variance weighted (fixed effects)
Weighted median
MR Egger
0.04
0.02
0.00
−0.02
SNP effect on iRAMIN
0.05
0.10
0.15
0.20
0.25
SNP effect on Blautia

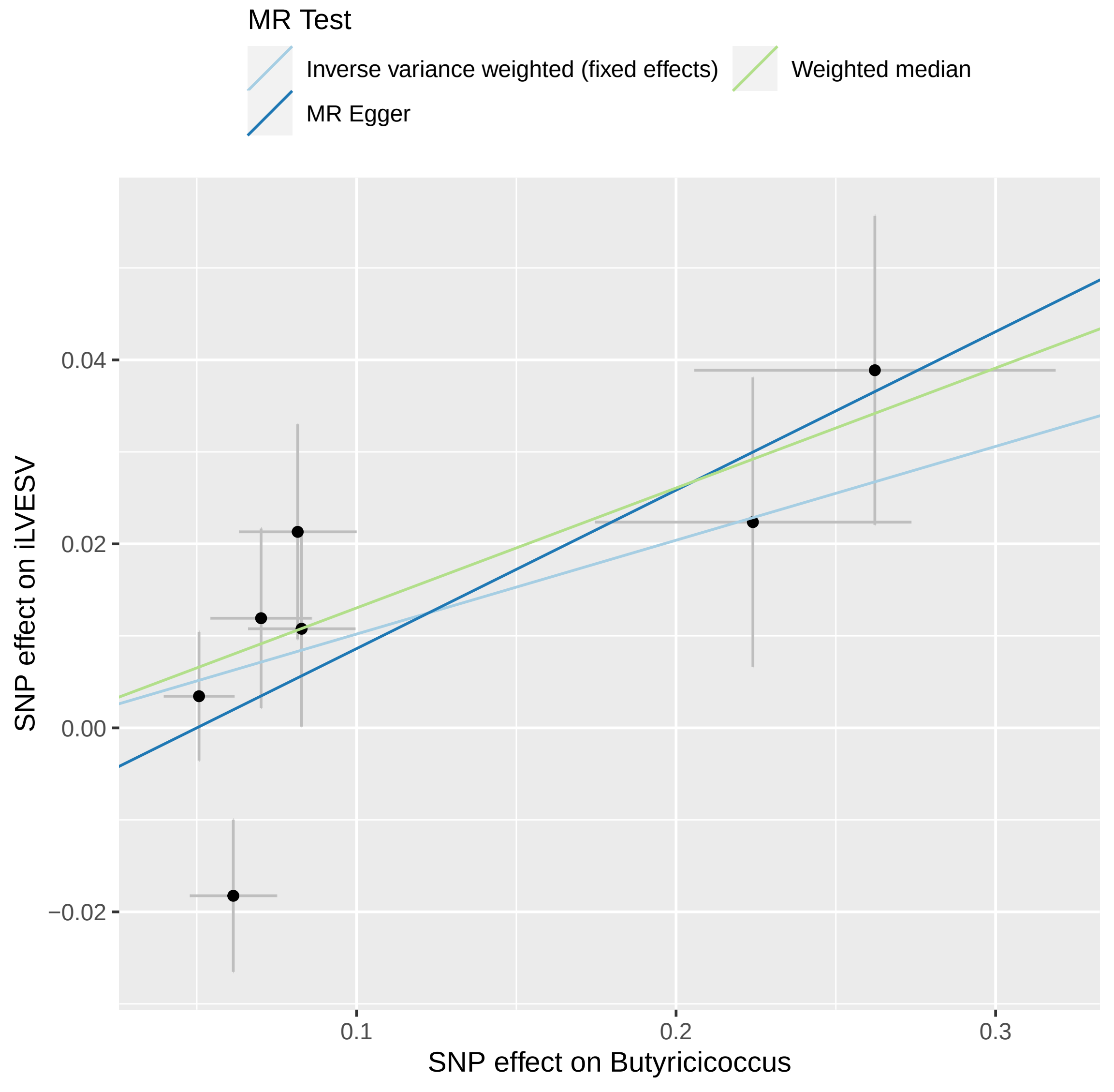
MR Test
Inverse variance weighted (fixed effects)
Weighted median
MR Egger
SNP effect on iLVESV
0.04
0.02
0.00
−0.02
0.1
0.2
0.3
SNP effect on Butyricicoccus

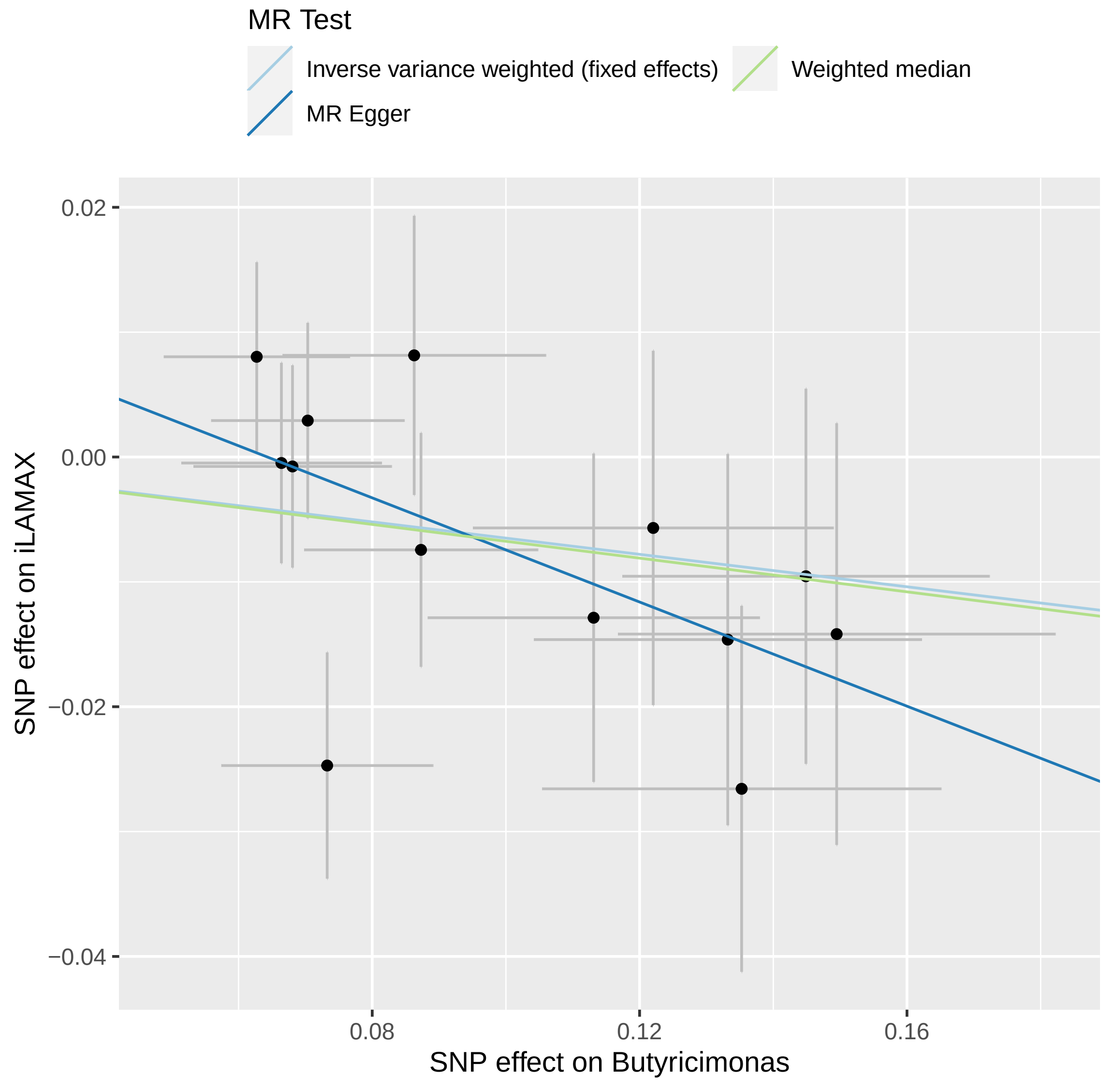
MR Test
Inverse variance weighted (fixed effects)
Weighted median
MR Egger
0.02
0.00
−0.02
−0.04
0.08
0.12
0.16
SNP effect on iLAMAX
SNP effect on Butyricimonas

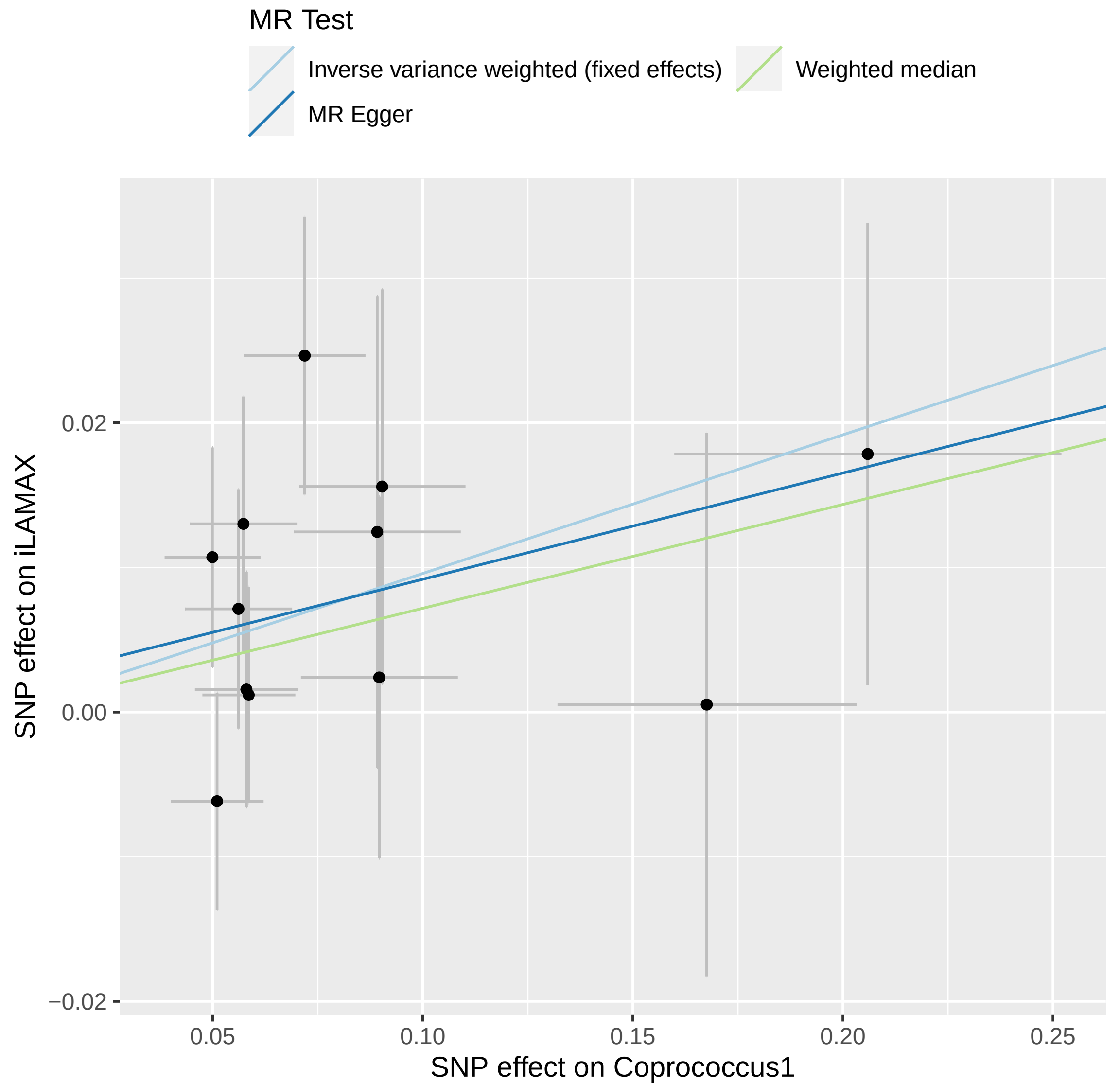
MR Test
Inverse variance weighted (fixed effects)
Weighted median
MR Egger
SNP effect on iLAMAX
0.02
0.00
−0.02
0.05
0.10
0.15
0.20
0.25
SNP effect on Coprococcus1

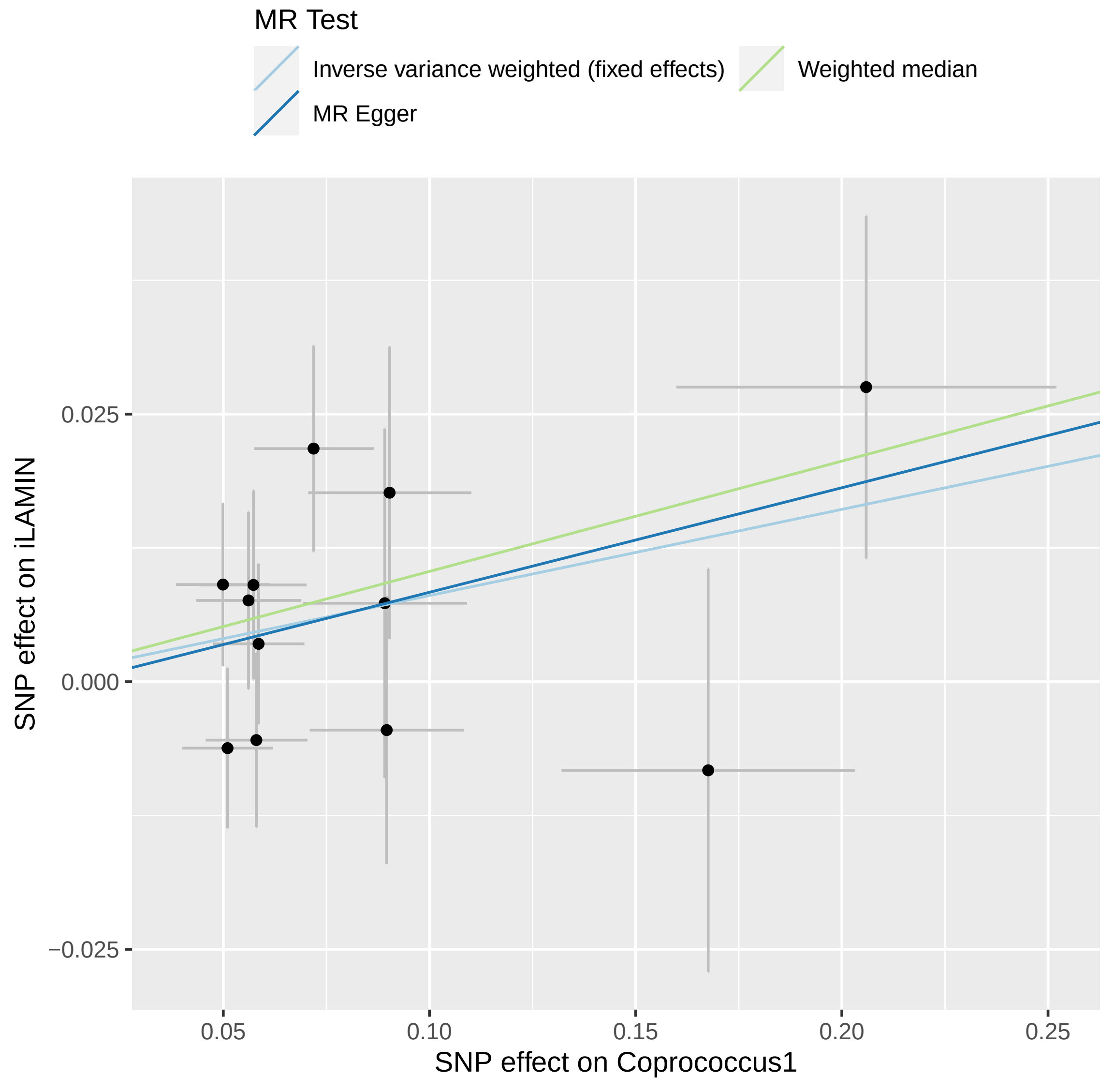
MR Test
Inverse variance weighted (fixed effects)
Weighted median
MR Egger
0.025
0.000
−0.025
0.05
0.10
0.15
0.20
0.25
SNP effect on Coprococcus1
SNP effect on iLAMIN

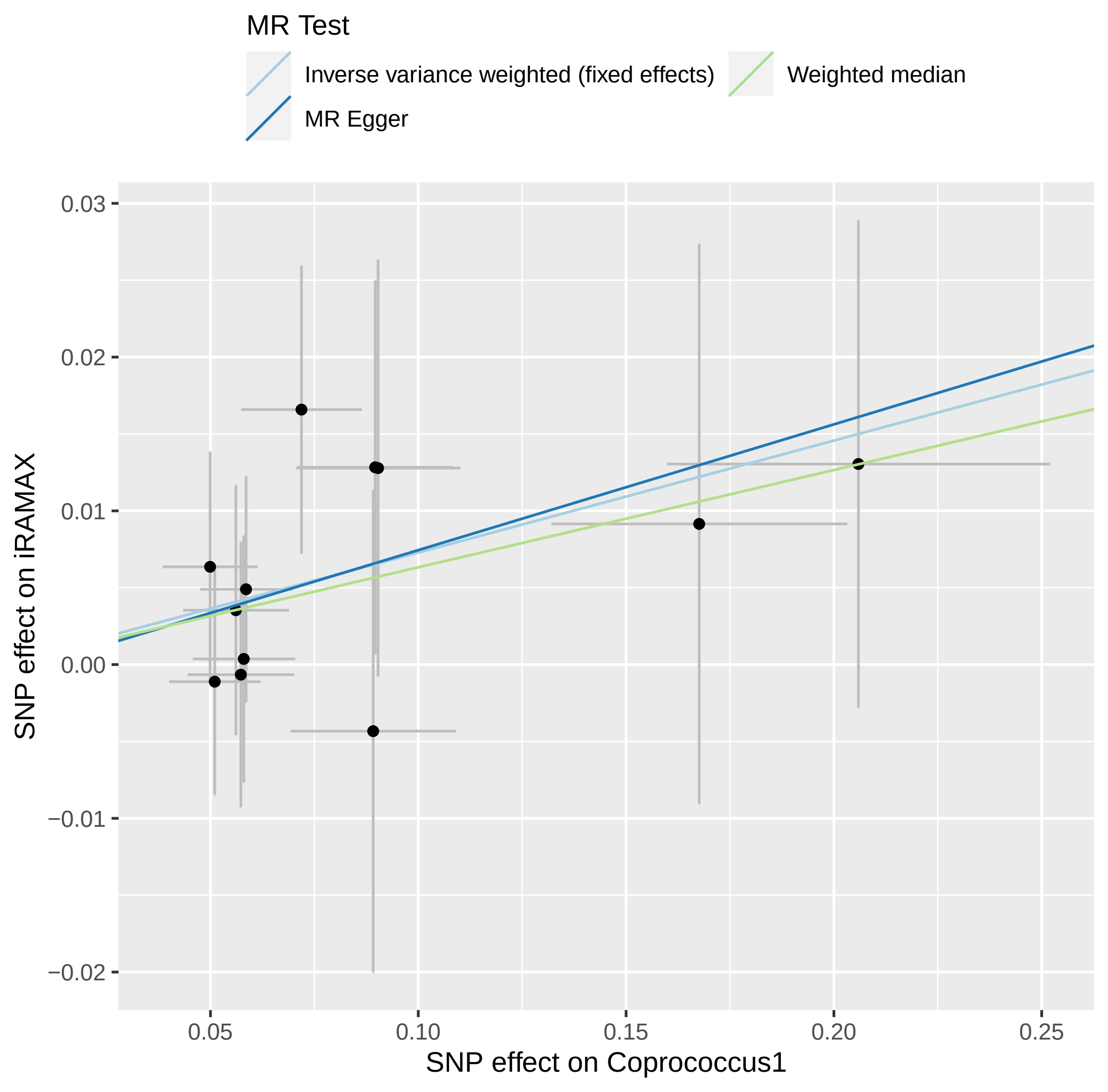
MR Test
Inverse variance weighted (fixed effects)
MR Egger
Weighted median
0.03
0.02
0.01
0.00
−0.01
−0.02
SNP effect on iRAMAX
0.05
0.10
0.15
0.20
0.25
SNP effect on Coprococcus1

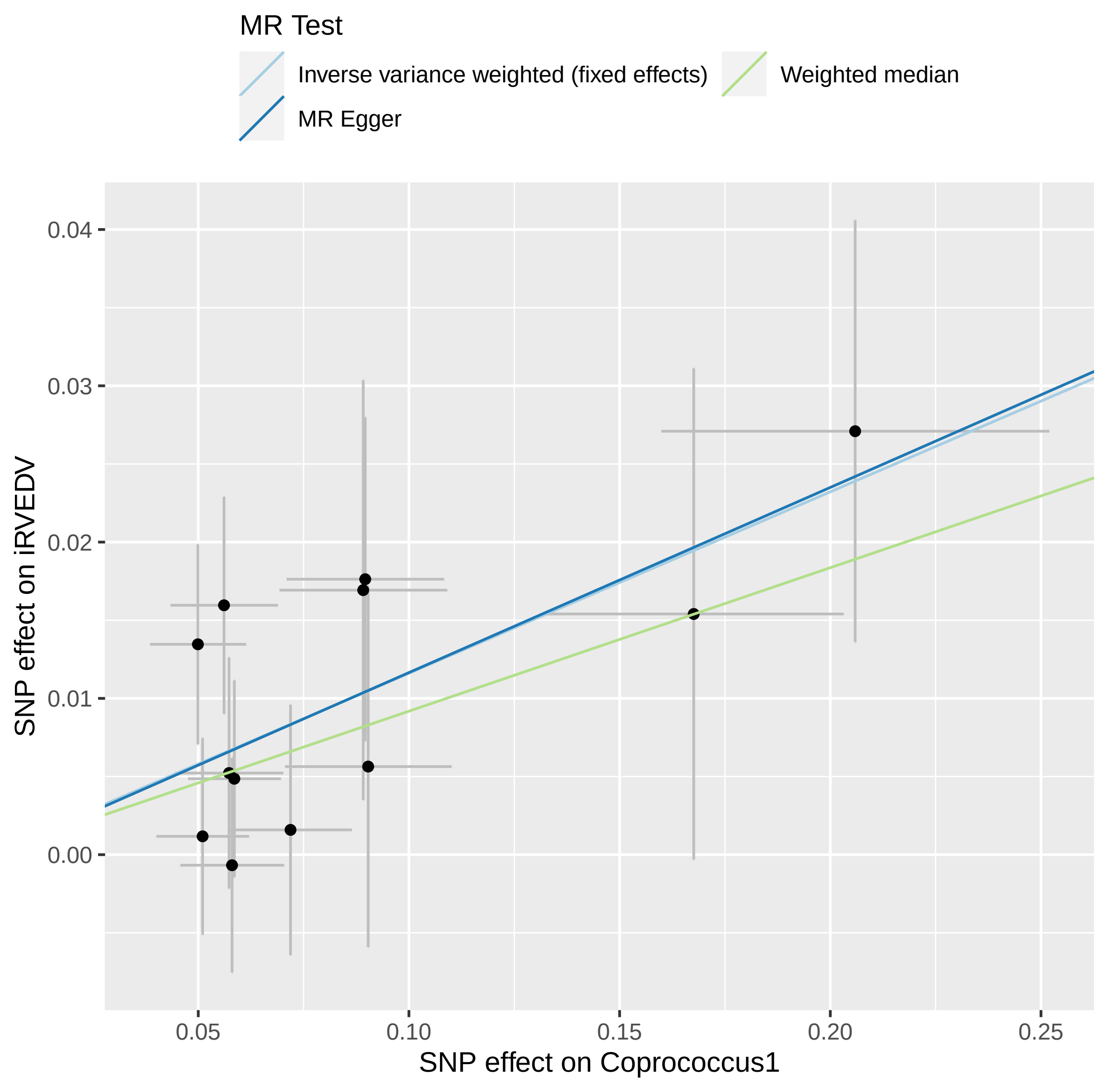

MR Test
Inverse variance weighted (fixed effects)
MR Egger
Weighted median
SNP effect on iRVEDV
0.04
0.03
0.02
0.01
0.00
0.05
0.10
0.15
0.20
0.25
SNP effect on Coprococcus1

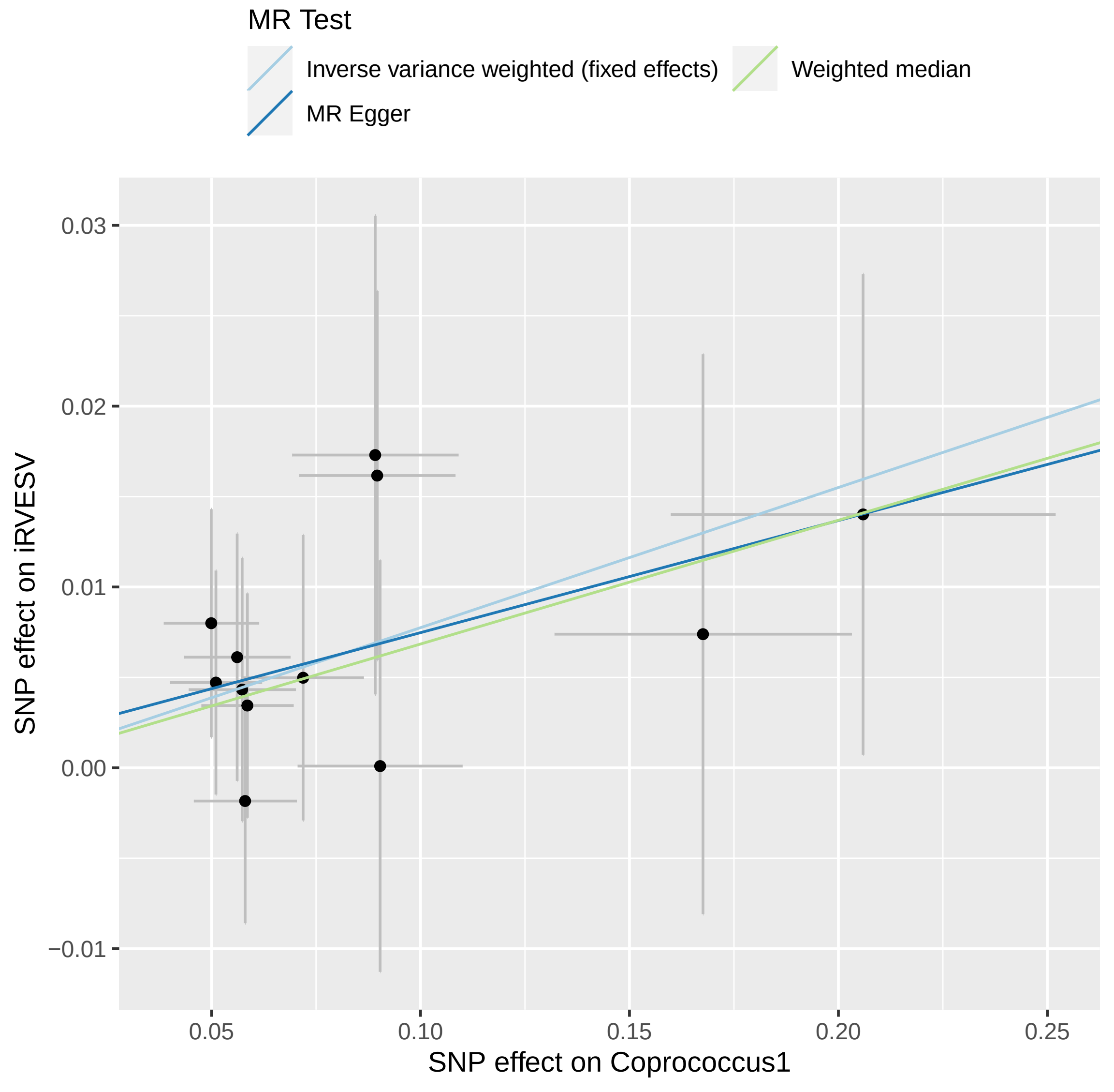
MR Test
Inverse variance weighted (fixed effects)
Weighted median
MR Egger
0.03
0.02
0.01
0.00
−0.01
SNP effect on iRVESV
0.05
0.10
0.15
0.20
0.25
SNP effect on Coprococcus1

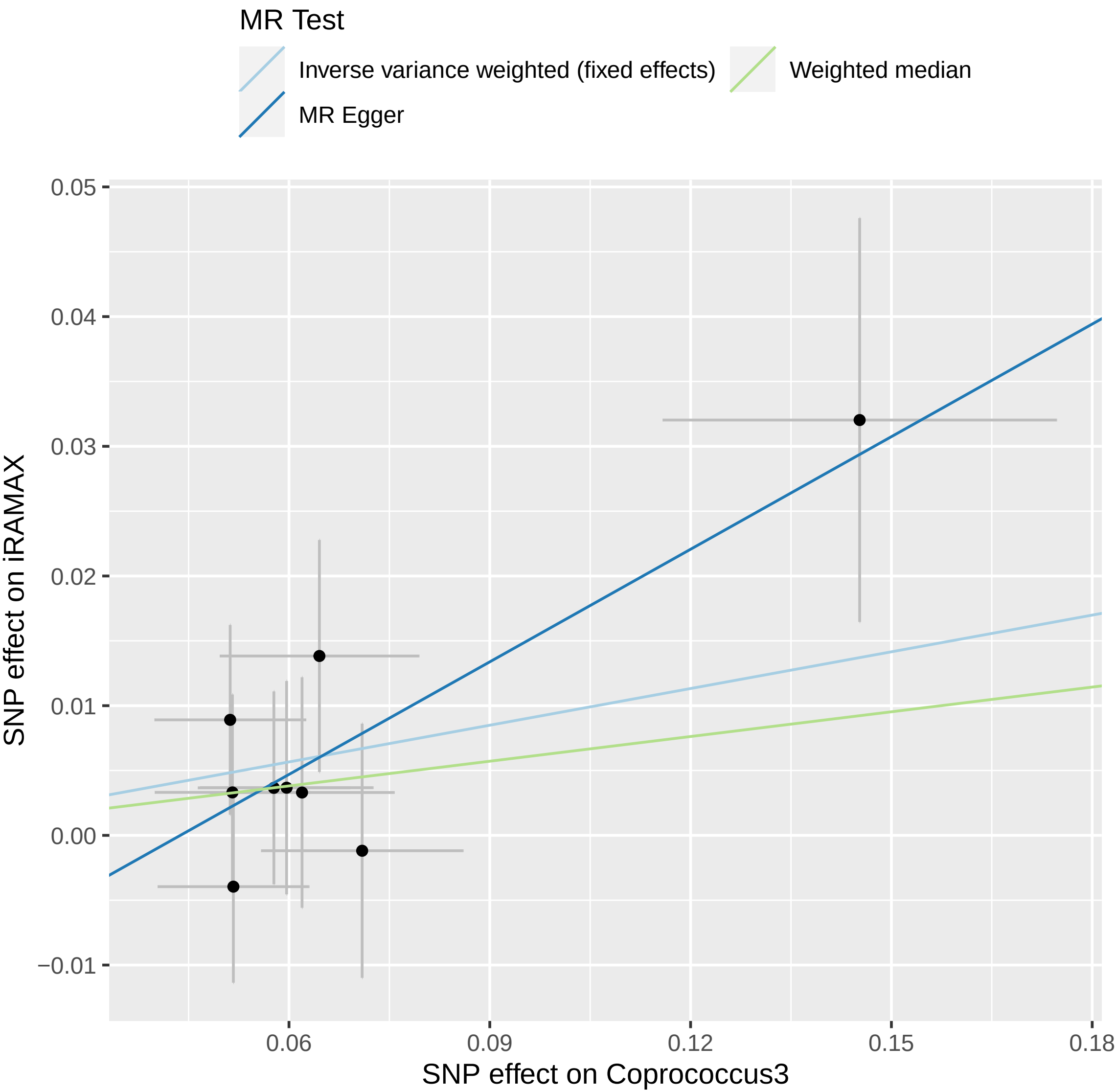
MR Test
Inverse variance weighted (fixed effects)
MR Egger
Weighted median
0.05
0.04
0.03
0.02
0.01
0.00
−0.01
SNP effect on iRAMAX
0.06
0.09
0.12
0.15
0.18
SNP effect on Coprococcus3

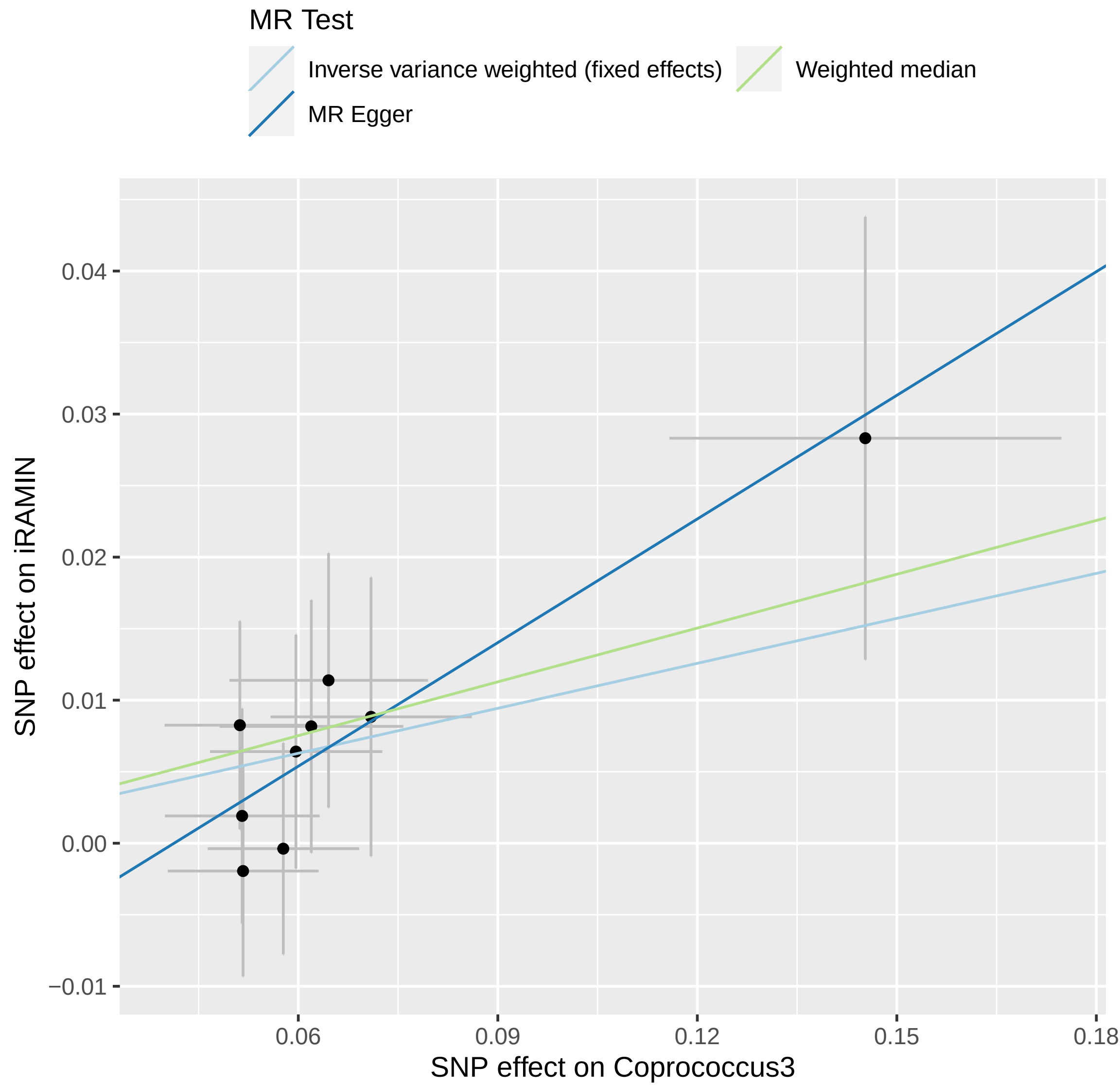

MR Test
Inverse variance weighted (fixed effects)
Weighted median
MR Egger
0.04
0.03
0.02
0.01
0.00
−0.01
0.06
0.09
0.12
0.15
0.18
SNP effect on iRAMIN
SNP effect on Coprococcus3

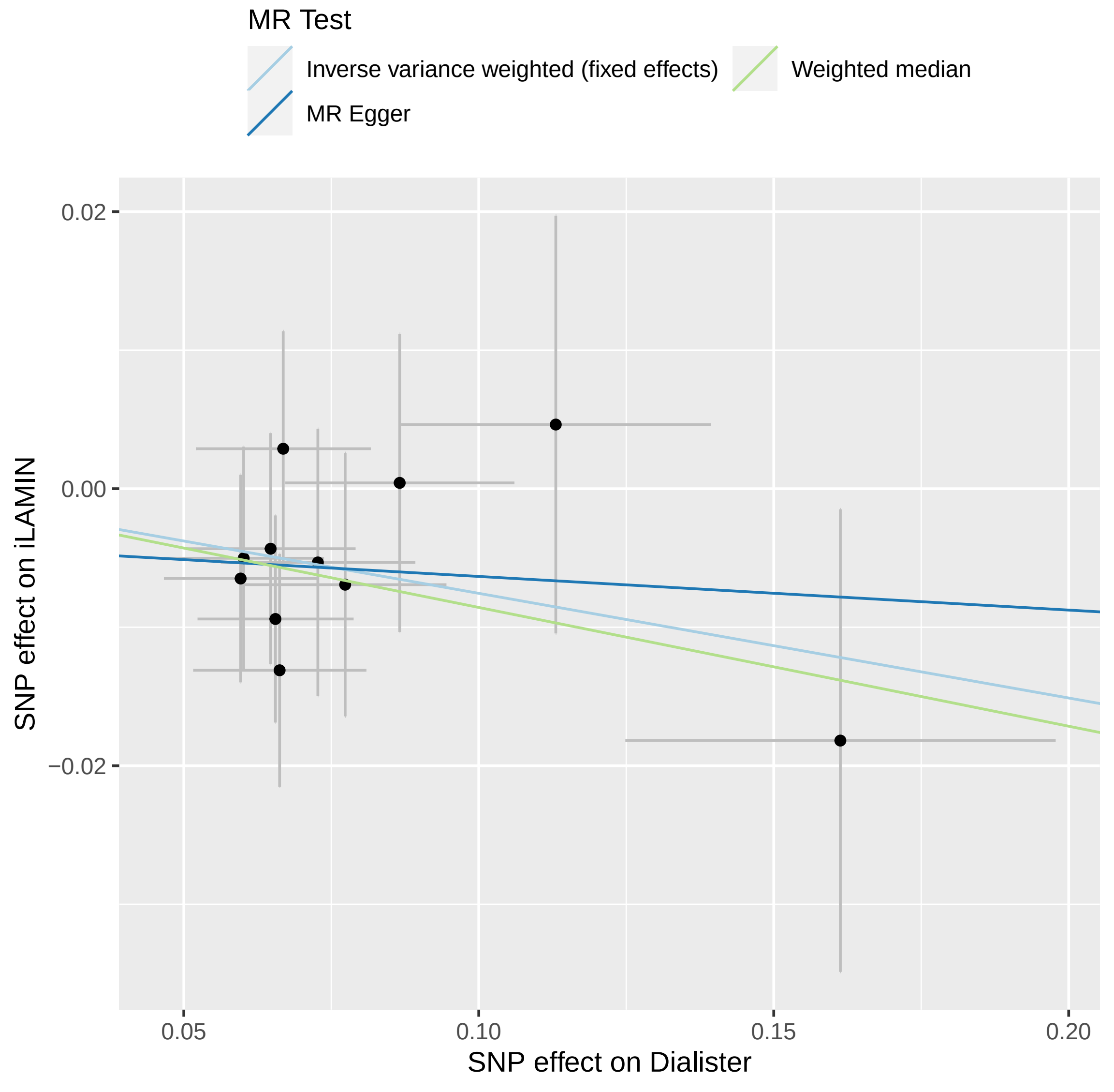

MR Test
Inverse variance weighted (fixed effects)
MR Egger
Weighted median
0.02
0.00
−0.02
0.05
0.10
0.15
0.20
SNP effect on Dialister
SNP effect on iLAMIN

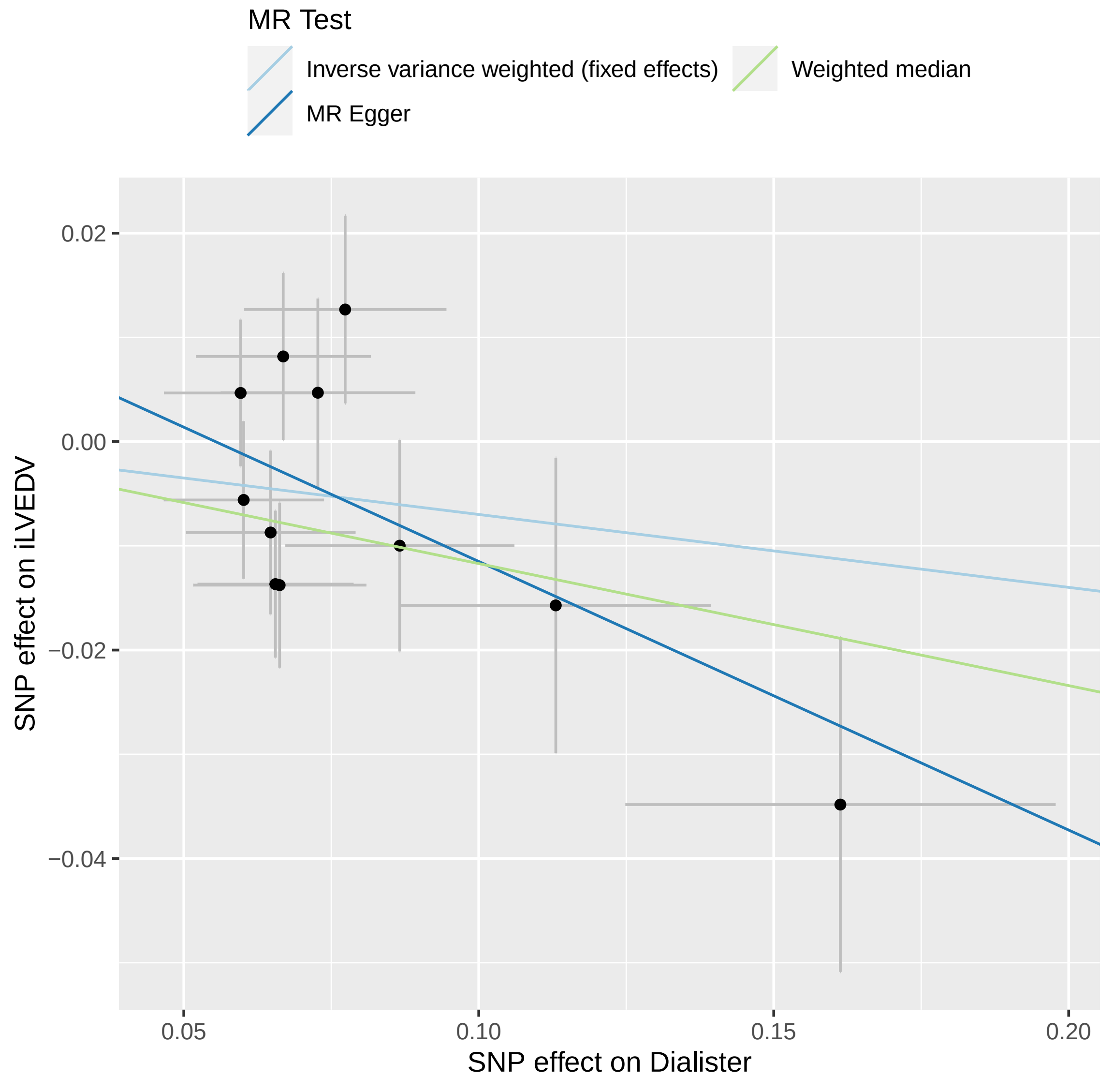

MR Test
Inverse variance weighted (fixed effects)
Weighted median
MR Egger
0.02
0.00
−0.02
−0.04
0.05
0.10
0.15
0.20
SNP effect on iLVEDV
SNP effect on Dialister

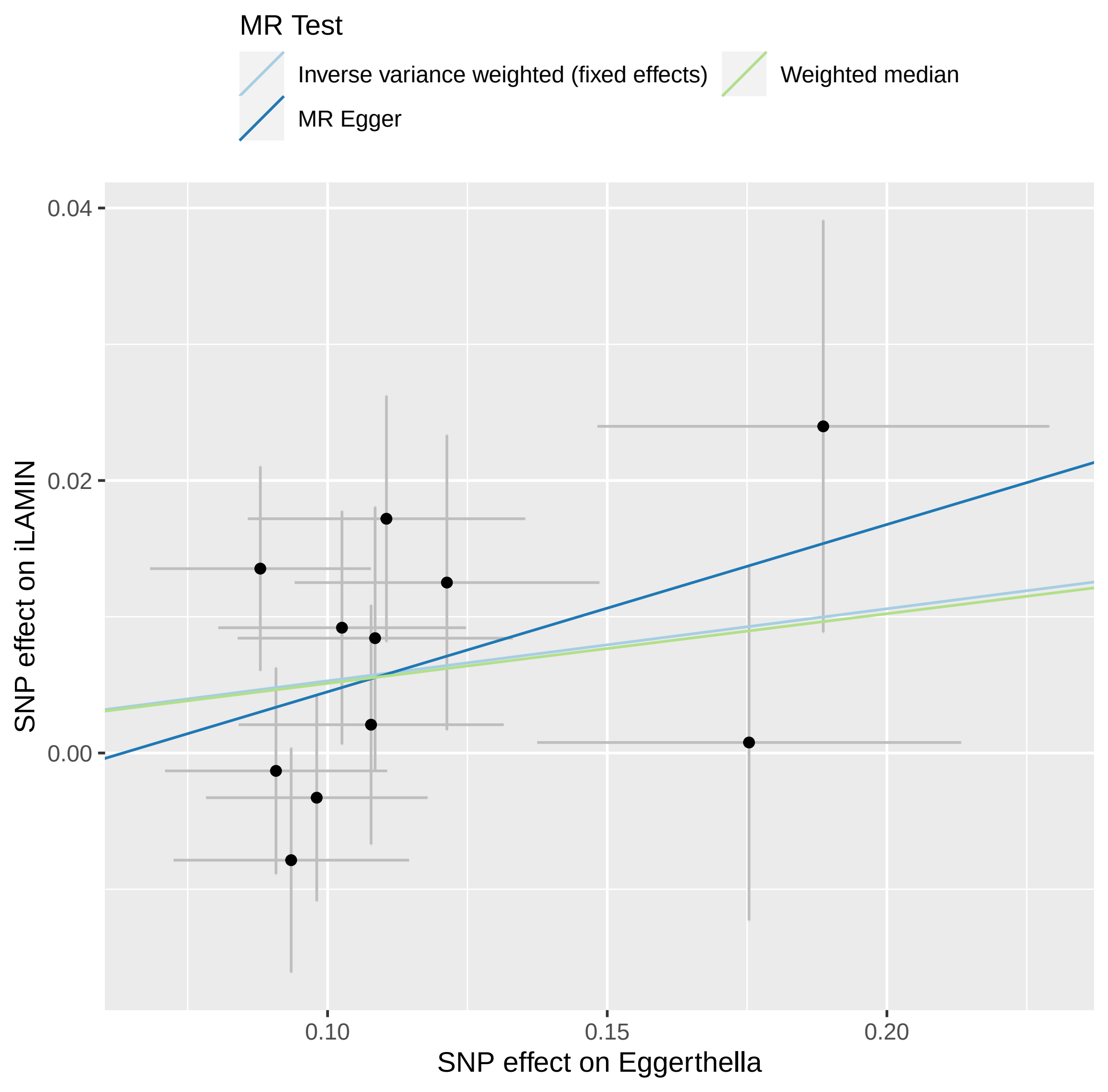

MR Test
Inverse variance weighted (fixed effects)
Weighted median
MR Egger
0.04
0.02
0.00
0.10
0.15
0.20
SNP effect on iLAMIN
SNP effect on Eggerthella

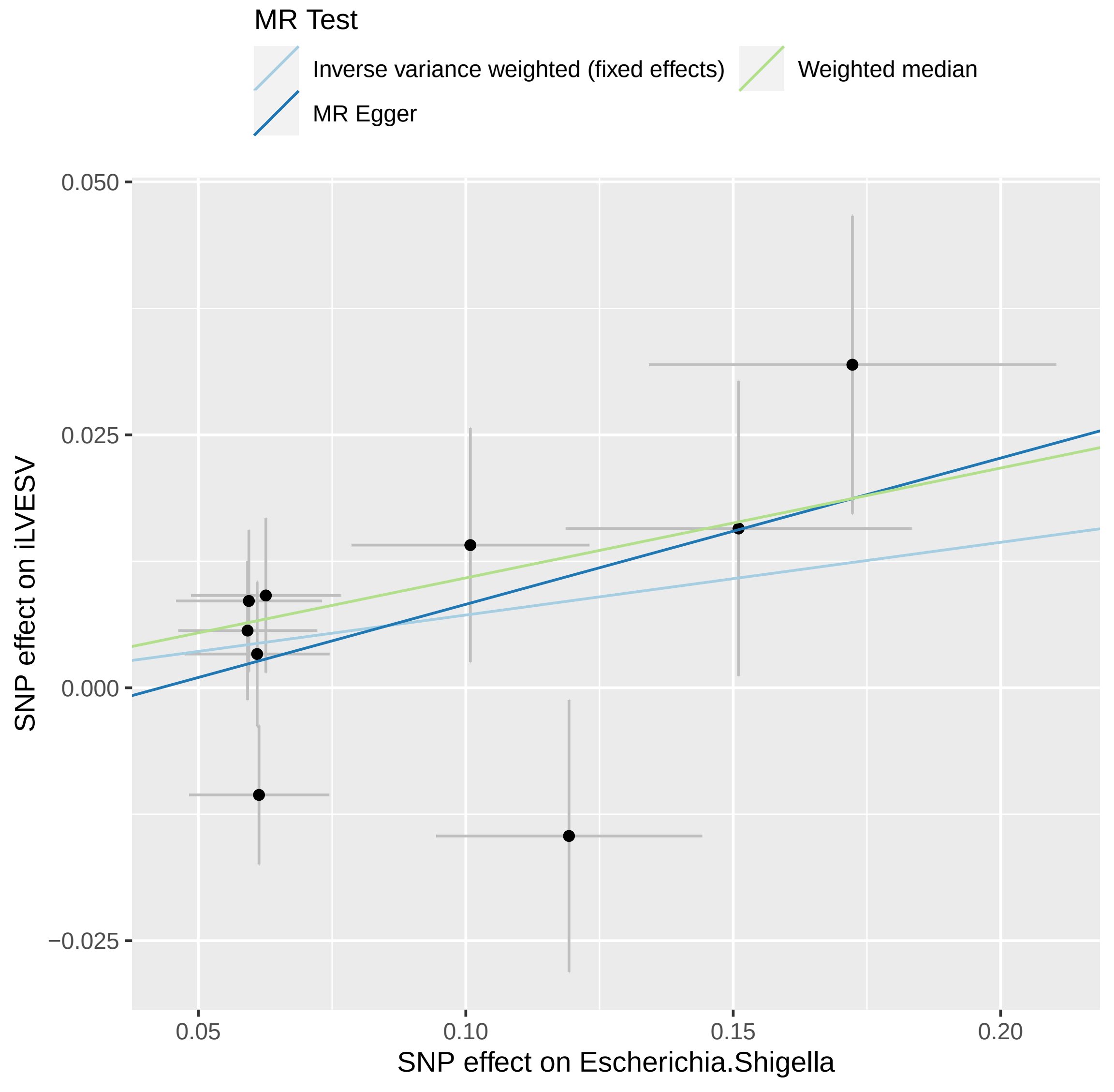
MR Test
Inverse variance weighted (fixed effects)
Weighted median
MR Egger
0.050
0.025
0.000
−0.025
0.05
0.10
0.15
0.20
SNP effect on iLVESV
SNP effect on Escherichia.Shigella

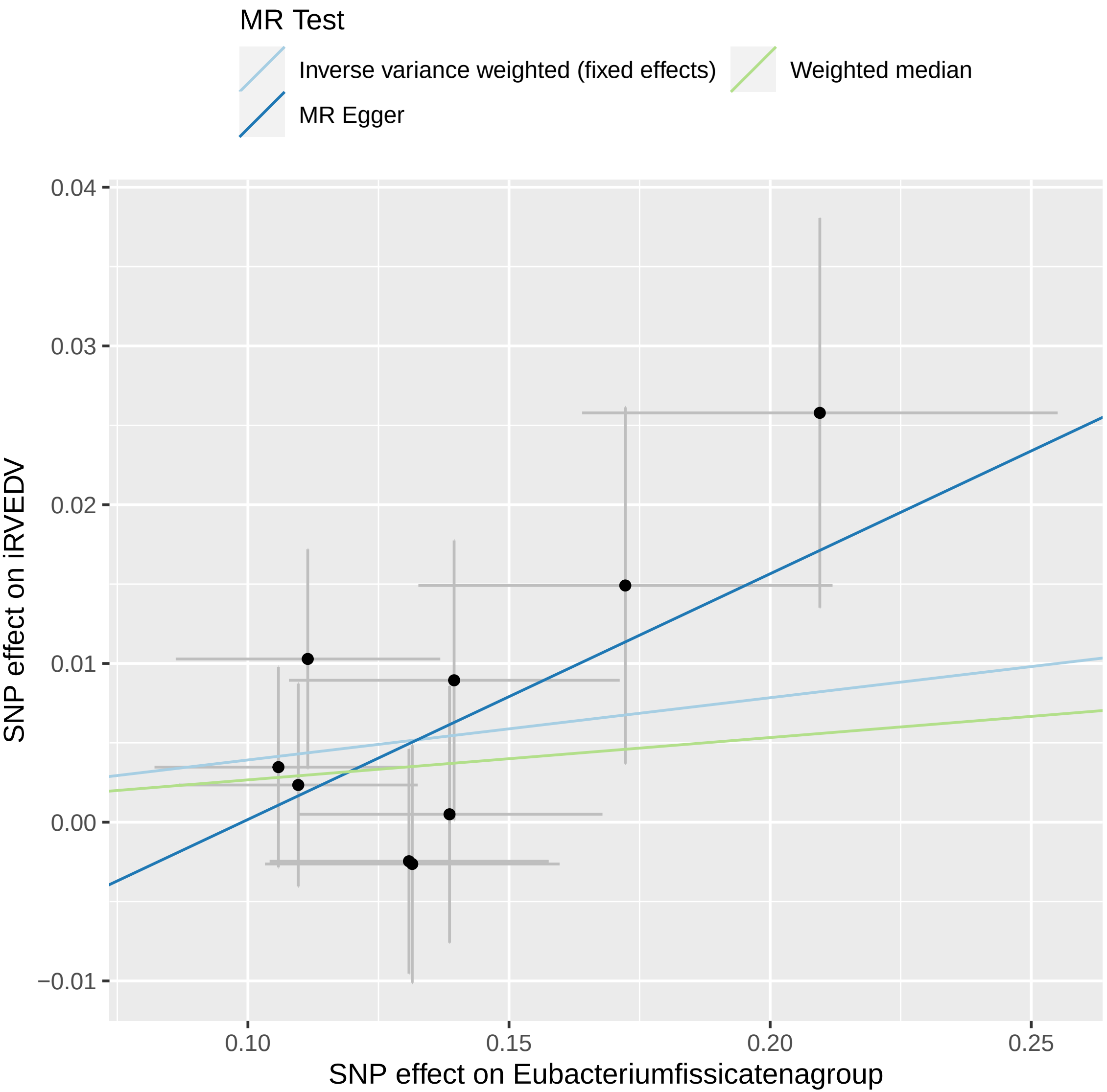

MR Test
Inverse variance weighted (fixed effects)
Weighted median
MR Egger
0.04
0.03
0.02
0.01
0.00
−0.01
0.10
0.15
0.20
0.25
SNP effect on iRVEDV
SNP effect on Eubacteriumfissicatenagroup

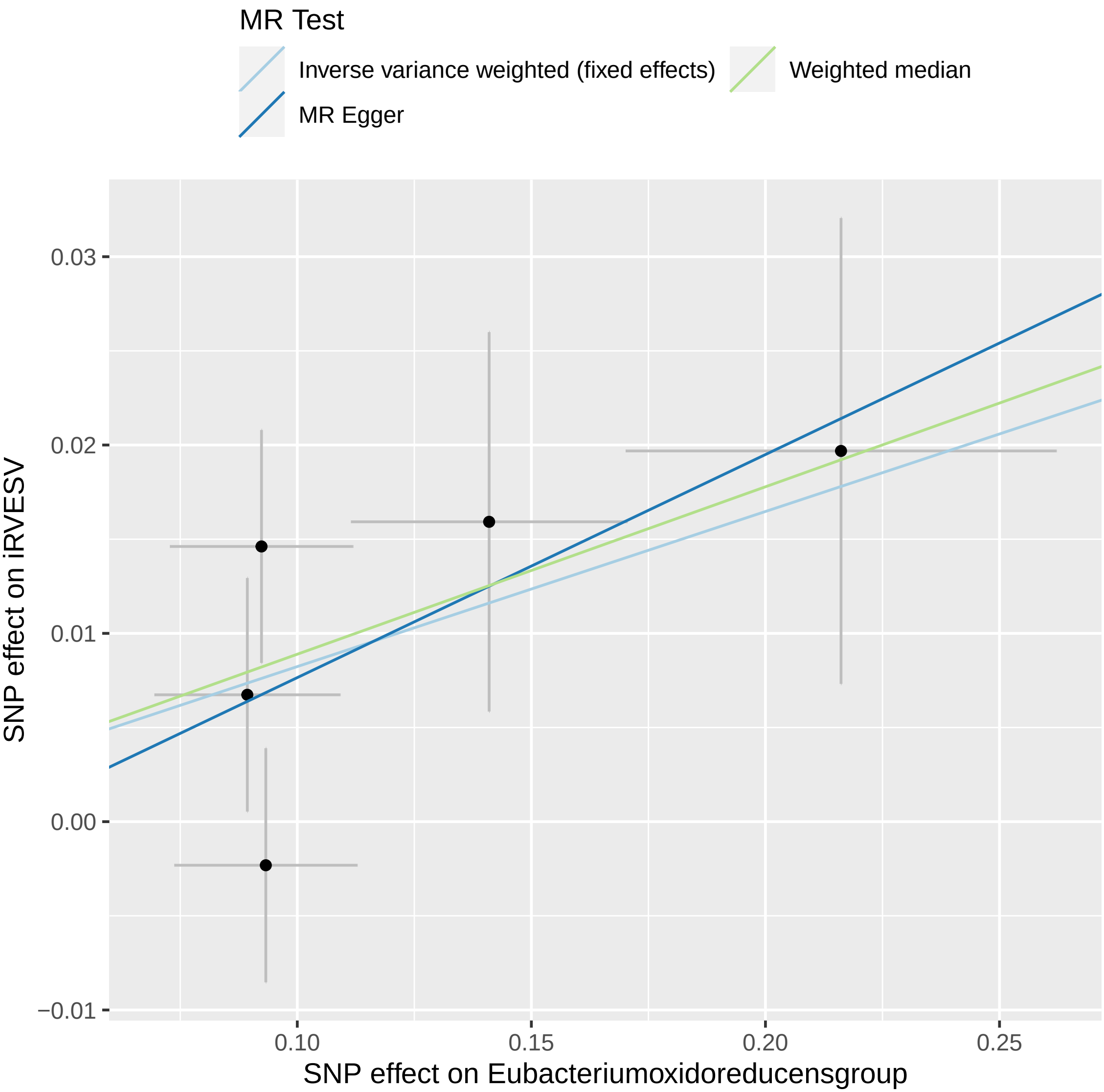
MR Test
Inverse variance weighted (fixed effects)
Weighted median
MR Egger
SNP effect on iRVESV
0.03
0.02
0.01
0.00
−0.01
0.10
0.15
0.20
0.25
SNP effect on Eubacteriumoxidoreducensgroup

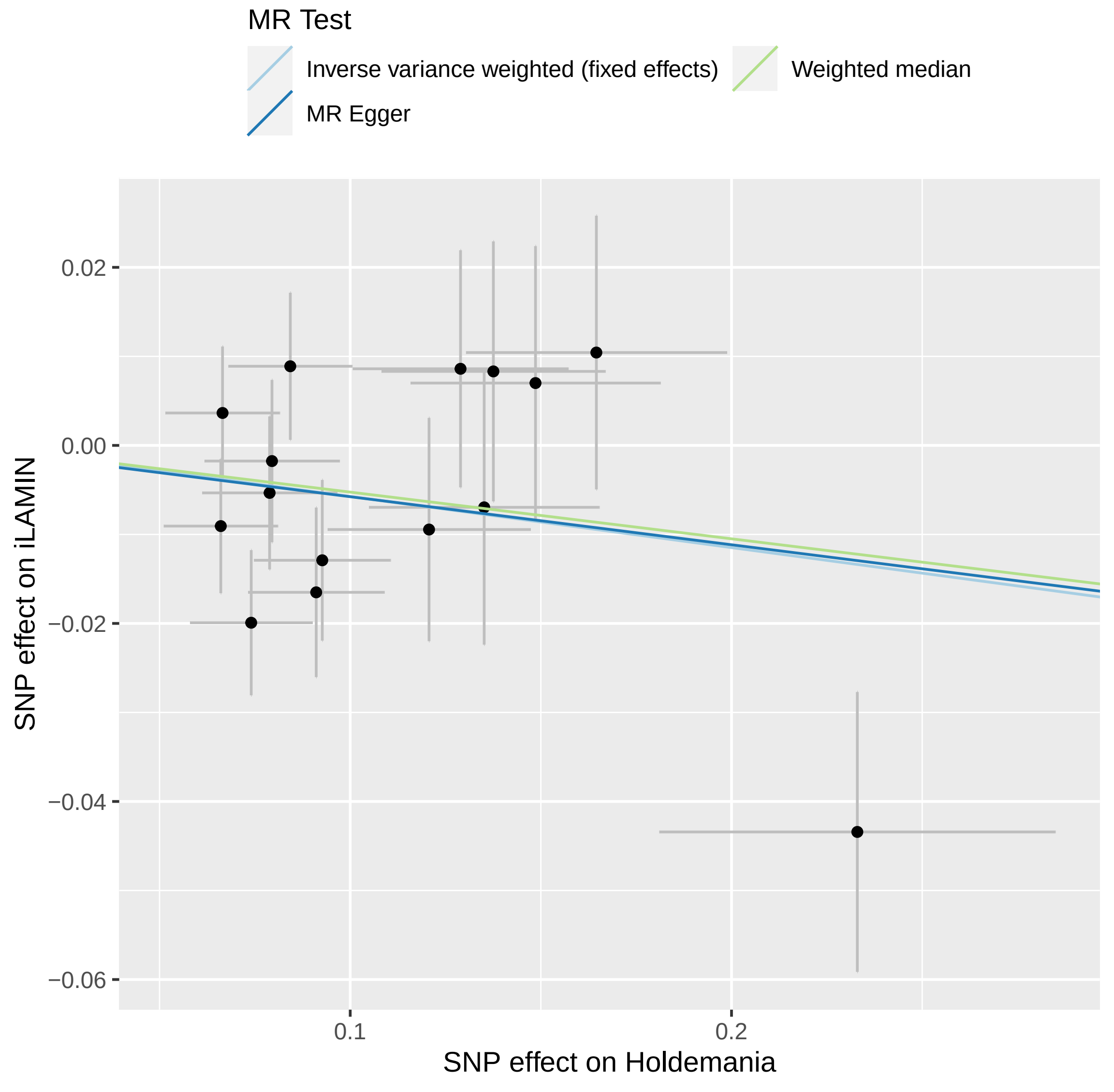
MR Test
Inverse variance weighted (fixed effects)
Weighted median
MR Egger
0.02
0.00
−0.02
−0.04
−0.06
0.1
0.2
SNP effect on iLAMIN
SNP effect on Holdemania

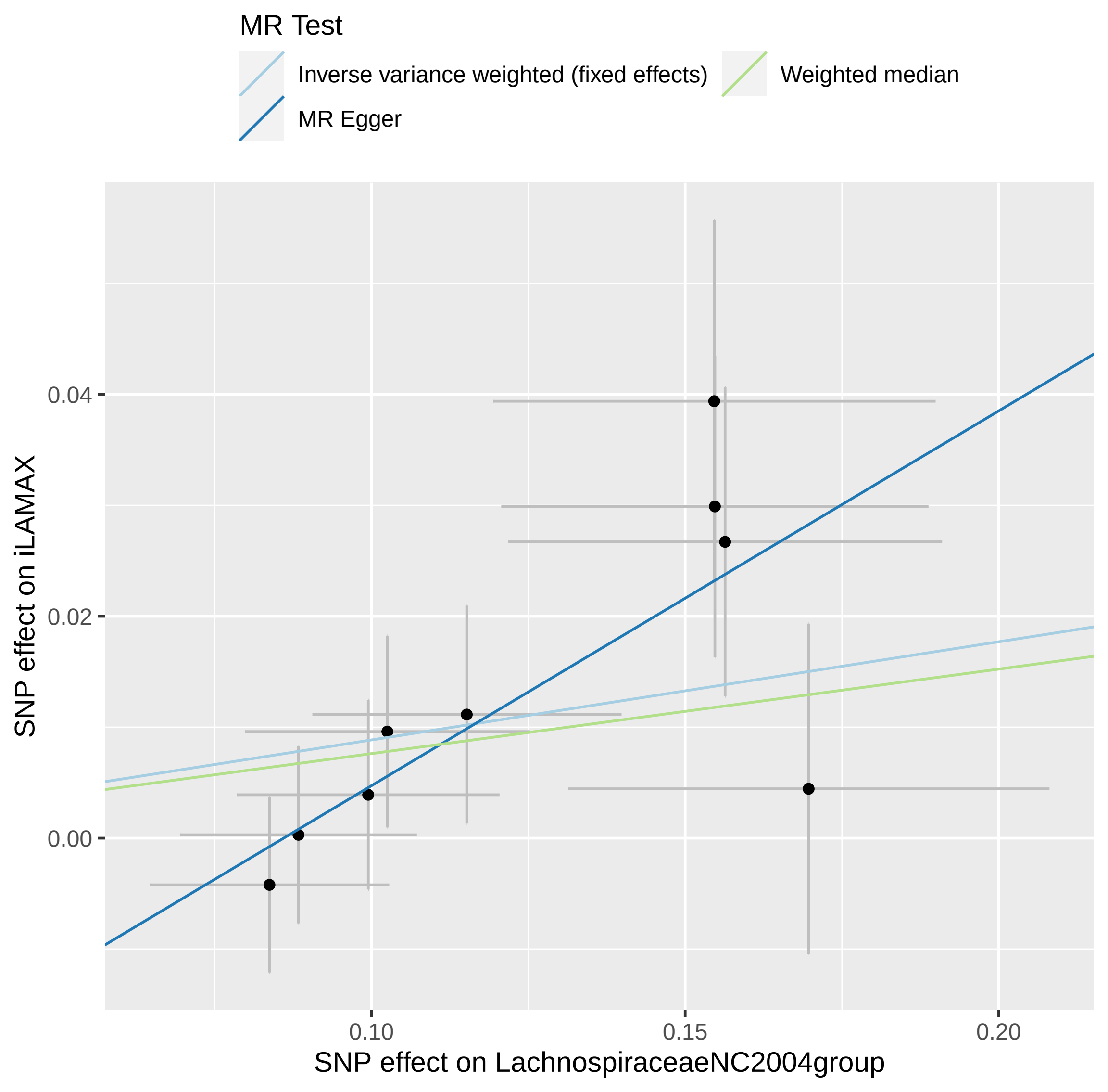
MR Test
Inverse variance weighted (fixed effects)
Weighted median
MR Egger
SNP effect on iLAMAX
0.04
0.02
0.00
0.10
0.15
0.20
SNP effect on LachnospiraceaeNC2004group

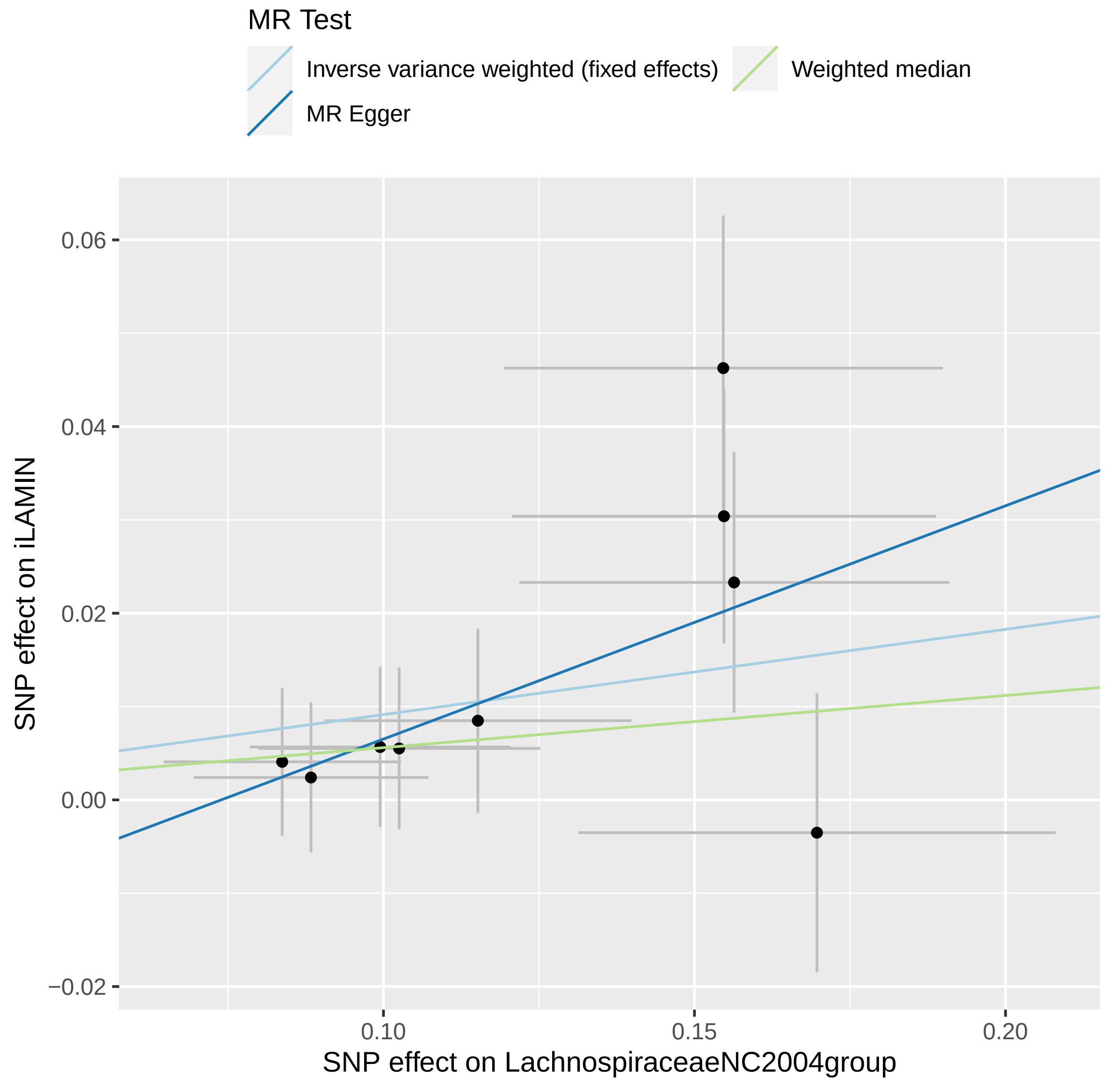
MR Test
Inverse variance weighted (fixed effects)
Weighted median
MR Egger
SNP effect on iLAMIN
0.06
0.04
0.02
0.00
−0.02
0.10
0.15
0.20
SNP effect on LachnospiraceaeNC2004group

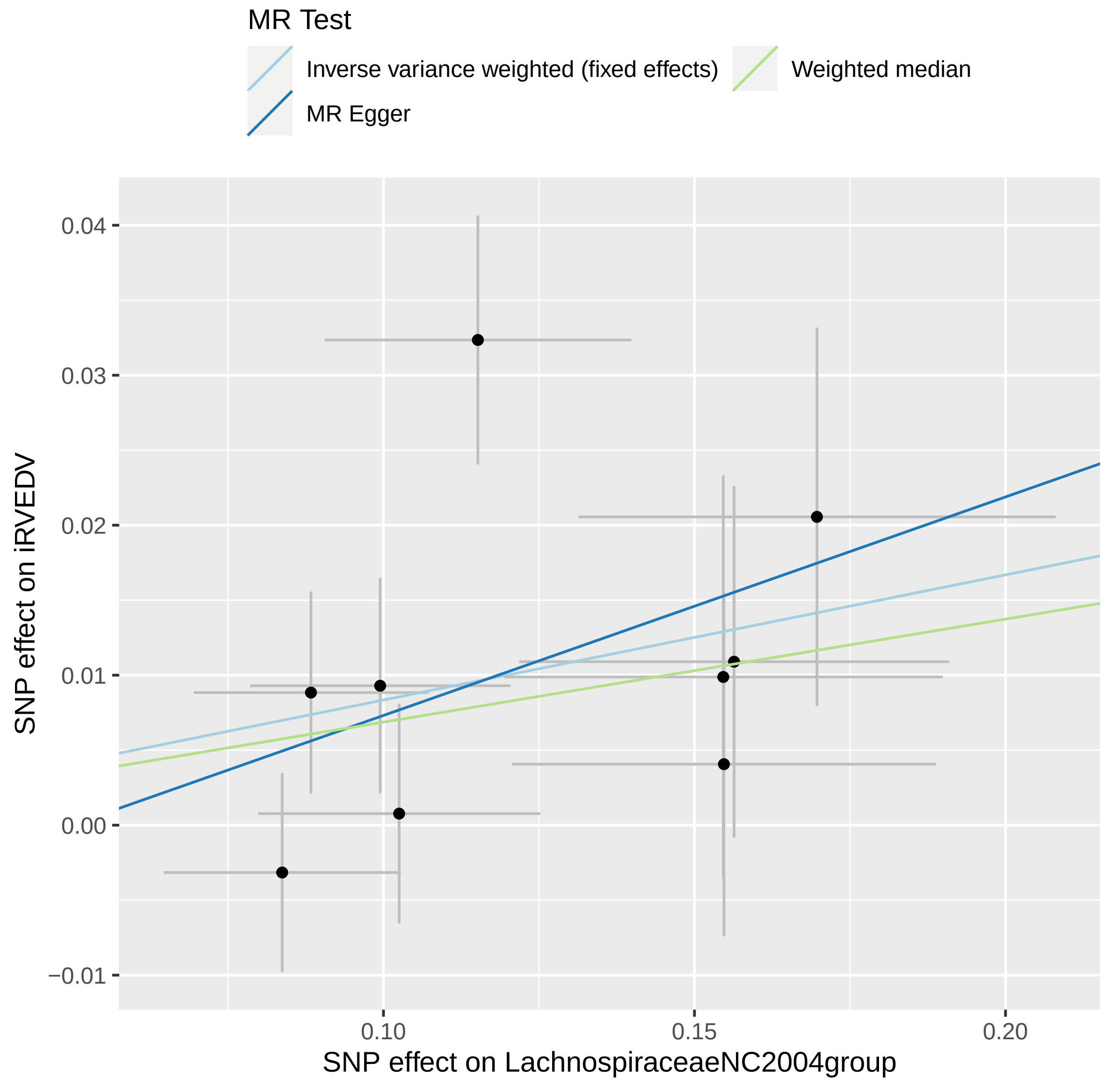

MR Test
Inverse variance weighted (fixed effects)
Weighted median
MR Egger
0.04
0.03
0.02
0.01
0.00
−0.01
0.10
0.15
0.20
SNP effect on iRVEDV
SNP effect on LachnospiraceaeNC2004group

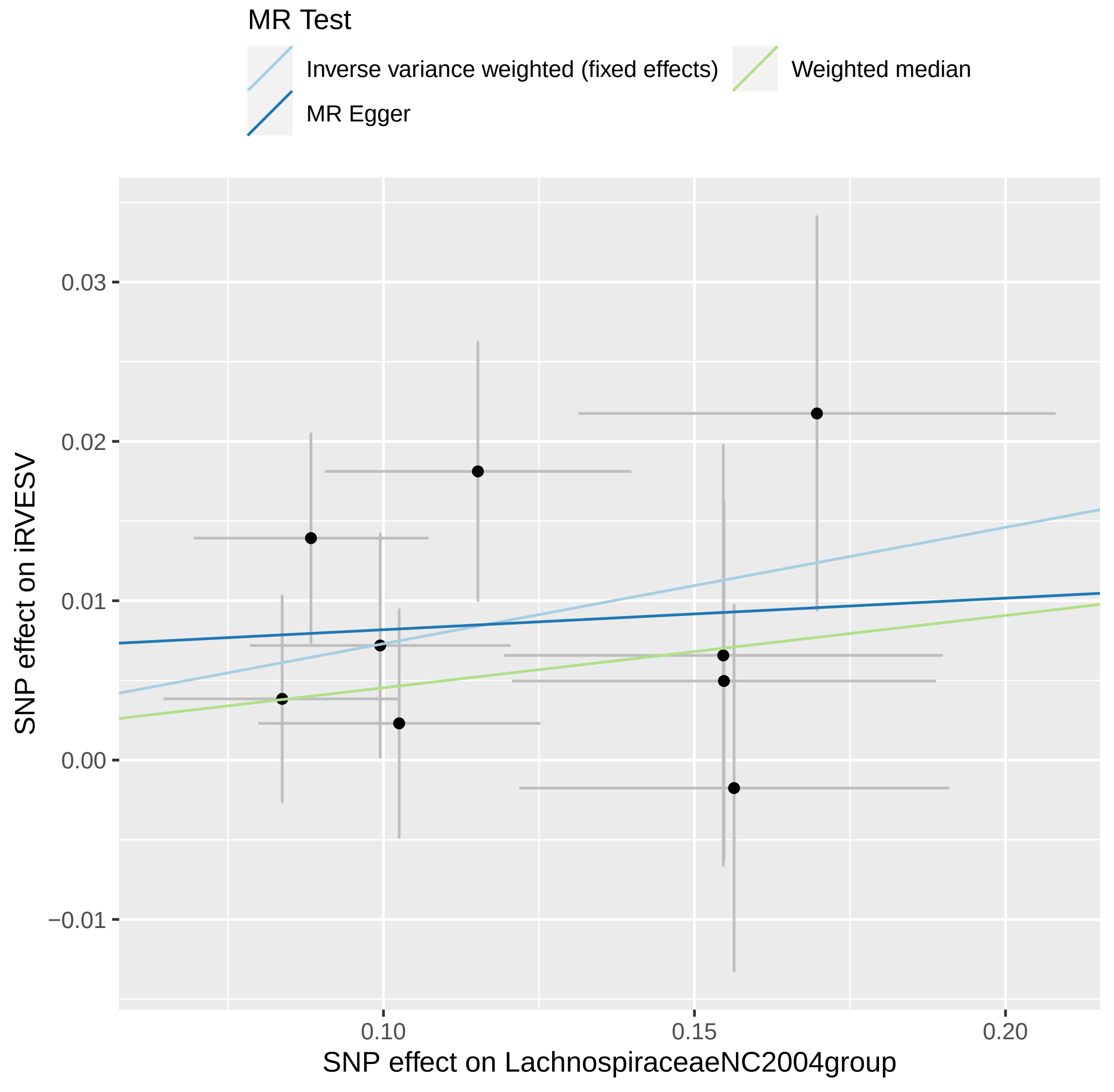

MR Test
Inverse variance weighted (fixed effects)
Weighted median
MR Egger
0.03
0.02
0.01
0.00
−0.01
0.10
0.15
0.20
SNP effect on iRVESV
SNP effect on LachnospiraceaeNC2004group

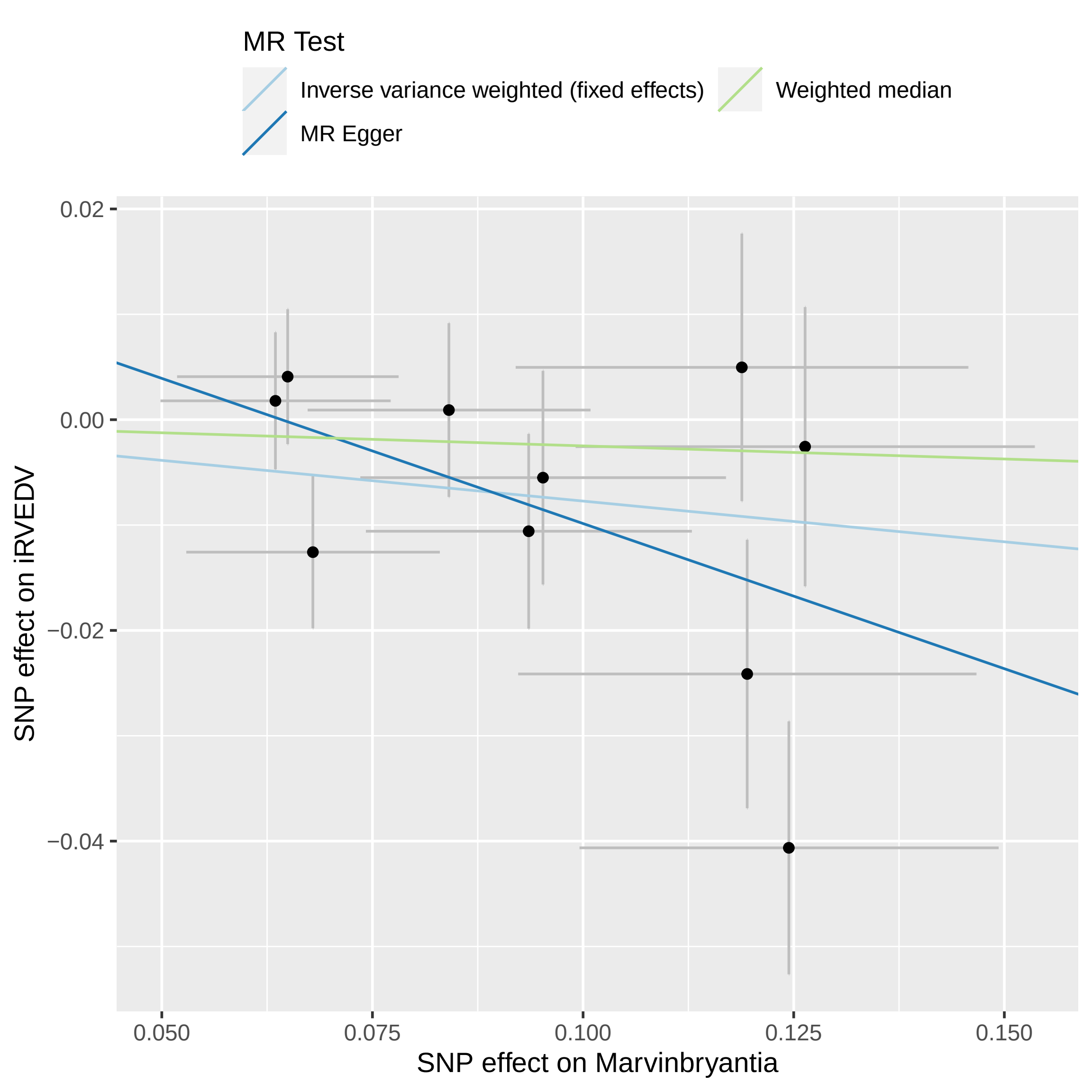
MR Test
Inverse variance weighted (fixed effects)
MR Egger
Weighted median
0.02
0.00
−0.02
−0.04
0.050
0.075
0.100
0.125
0.150
SNP effect on iRVEDV
SNP effect on Marvinbryantia

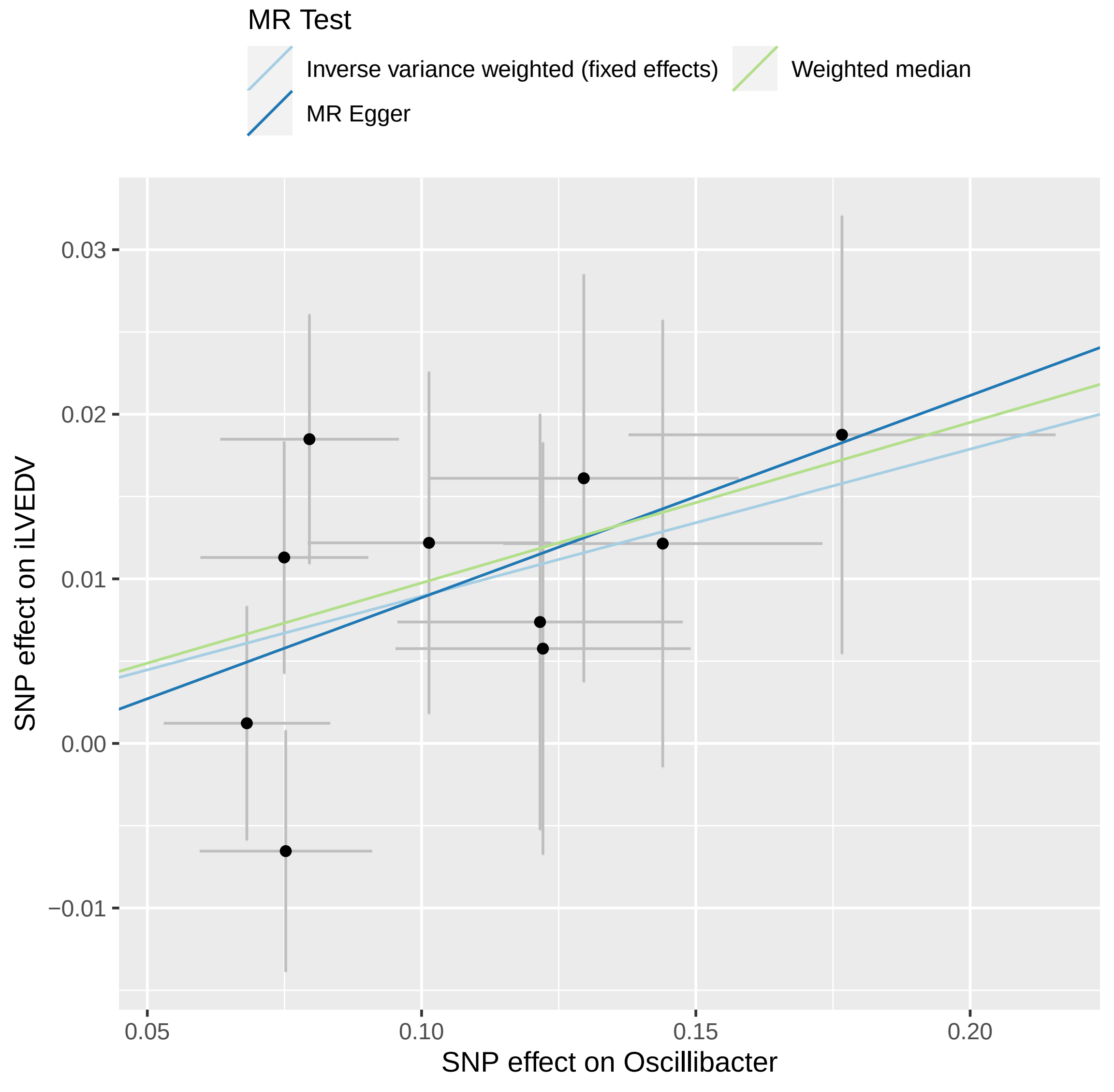
MR Test
Inverse variance weighted (fixed effects)
Weighted median
MR Egger
0.03
0.02
0.01
0.00
−0.01
0.05
0.10
0.15
0.20
SNP effect on iLVEDV
SNP effect on Oscillibacter

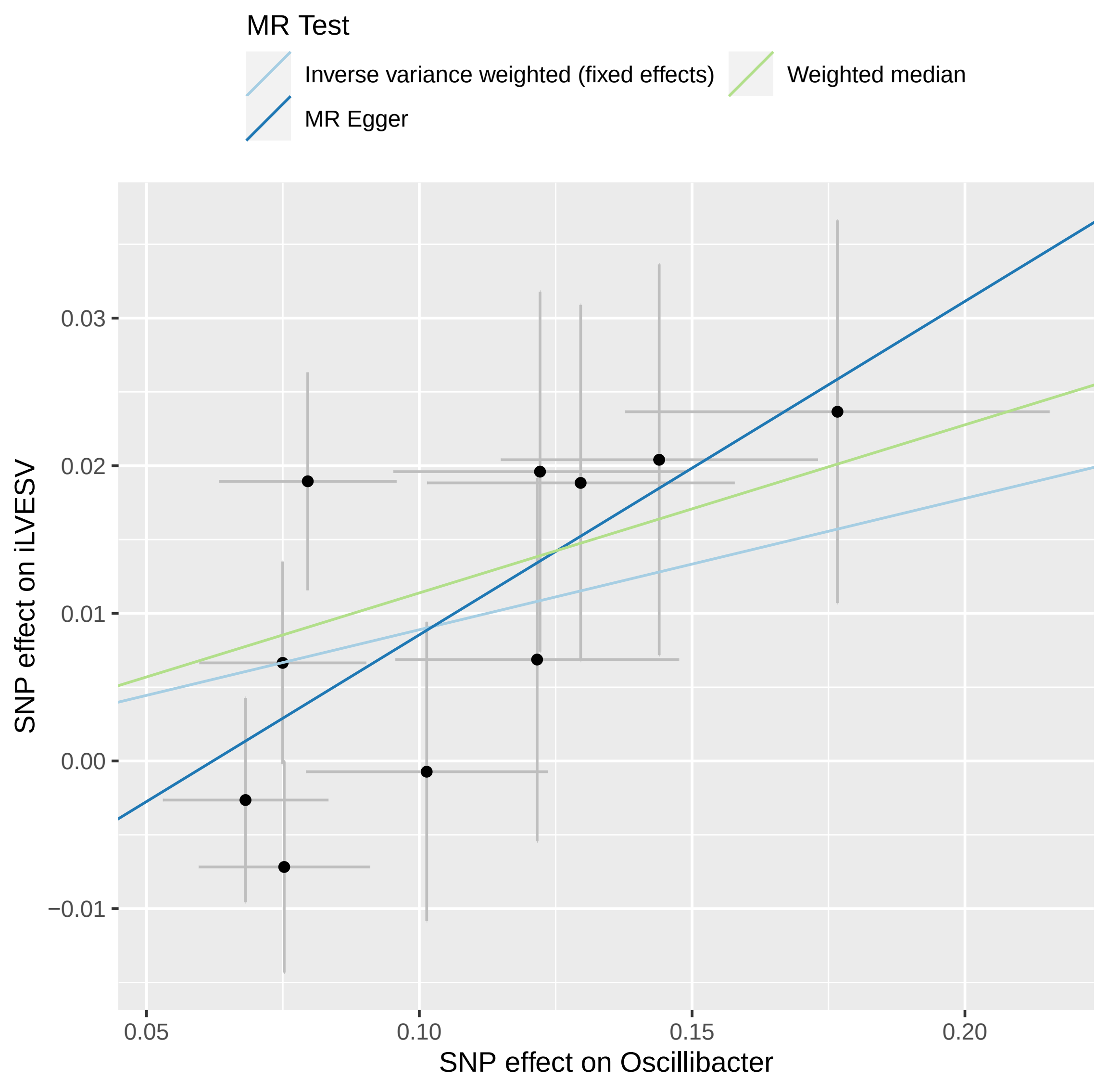
MR Test
Inverse variance weighted (fixed effects)
MR Egger
Weighted median
SNP effect on iLVESV
SNP effect on Oscillibacter
0.03
0.02
0.01
0.00
−0.01
0.05
0.10
0.15
0.20

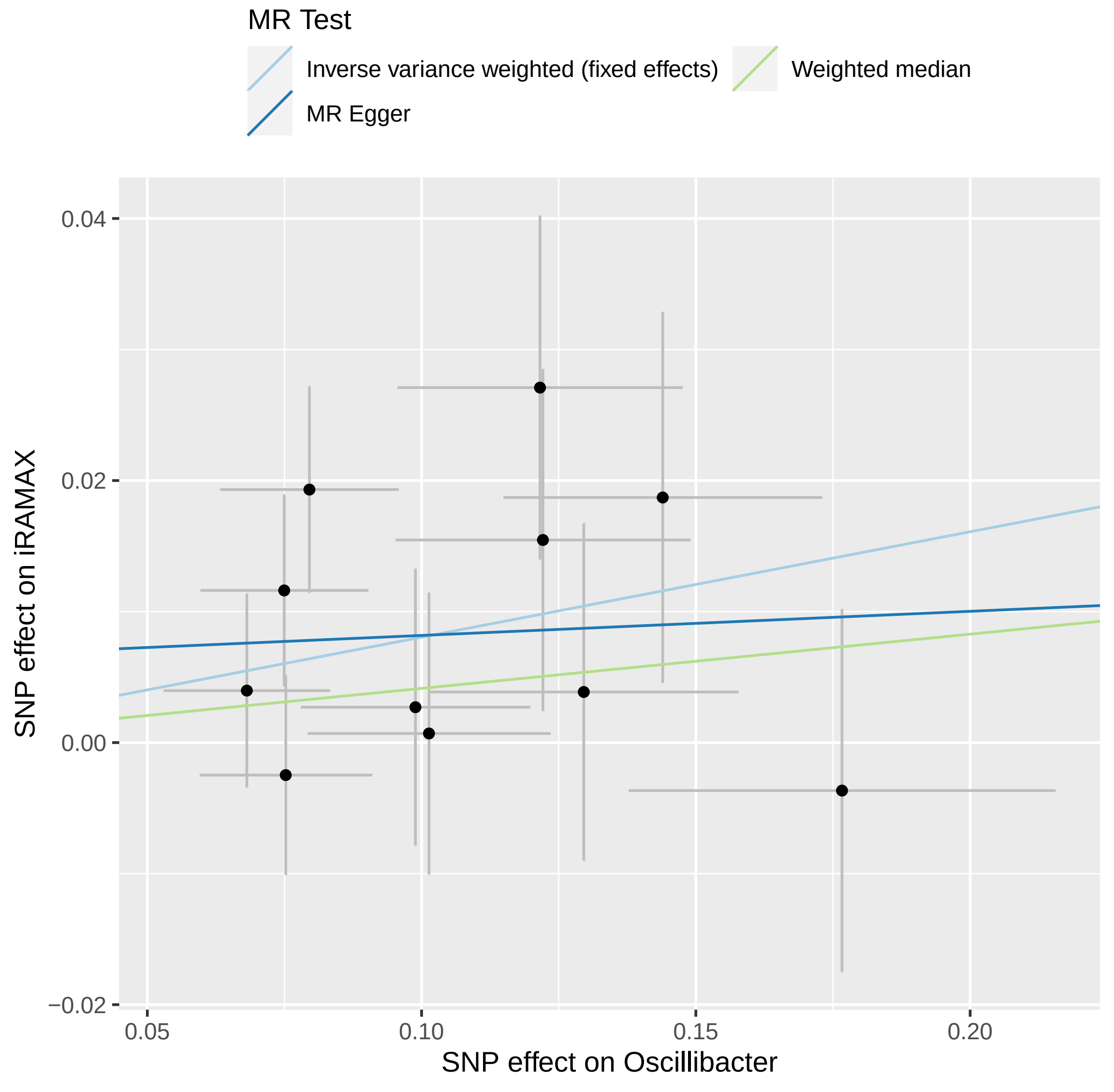
MR Test
Inverse variance weighted (fixed effects)
Weighted median
MR Egger
SNP effect on iRAMAX
0.04
0.02
0.00
−0.02
0.05
0.10
0.15
0.20
SNP effect on Oscillibacter

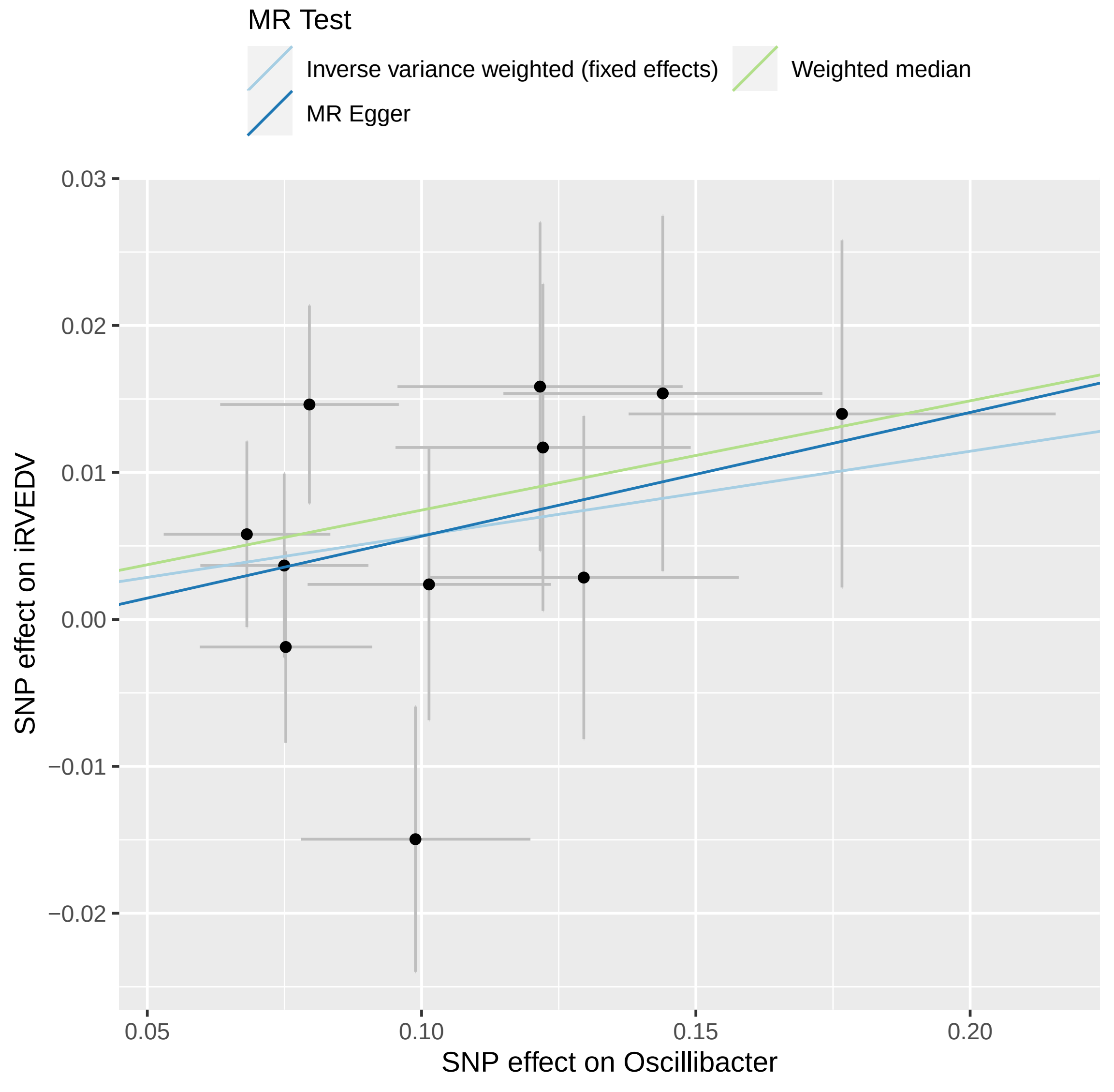
MR Test
Inverse variance weighted (fixed effects)
Weighted median
MR Egger
0.03
0.02
0.01
0.00
−0.01
−0.02
0.05
0.10
0.15
0.20
SNP effect on iRVEDV
SNP effect on Oscillibacter

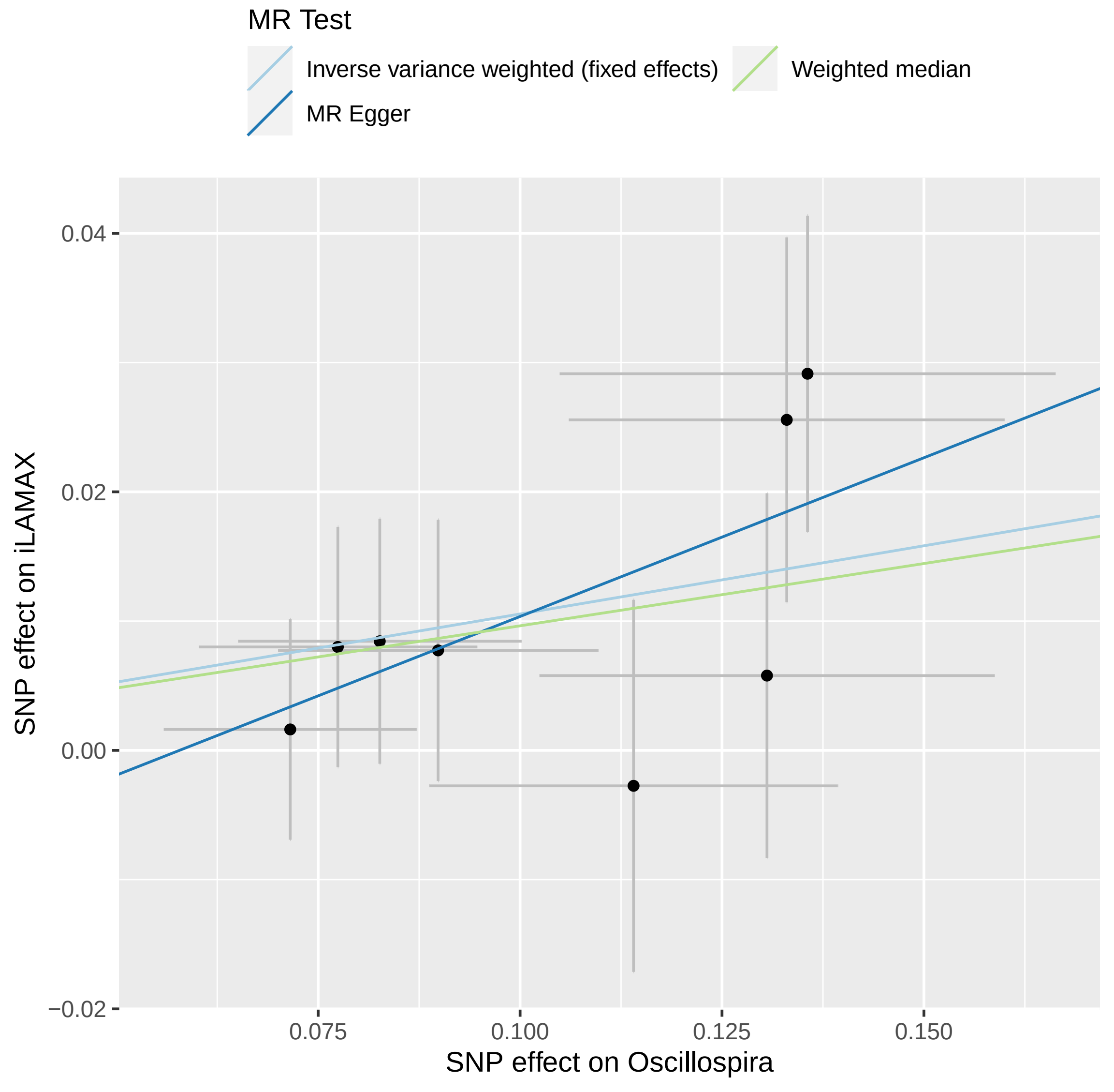

MR Test
Inverse variance weighted (fixed effects)
Weighted median
MR Egger
0.04
0.02
0.00
−0.02
SNP effect on iLAMAX
0.075
0.100
0.125
0.150
SNP effect on Oscillospira

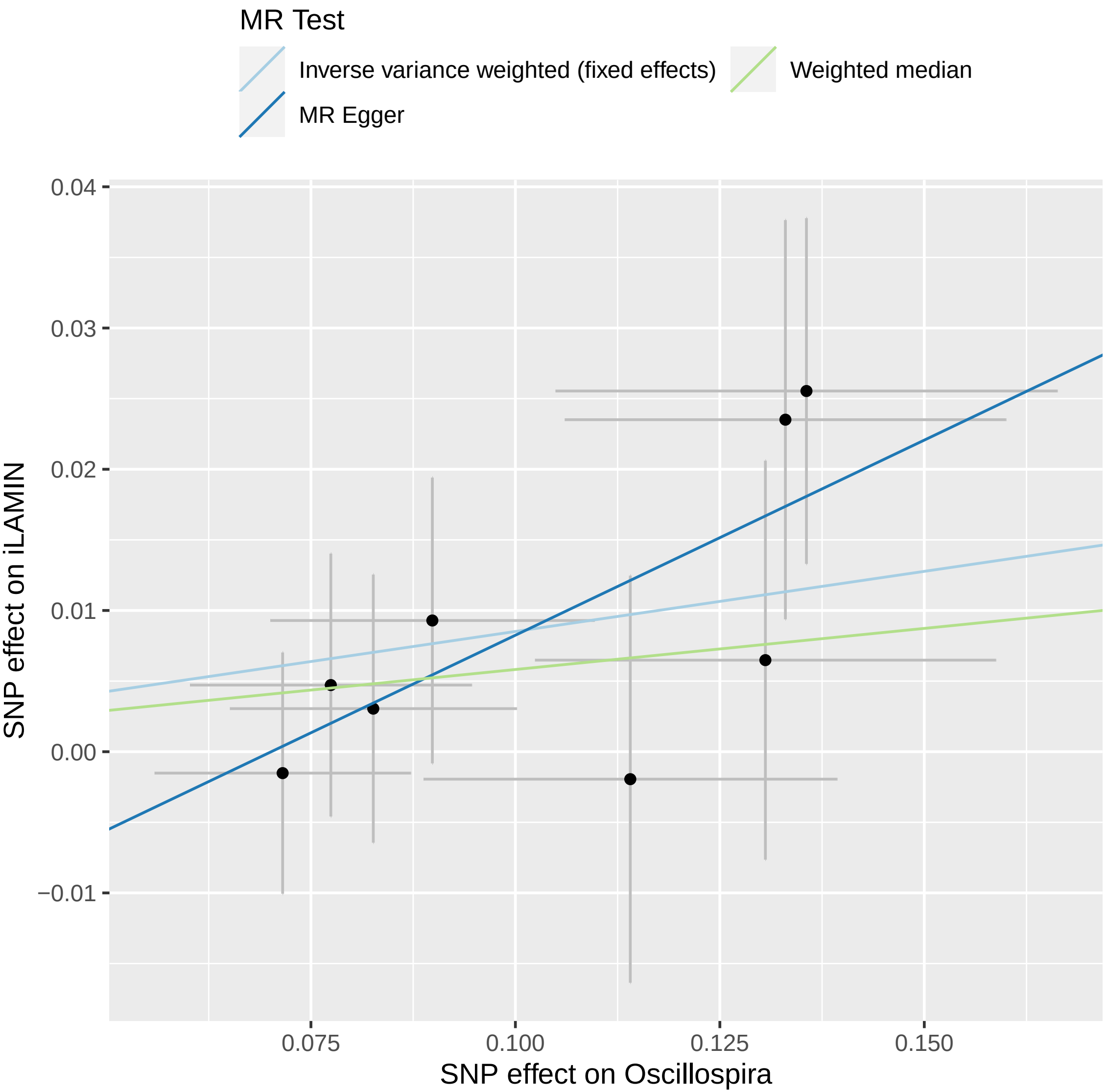

MR Test
Inverse variance weighted (fixed effects)
Weighted median
MR Egger
0.04
0.03
0.02
0.01
0.00
−0.01
0.075
0.100
0.125
0.150
SNP effect on iLAMIN
SNP effect on Oscillospira

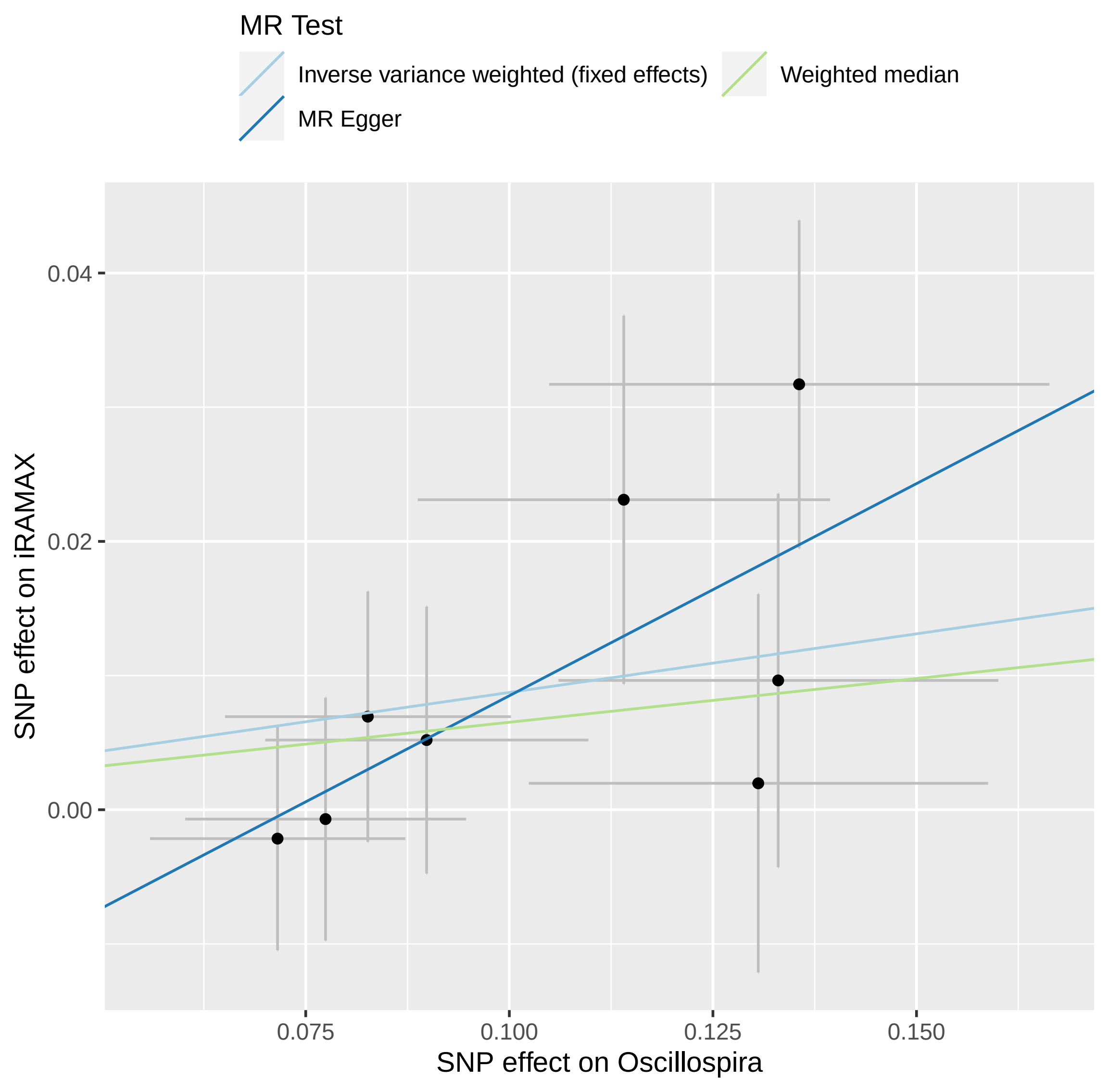
MR Test
Inverse variance weighted (fixed effects)
MR Egger
Weighted median
SNP effect on iRAMAX
0.04
0.02
0.00
0.075
0.100
0.125
0.150
SNP effect on Oscillospira

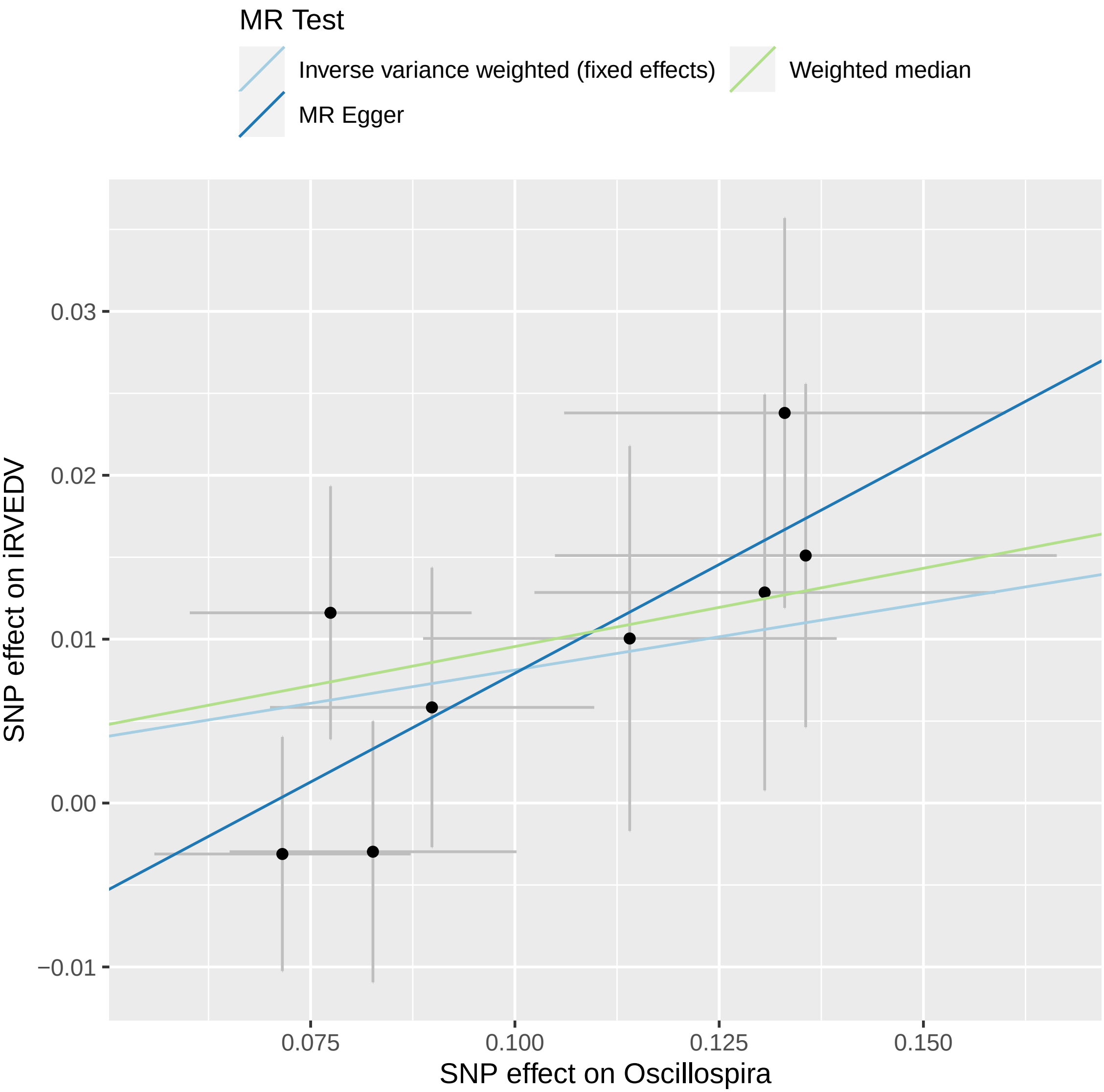

MR Test
Inverse variance weighted (fixed effects)
Weighted median
MR Egger
0.03
0.02
0.01
0.00
−0.01
0.075
0.100
0.125
0.150
SNP effect on iRVEDV
SNP effect on Oscillospira

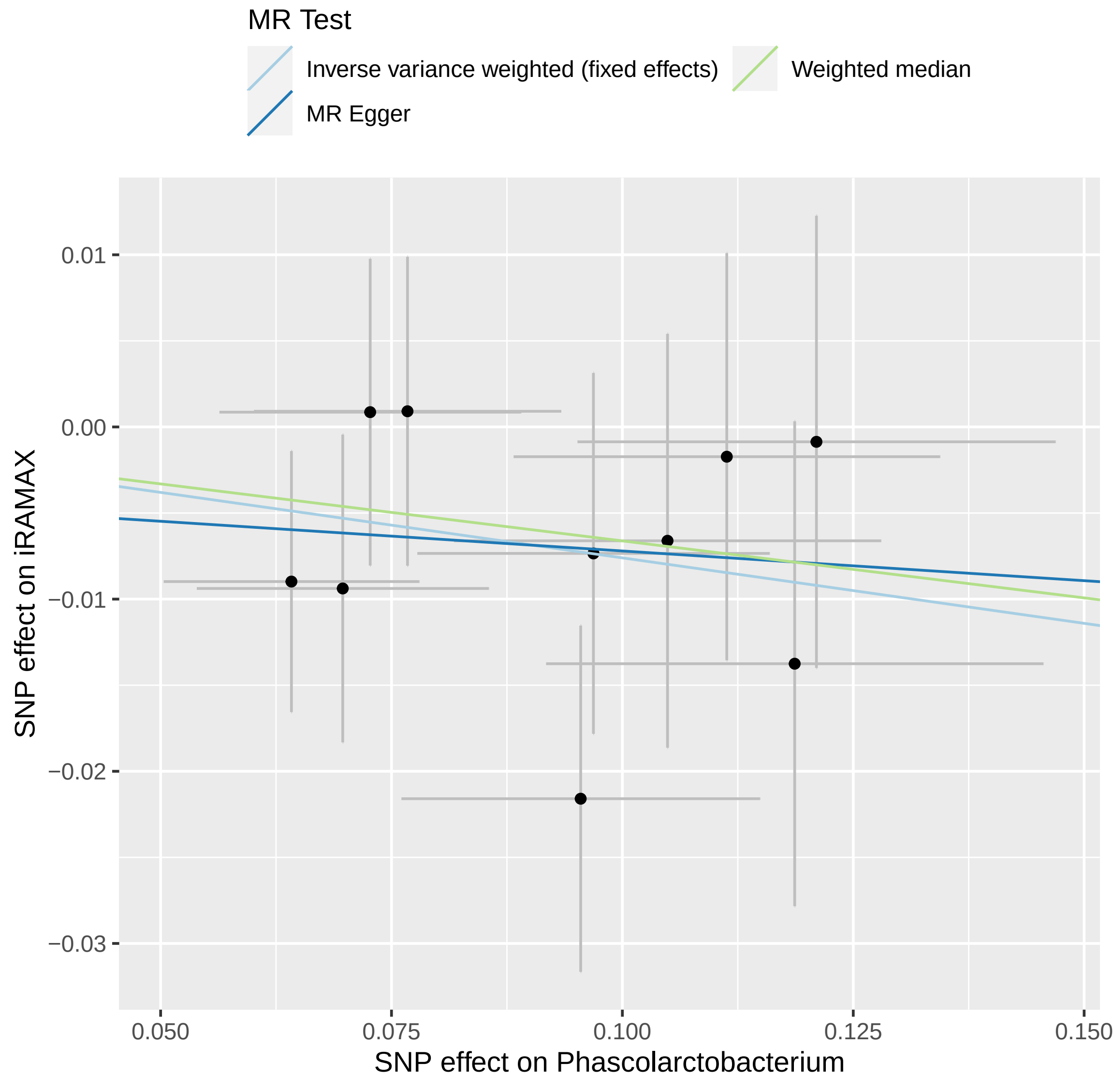

MR Test
Inverse variance weighted (fixed effects)
Weighted median
MR Egger
0.01
0.00
−0.01
−0.02
−0.03
SNP effect on iRAMAX
0.050
0.075
0.100
0.125
0.150
SNP effect on Phascolarctobacterium

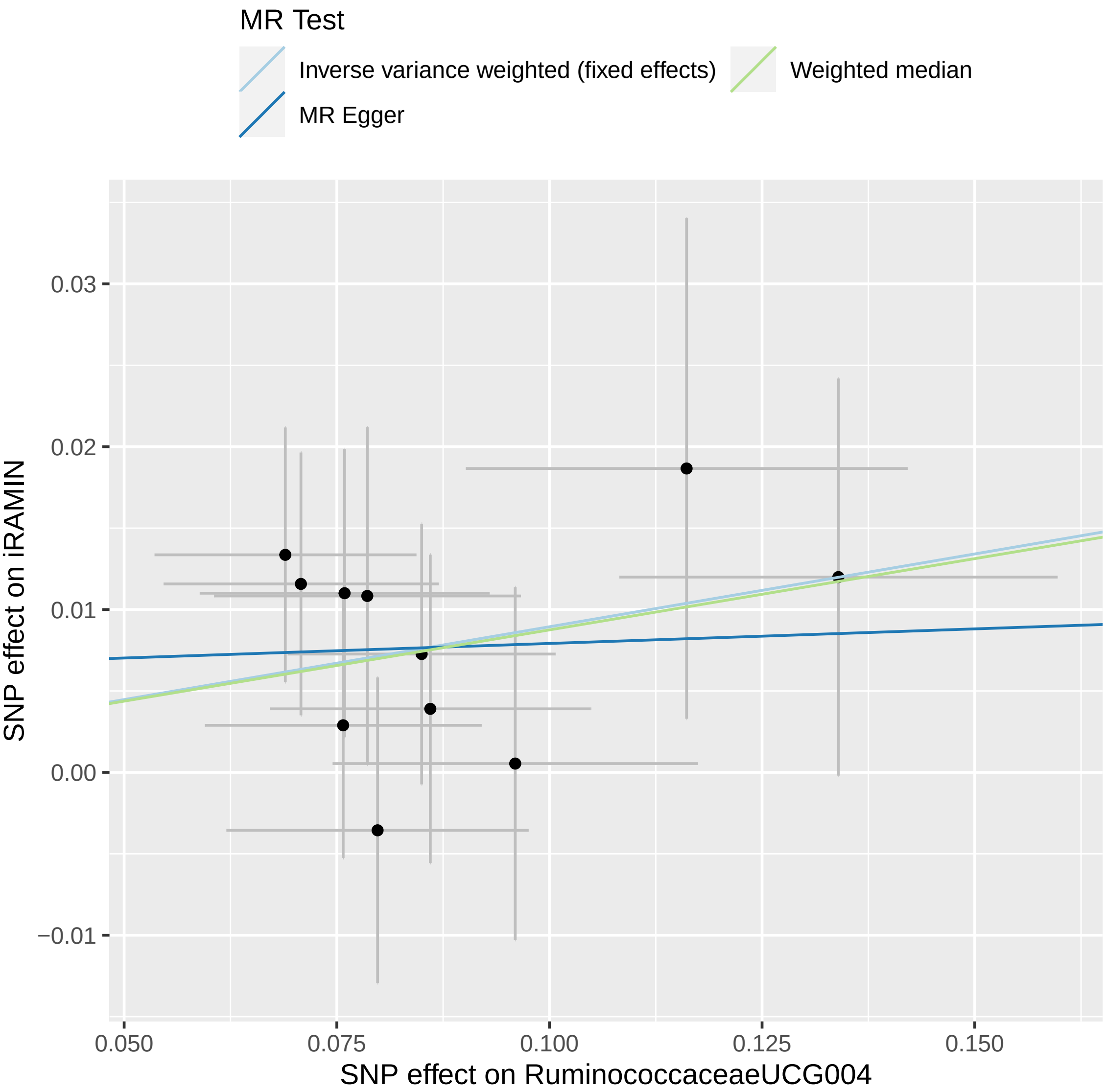

MR Test
Inverse variance weighted (fixed effects)
Weighted median
MR Egger
0.03
0.02
0.01
0.00
−0.01
0.050
0.075
0.100
0.125
0.150
SNP effect on iRAMIN
SNP effect on RuminococcaceaeUCG004

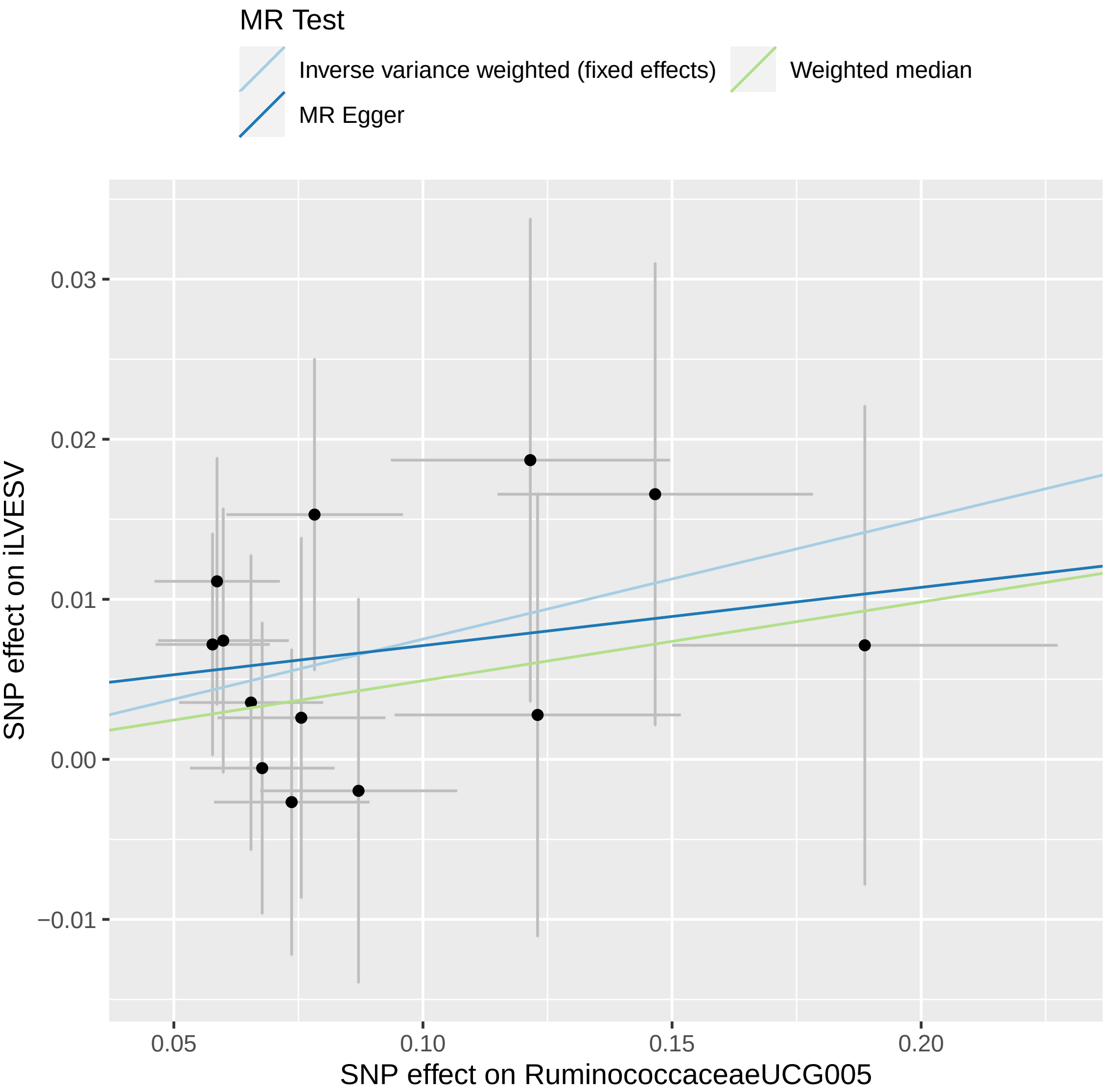
MR Test
Inverse variance weighted (fixed effects)
Weighted median
MR Egger
SNP effect on iLVESV
0.03
0.02
0.01
0.00
−0.01
0.05
0.10
0.15
0.20
SNP effect on RuminococcaceaeUCG005

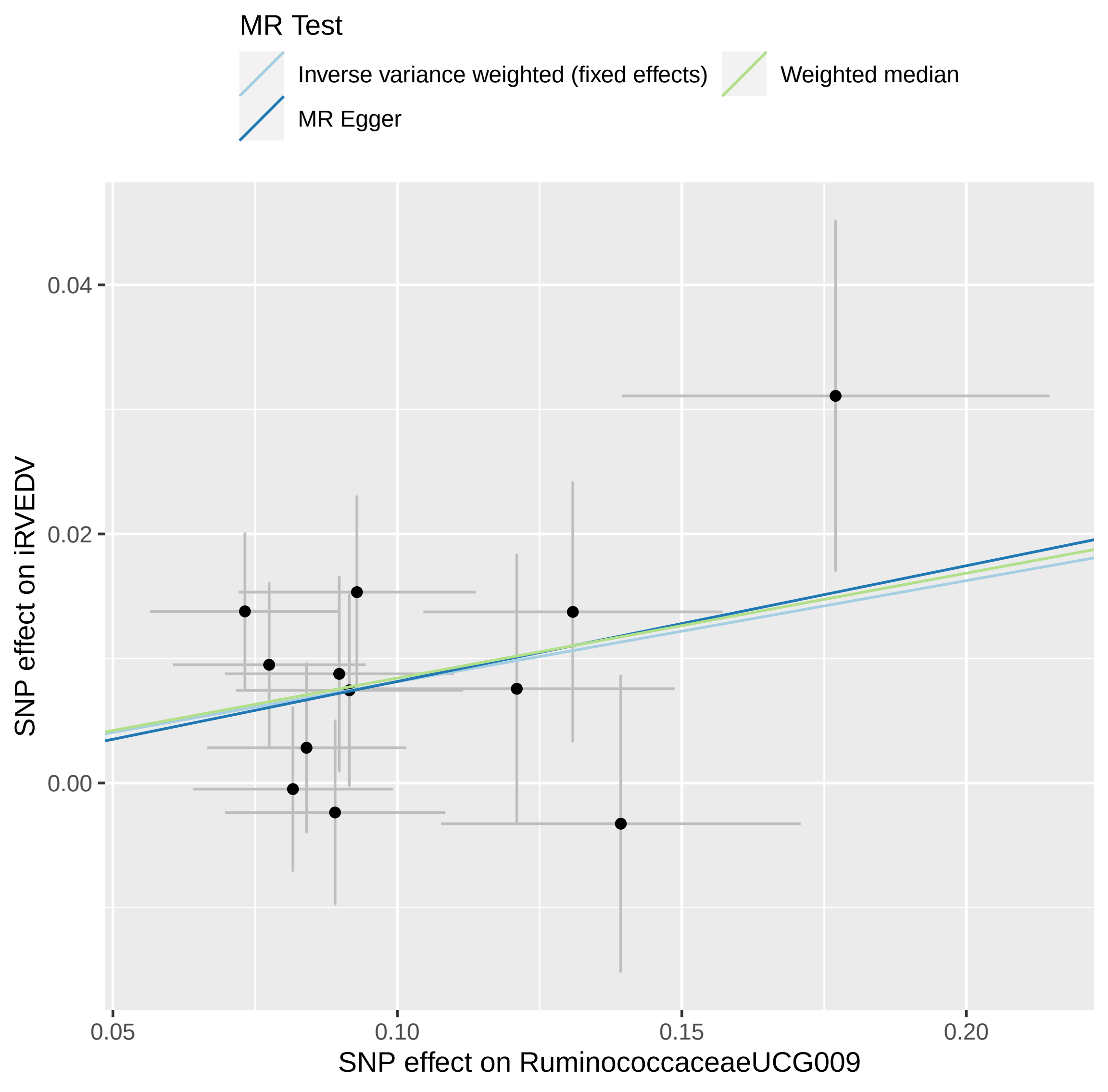
MR Test
Inverse variance weighted (fixed effects)
MR Egger
Weighted median
0.04
0.02
0.00
0.05
0.10
0.15
0.20
SNP effect on iRVEDV
SNP effect on RuminococcaceaeUCG009

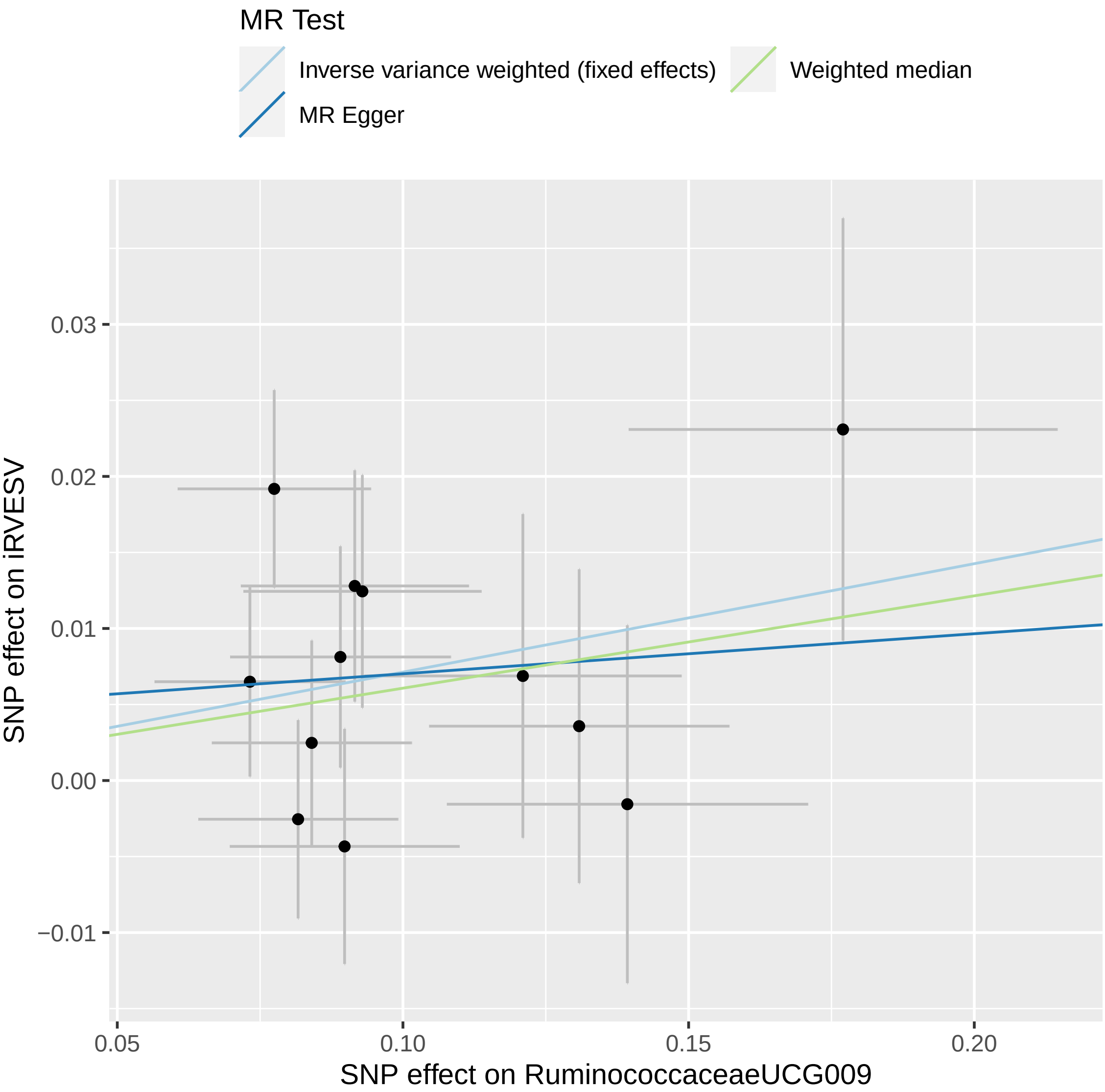
MR Test
Inverse variance weighted (fixed effects)
Weighted median
MR Egger
0.03
0.02
0.01
0.00
−0.01
SNP effect on iRVESV
0.05
0.10
0.15
0.20
SNP effect on RuminococcaceaeUCG009

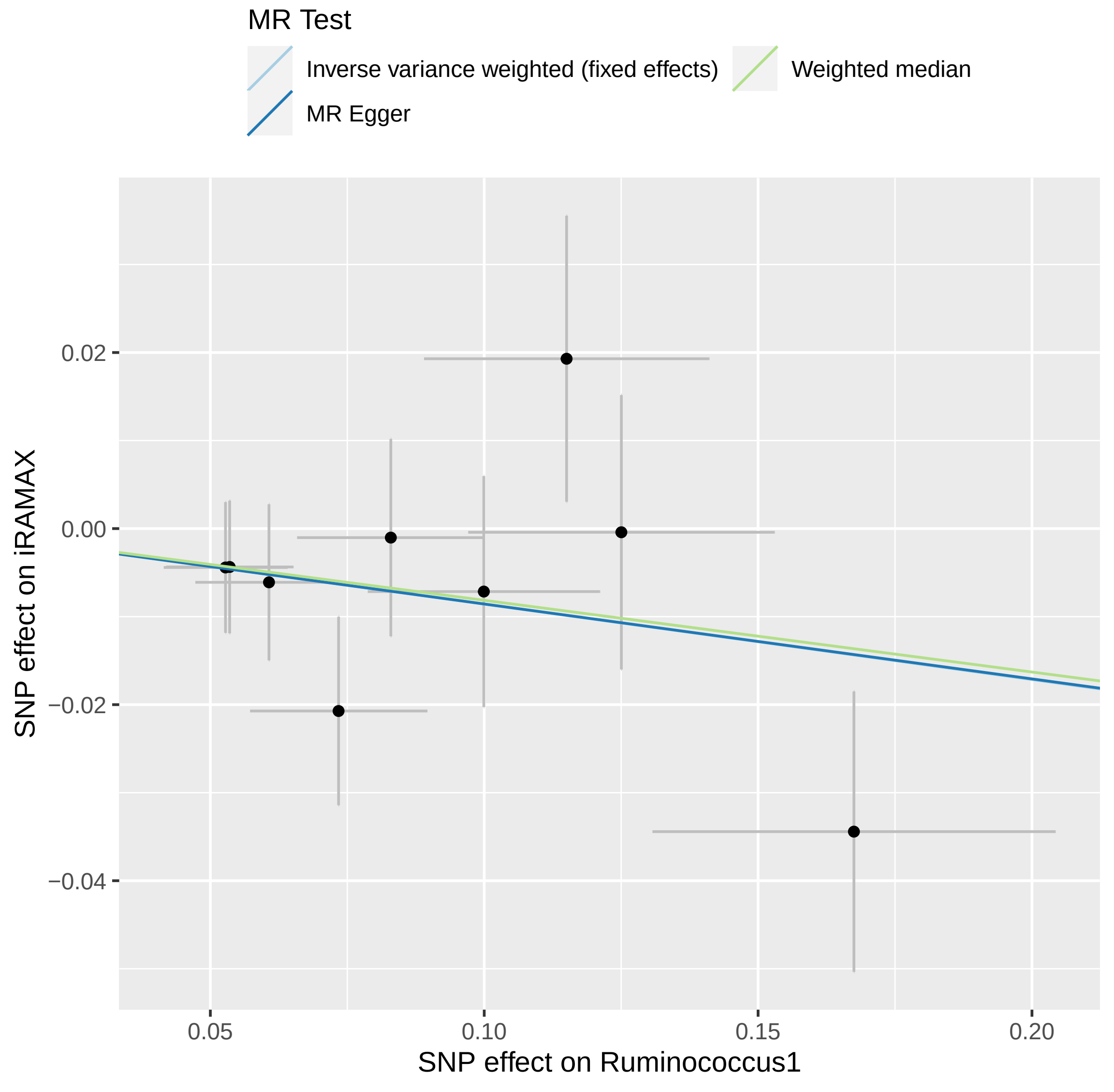
MR Test
Inverse variance weighted (fixed effects)
Weighted median
MR Egger
0.02
0.00
−0.02
−0.04
0.05
0.10
0.15
0.20
SNP effect on iRAMAX
SNP effect on Ruminococcus1

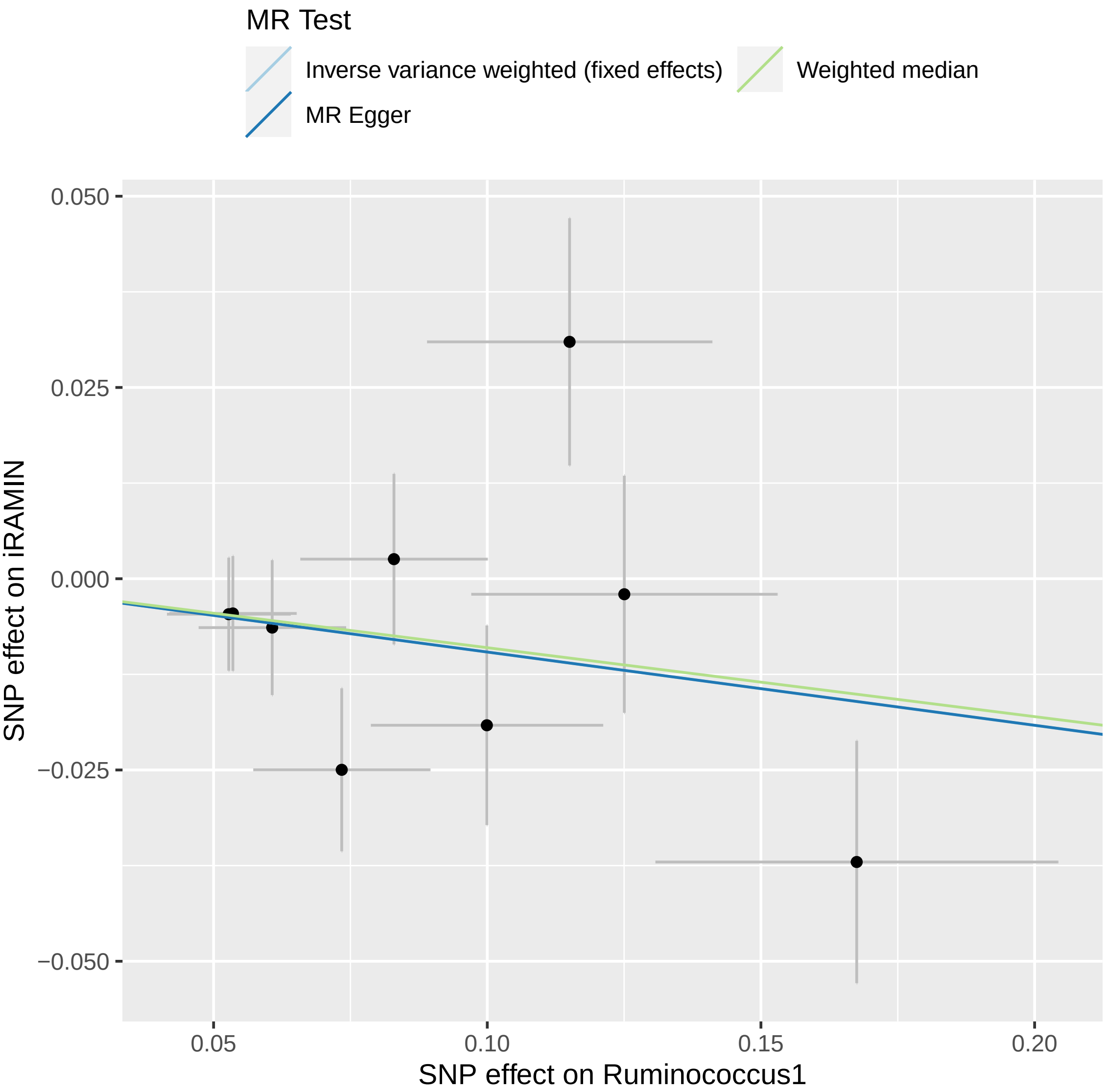
MR Test
Inverse variance weighted (fixed effects)
MR Egger
Weighted median
SNP effect on iRAMIN
0.050
0.025
0.000
−0.025
−0.050
SNP effect on Ruminococcus1
0.05
0.10
0.15
0.20

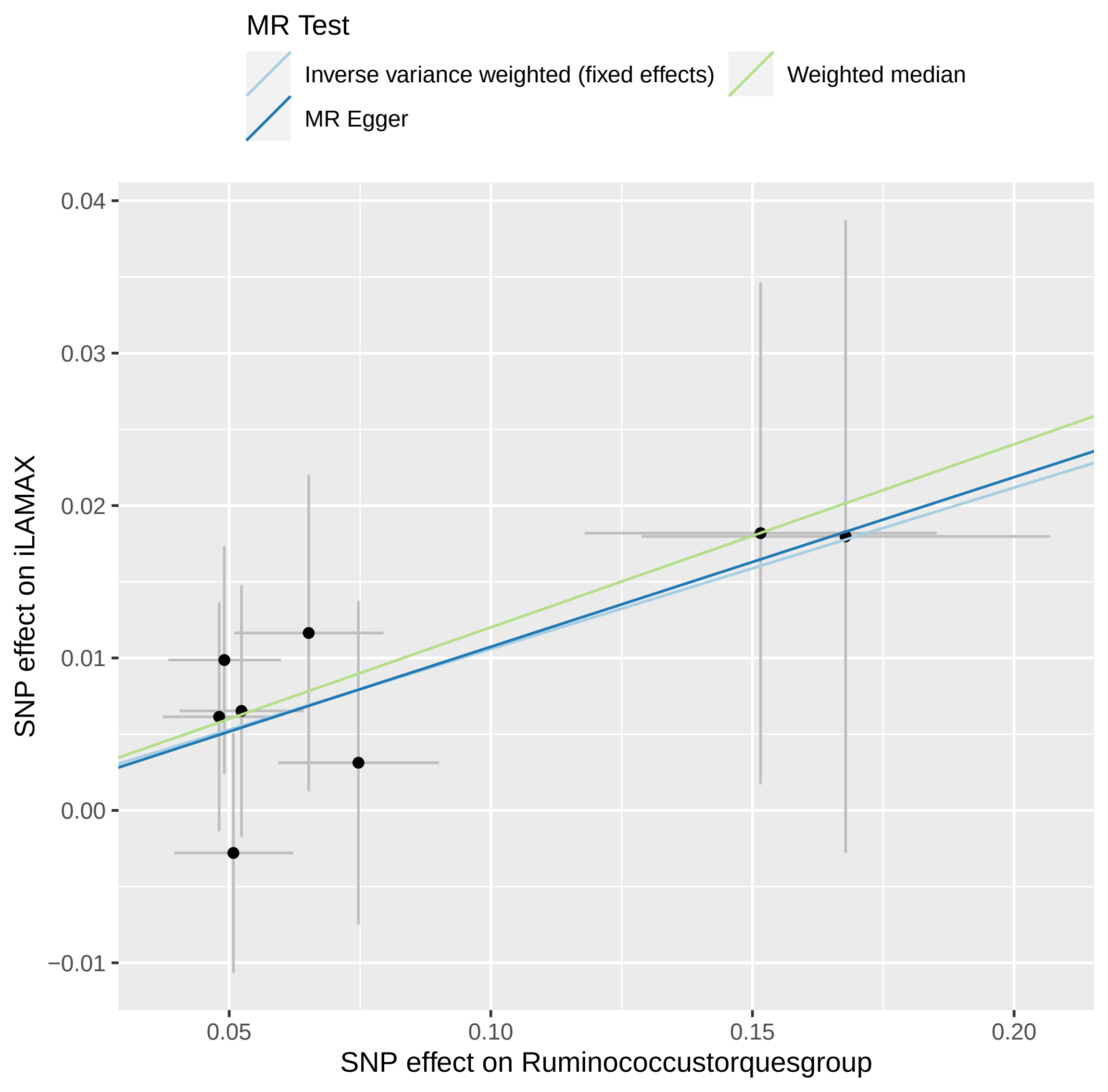
MR Test
Inverse variance weighted (fixed effects)
Weighted median
MR Egger
0.04
0.03
0.02
0.01
0.00
−0.01
0.05
0.10
0.15
0.20
SNP effect on iLAMAX
SNP effect on Ruminococcustorquesgroup

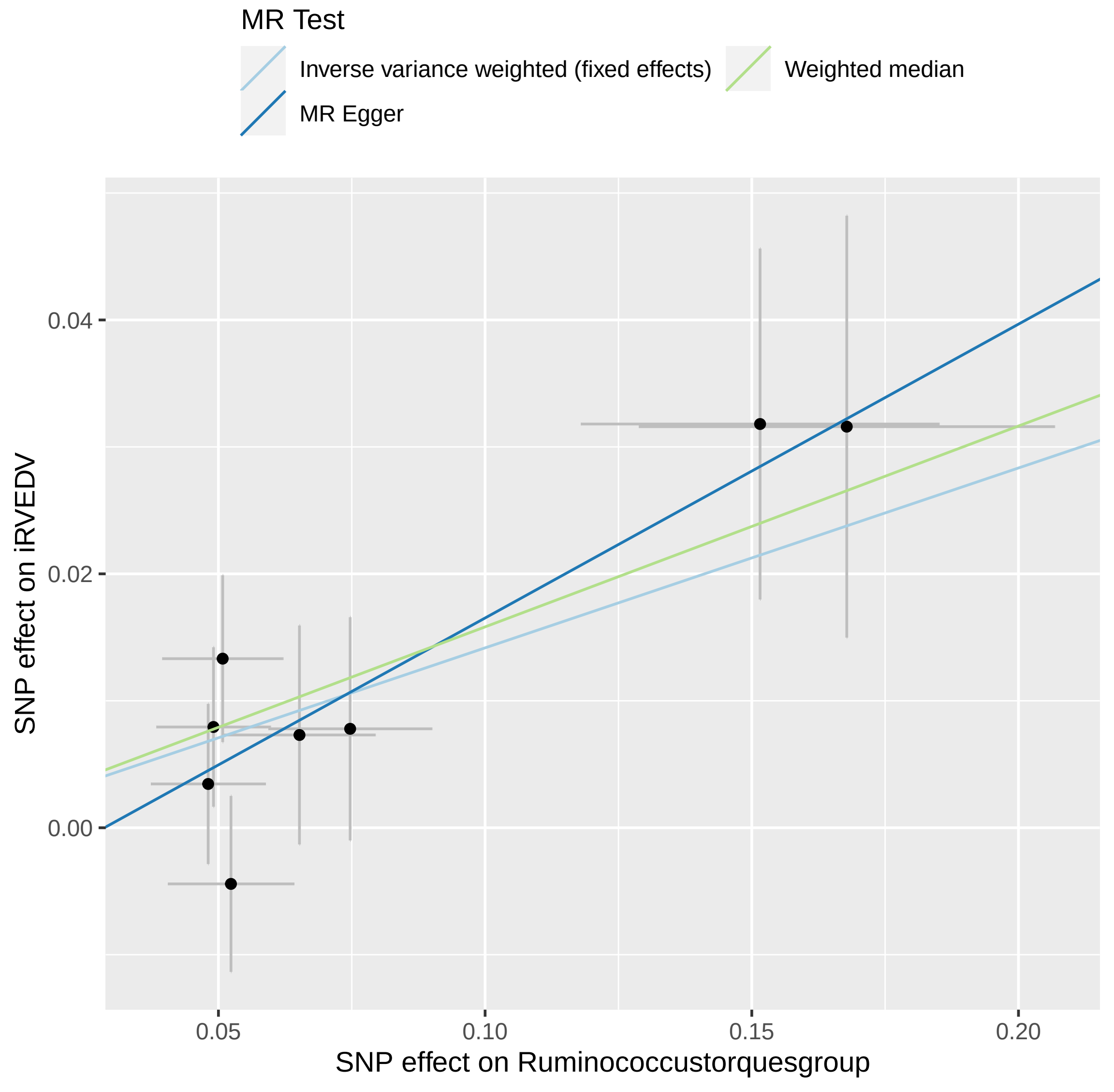

MR Test
Inverse variance weighted (fixed effects)
MR Egger
Weighted median
SNP effect on iRVEDV
0.04
0.02
0.00
0.05
0.10
0.15
0.20
SNP effect on Ruminococcustorquesgroup

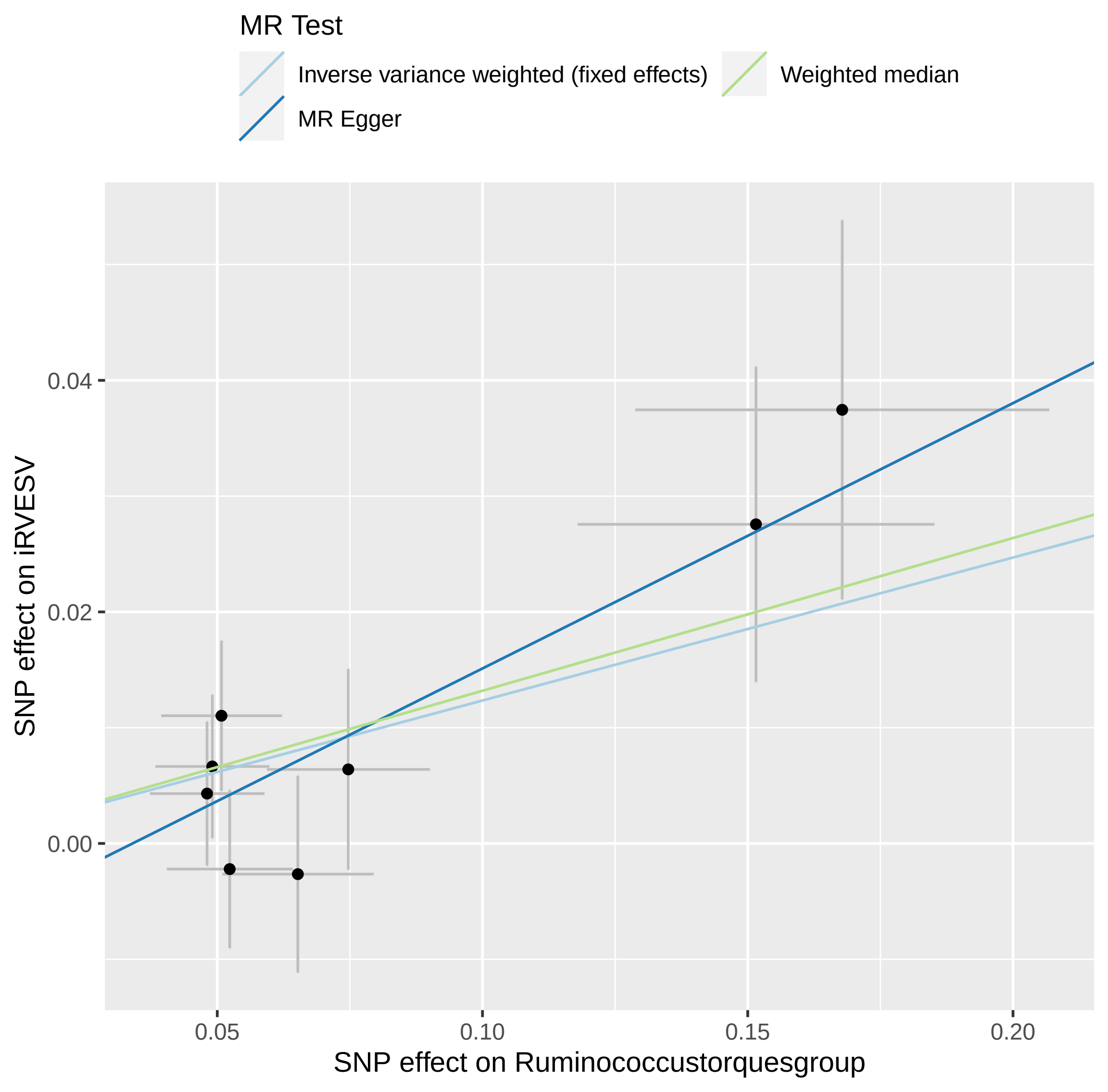
MR Test
Inverse variance weighted (fixed effects)
MR Egger
Weighted median
SNP effect on iRVESV
0.04
0.02
0.00
0.05
0.10
0.15
0.20
SNP effect on Ruminococcustorquesgroup

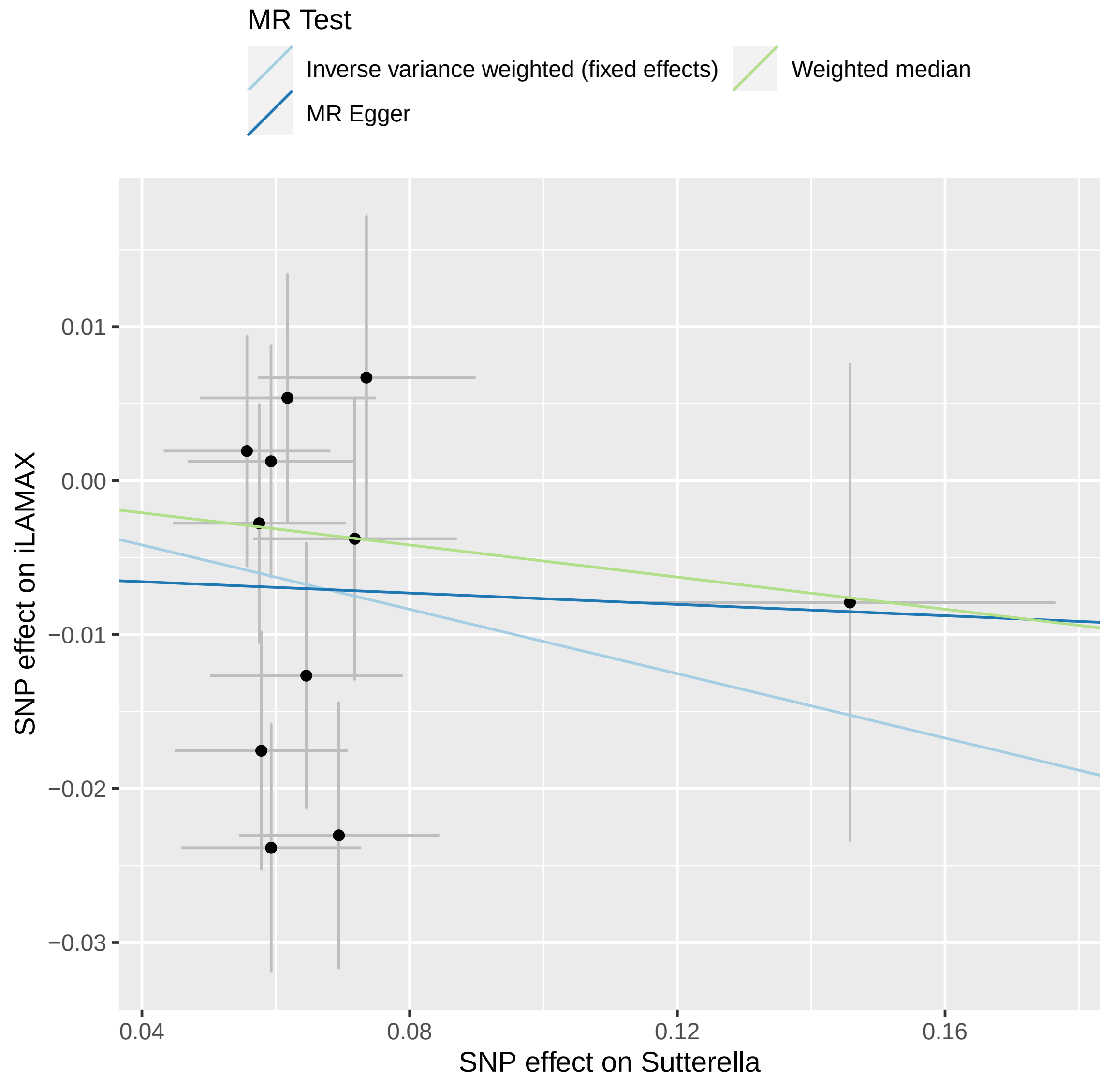
MR Test
Inverse variance weighted (fixed effects)
MR Egger
Weighted median
SNP effect on iLAMAX
0.01
0.00
−0.01
−0.02
−0.03
0.04
0.08
0.12
0.16
SNP effect on Sutterella

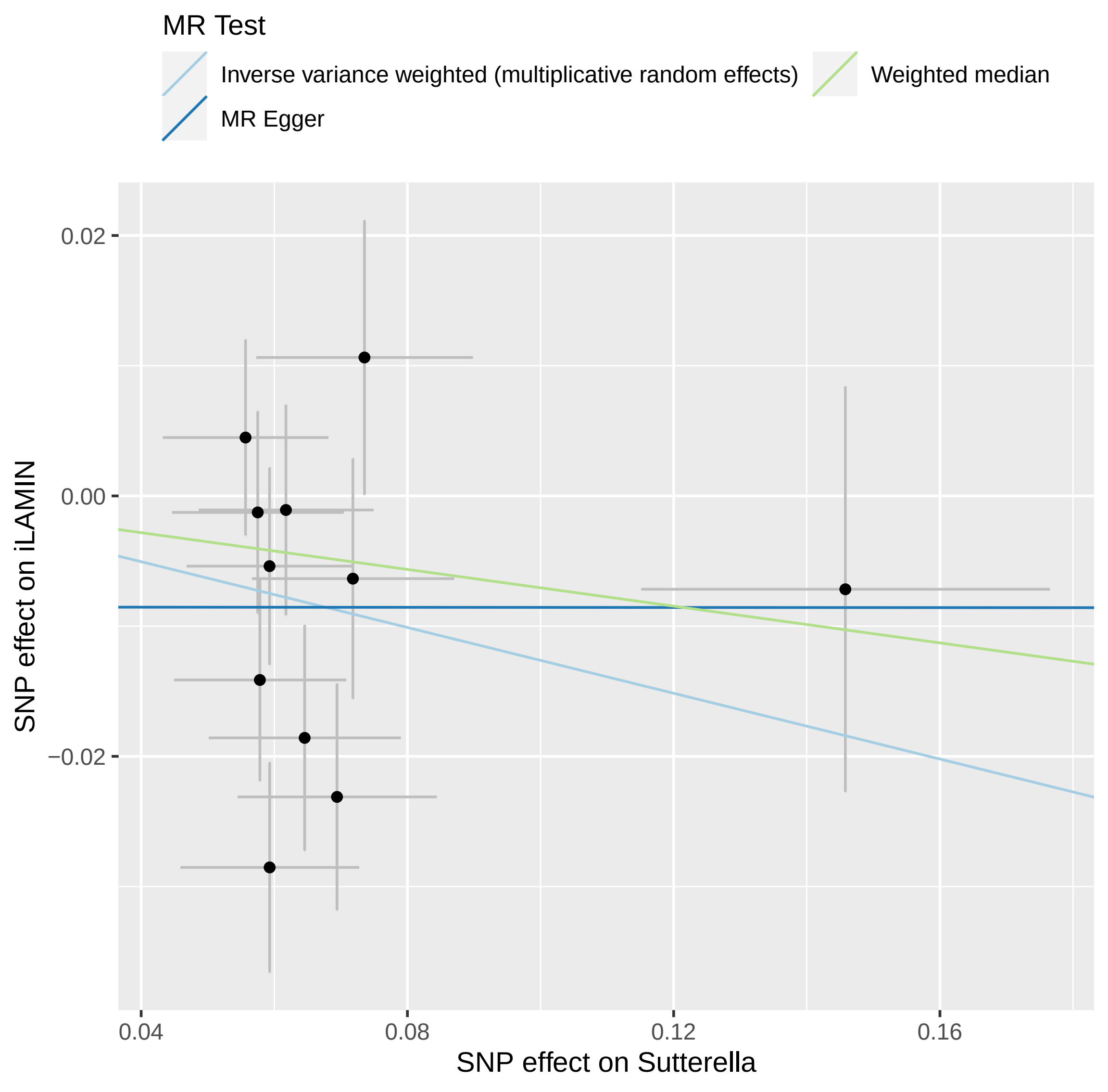

MR Test
Inverse variance weighted (multiplicative random effects)
Weighted median
MR Egger
0.02
0.00
−0.02
0.04
0.08
0.12
0.16
SNP effect on iLAMIN
SNP effect on Sutterella

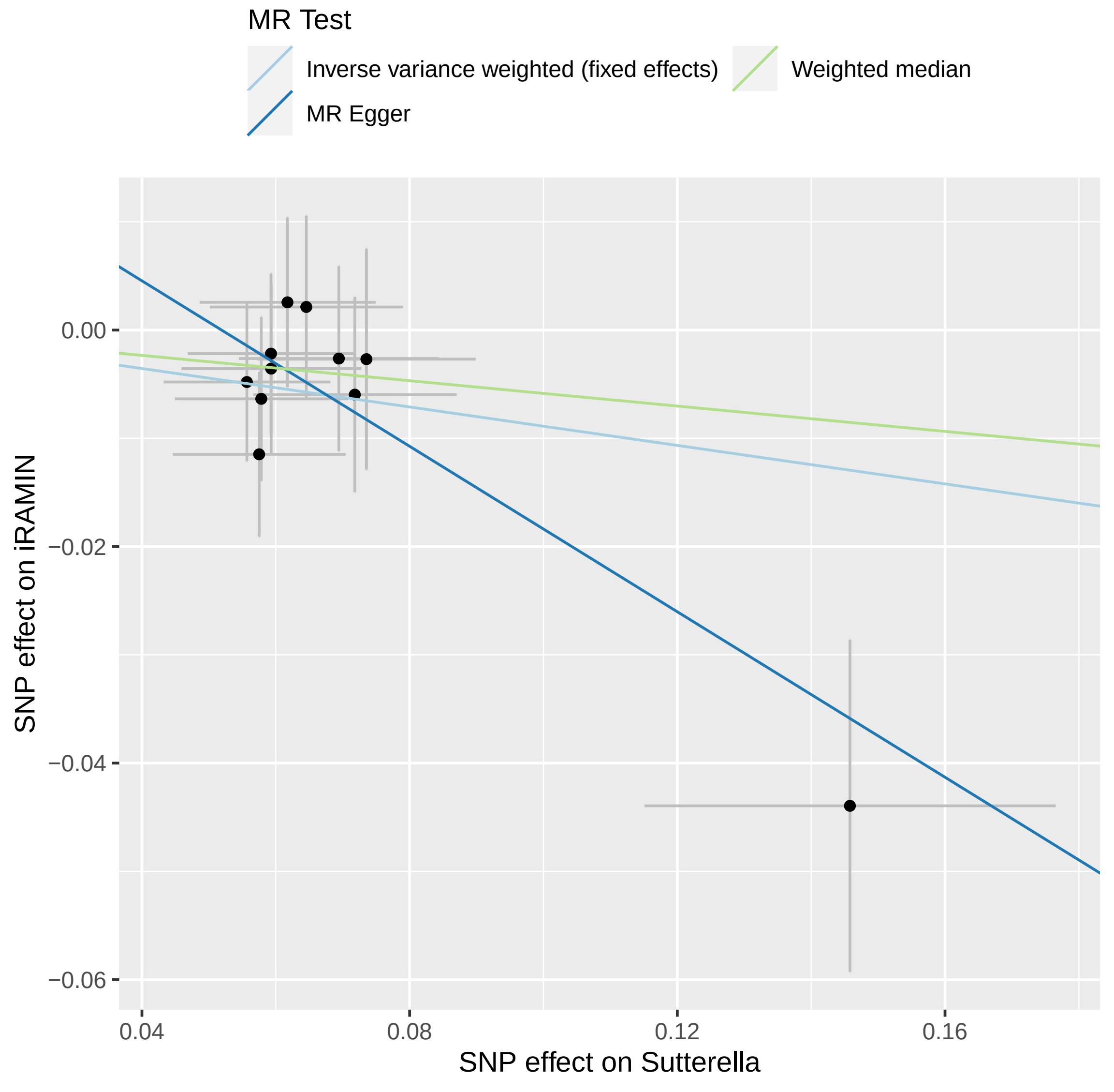
MR Test
Inverse variance weighted (fixed effects)
Weighted median
MR Egger
0.00
−0.02
−0.04
−0.06
0.04
0.08
0.12
0.16
SNP effect on Sutterella
SNP effect on iRAMIN

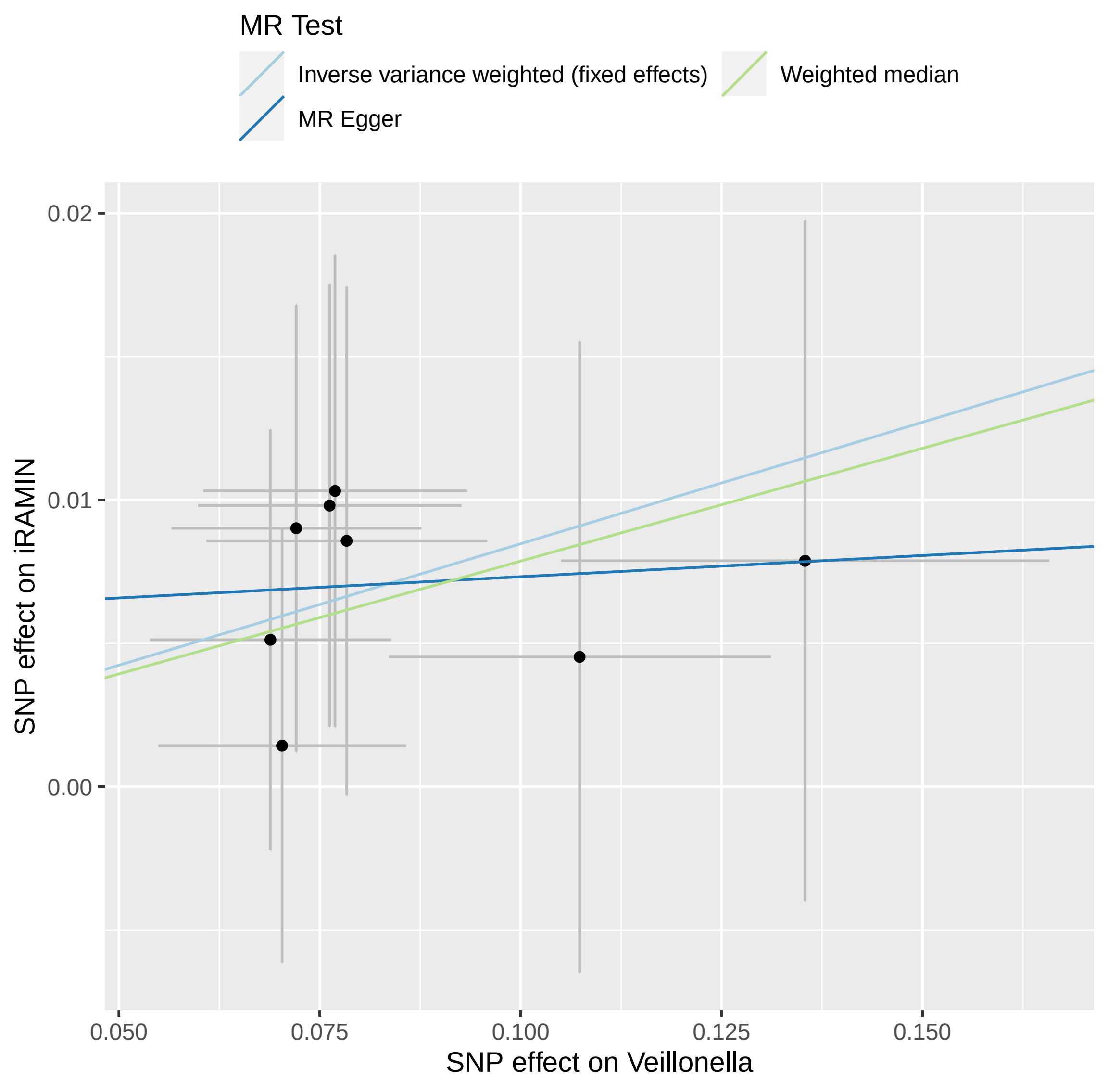
MR Test
Inverse variance weighted (fixed effects)
Weighted median
MR Egger
SNP effect on iRAMIN
0.02
0.01
0.00
0.050
0.075
0.100
0.125
0.150
SNP effect on Veillonella
