# Supplementary material for: Gut microbiota as a residual risk factor causally influencing cardiac structure and function: Mendelian randomization analysis and biological annotation
Source: Front Microbiol. 2024 Jul 26;15:1410272. doi: 10.3389/fmicb.2024.1410272 (PMC11316272; doi:10.3389/fmicb.2024.1410272)
Supplement: SUPPLEMENTARY FIGURE S4 — Scatter plots for gut microbiota on cardiac function. [file Data_Sheet_4.PDF]

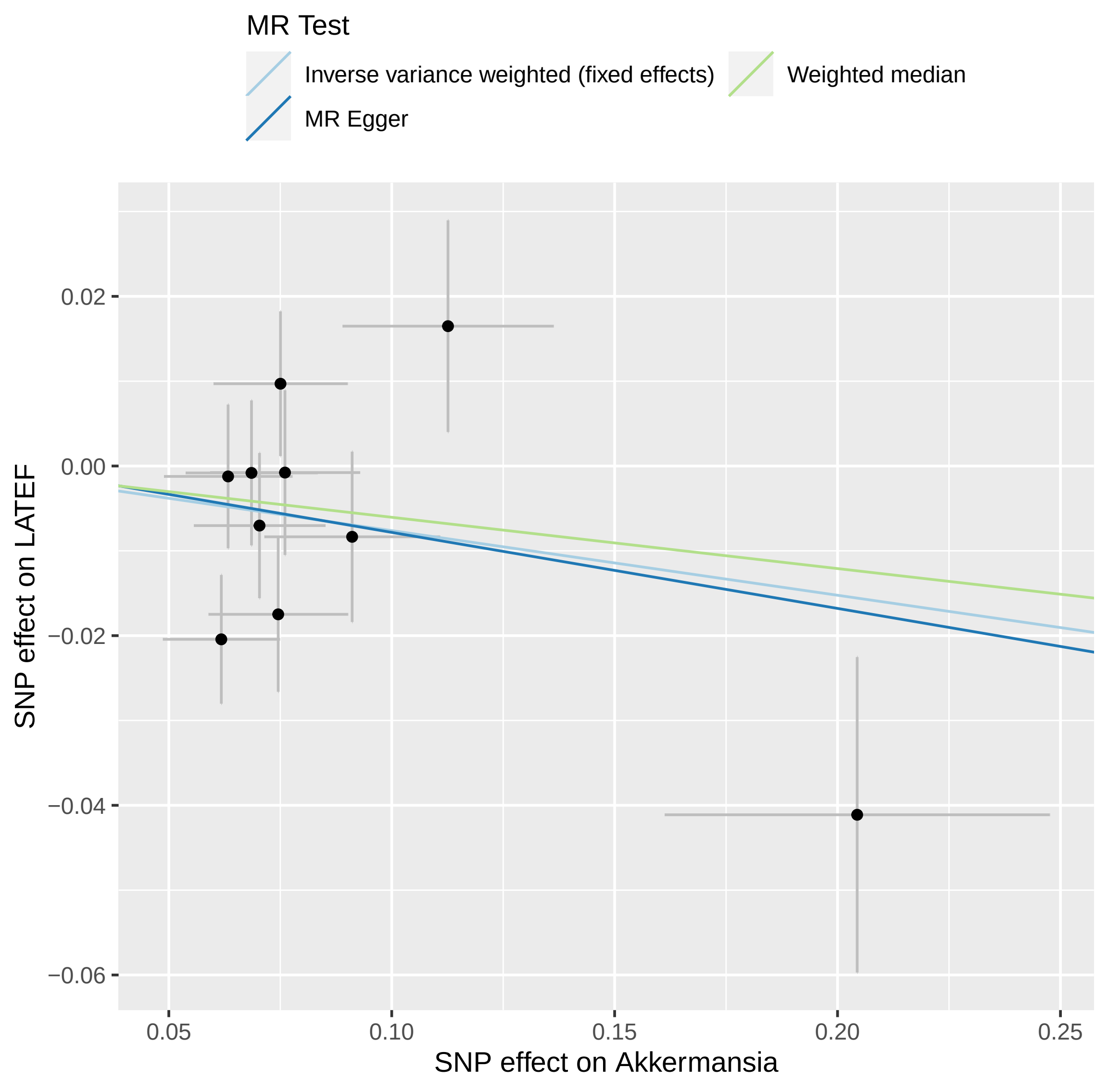
MR Test
Inverse variance weighted (fixed effects)
Weighted median
MR Egger
0.02
0.00
−0.02
−0.04
−0.06
0.05
0.10
0.15
0.20
0.25
SNP effect on LATEF
SNP effect on Akkermansia

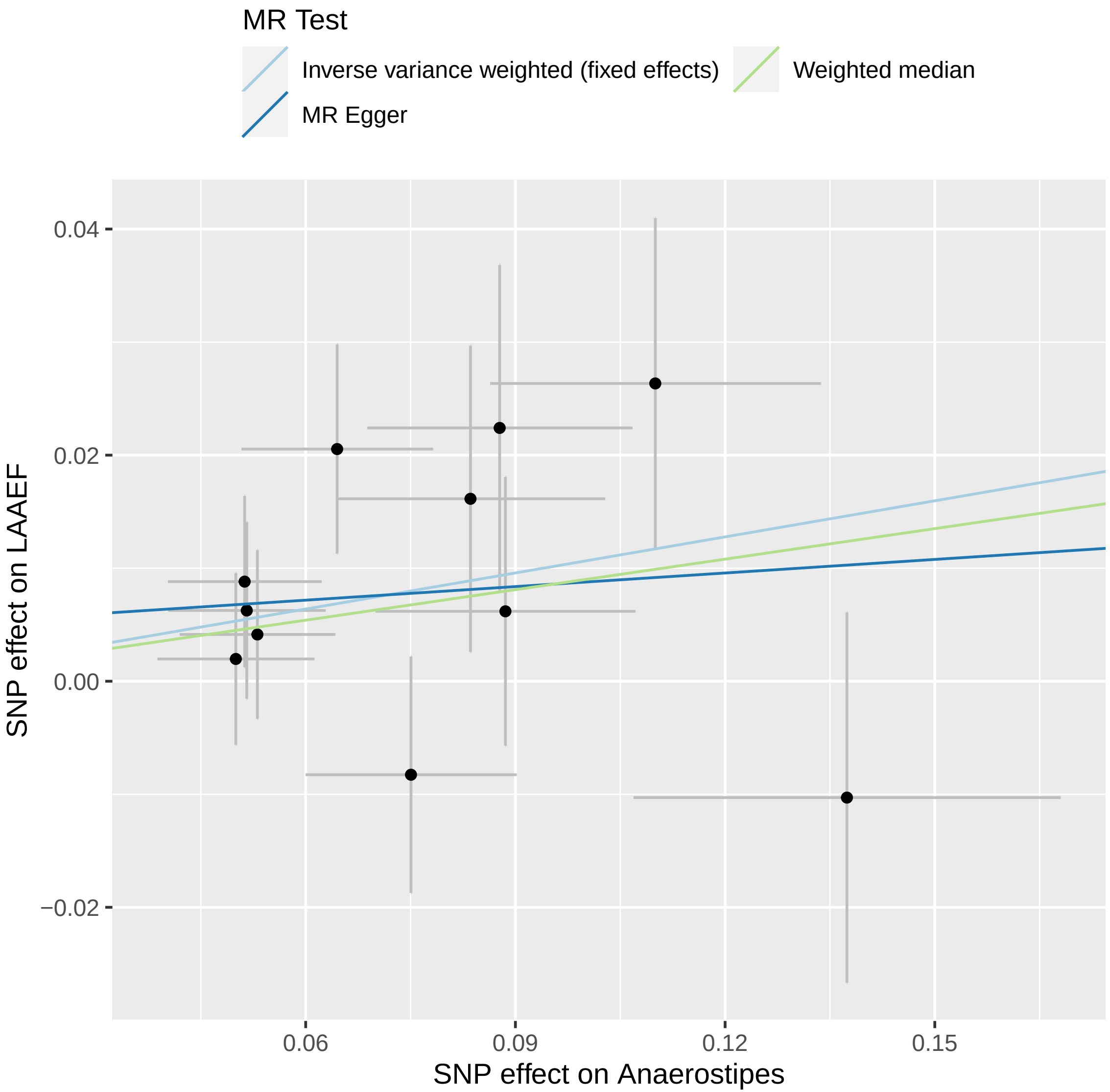

MR Test
Inverse variance weighted (fixed effects)
Weighted median
MR Egger
0.04
0.02
0.00
−0.02
SNP effect on LAAEF
0.06
0.09
0.12
0.15
SNP effect on Anaerostipes

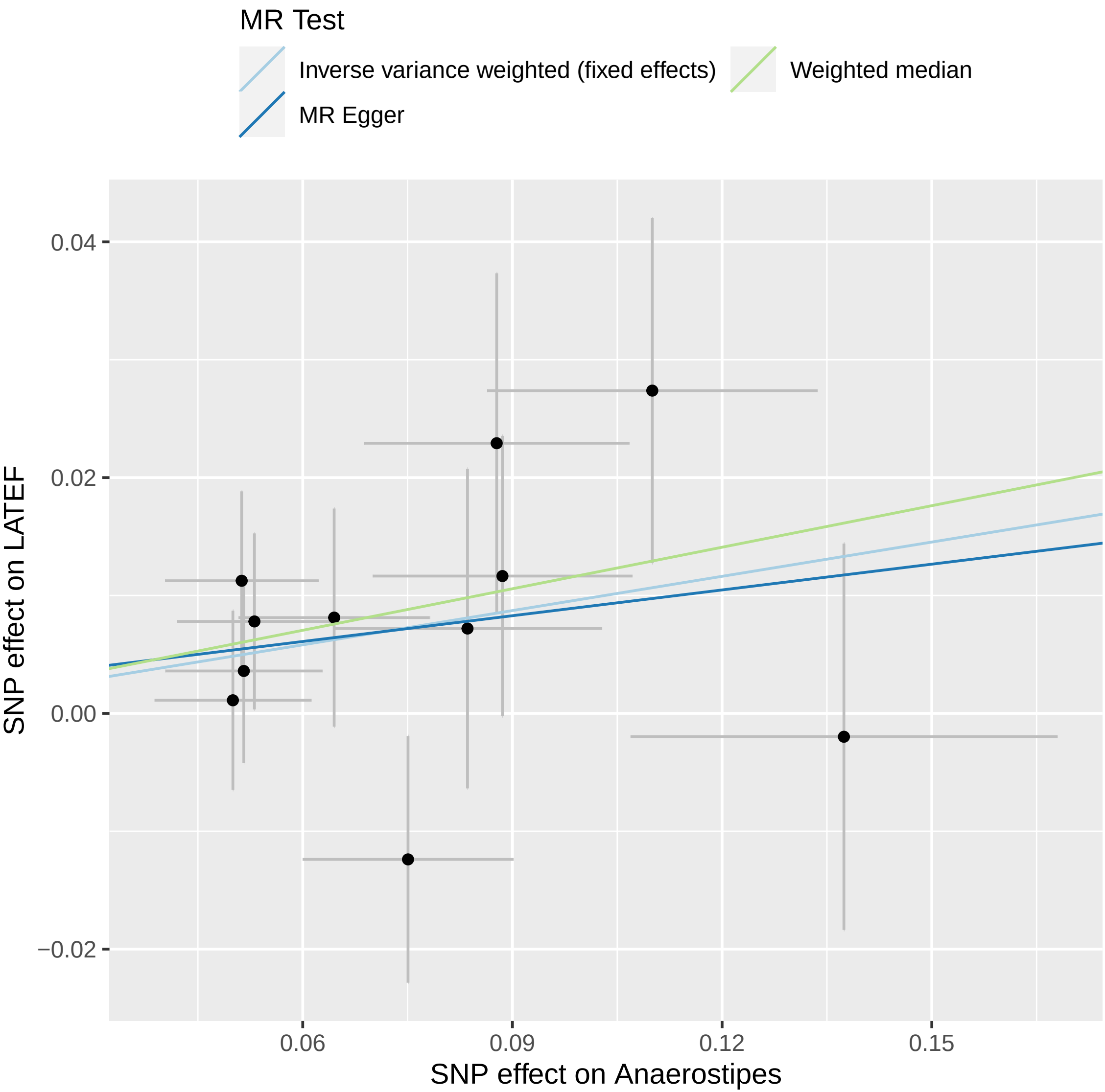
MR Test
Inverse variance weighted (fixed effects)
Weighted median
MR Egger
0.04
0.02
0.00
−0.02
0.06
0.09
0.12
0.15
SNP effect on LATEF
SNP effect on Anaerostipes

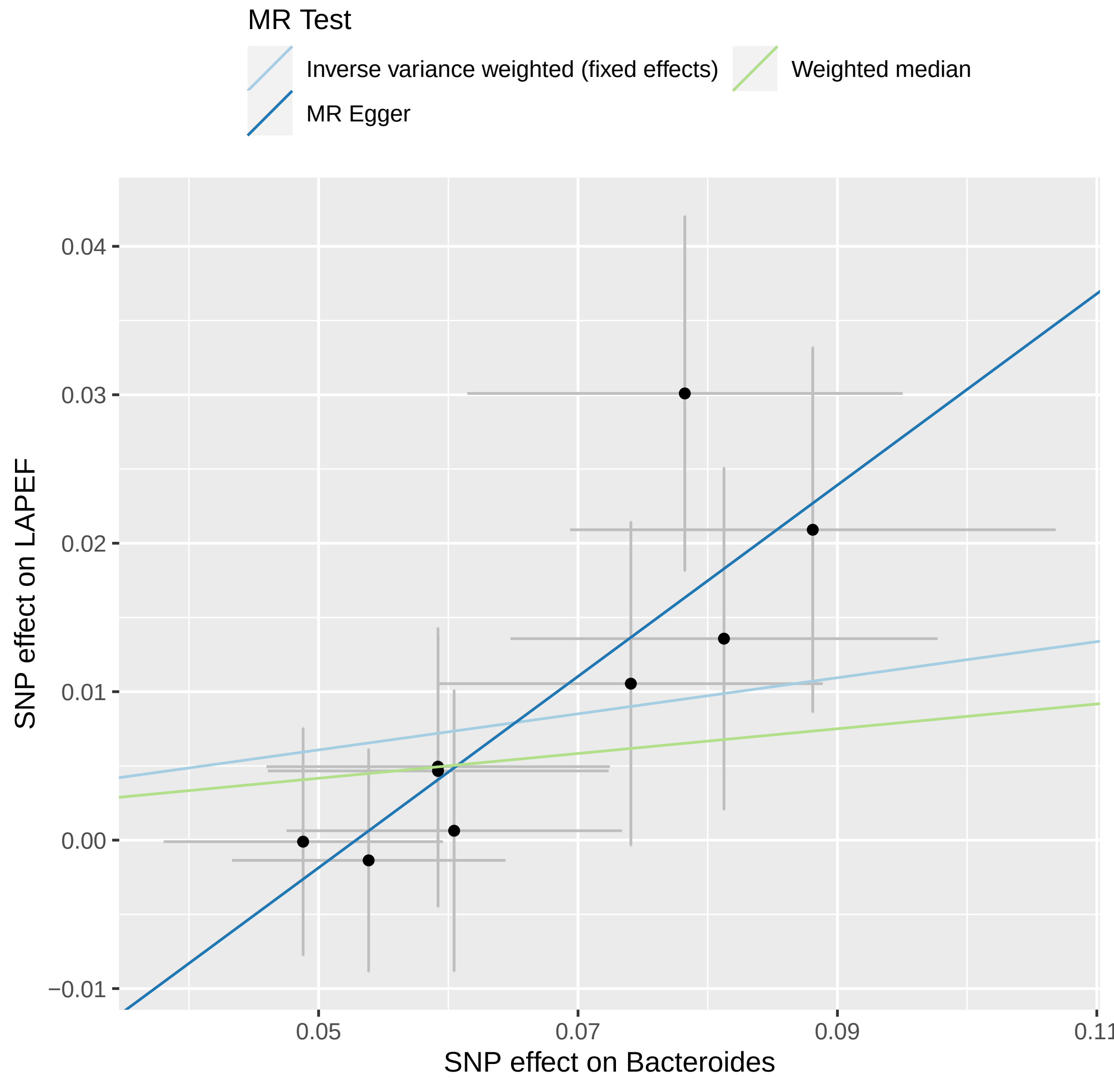

MR Test
Inverse variance weighted (fixed effects)
Weighted median
MR Egger
SNP effect on LAPEF
0.04
0.03
0.02
0.01
0.00
−0.01
0.05
0.07
0.09
0.11
SNP effect on Bacteroides

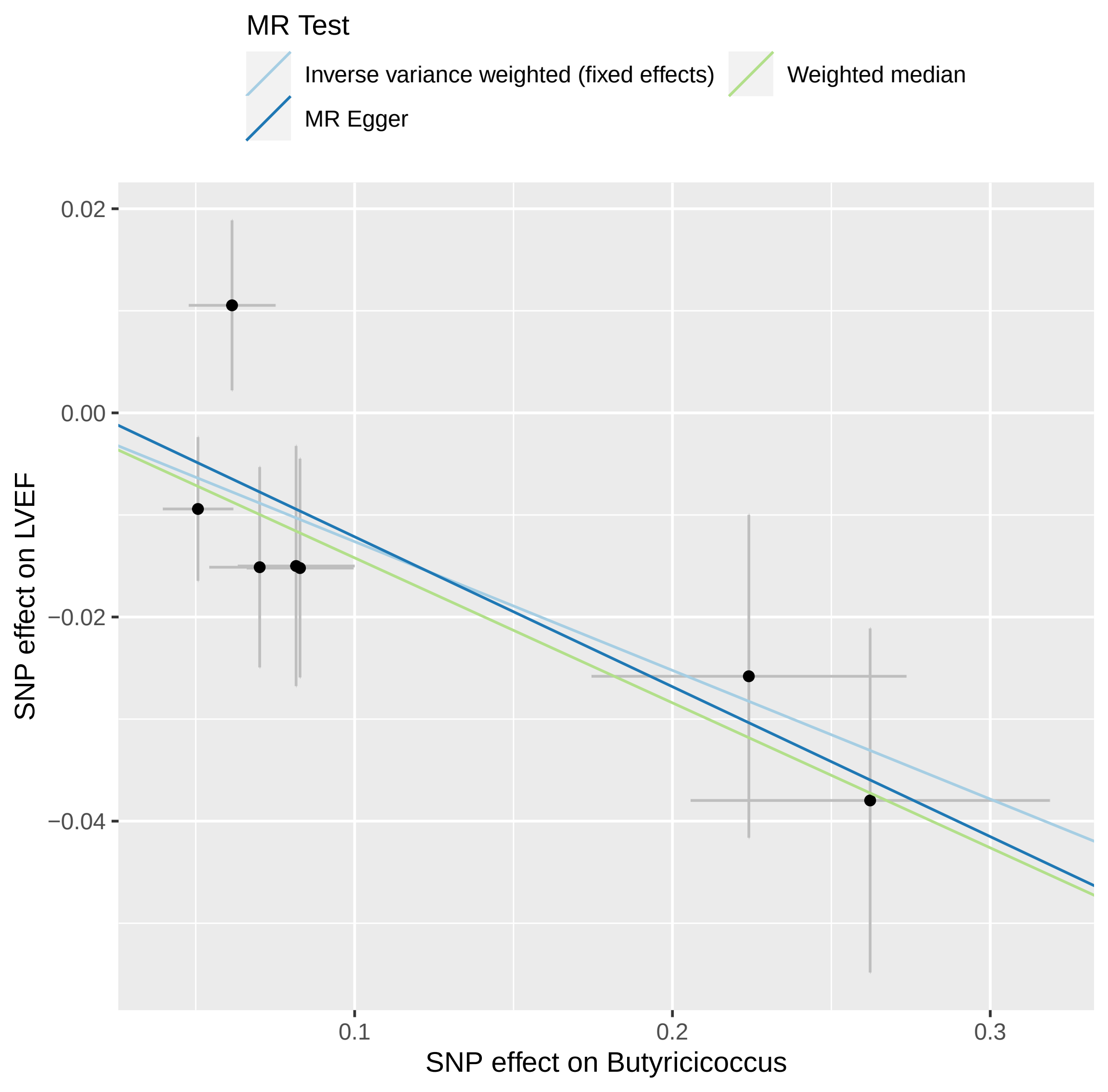
MR Test
Inverse variance weighted (fixed effects)
Weighted median
MR Egger
0.02
0.00
−0.02
−0.04
0.1
0.2
0.3
SNP effect on LVEF
SNP effect on Butyricicoccus

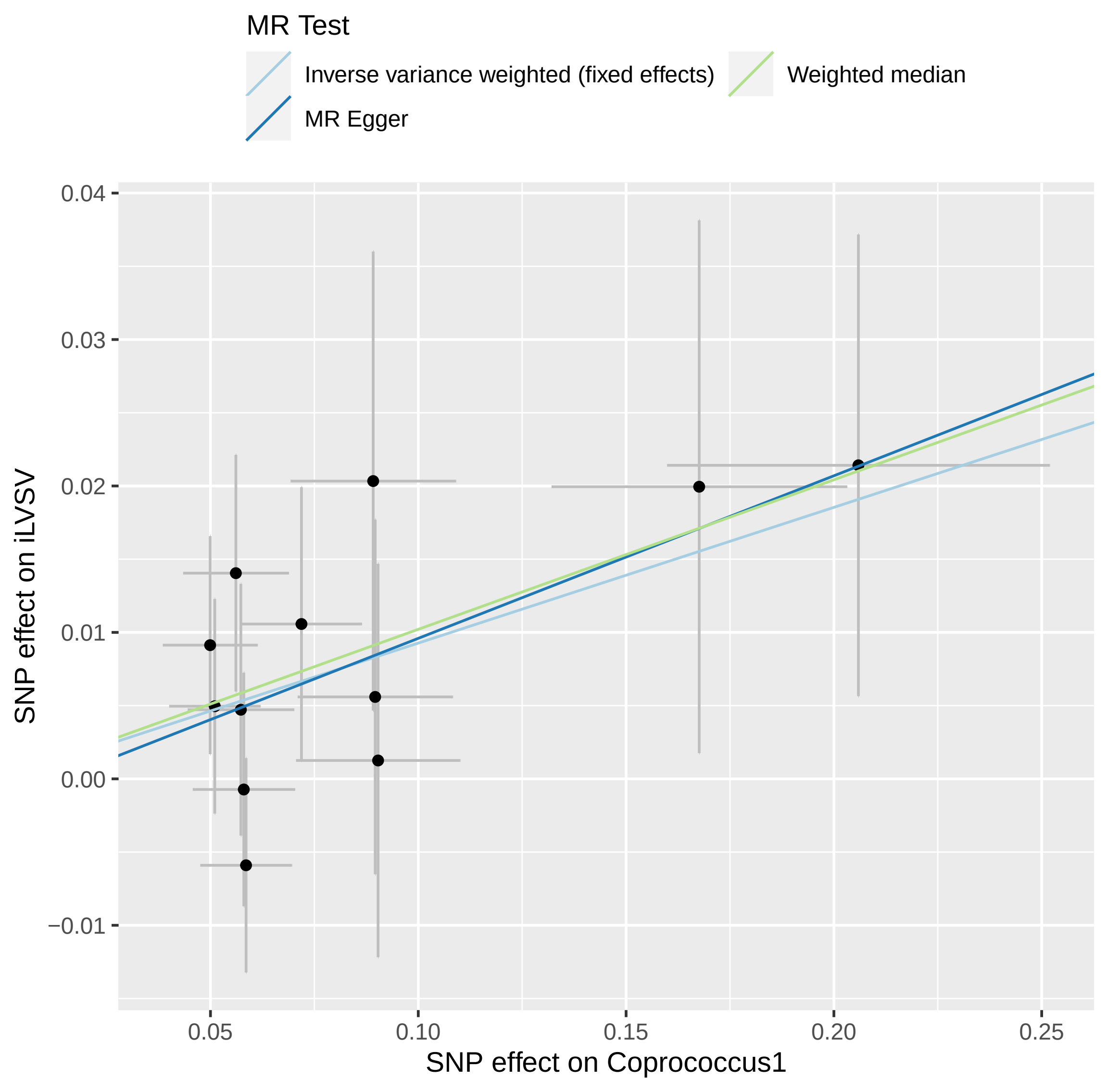
MR Test
Inverse variance weighted (fixed effects)
Weighted median
MR Egger
SNP effect on iLVSV
0.04
0.03
0.02
0.01
0.00
−0.01
0.05
0.10
0.15
0.20
0.25
SNP effect on Coprococcus1

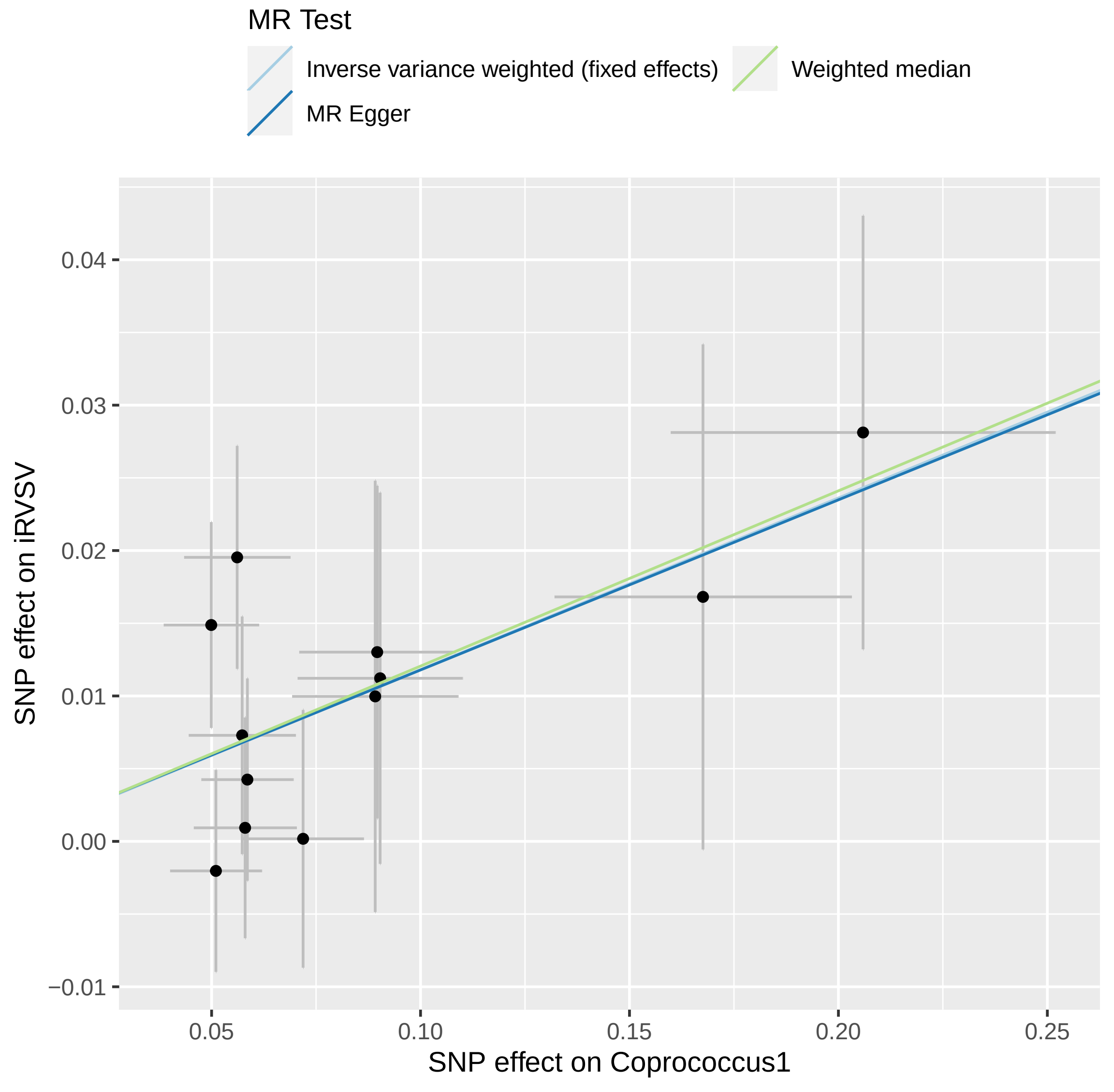

MR Test
Inverse variance weighted (fixed effects)
MR Egger
Weighted median
0.04
0.03
0.02
0.01
0.00
−0.01
SNP effect on iRVSV
0.05
0.10
0.15
0.20
0.25
SNP effect on Coprococcus1

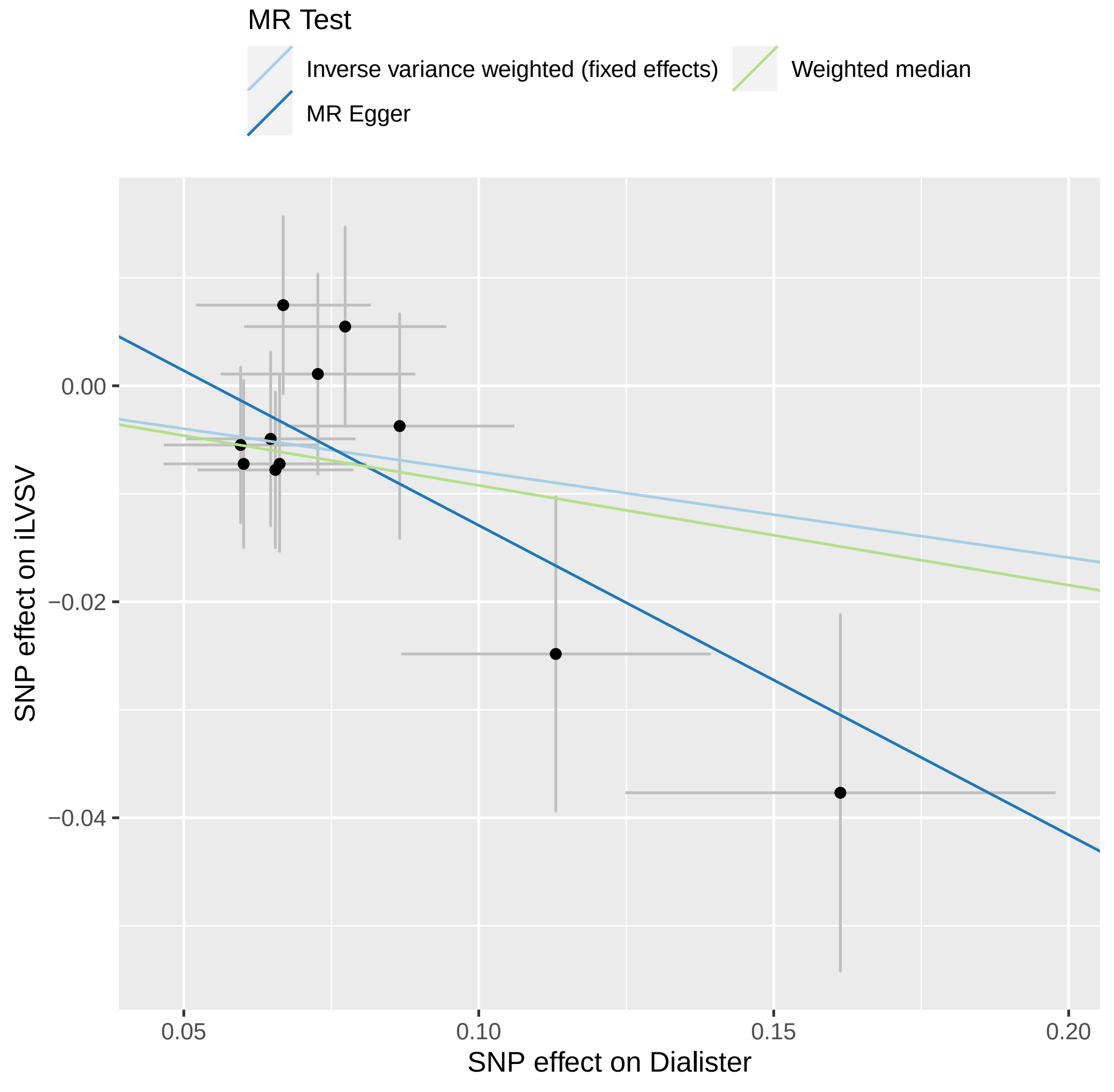
MR Test
Inverse variance weighted (fixed effects)
Weighted median
MR Egger
SNP effect on iLVSV
0.00
−0.02
−0.04
0.05
0.10
0.15
0.20
SNP effect on Dialister

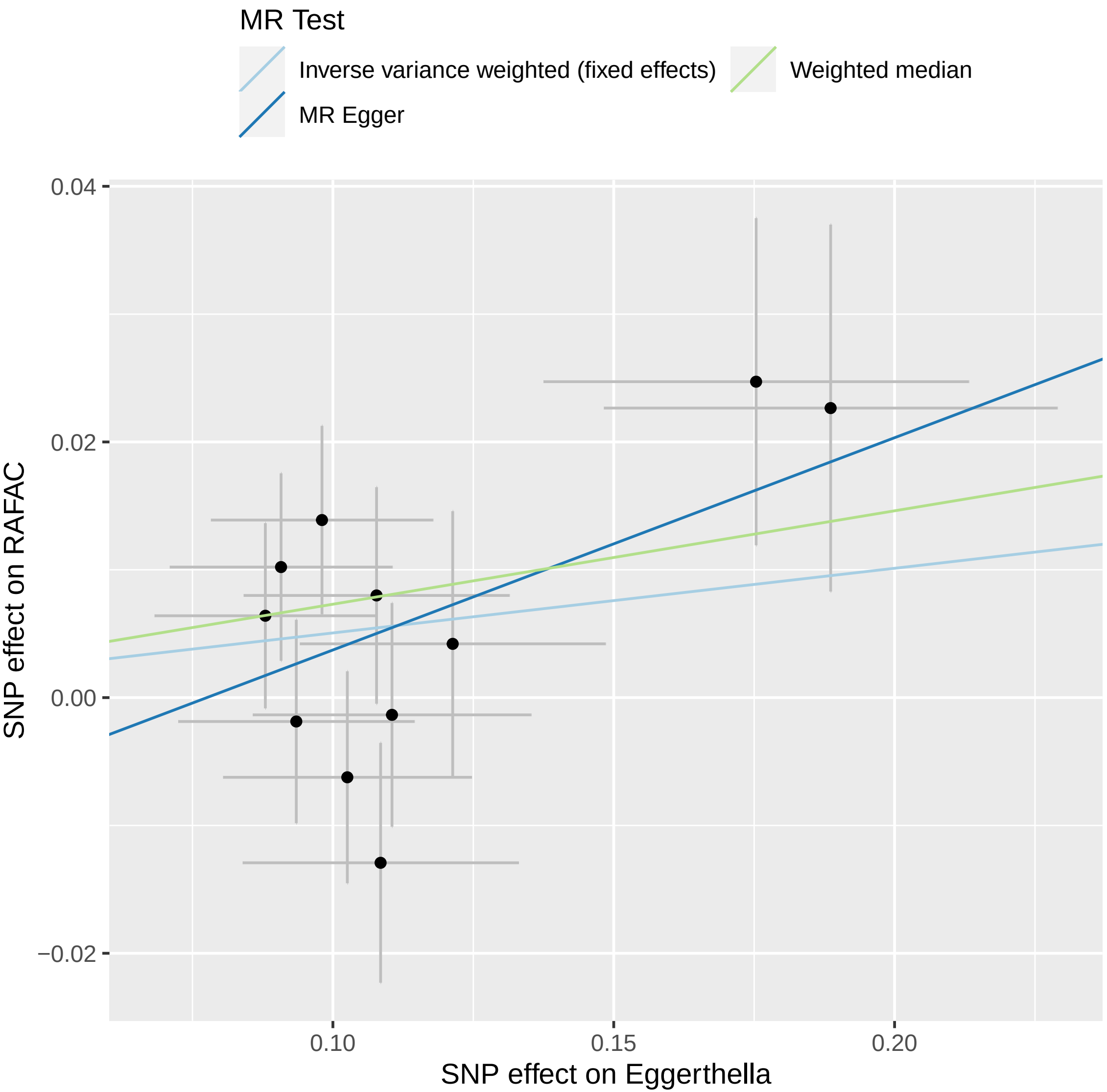
MR Test
Inverse variance weighted (fixed effects)
Weighted median
MR Egger
0.04
0.02
0.00
−0.02
0.10
0.15
0.20
SNP effect on RAFAC
SNP effect on Eggerthella

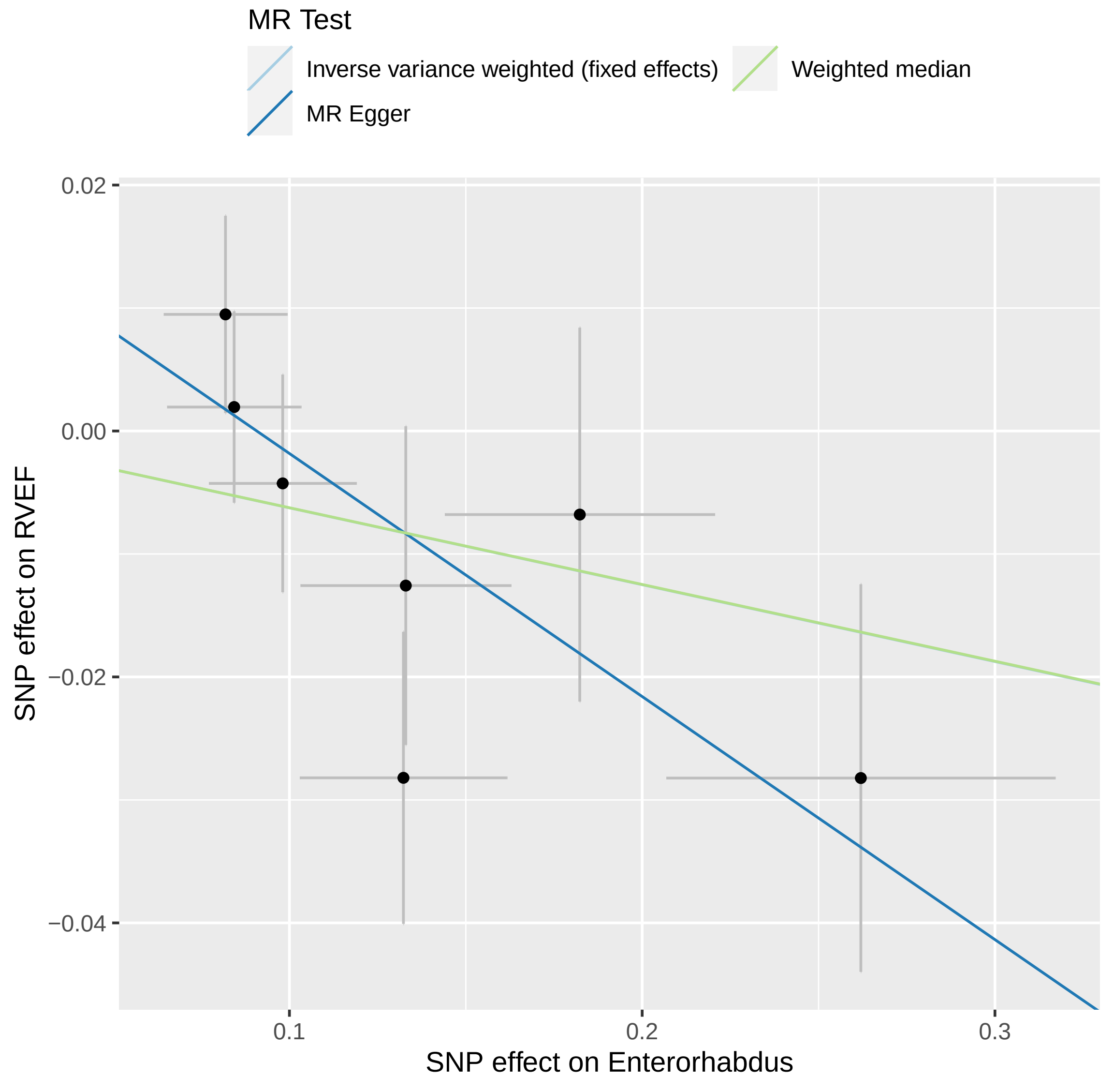
MR Test
Inverse variance weighted (fixed effects)
Weighted median
MR Egger
0.02
0.00
−0.02
−0.04
SNP effect on RVEF
0.1
0.2
0.3
SNP effect on Enterorhabdus

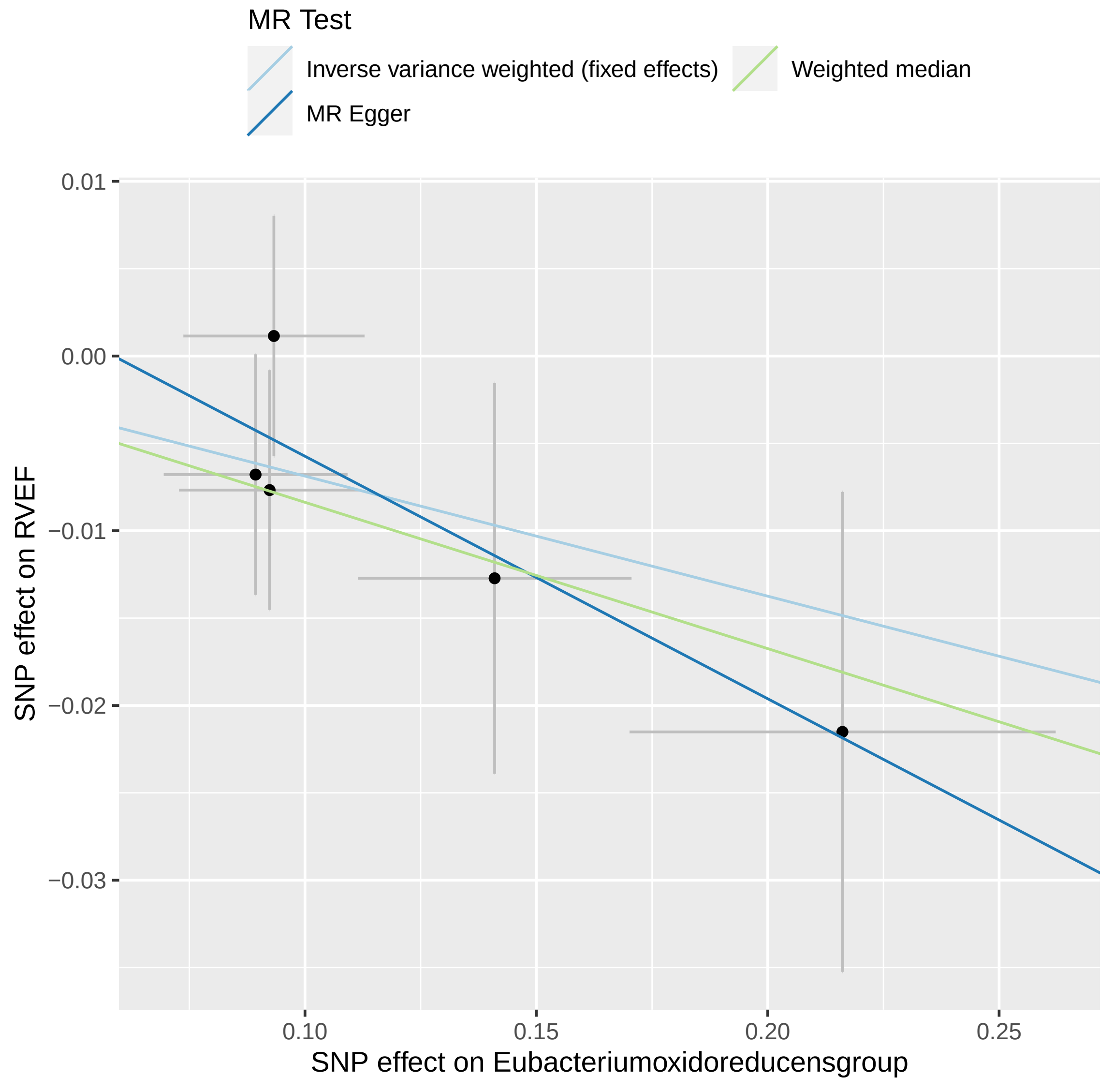
MR Test
Inverse variance weighted (fixed effects)
Weighted median
MR Egger
0.01
0.00
−0.01
−0.02
−0.03
SNP effect on RVEF
0.10
0.15
0.20
0.25
SNP effect on Eubacteriumoxidoreducensgroup

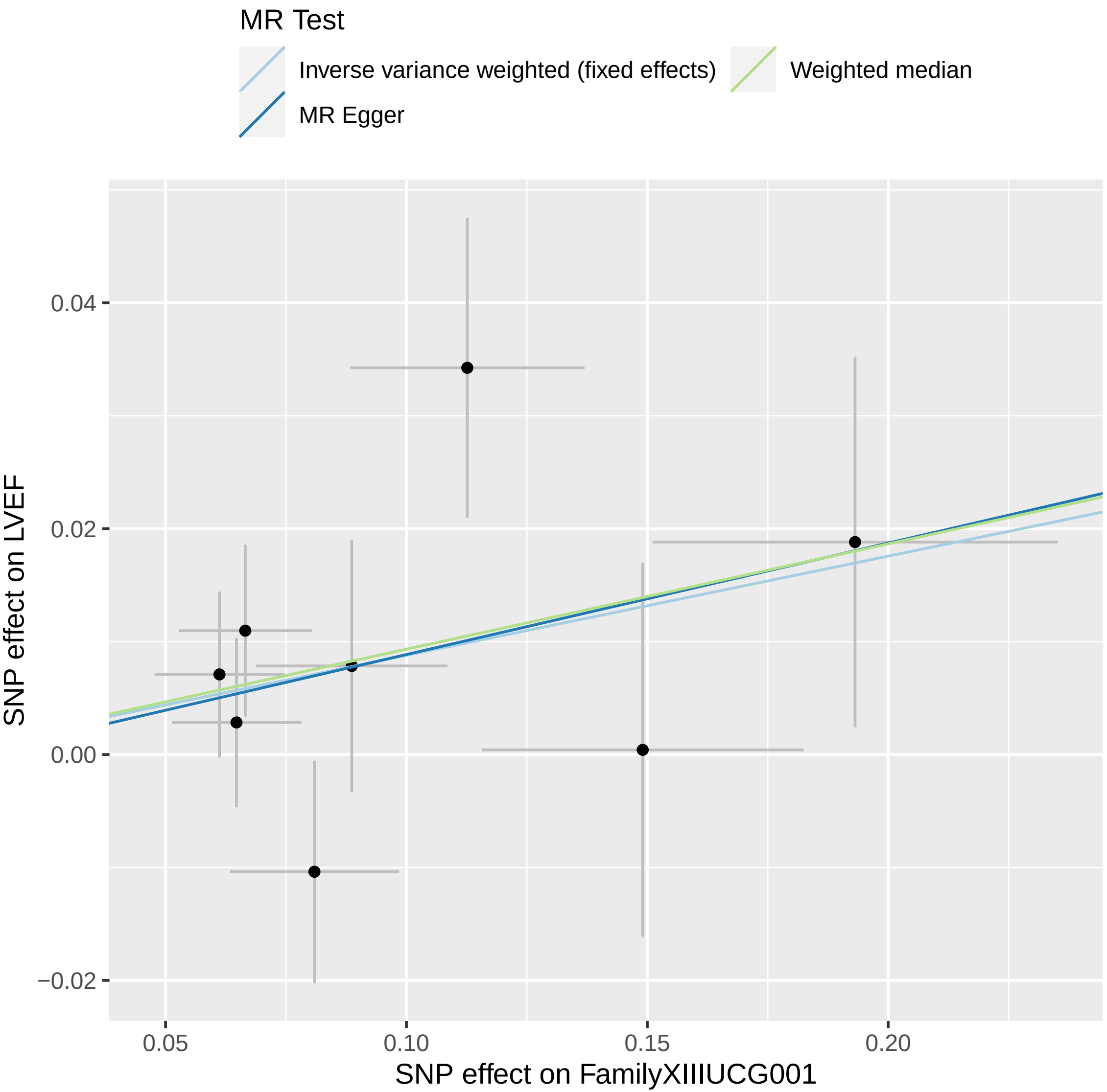
MR Test
Inverse variance weighted (fixed effects)
MR Egger
Weighted median
0.04
0.02
0.00
−0.02
0.05
0.10
0.15
0.20
SNP effect on LVEF
SNP effect on FamilyXIIIUCG001

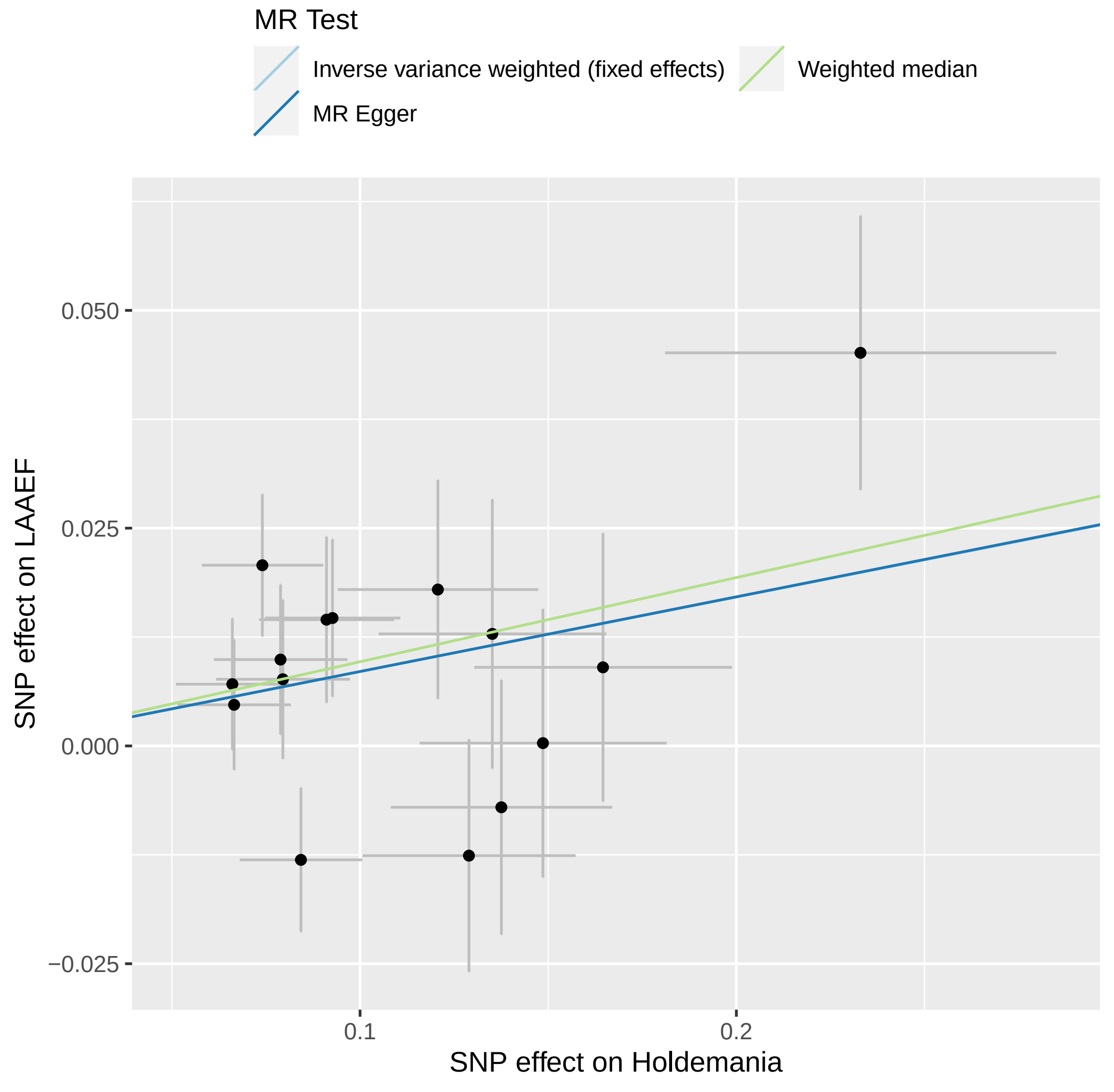
MR Test
Inverse variance weighted (fixed effects)
Weighted median
MR Egger
0.050
0.025
0.000
−0.025
0.1
0.2
SNP effect on LAAEF
SNP effect on Holdemania

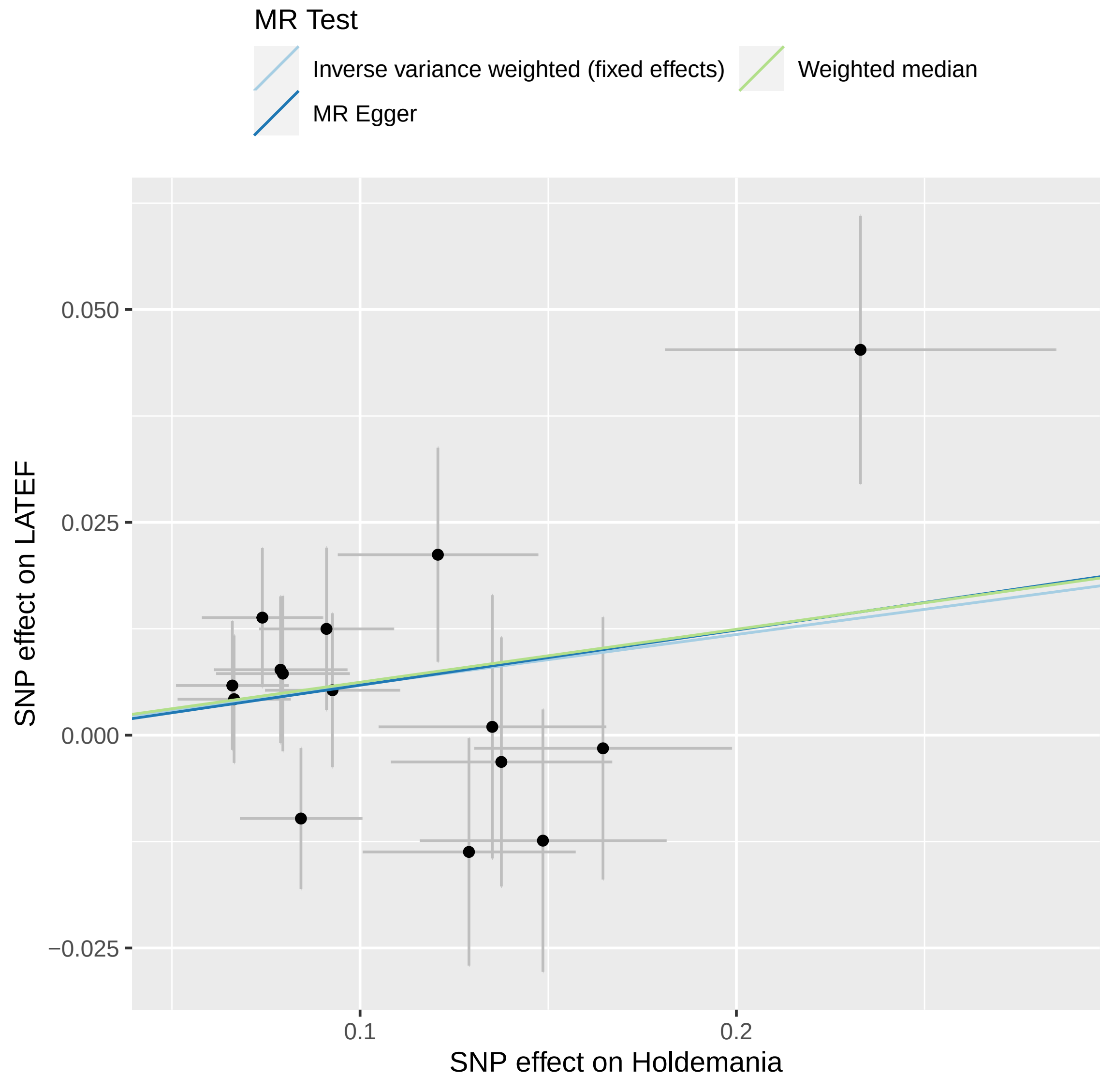
MR Test
Inverse variance weighted (fixed effects)
Weighted median
MR Egger
0.050
0.025
0.000
−0.025
0.1
0.2
SNP effect on LATEF
SNP effect on Holdemania

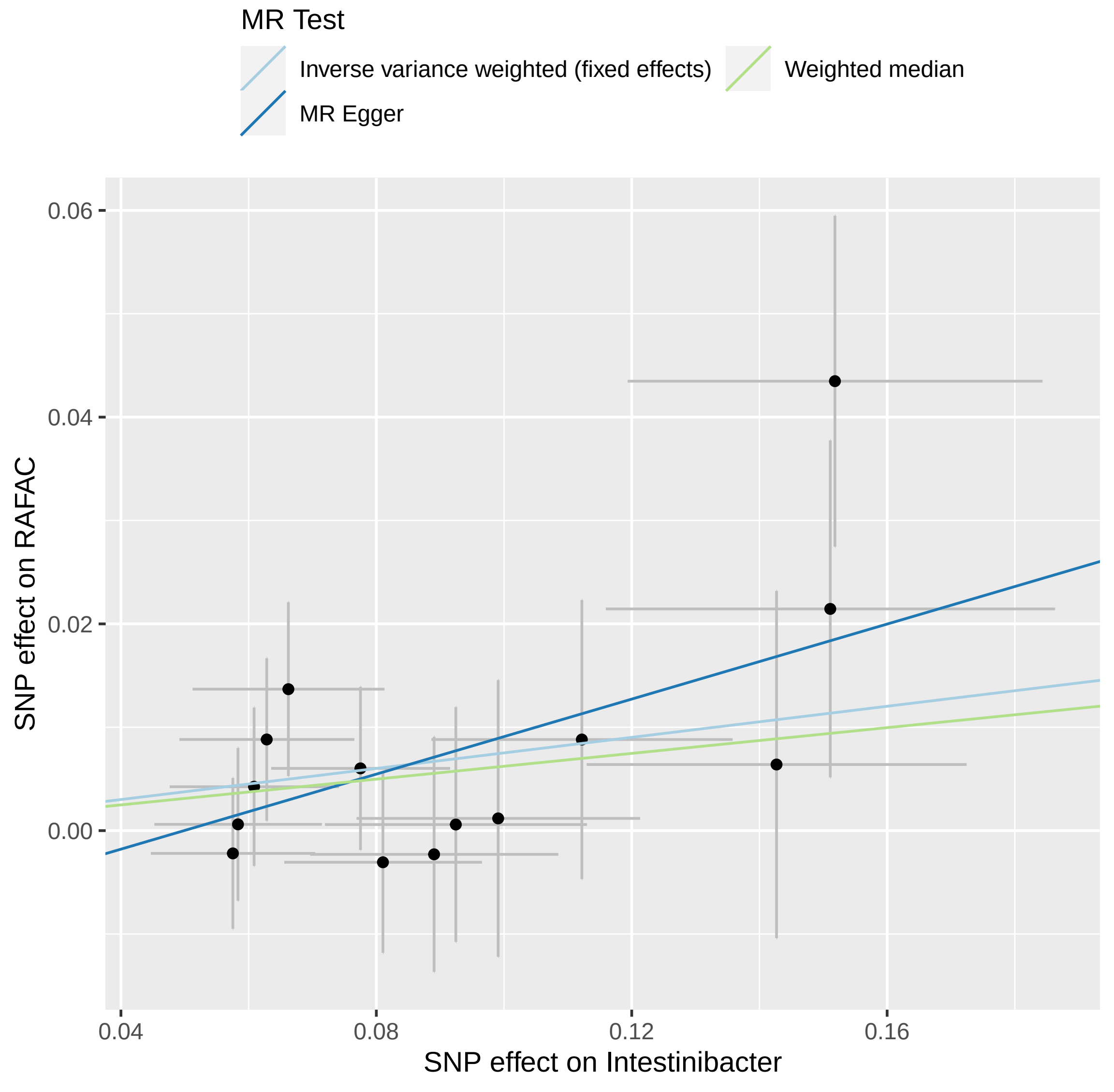

MR Test
Inverse variance weighted (fixed effects)
Weighted median
MR Egger
0.06
0.04
0.02
0.00
SNP effect on RAFAC
0.04
0.08
0.12
0.16
SNP effect on Intestinibacter

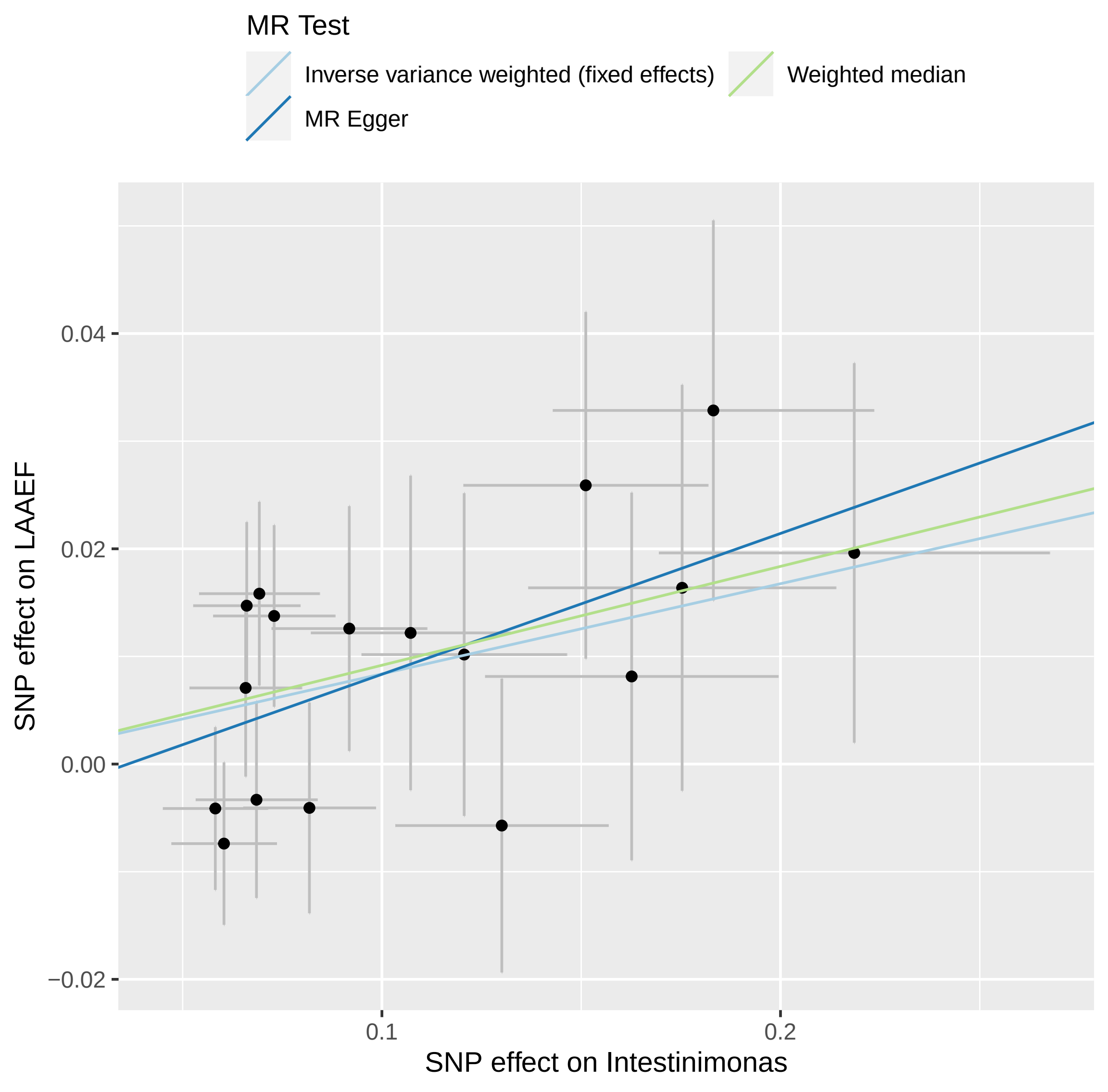
MR Test
Inverse variance weighted (fixed effects)
Weighted median
MR Egger
SNP effect on LAAEF
0.04
0.02
0.00
−0.02
0.1
0.2
SNP effect on Intestinimonas

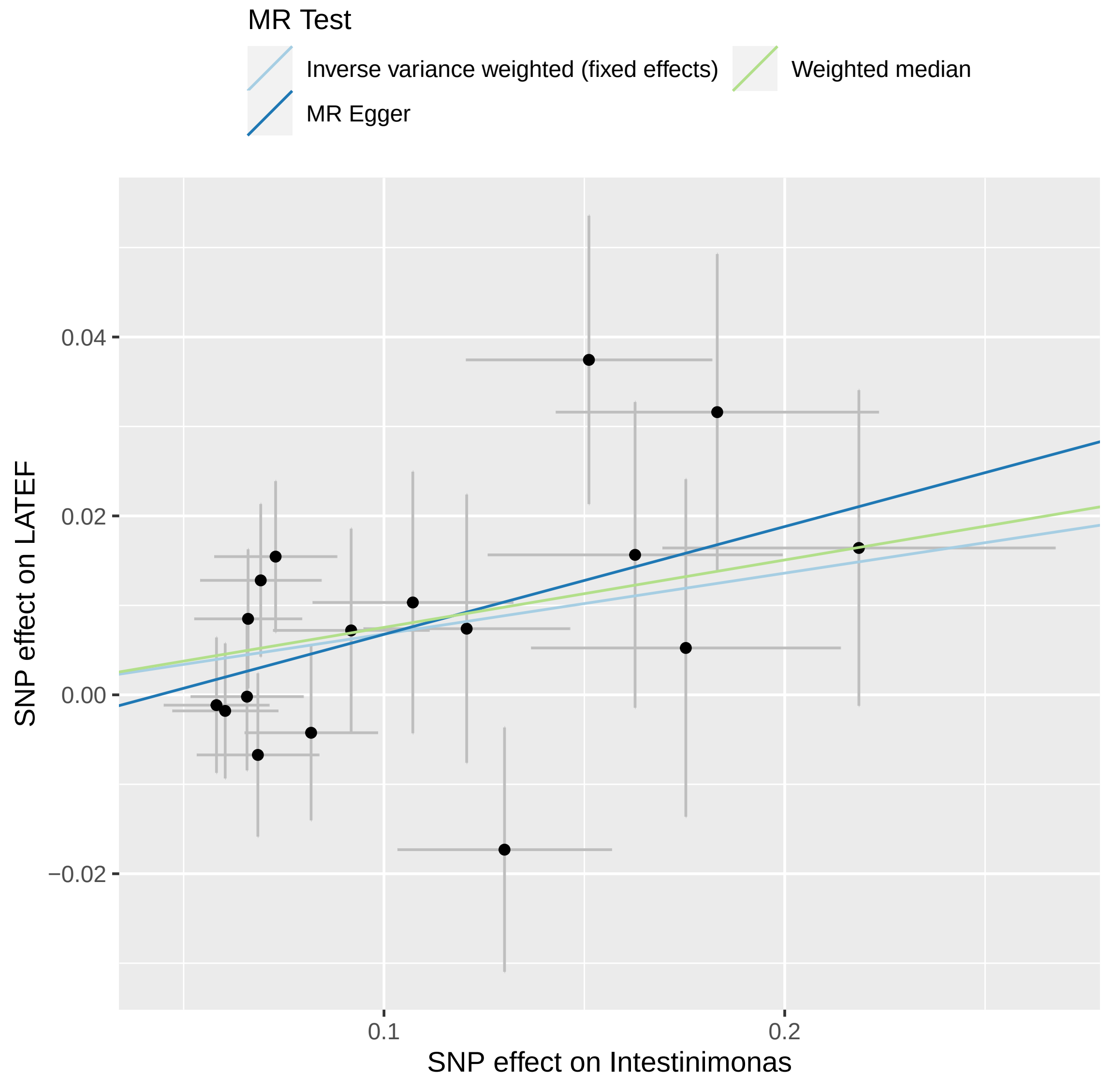
MR Test
Inverse variance weighted (fixed effects)
Weighted median
MR Egger
0.04
0.02
0.00
−0.02
SNP effect on LATEF
0.1
0.2
SNP effect on Intestinimonas

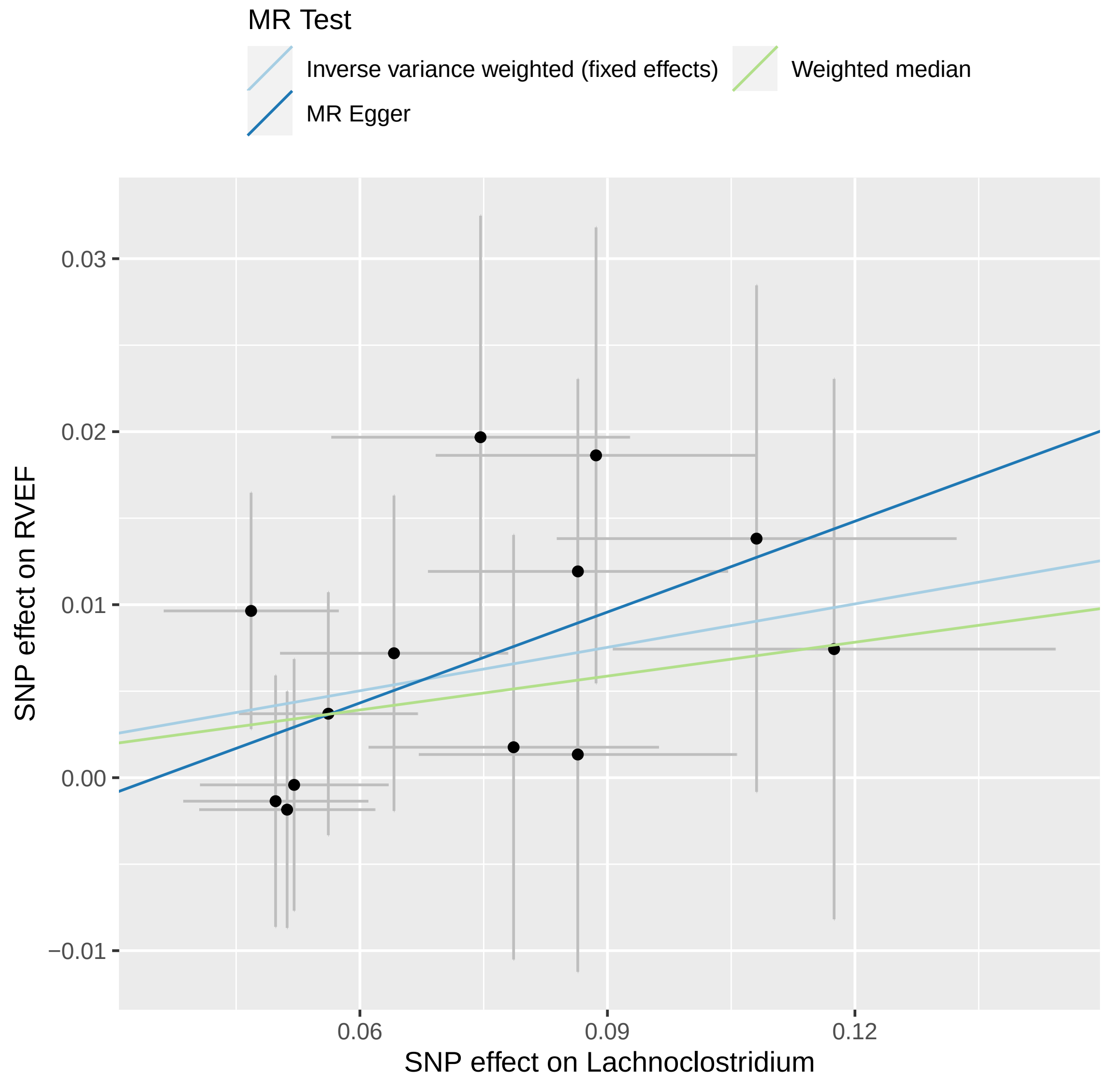
MR Test
Inverse variance weighted (fixed effects)
Weighted median
MR Egger
SNP effect on RVEF
0.03
0.02
0.01
0.00
−0.01
0.06
0.09
0.12
SNP effect on Lachnoclostridium

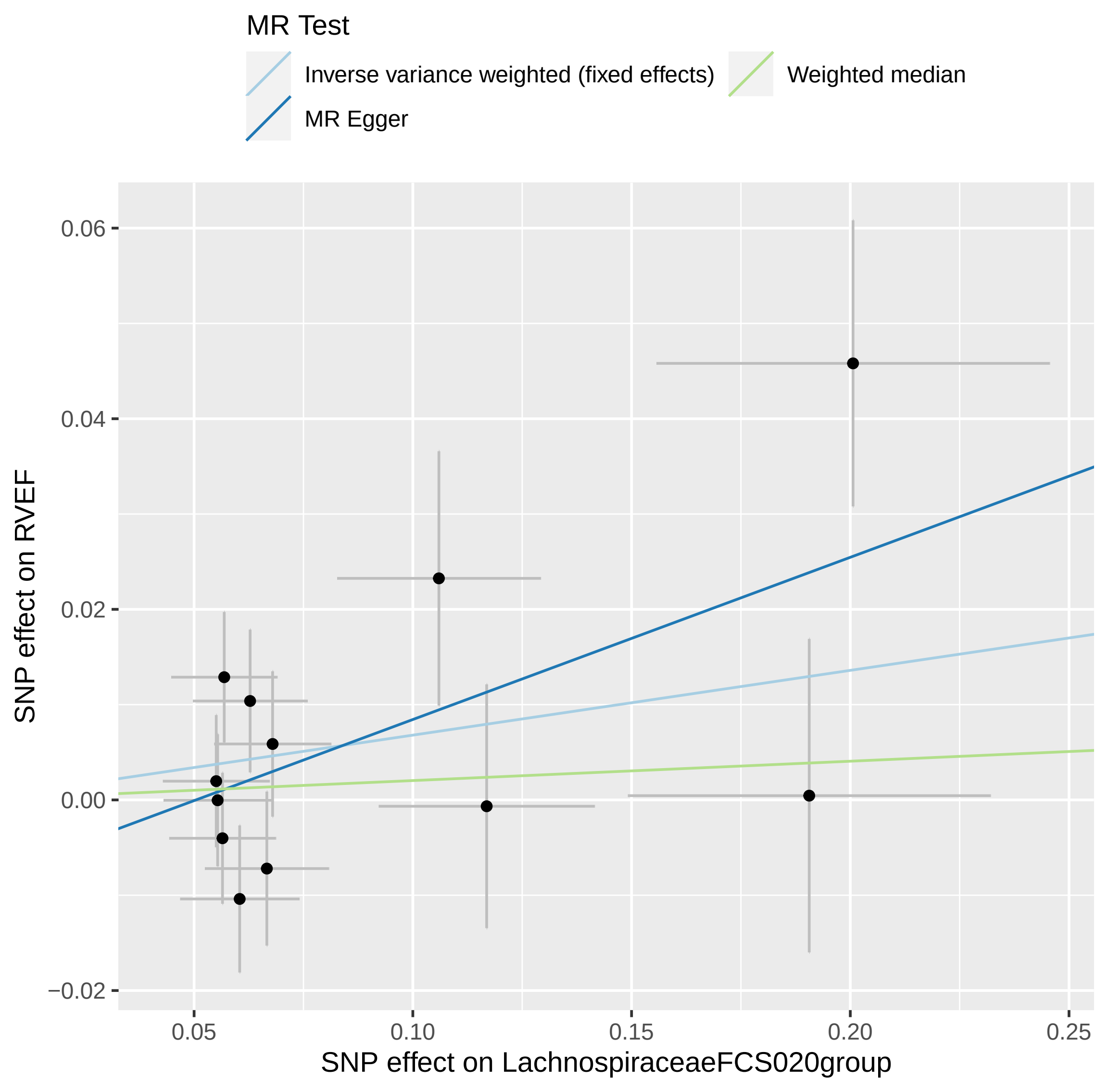
MR Test
Inverse variance weighted (fixed effects)
Weighted median
MR Egger
0.06
0.04
0.02
0.00
−0.02
SNP effect on RVEF
0.05
0.10
0.15
0.20
0.25
SNP effect on LachnospiraceaeFCS020group

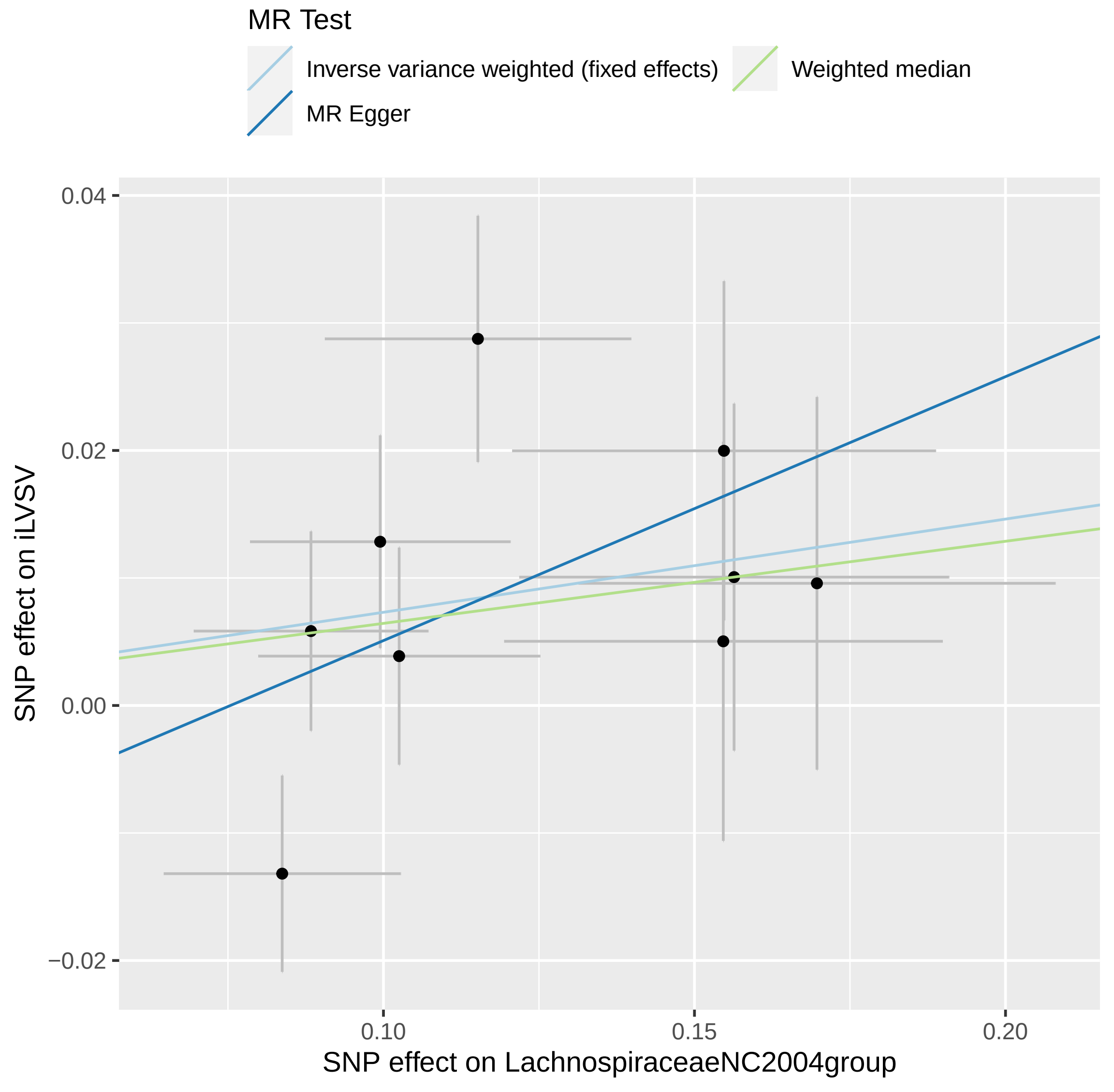

MR Test
Inverse variance weighted (fixed effects)
MR Egger
Weighted median
SNP effect on iLVSV
0.04
0.02
0.00
−0.02
0.10
0.15
0.20
SNP effect on LachnospiraceaeNC2004group

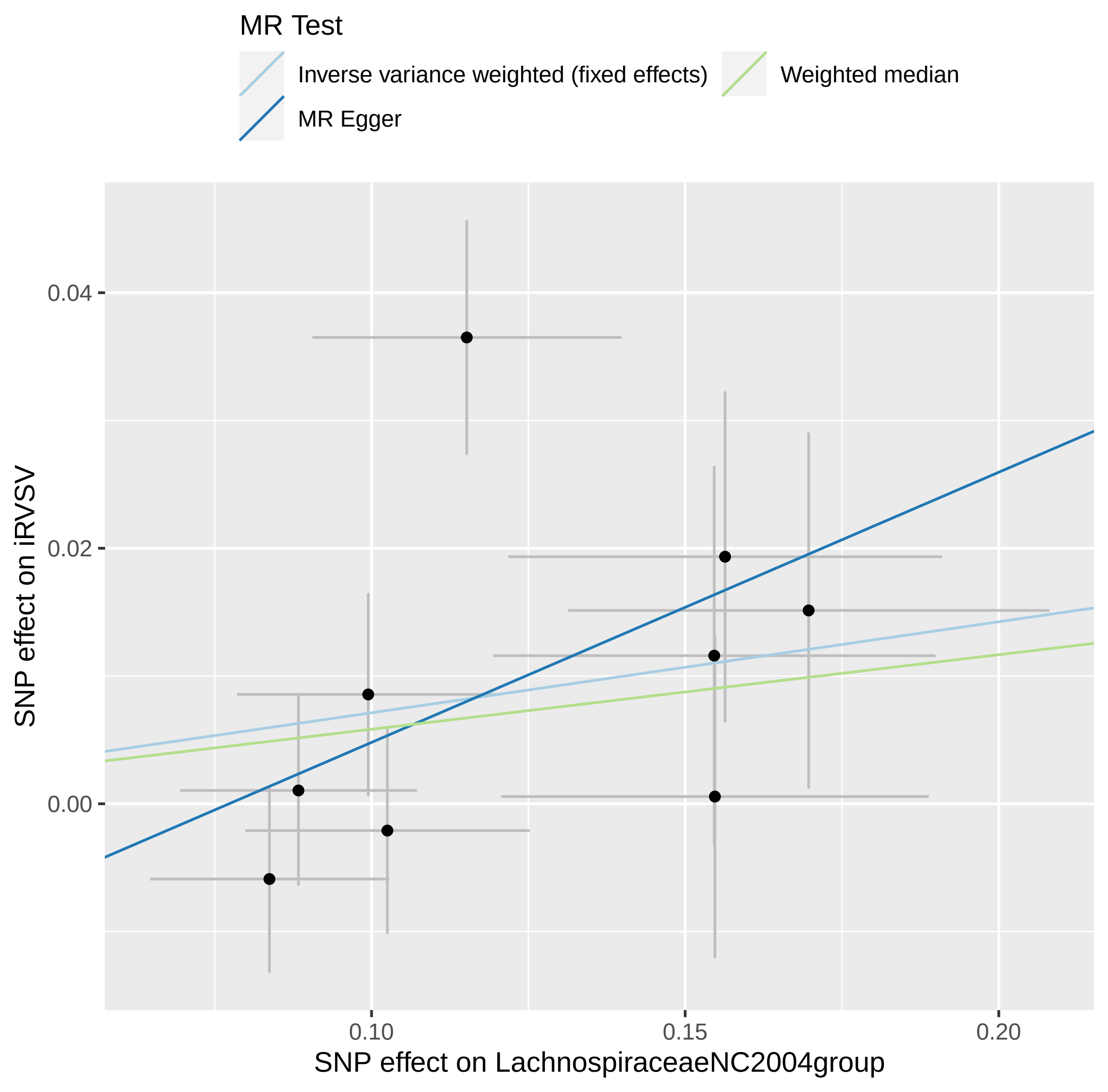

MR Test
Inverse variance weighted (fixed effects)
Weighted median
MR Egger
0.04
0.02
0.00
0.10
0.15
0.20
SNP effect on iRVSV
SNP effect on LachnospiraceaeNC2004group

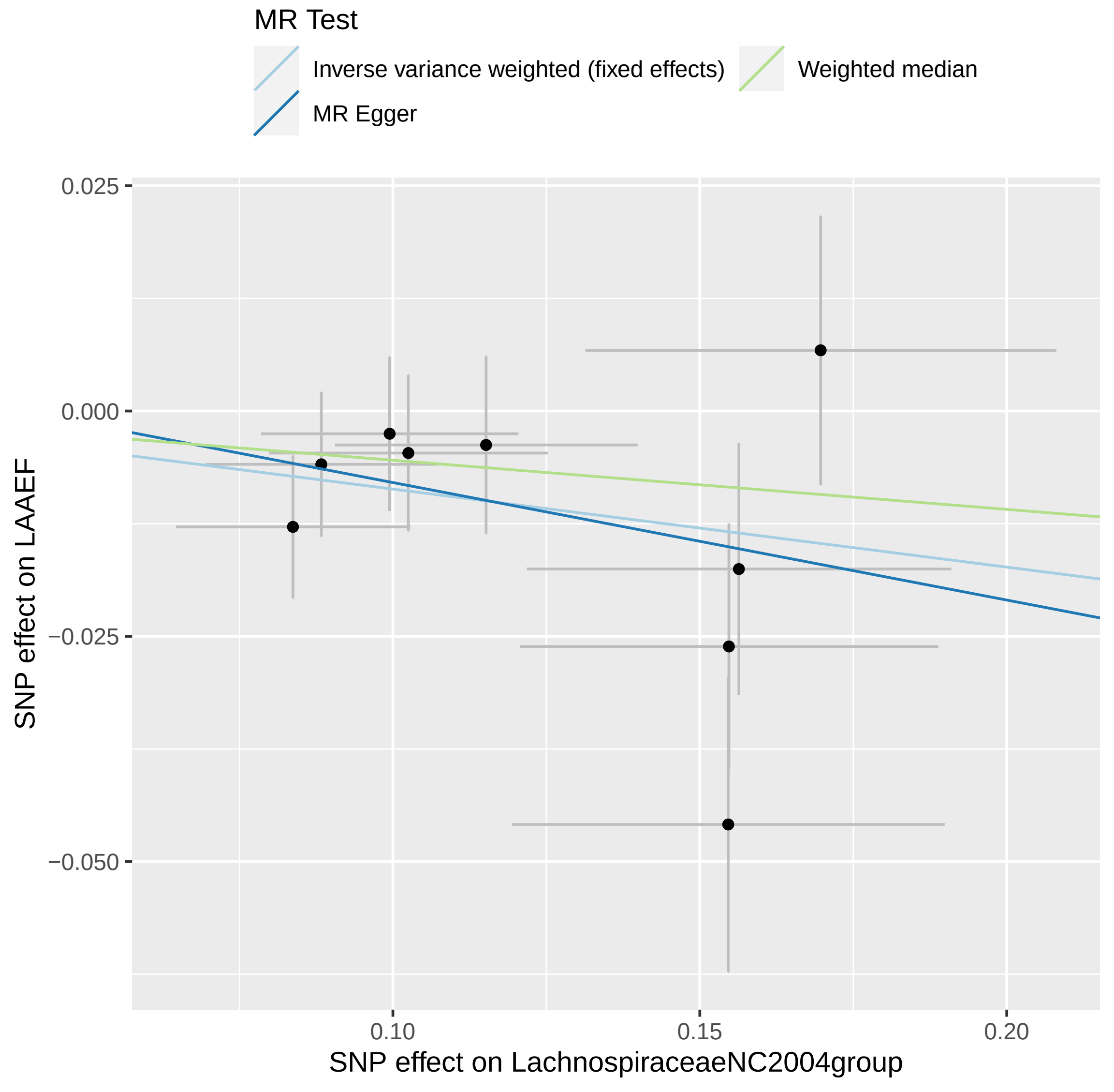
MR Test
Inverse variance weighted (fixed effects)
Weighted median
MR Egger
0.025
0.000
−0.025
−0.050
SNP effect on LAAEF
0.10
0.15
0.20
SNP effect on LachnospiraceaeNC2004group

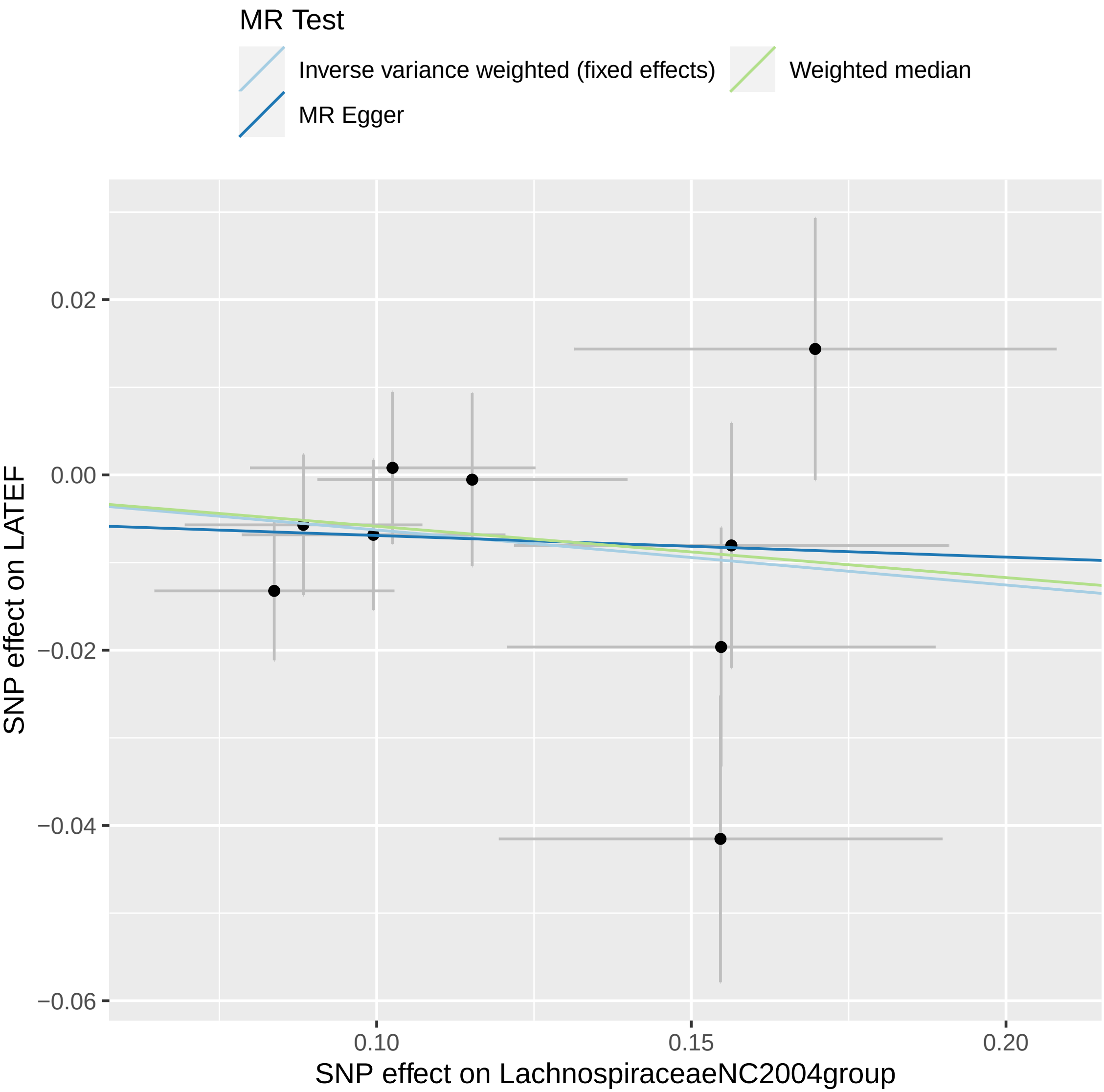
MR Test
Inverse variance weighted (fixed effects)
Weighted median
MR Egger
SNP effect on LATEF
0.02
0.00
−0.02
−0.04
−0.06
0.10
0.15
0.20
SNP effect on LachnospiraceaeNC2004group

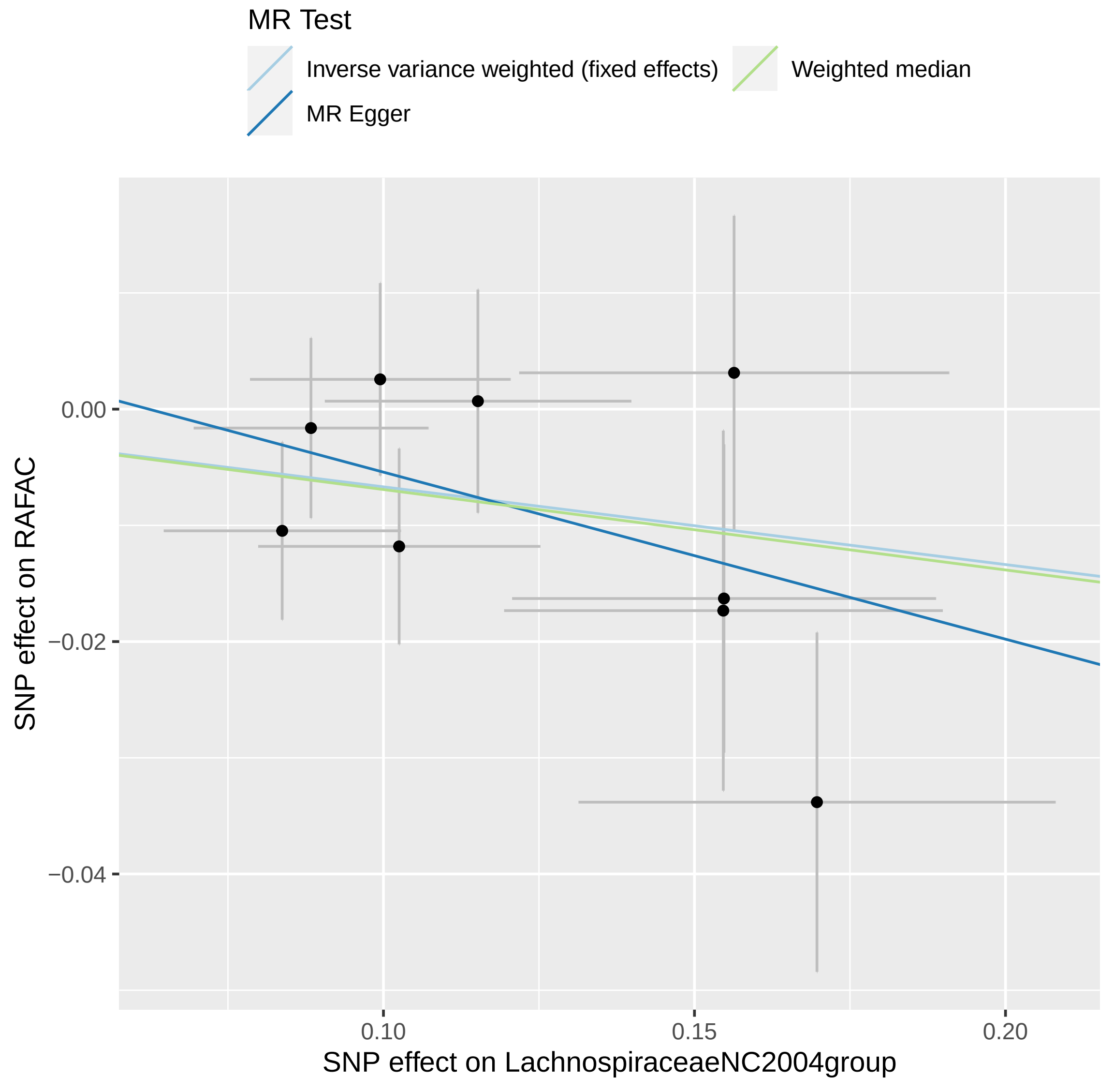

MR Test
Inverse variance weighted (fixed effects)
Weighted median
MR Egger
0.00
−0.02
−0.04
0.10
0.15
0.20
SNP effect on RAFAC
SNP effect on LachnospiraceaeNC2004group

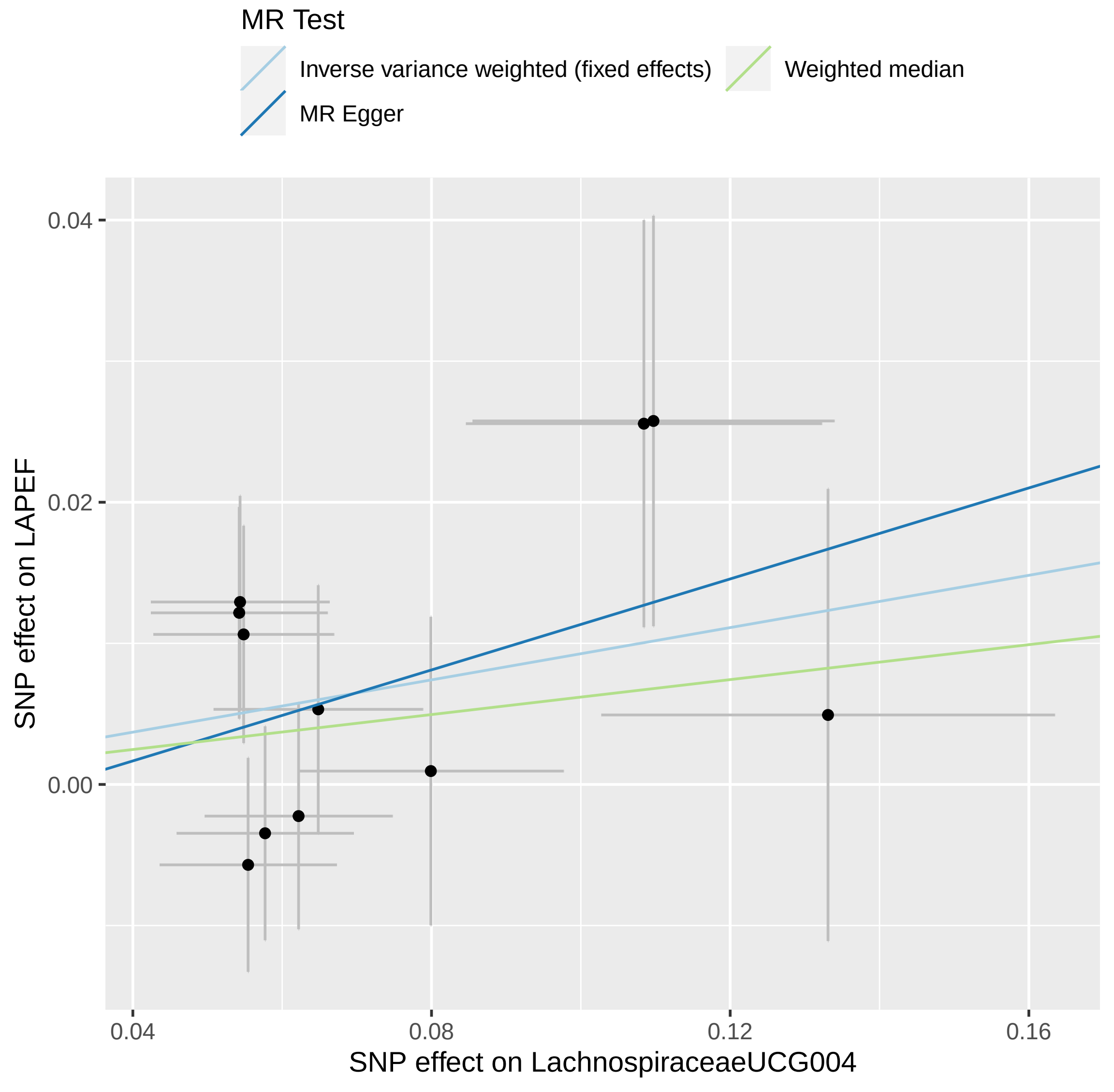
MR Test
Inverse variance weighted (fixed effects)
Weighted median
MR Egger
SNP effect on LAPEF
0.04
0.02
0.00
0.04
0.08
0.12
0.16
SNP effect on LachnospiraceaeUCG004

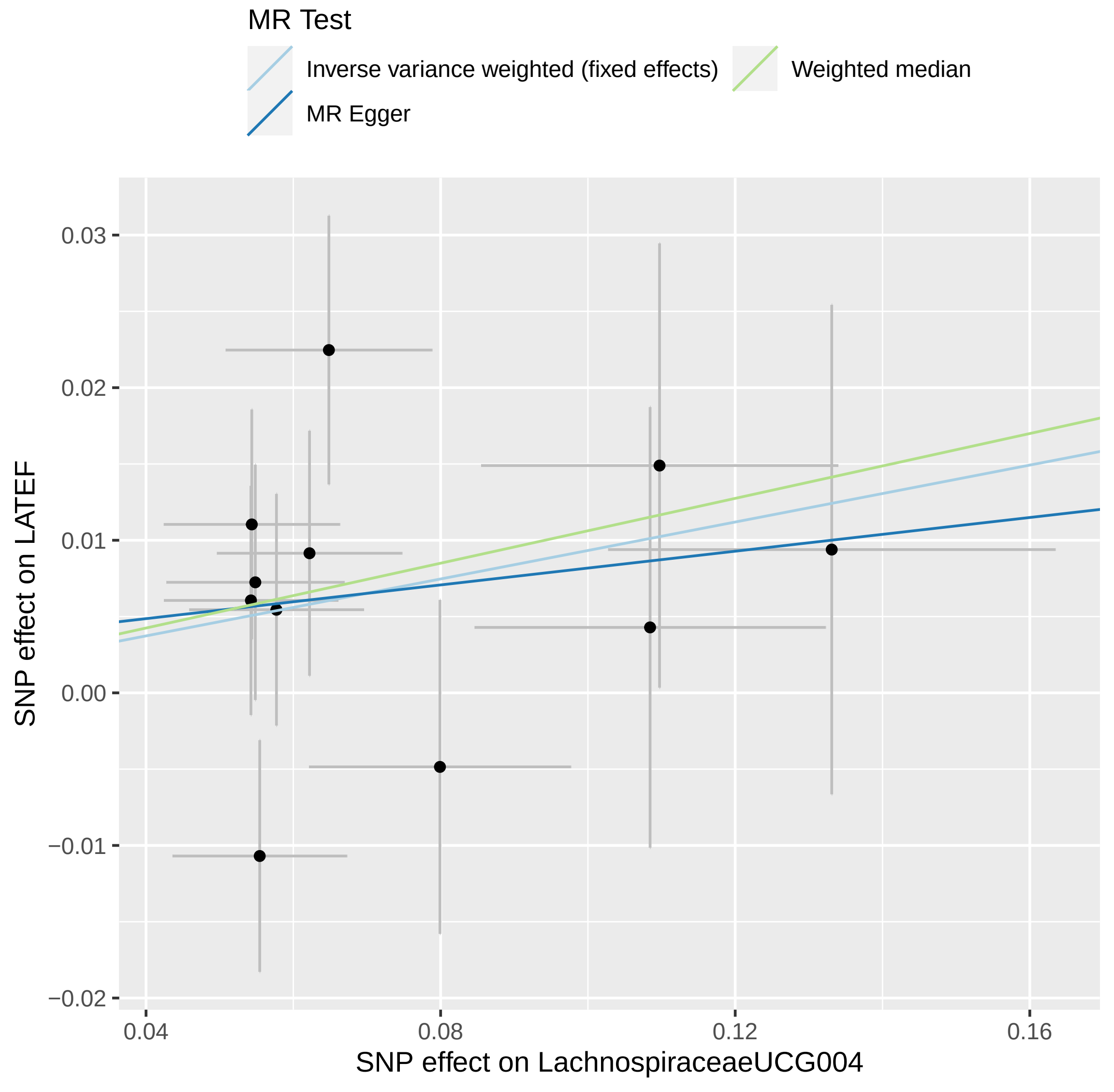
MR Test
Inverse variance weighted (fixed effects)
Weighted median
MR Egger
0.03
0.02
0.01
0.00
−0.01
−0.02
0.04
0.08
0.12
0.16
SNP effect on LATEF
SNP effect on LachnospiraceaeUCG004

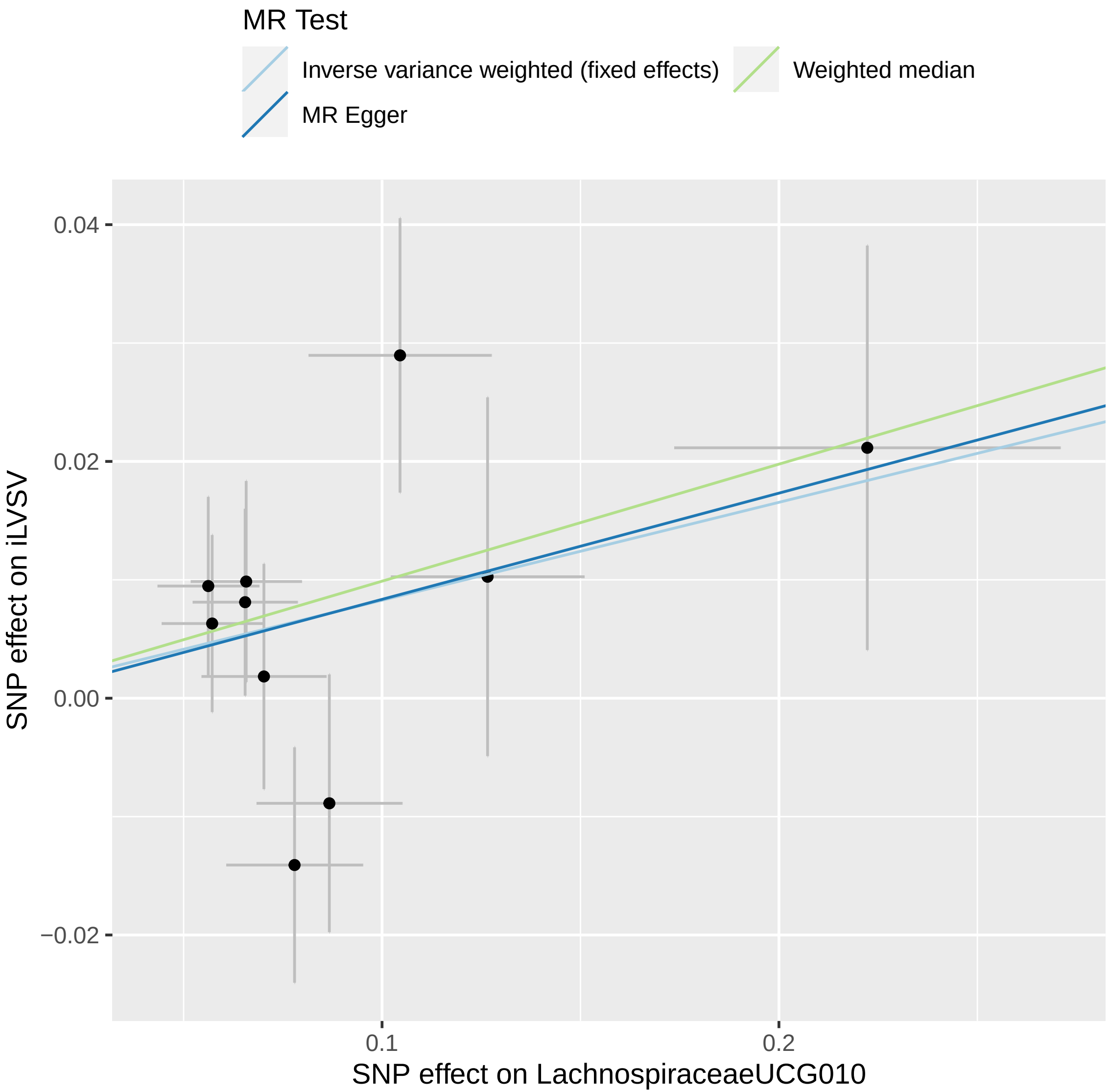
MR Test
Inverse variance weighted (fixed effects)
Weighted median
MR Egger
0.04
0.02
0.00
−0.02
0.1
0.2
SNP effect on iLVSV
SNP effect on LachnospiraceaeUCG010

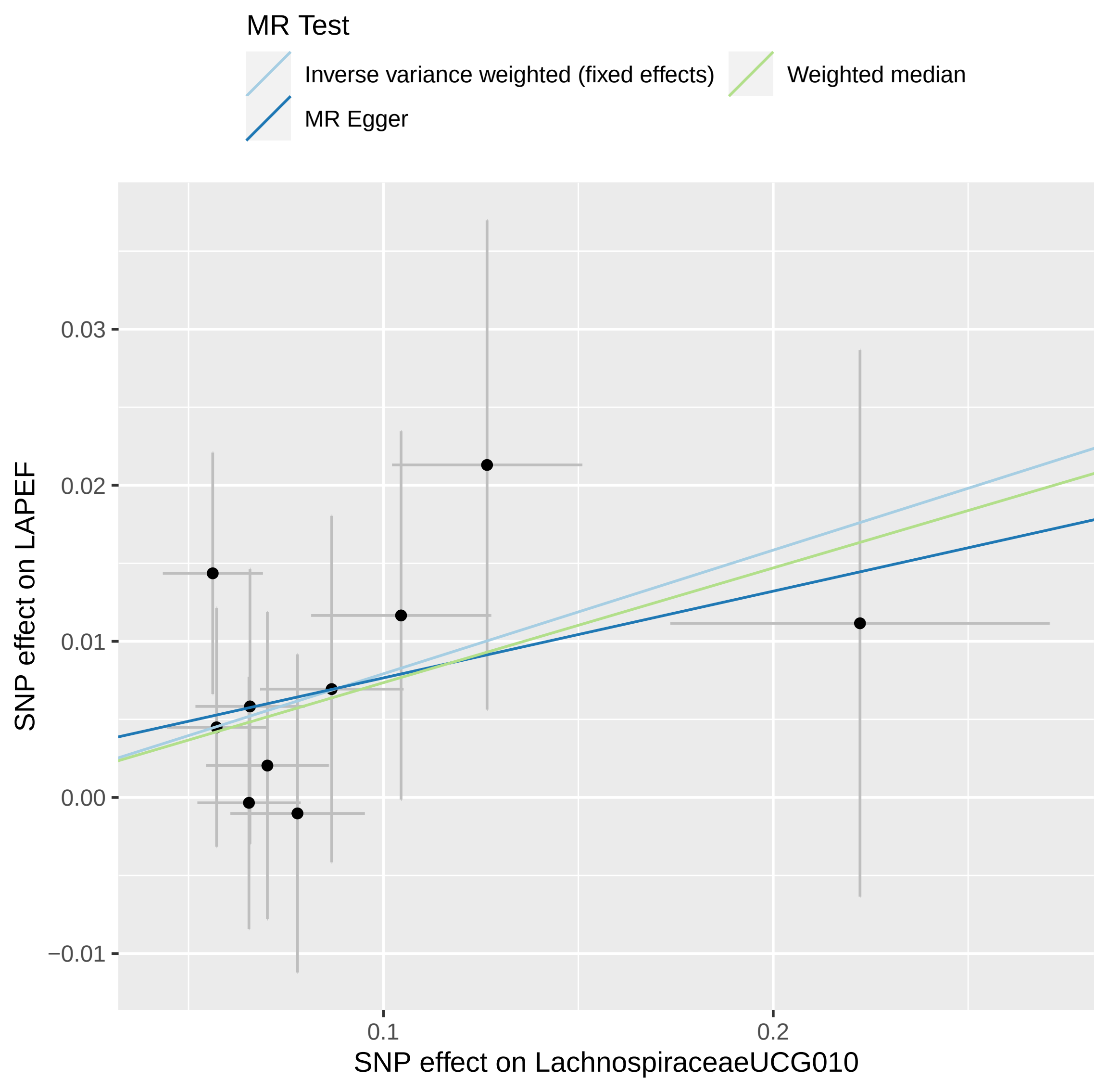
MR Test
Inverse variance weighted (fixed effects)
Weighted median
MR Egger
0.03
0.02
0.01
0.00
−0.01
0.1
0.2
SNP effect on LAPEF
SNP effect on LachnospiraceaeUCG010

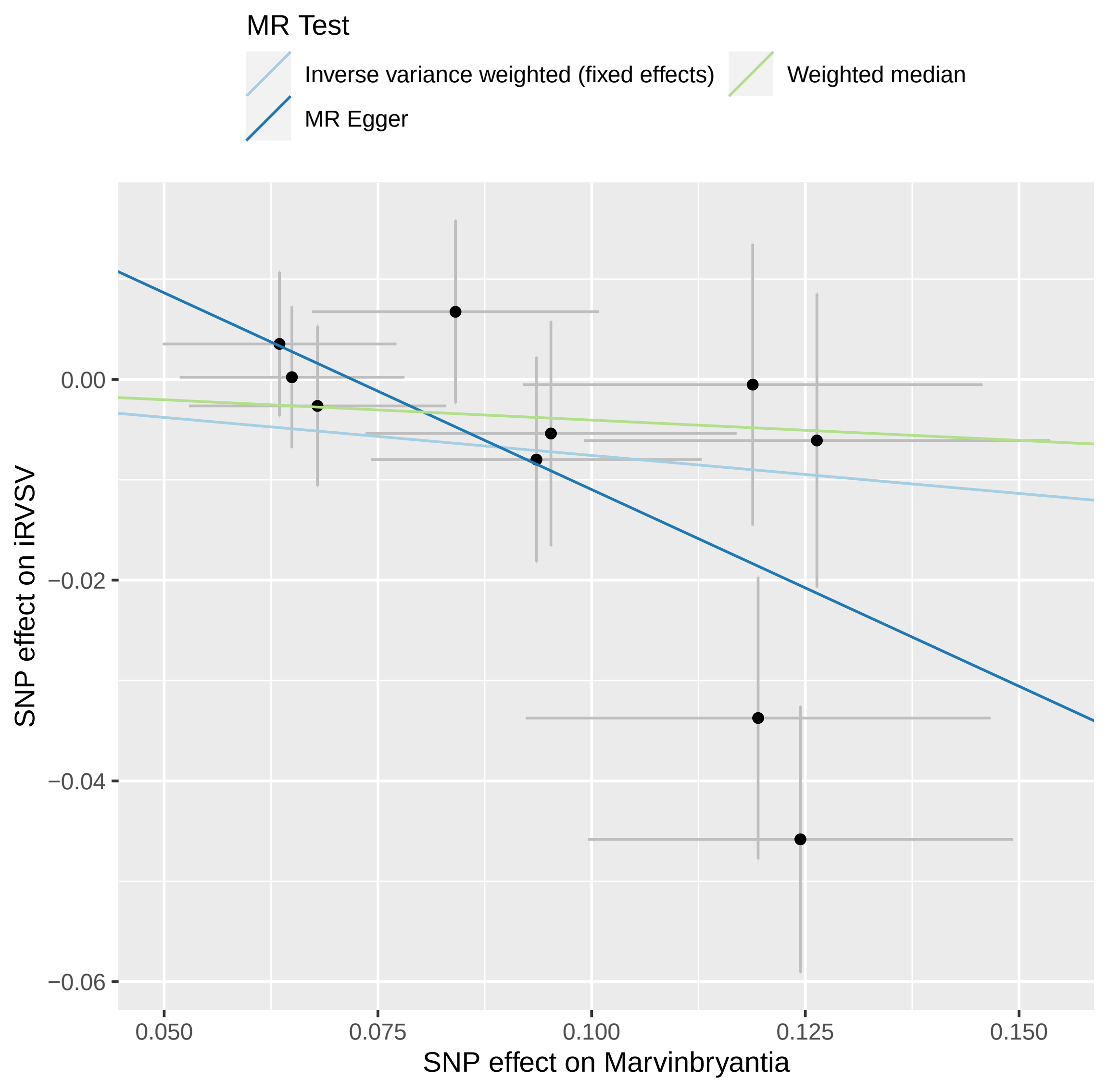
MR Test
Inverse variance weighted (fixed effects)
Weighted median
MR Egger
0.00
−0.02
−0.04
−0.06
0.050
0.075
0.100
0.125
0.150
SNP effect on iRVSV
SNP effect on Marvinbryantia

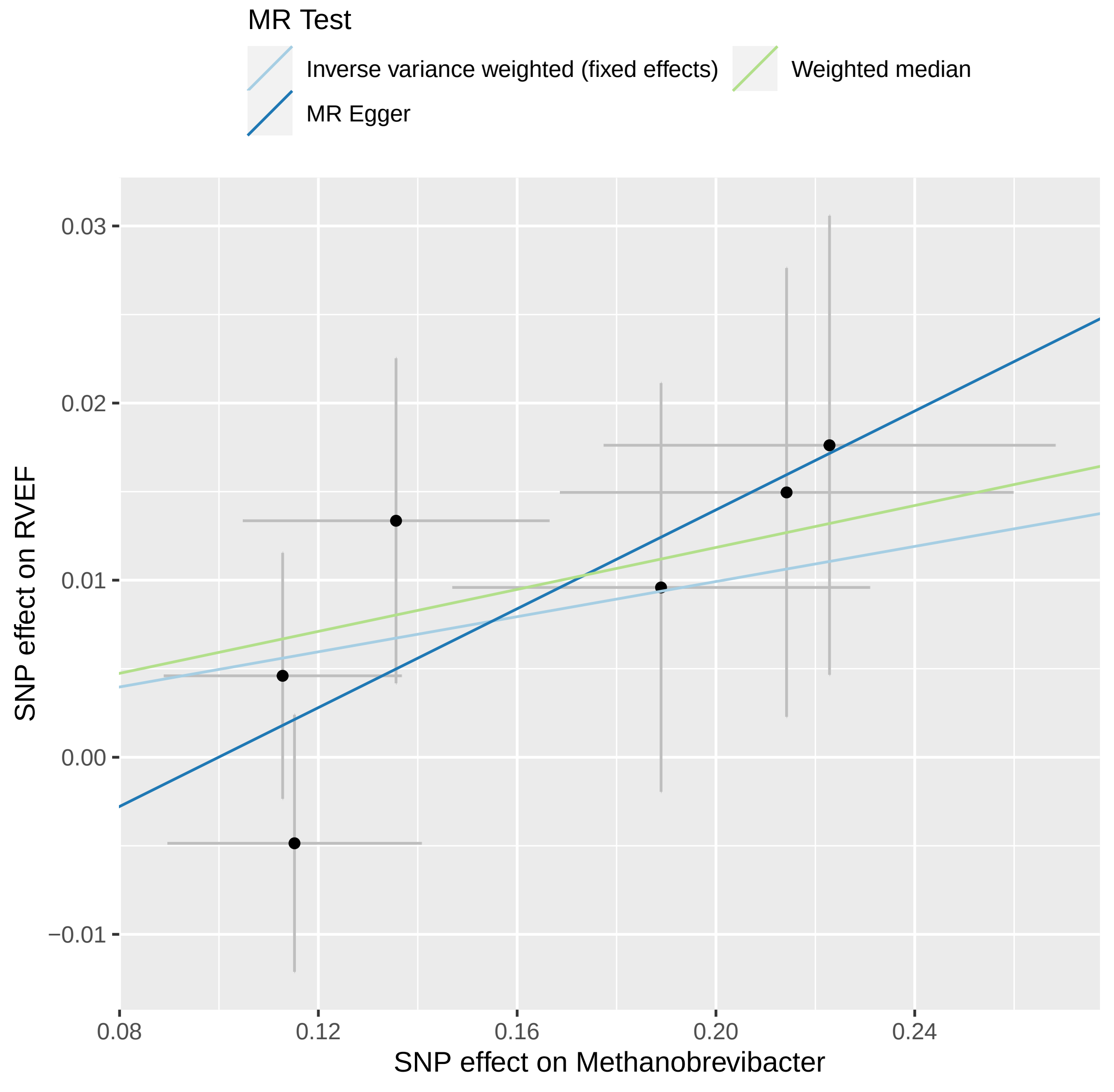

MR Test
Inverse variance weighted (fixed effects)
Weighted median
MR Egger
0.03
0.02
0.01
0.00
−0.01
SNP effect on RVEF
0.08
0.12
0.16
0.20
0.24
SNP effect on Methanobrevibacter

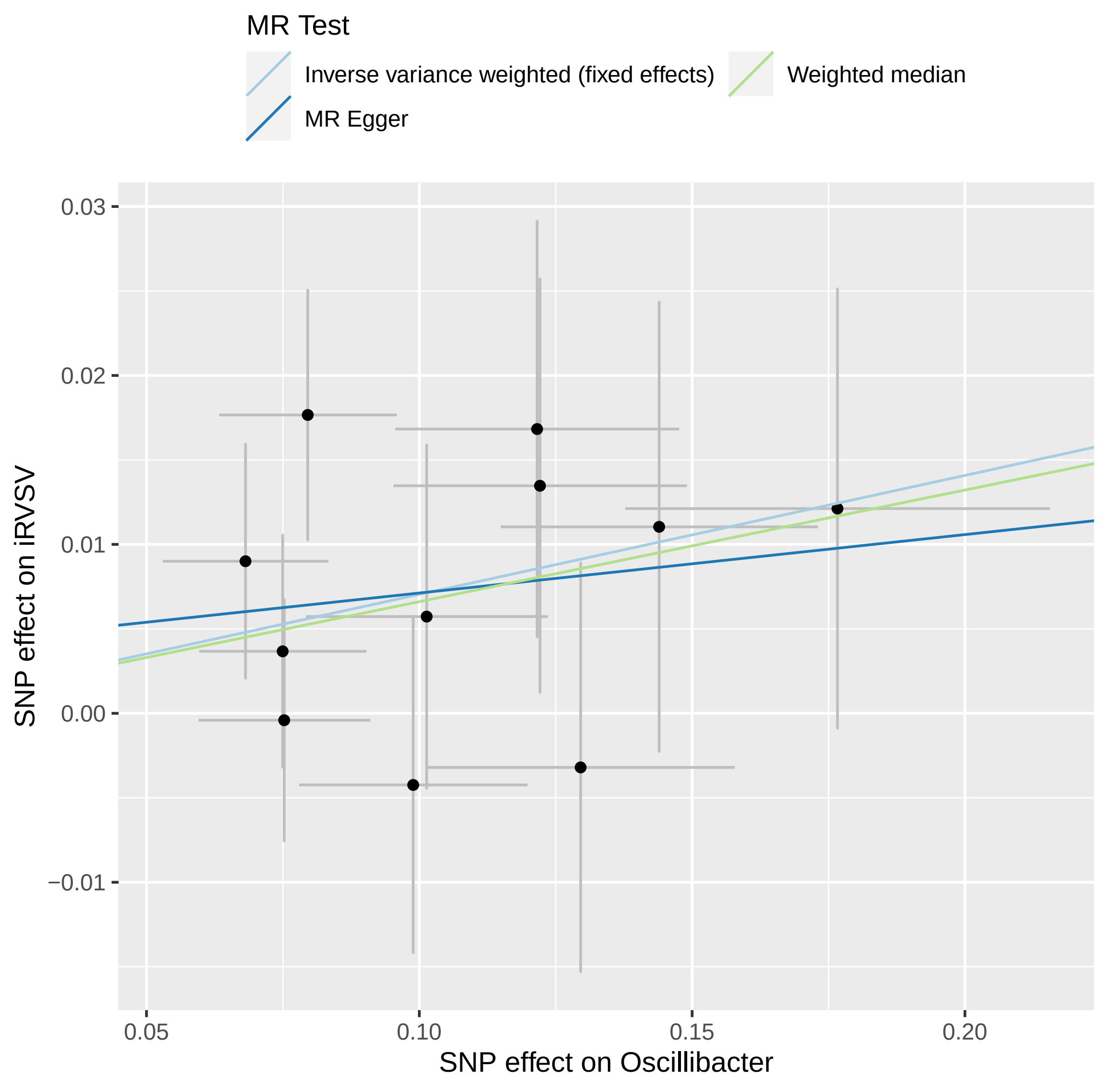
MR Test
Inverse variance weighted (fixed effects)
Weighted median
MR Egger
0.03
0.02
0.01
0.00
−0.01
SNP effect on iRVSV
0.05
0.10
0.15
0.20
SNP effect on Oscillibacter

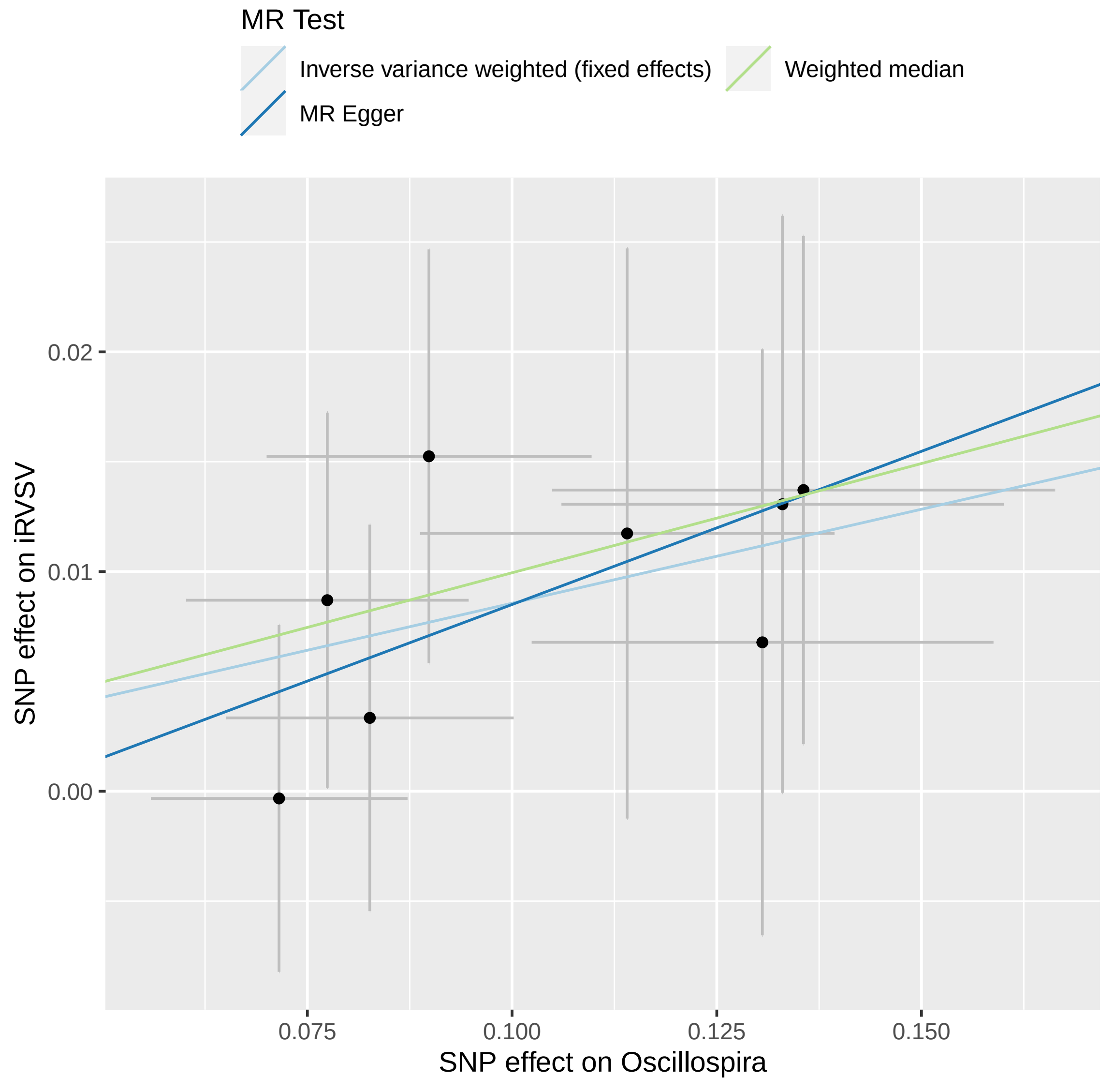
MR Test
Inverse variance weighted (fixed effects)
Weighted median
MR Egger
0.02
0.01
0.00
0.075
0.100
0.125
0.150
SNP effect on iRVSV
SNP effect on Oscillospira

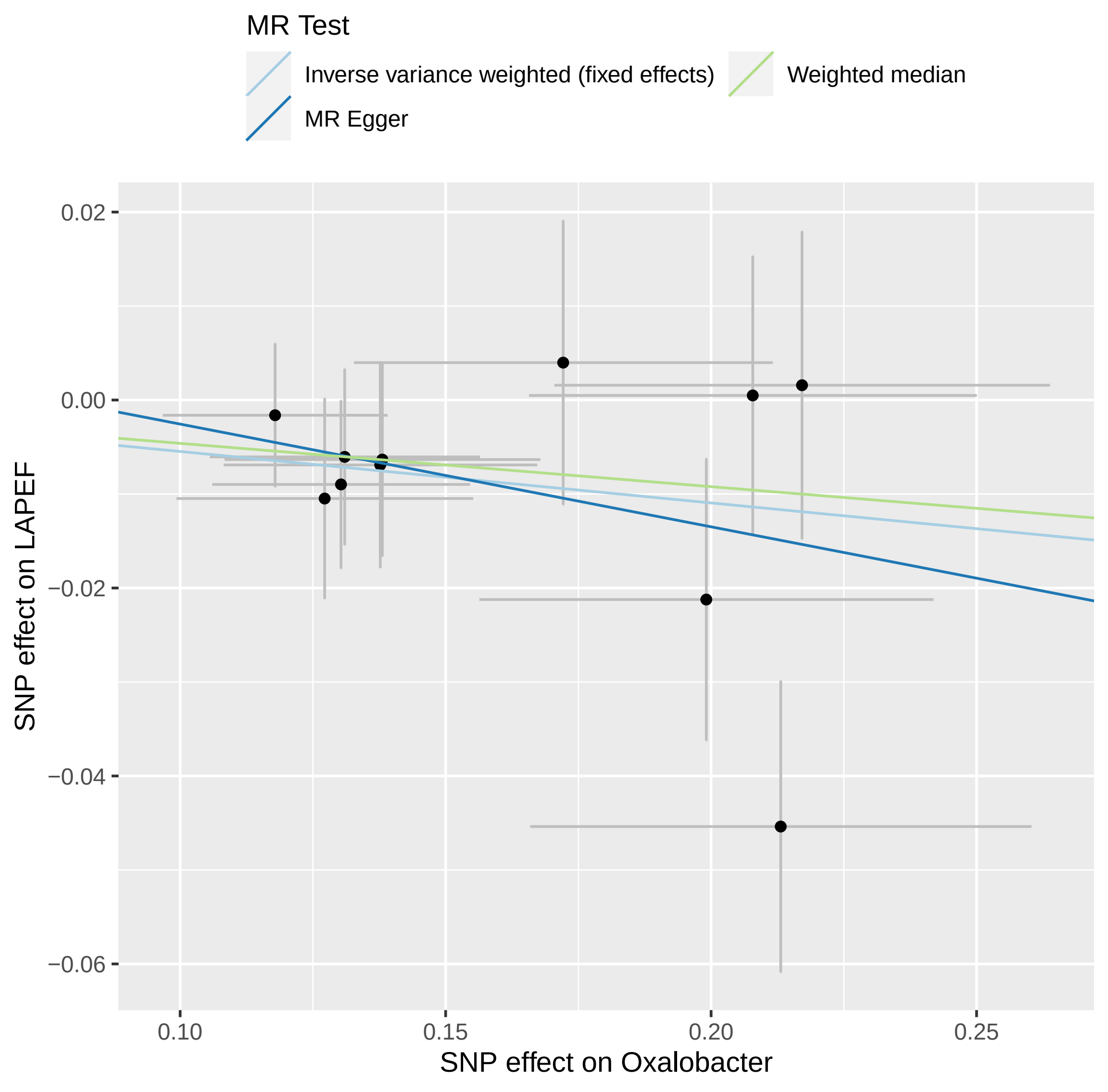
MR Test
Inverse variance weighted (fixed effects)
Weighted median
MR Egger
0.02
0.00
−0.02
−0.04
−0.06
SNP effect on LAPEF
0.10
0.15
0.20
0.25
SNP effect on Oxalobacter

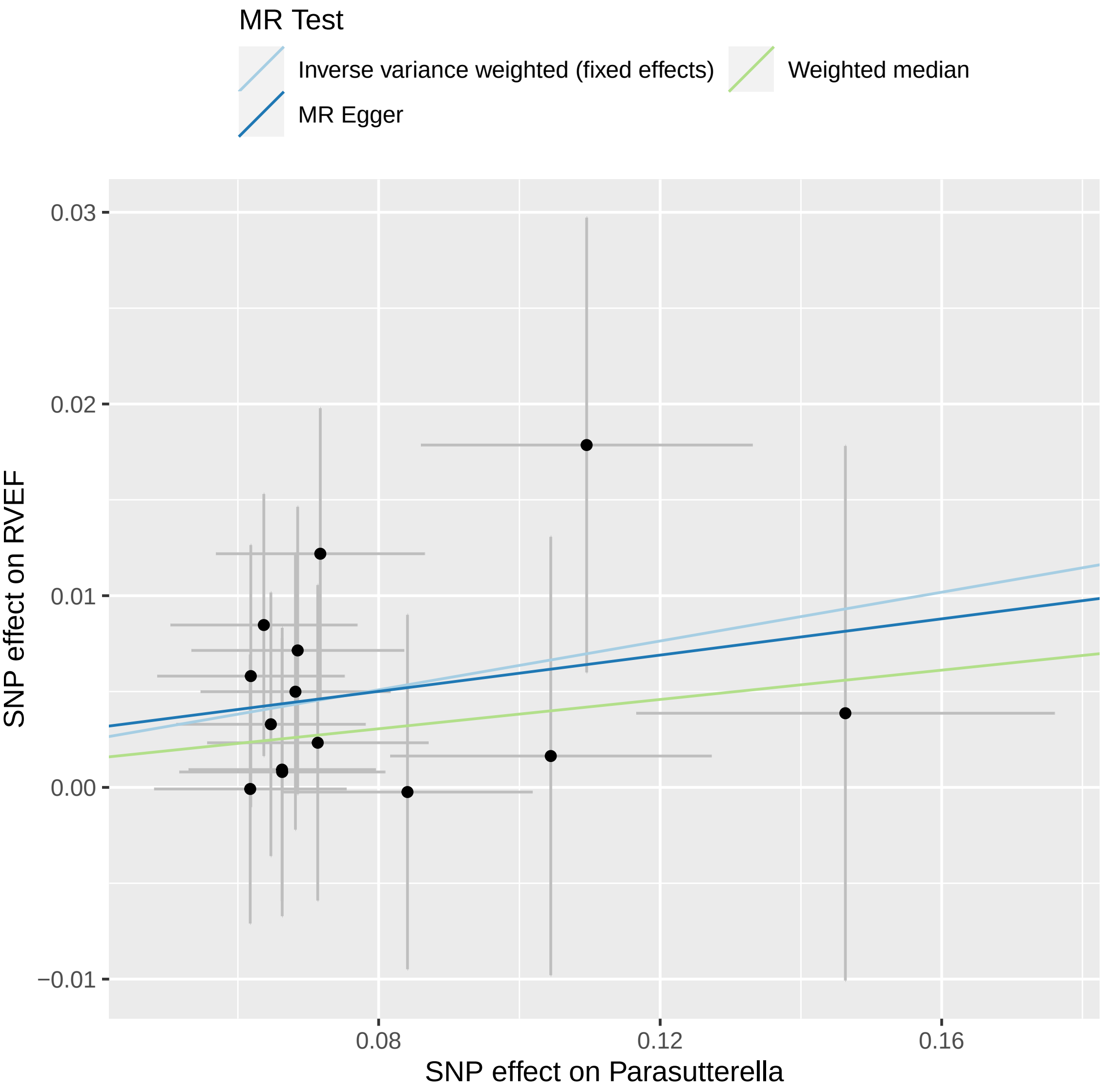

MR Test
Inverse variance weighted (fixed effects)
Weighted median
MR Egger
0.03
0.02
0.01
0.00
−0.01
0.08
0.12
0.16
SNP effect on RVEF
SNP effect on Parasutterella

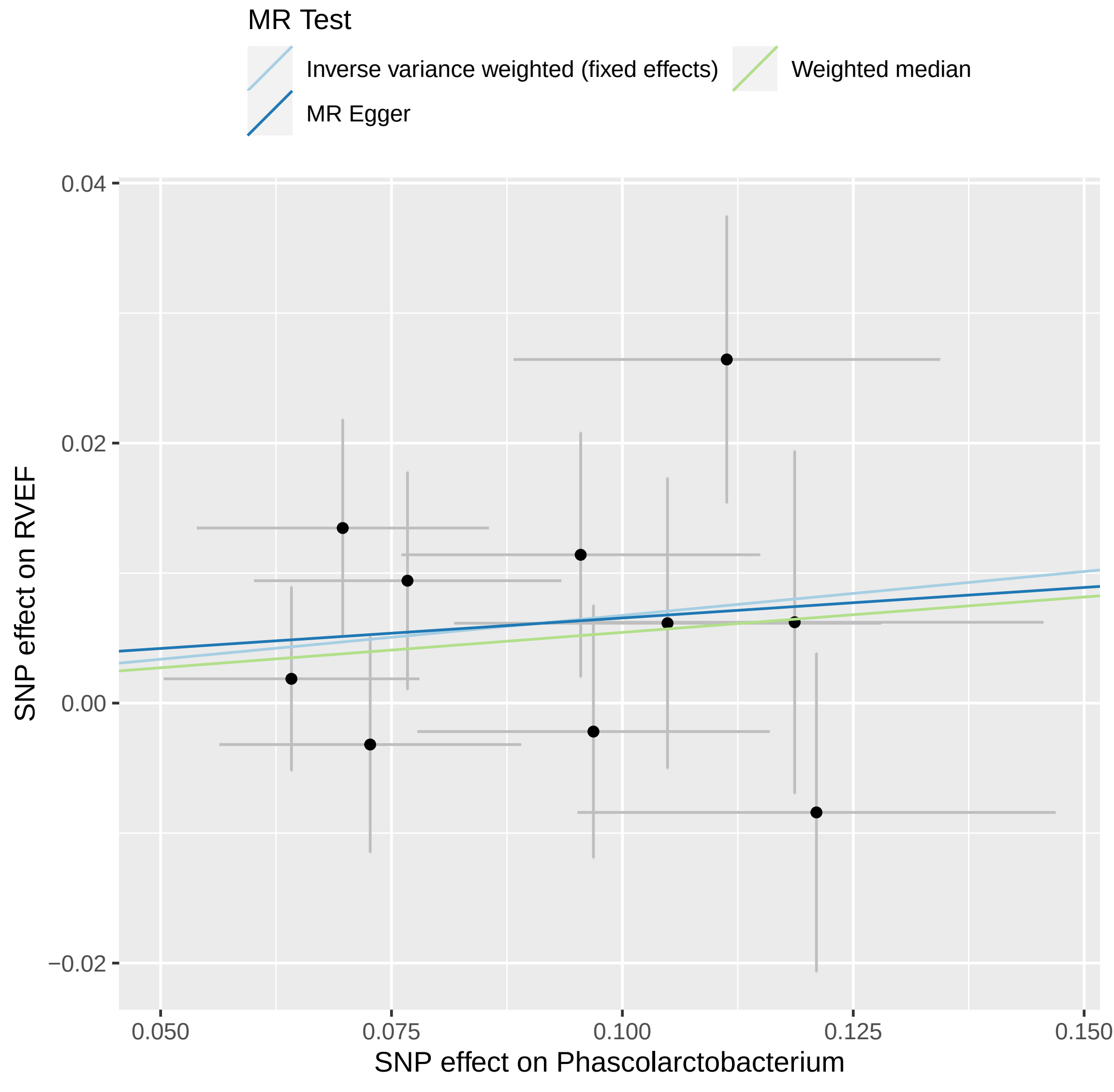
MR Test
Inverse variance weighted (fixed effects)
Weighted median
MR Egger
0.04
0.02
0.00
−0.02
SNP effect on RVEF
0.050
0.075
0.100
0.125
0.150
SNP effect on Phascolarctobacterium

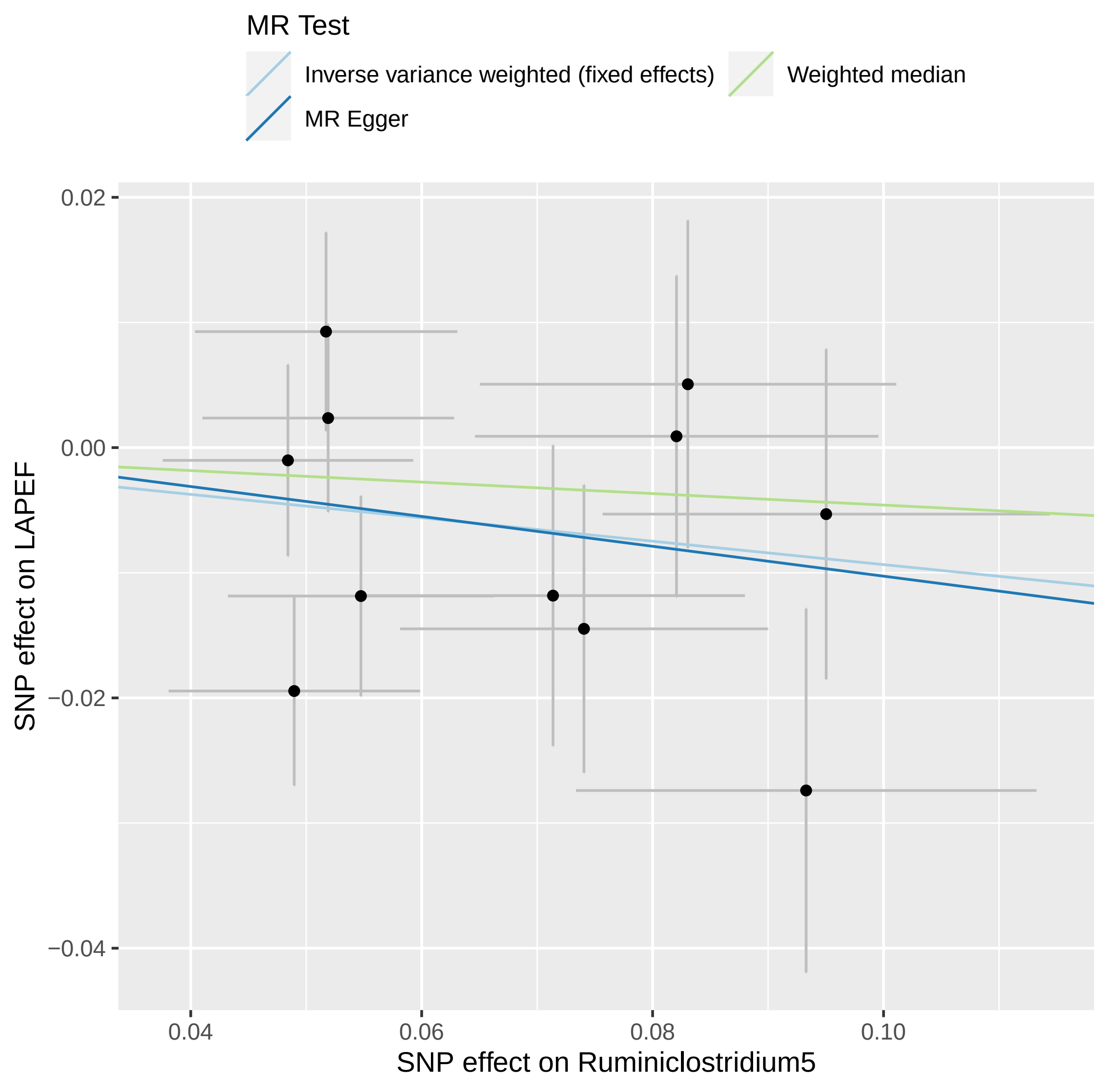
MR Test
Inverse variance weighted (fixed effects)
Weighted median
MR Egger
SNP effect on LAPEF
SNP effect on Ruminiclostridium5
0.02
0.00
−0.02
−0.04
0.04
0.06
0.08
0.10

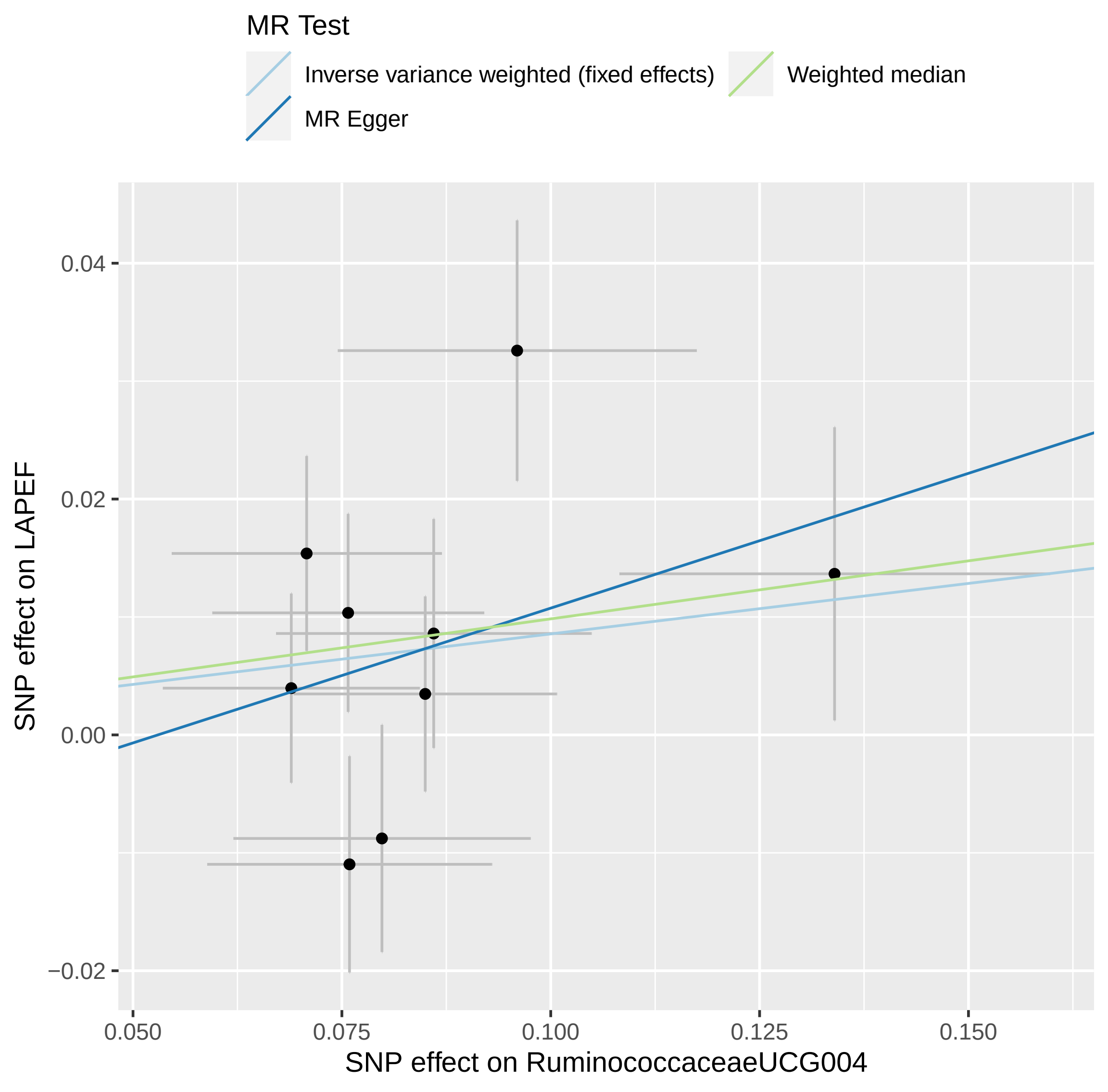
MR Test
Inverse variance weighted (fixed effects)
MR Egger
Weighted median
SNP effect on LAPEF
0.04
0.02
0.00
−0.02
0.050
0.075
0.100
0.125
0.150
SNP effect on RuminococcaceaeUCG004

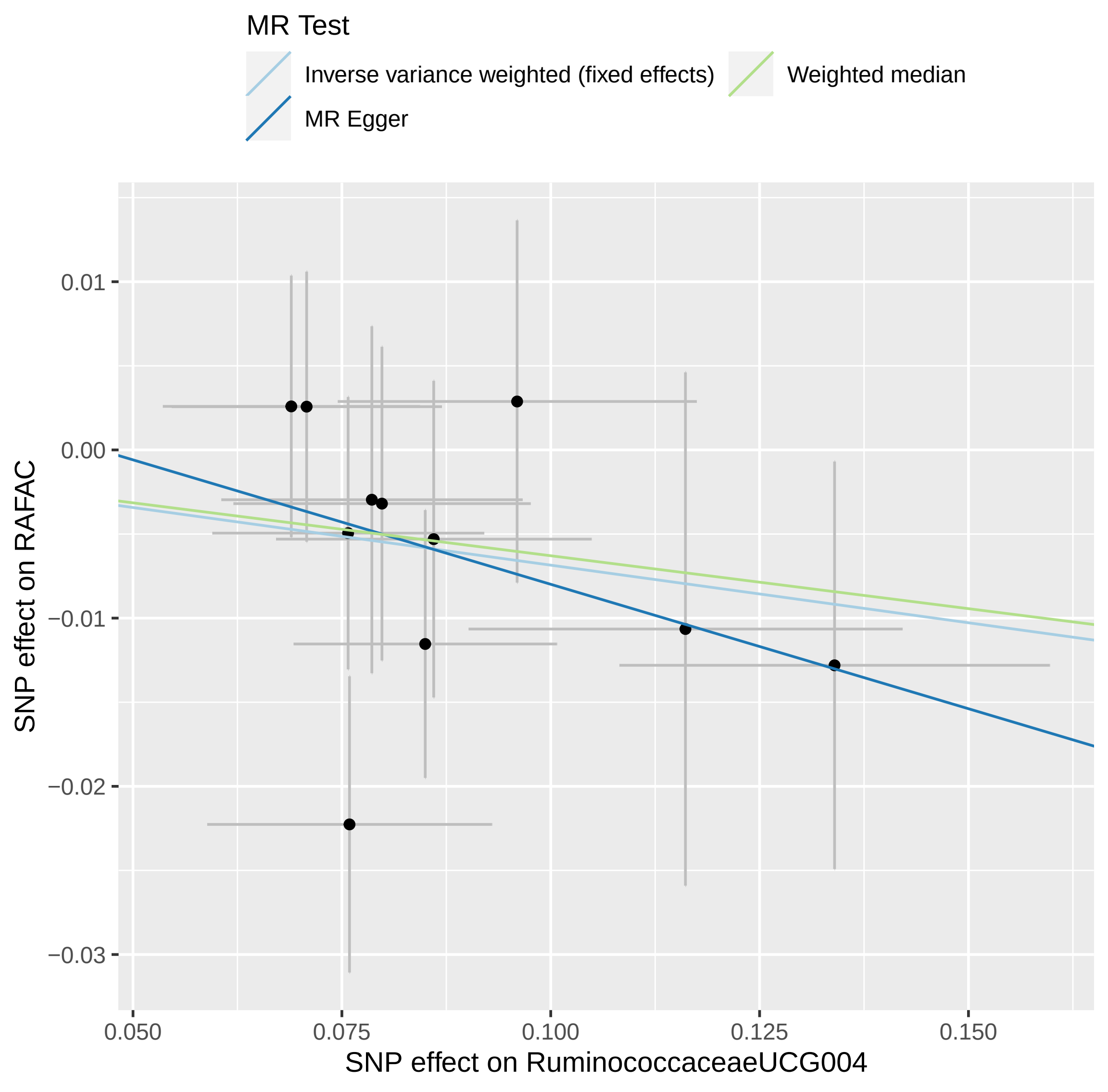
MR Test
Inverse variance weighted (fixed effects)
Weighted median
MR Egger
0.01
0.00
−0.01
−0.02
−0.03
0.050
0.075
0.100
0.125
0.150
SNP effect on RAFAC
SNP effect on RuminococcaceaeUCG004

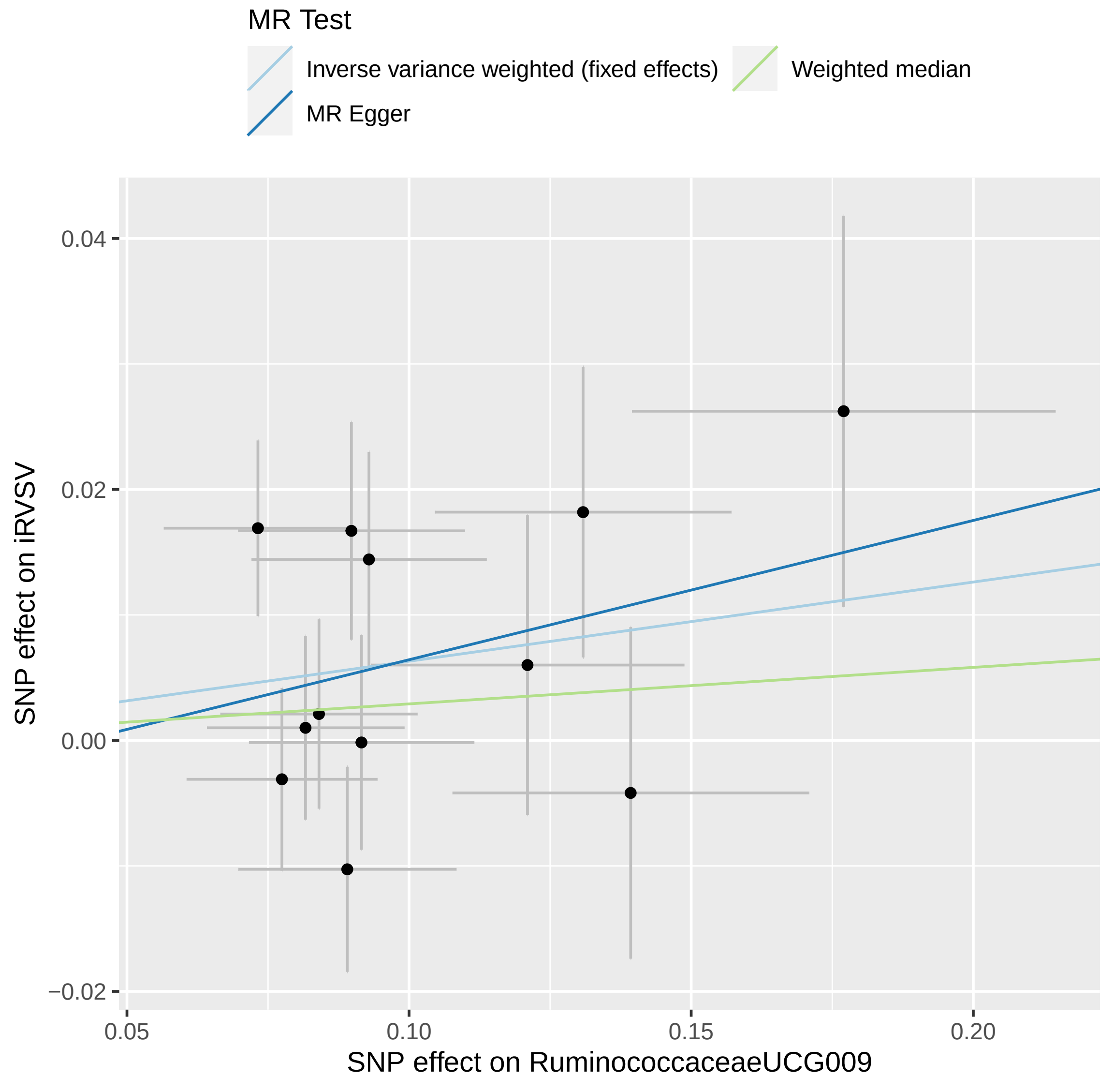
MR Test
Inverse variance weighted (fixed effects)
Weighted median
MR Egger
0.04
0.02
0.00
−0.02
0.05
0.10
0.15
0.20
SNP effect on iRVSV
SNP effect on RuminococcaceaeUCG009

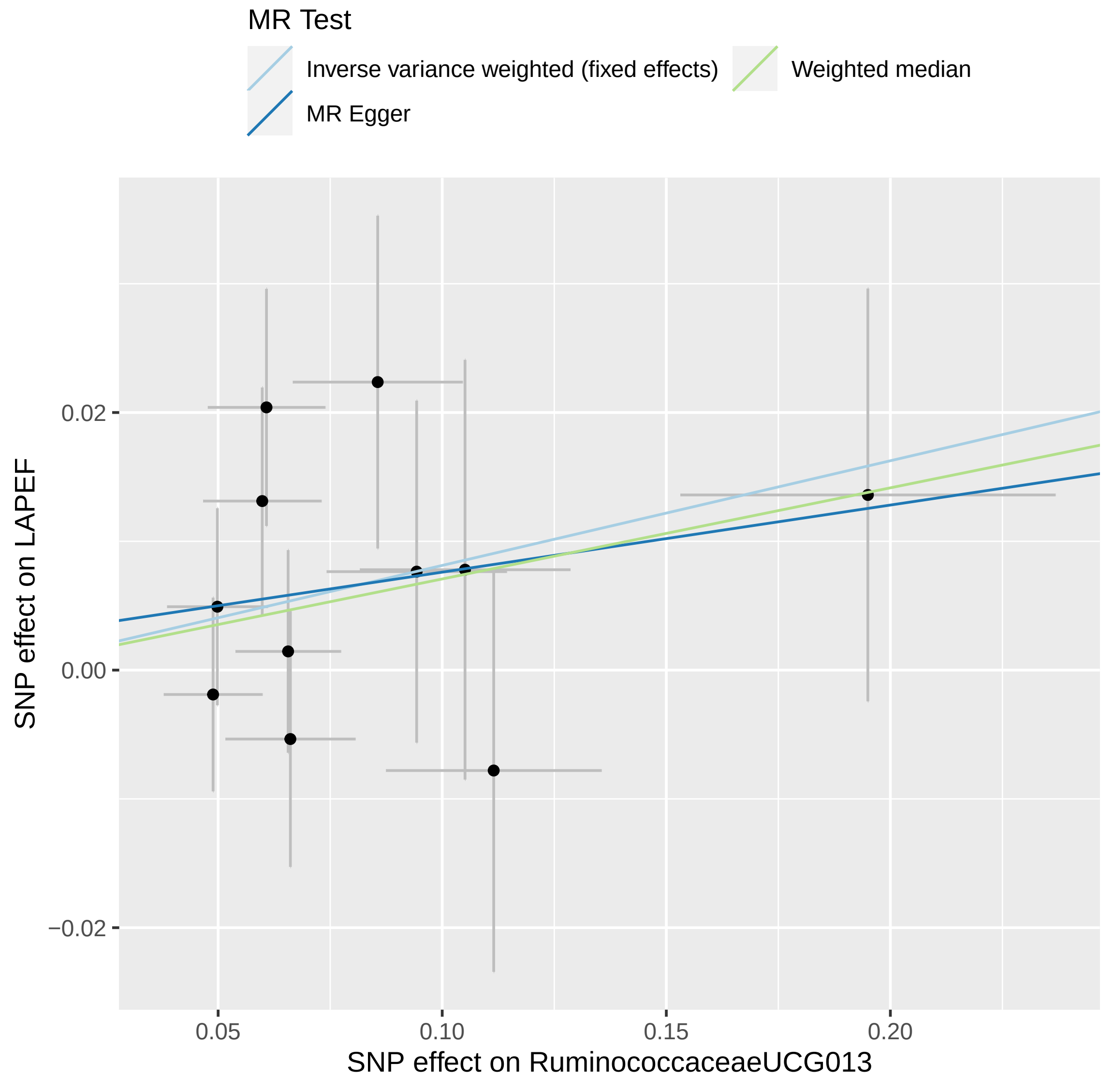
MR Test
Inverse variance weighted (fixed effects)
Weighted median
MR Egger
0.02
0.00
−0.02
0.05
0.10
0.15
0.20
SNP effect on LAPEF
SNP effect on RuminococcaceaeUCG013

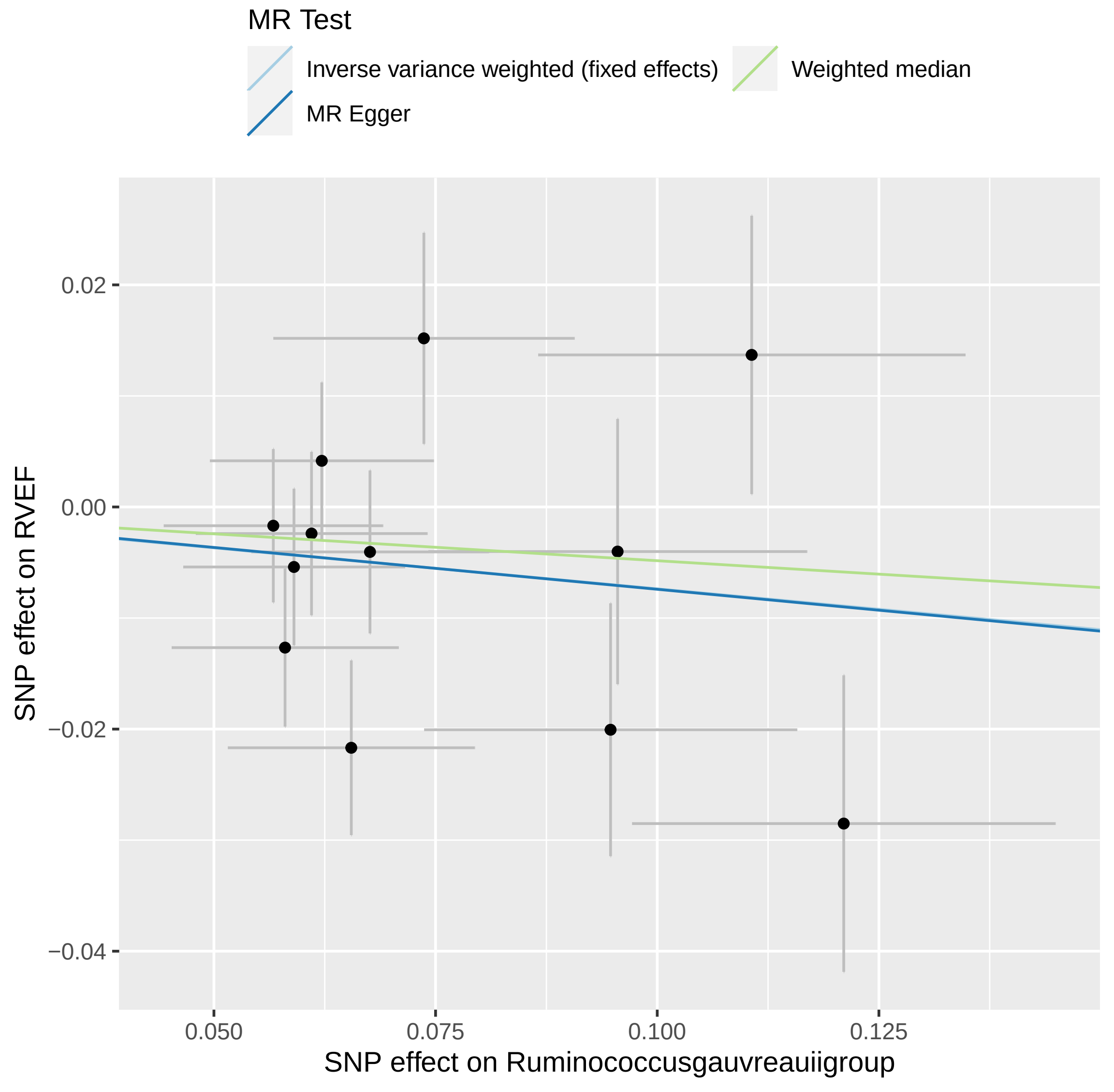
MR Test
Inverse variance weighted (fixed effects)
Weighted median
MR Egger
0.02
0.00
−0.02
−0.04
SNP effect on RVEF
0.050
0.075
0.100
0.125
SNP effect on Ruminococcusgauvreauiigroup

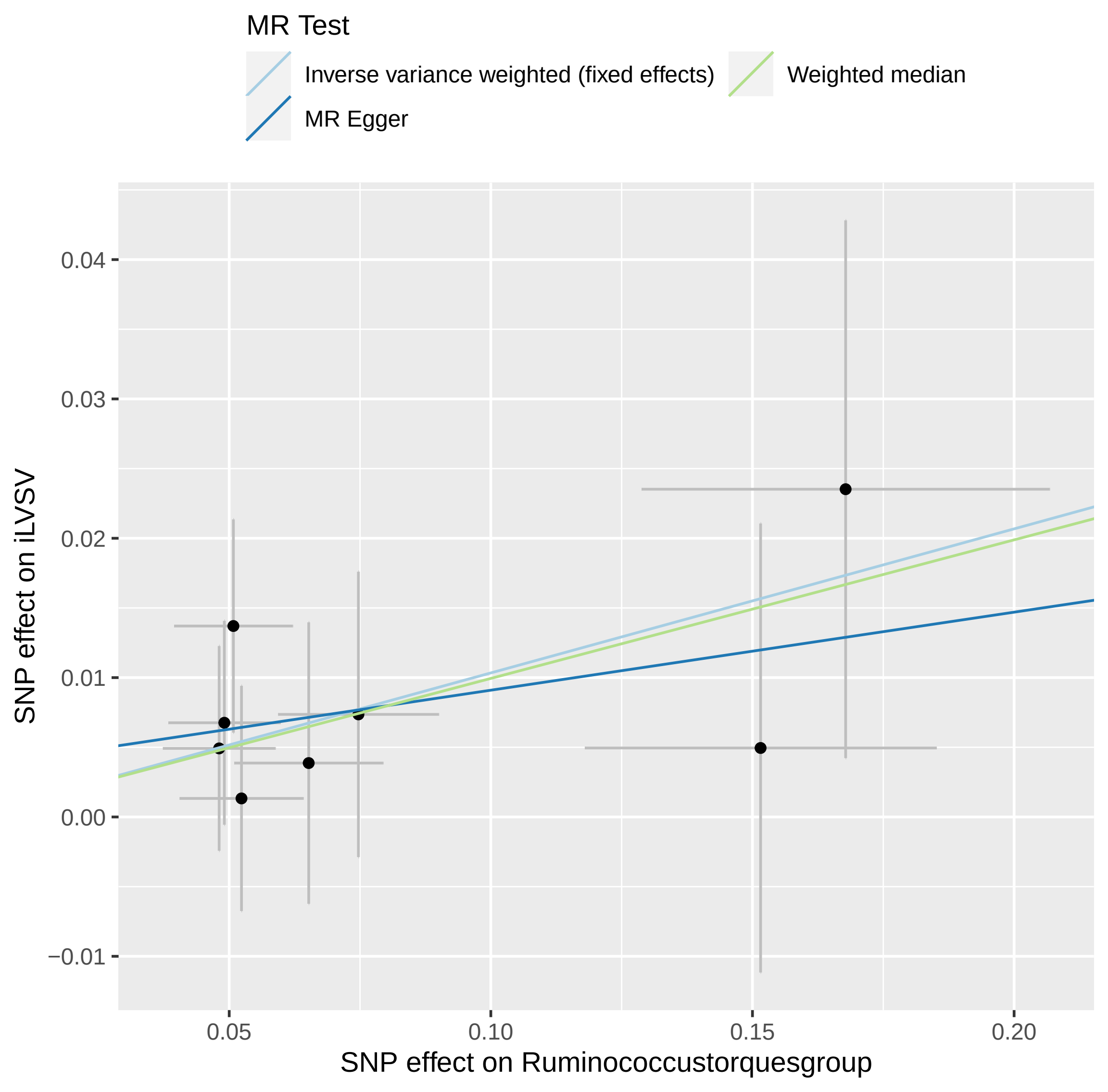
MR Test
Inverse variance weighted (fixed effects)
Weighted median
MR Egger
SNP effect on iLVSV
0.04
0.03
0.02
0.01
0.00
−0.01
0.05
0.10
0.15
0.20
SNP effect on Ruminococcustorquesgroup

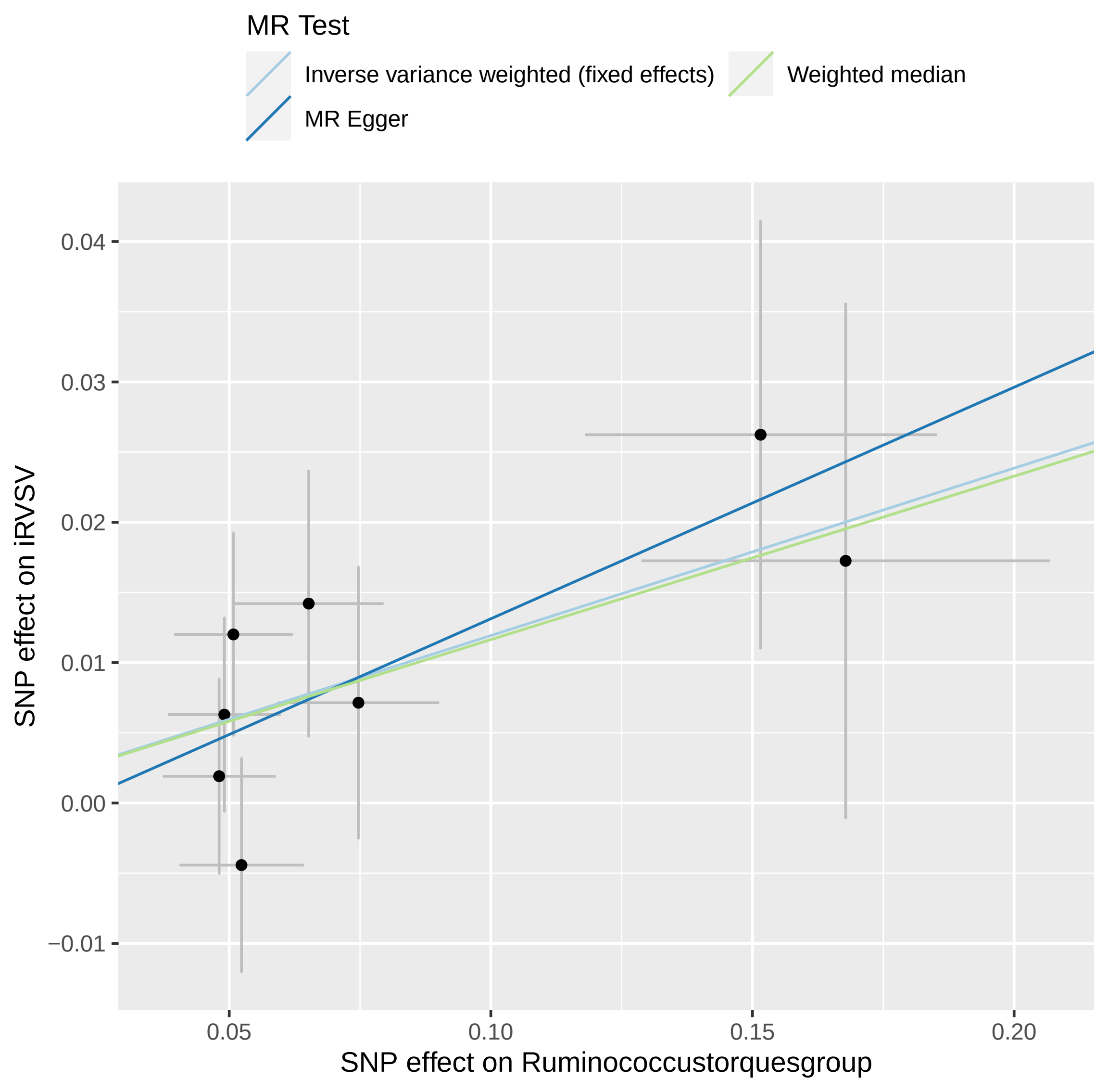

MR Test
Inverse variance weighted (fixed effects)
Weighted median
MR Egger
0.04
0.03
0.02
0.01
0.00
−0.01
0.05
0.10
0.15
0.20
SNP effect on iRVSV
SNP effect on Ruminococcustorquesgroup

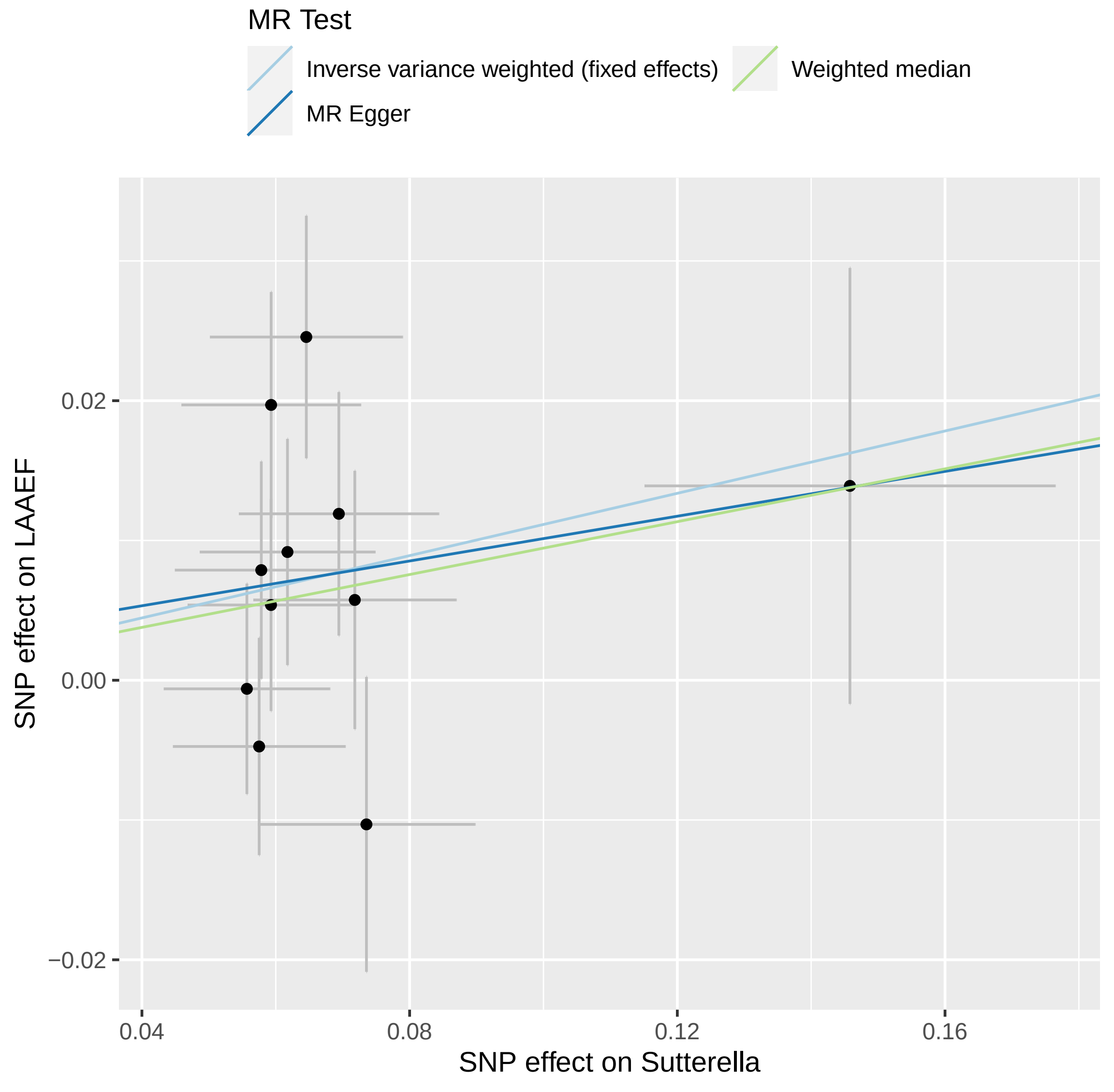
MR Test
Inverse variance weighted (fixed effects)
Weighted median
MR Egger
SNP effect on LAAEF
0.02
0.00
−0.02
0.04
0.08
0.12
0.16
SNP effect on Sutterella

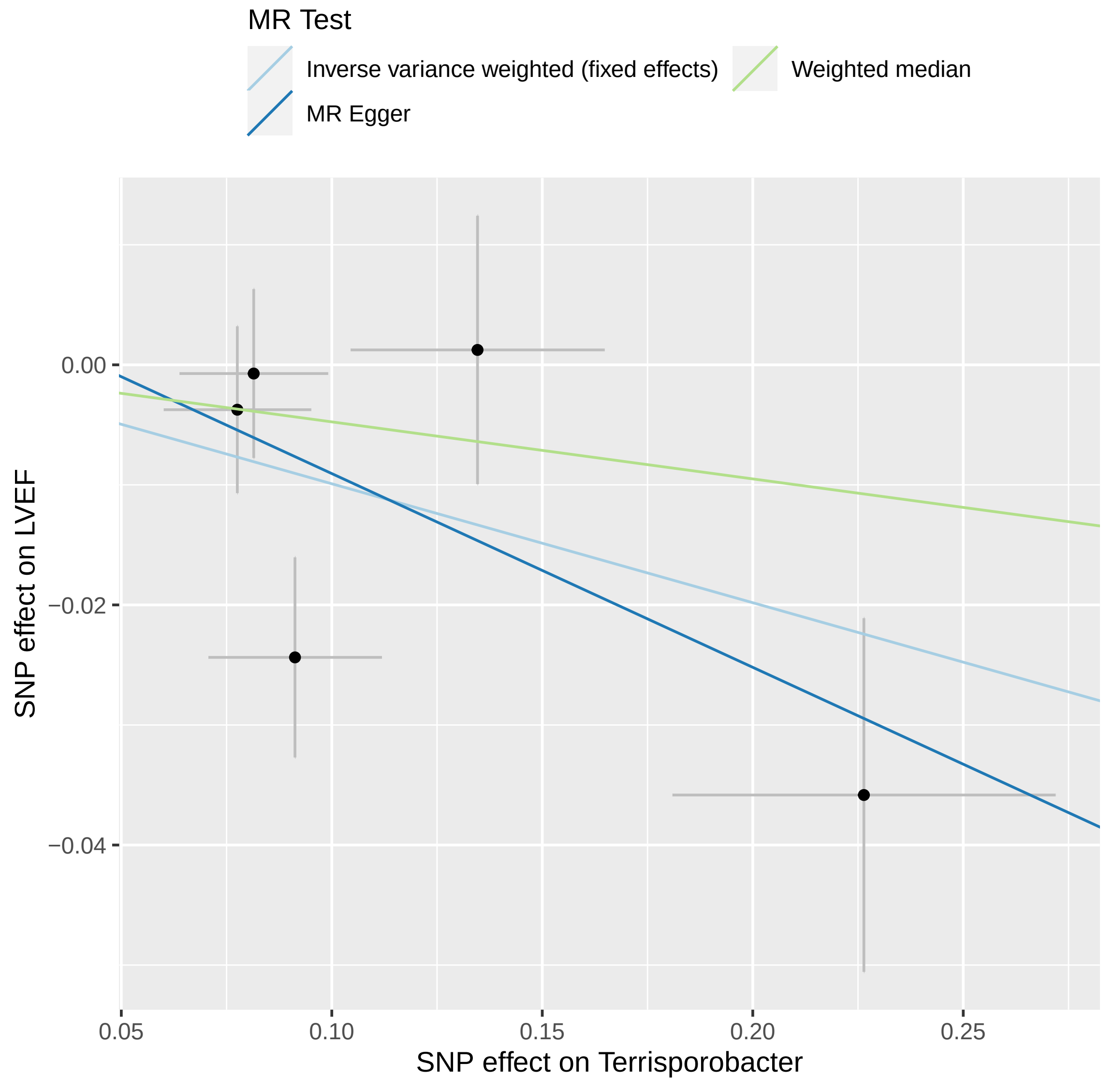
MR Test
Inverse variance weighted (fixed effects)
Weighted median
MR Egger
0.00
−0.02
−0.04
0.05
0.10
0.15
0.20
0.25
SNP effect on LVEF
SNP effect on Terrisporobacter
